# Mild chronic exposure to pesticides alters physiological markers of honey bee health without perturbing the core gut microbiota

**DOI:** 10.1038/s41598-022-08009-2

**Published:** 2022-03-11

**Authors:** Hanine Almasri, Joanito Liberti, Jean-Luc Brunet, Philipp Engel, Luc P. Belzunces

**Affiliations:** 1 INRAE, UR 406 A&E, Laboratoire de Toxicologie Environnementale, 84000 Avignon, France; 2 Department of Fundamental Microbiology, University of Lausanne, Biophore Building, 1015 Lausanne, Switzerland; 3 Department of Ecology and Evolution, University of Lausanne, Biophore Building, 1015 Lausanne, Switzerland

**Keywords:** Environmental sciences, Environmental impact, Microbial ecology

## Abstract

Recent studies highlighted that exposure to glyphosate can affect specific members of the core gut microbiota of honey bee workers. However, in this study, bees were exposed to relatively high glyphosate concentrations. Here, we chronically exposed newly emerged honey bees to imidacloprid, glyphosate and difenoconazole, individually and in a ternary mixture, at an environmental concentration of 0.1 µg/L. We studied the effects of these exposures on the establishment of the gut microbiota, the physiological status, the longevity, and food consumption of the host. The core bacterial species were not affected by the exposure to the three pesticides. Negative effects were observed but they were restricted to few transient non-core bacterial species. However, in the absence of the core microbiota, the pesticides induced physiological disruption by directly altering the detoxification system, the antioxidant defenses, and the metabolism of the host. Our study indicates that even mild exposure to pesticides can directly alter the physiological homeostasis of newly emerged honey bees and particularly if the individuals exhibit a dysbiosis (i.e. mostly lack the core microbiota). This highlights the importance of an early establishment of a healthy gut bacterial community to strengthen the natural defenses of the honey bee against xenobiotic stressors.

## Introduction

Through the production of honey, wax, royal jelly, pollen and venom, honey bees constitute a source of income for more than 600,000 beekeepers in Europe^1, 2^. Honey bees also provide, along with other pollinators, ecosystem and agricultural services through the pollination of wild flora and crops used for human consumption^3–6^. Despite the vital importance of honey bees, the number of managed honey bee colonies has decreased almost worldwide in the last few decades^7–9^. The intense development of agriculture and the appearance of several parasites threatening honey bee health have increased the risk of exposure of honey bees to pesticides and jointly contributed to a steady decline in the number of honey bee colonies^10–13^.

Honey bees can be exposed during foraging to a wide variety of pesticides, such as insecticides, herbicides and fungicides, the three main classes of pesticides used worldwide. These pesticides can be transferred into the colony at residual concentrations via contaminated food (nectar and pollen), which results in the contamination of beehive matrices such as honey, beebread and wax^14–17^. The exposure of honey bees to pesticides could have lethal and sublethal effects. For example, besides their high acute toxicities, neonicotinoid insecticides, such as imidacloprid, are able to impair the cognitive functions, the immune system, the energetic metabolism as well as the detoxification and the antioxidant systems of honey bees^18–22^. Herbicides and fungicides have a low acute toxicity to honey bees. Nevertheless, they can induce adverse sublethal effects. For example, the herbicide glyphosate affects the oxidative balance, the cognitive functions and the larval development of honey bees^23–25^. Fungicides, such as those belonging to the azole family that includes difenoconazole, also have negative effects on honey bees. However, the majority of the studies on toxicity of fungicides has focused on their ability to induce synergistic effects with other pesticides such as pyrethroid and neonicotinoid insecticides^26, 27^.

The effect of pesticides on the host gut microbiota has recently benefited from a growing interest, as the gut constitutes the primary site of interaction with ingested pesticides^28, 29^. The honey bee gut harbors a specific bacterial community of low taxonomic complexity dominated by eight to ten bacterial phylotypes^30–33^, five of which (*Gilliamella apicola*, *Snodgrassella alvi*, *Bifidobacterium asteroides*, *Lactobacillus* Firm-4 and *Lactobacillus* Firm-5) represent the core gut microbiota found in every honey bee worker throughout the planet^34^. Besides these bacterial species, other less abundant species can also be present^31, 35^*.* Increasing evidence suggests that the gut microbiota has a direct effect on honey bee health by defending the host from pathogens^36, 37^, activating the innate immune system^38^, digesting some food components^39, 40^, neutralizing dietary toxins, and biosynthesizing nutrients^41, 42^.

The effects of pesticides on the honey bee gut microbiota were evaluated by several recent studies^2, 29, 43–49^. However, several gaps still exist to understand the effect of the pesticides on the establishment of the gut microbiota. For example, studies on the effect of glyphosate during and after gut colonization were based on the exposure of honey bees to glyphosate at concentrations found in the worst-case scenarios under semi-field experiments^50^. These concentrations were at least five times higher than those encountered by emerged honey bees in the beehive matrices^2, 43, 44, 51^, which did not exceed 342 µg/kg in honey and 58.4 µg/kg in beebread^52–54^. Secondly, recent studies concerning the effect of imidacloprid on gut microbiota did not focus on determining its effect on the early gut colonization^45, 46^. Thirdly, the effect of triazole fungicides on the bee gut microbiota has not been investigated, in spite of their frequent uses in agriculture and their frequent detections in the beehive matrices^55–59^. Moreover, the studies have focused mainly on the effect of a single pesticide on the gut microbiota. Therefore, we lack knowledge about the potential synergistic effects of mixtures of different pesticides, as they often occur in combination in agricultural landscapes and in the beehive matrices^17, 58, 60^.

In the present study, we investigated the effect of chronic exposure at an environmental concentration of 0.1 µg/L to imidacloprid, difenoconazole and glyphosate, individually and in ternary mixture, on the early gut colonization and physiology of newly emerged honey bees. The concentration of 0.1 µg/L was chosen because it corresponds to the lowest concentration at which it was shown that imidacloprid, difenoconazole, and glyphosate can interact when they occur as mixtures^61^. We performed quantitative PCR (qPCR) and 16S rRNA gene amplicon sequencing to assess the effects of the three pesticides on the total load and composition of the gut microbiota. In addition, we quantified the effects of pesticides and gut colonization on the physiological status of honey bees by studying the modulation of five physiological markers: glutathione-*S*-transferase (GST), glucose-*6*-phosphate dehydrogenase (G6PDH), lactate dehydrogenase (LDH), alkaline phosphatase (ALP) and phenoloxidase (POx). These physiological markers are involved in the detoxification system, oxidant defenses, metabolism, and immunity. Therefore, their alterations reflect perturbations in the key physiological functions and in the normal development of honey bees. Overall, our results show that the three pesticides and their ternary mixture at 0.1 µg/L did not affect the total bacterial load and the abundance of core bacterial species. However, they induce changes in the key physiological functions, which become more pronounced when the core gut bacterial community could not establish.

## Results

### Effect of pesticide treatments on gut microbiota loads and community composition

To test the influence of chronic exposure to pesticides on early gut colonization, we performed our experiment on newly emerged honey bees, which we colonized (CL) or not (MD, i.e. microbiota-depleted) with a gut homogenate and exposed for five consecutive days to low concentrations (0.1 µg/L) of pesticides.

**Figure 1:**
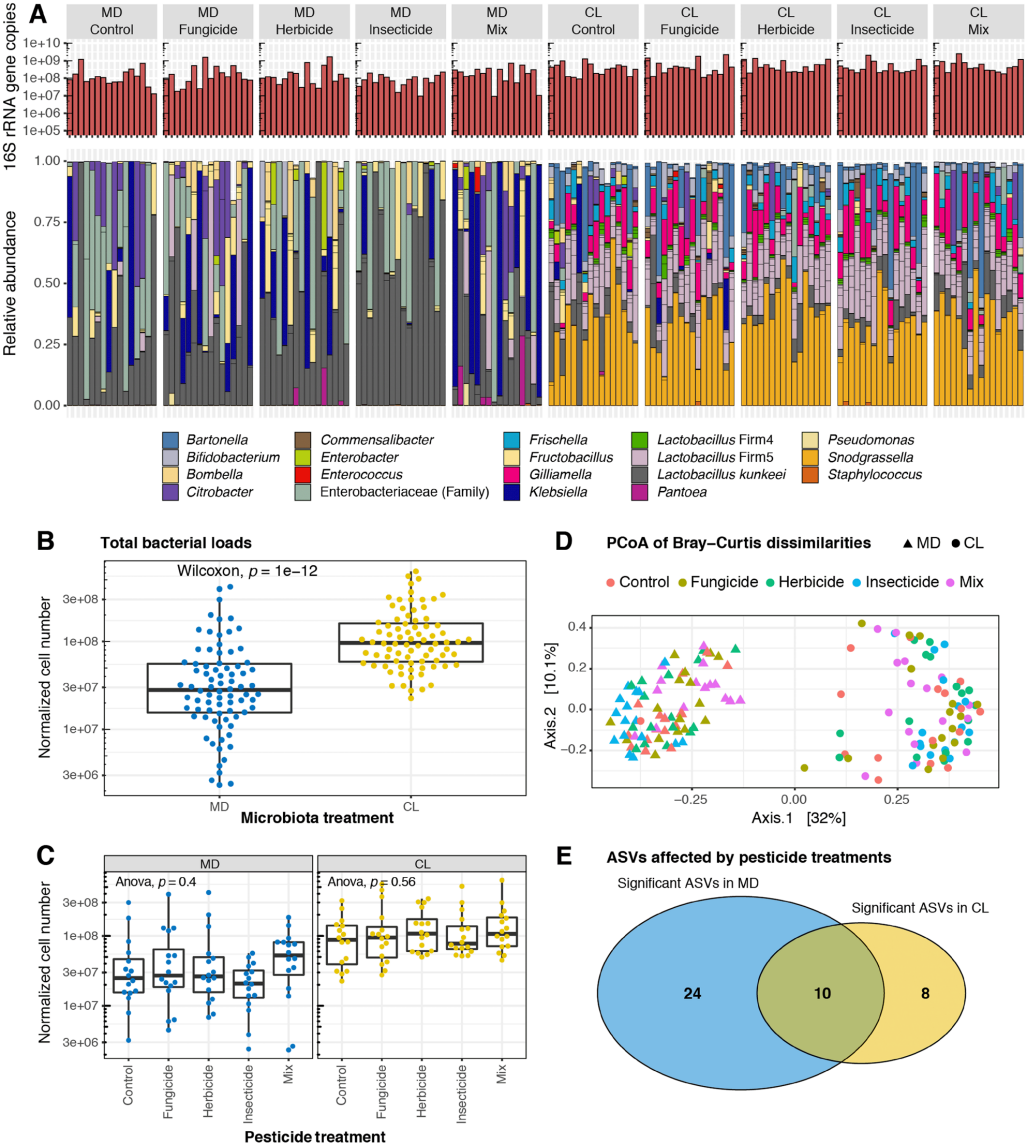
Effects of imidacloprid, difenoconazole and glyphosate on the establishment of gut microbiota. Gut community composition of colonized (CL) and microbiota-depleted (MD) honey bees following exposure to the fungicide (difenoconazole), herbicide (glyphosate) or insecticide (imidacloprid), alone or in a ternary mixture (Mix). (**A**) Stacked bar plots show the relative abundance of gut bacterial genera in control and pesticide-treated honey bees. Each column represents an individual bee. (**B**) Boxplots of total bacterial 16S rRNA gene copies estimated by qPCR in CL and MD bees. (**C**) Boxplots of total bacterial 16S rRNA gene copies in control bees and in bees exposed to the different pesticides, reported separately by gut microbiota colonization treatment. (**D**) Principal coordinate analysis of Bray–Curtis dissimilarities based on amplicon-sequence data normalized by total bacterial loads as obtained by qPCR. (**E**) Venn diagram showing the distribution of the 42 ASVs that differed in abundance between pesticide treatments in permutation ANOVAs in either the microbiota-depleted group, the colonized group, or in both treatment groups. Note that not all these ASVs have significant Tukey post hoc tests in pairwise comparisons between pesticide treatments and controls. See Table S1 and S2 for further details.

Gut bacterial loads were significantly different and up to three times higher in CL compared to MD groups (Wilcoxon rank sum tests, *p* = 1e−12), and while the CL bees harbored a core gut microbiota, MD bees were only colonized by opportunistic bacteria, the majority of which are known to typically reside in the hive environment (Fig. 1A,B). ADONIS tests based on Bray–Curtis dissimilarities and ANOSIM tests based on weighted and unweighted UniFrac distances of MiSeq data normalized by qPCR, showed a statistically significant difference between CL and MD honey bees (*p* = 0.001 for all tests). In addition, principal coordinate analyses revealed that the samples were significantly separated according to the gut colonization status (Fig. 1D, Fig. S1).

Average total bacterial abundances were similar in all comparisons of pesticide-treated and control bees in both CL and MD groups (ANOVA, *p* = 0.4 for MD and *p* = 0.56 for CL) (Fig. 1C). ADONIS and ANOSIM tests did not show any statistical difference between pesticide treatments in CL honey bees (all *p* > 0.05), while a significant pesticide treatment effect was observed in MD bees for all *p* < 0.025). However, size effects were small (ADONIS Bray–Curtis dissimilarities, R^2^ = 0.10; ANOSIM weighted UniFrac, R = 0.076; ANOSIM unweighted UniFrac, R = 0.038) and a significant Betadisper test (*p* = 0.002) suggested that pesticide treatments in MD were not homogeneous in their multivariate dispersions. We nevertheless assessed pesticide treatment effects on 16S rRNA gene copy numbers for all individual ASVs by means of permutation ANOVA. In total, 42 ASVs had significant treatment effects (Fig. 1E), ten of which were common between CL and MD groups. In MD and CL groups respectively, 24 and 10 ASVs had significant pesticide treatment effects (Fig. S2A,B). Only two of these ASVs are commonly found in the honey bee gut^62–64^: *Frischella* and *Arsenophonus*, which were either only affected in CL, or in both CL and MD, respectively. However, post hoc tests showed that these ASVs were not significantly different in pairwise comparisons with control groups (all P > 0.05; Tables S1, S2). Thus, these effects only represented changes in opportunistic transient bacteria of relatively low abundance and facultative presence across individual bee guts.

### Physiological effects of pesticides

**Figure 2:**
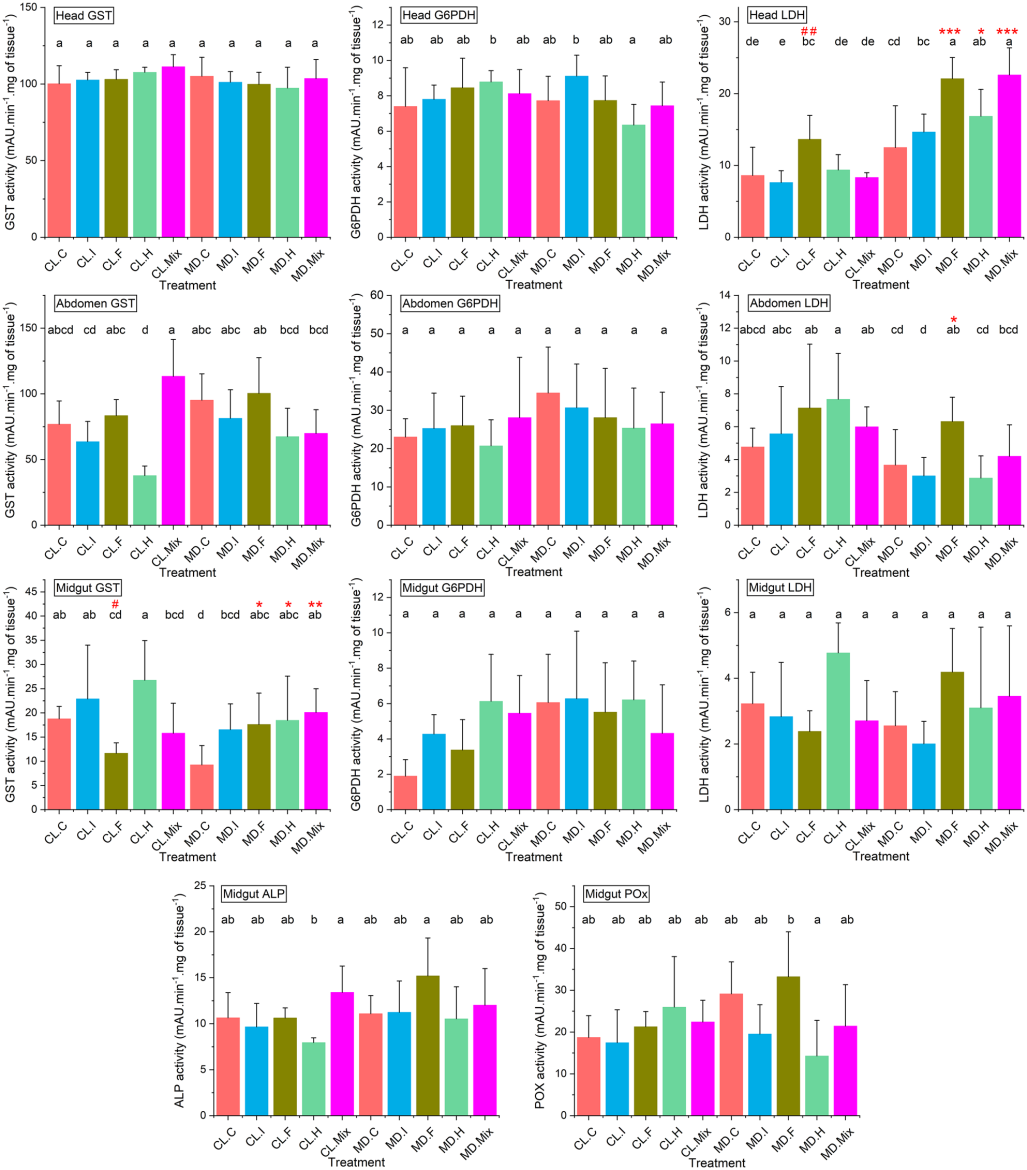
Physiological impacts of pesticides in colonized (CL) and microbiota-depleted (MD) honey bees. For 5 days, colonized (CL) and microbiota-depleted (MD) newly emerged honey bees were fed sucrose solutions containing no pesticides (C, Control), imidacloprid (I, Insecticide), glyphosate (H, Herbicide), difenoconazole (F, Fungicide) or the ternary mixture of these pesticides (Mix) at the concentration of 0.1 µg/L in food. The impact of the exposure to pesticides on the physiology of the surviving honey bees at day five was investigated through an analysis of three common markers in the head, abdomen and midgut (GST, G6PDH and LDH) and two specific markers in the midgut (ALP and POx). ANOVA or Kruskal–Wallis tests were applied to detect significant differences between treatments. Treatments with different letters are significantly different (*p* < 0.05). # indicates a significant difference in the marker levels between colonized honey bees exposed to pesticides and their colonized control (CL.C). * and # indicate a significant difference in the marker levels between microbiota-depleted honey bees exposed to pesticides and their control (MD.C) (* or #: *p* ≤ 0.05; ** or ##: *p* ≤ 0.01; *** or ###: *p* ≤ 0.001).

The effects of imidacloprid, difenoconazole and glyphosate individually and in ternary mixture on the physiological status of honey bees were determined by studying the modulations of five physiological markers in the head, abdomen and midgut (Fig. 2) (Tables S3, S4). In CL honey bees, GST in the head and abdomen, G6PDH in the head, abdomen and midgut, LDH in the abdomen and midgut and ALP and POx in the midgut were not modulated following exposure to pesticides. However, the fungicide increased the activity of LDH in the head (*p* < 0.01) and decreased the activity of GST in the midgut (*p* < 0.05) (Tables S3, S4-A). In MD honey bees, GST in the head and abdomen, G6PDH in the head, abdomen and midgut, LDH, ALP and POx in the midgut were not modulated following exposure to pesticides. However, the fungicide increased the activity of LDH in the head (*p* < 0.001) and abdomen (*p* < 0.05) and GST in the midgut (*p* < 0.05). In addition, the herbicide and the Mix increased the activity of LDH in the head (*p* < 0.05 for Herbicide and *p* < 0.001 for Mix) and GST in the midgut (*p* < 0.05 for Herbicide and *p* < 0.01 for Mix) (Tables S3, S4-B).

### Effect of gut colonization on physiological markers

To detect the potential impact of gut colonization on the physiological status of the experimental bees, we compared enzymatic activities of CL and MD bees exposed to the same pesticide treatments (Fig. 2) (Tables S3, S4, S5). In control unexposed honey bees (MD.Control and CL.Control), GST in the midgut was the only enzyme differently modulated between CL and MD honey bees with a higher activity following gut colonization (*p* < 0.01). In honey bees exposed to the insecticide, LDH in the head and abdomen were modulated differently based on the gut colonization status. LDH activity was lower in the head and higher in the abdomen of CL compared to MD honey bees (*p* < 0.001 for head LDH and *p* < 0.05 for abdomen LDH). In honey bees exposed to the fungicide, the activity of LDH in the head was lower in CL honey bees compared to MD (*p* < 0.01). In honey bees exposed to the herbicide, the activities of G6PDH in the head and LDH in the abdomen were higher in CL compared to the MD honey bees (*p* < 0.05 for head G6PDH and *p* < 0.01 for abdomen LDH). However, the activity of LDH in the head was lower in CL honey bees (*p* < 0.05). In honey bees exposed to the ternary mixture, the activities of GST in the abdomen was higher in CL honey bees compared to the MD ones (*p* < 0.05) and the activity of LDH in the head was lower in CL honey bees (*p* < 0.001).

**Figure 3:**
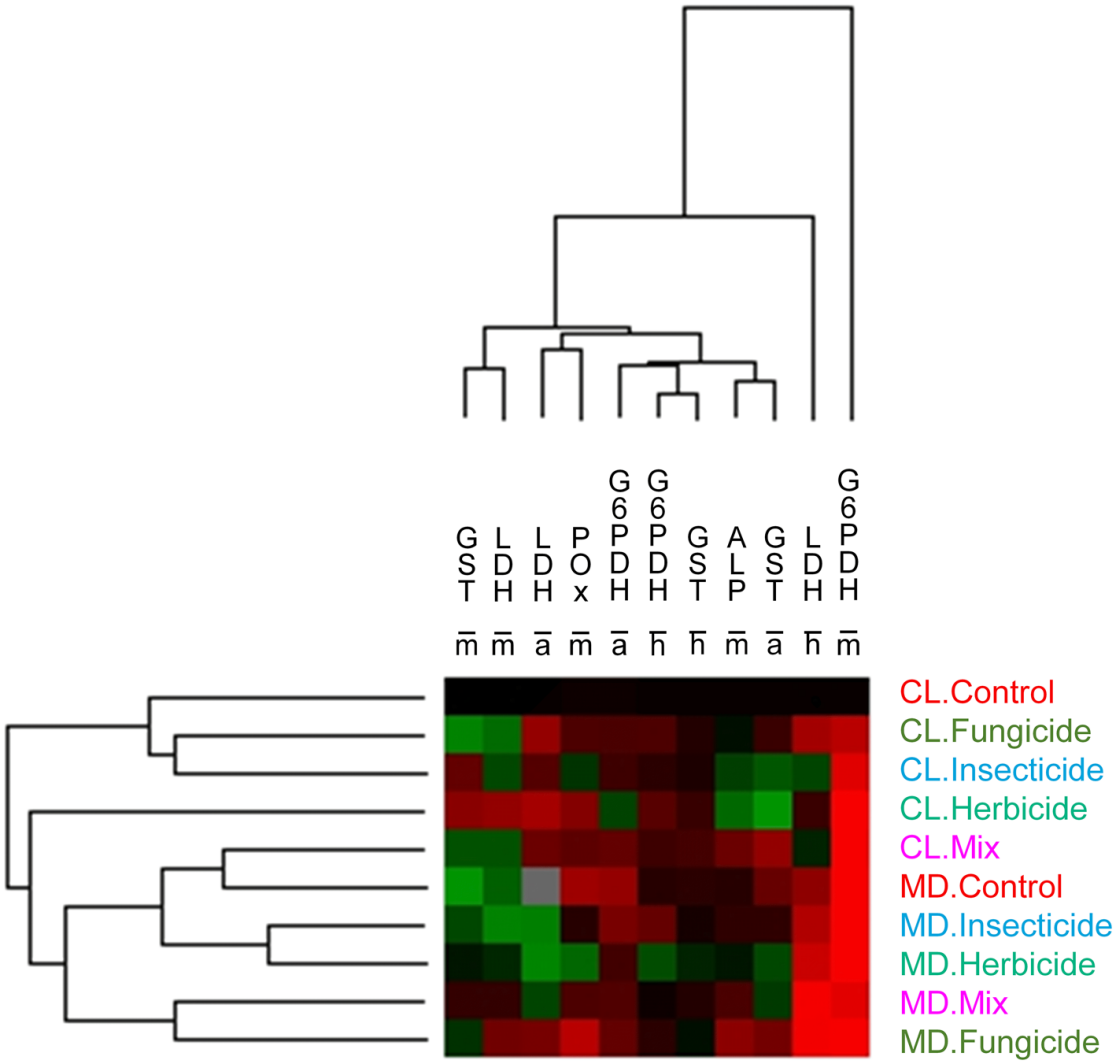
Effects of the pesticides and gut colonization on the physiological state of honey bees. The levels of physiological markers in colonized (CL) and microbiota-depleted (MD) newly emerged honey bees exposed or not to pesticides was submitted to a cluster analysis to assess, with an integrative approach, the effect of pesticide treatments and gut colonization status on the physiological markers analyzed in the head (h), abdomen (a) and midgut (m). Colonized (CL) and microbiota-depleted (MD) honey bees were fed sucrose solutions containing no pesticides (Control), imidacloprid (Insecticide), glyphosate (Herbicide), difenoconazole (Fungicide) or the ternary mixture (Mix) at concentration of 0.1 µg/L in food. The distance measured was Euclidian distance with UPGMA as the linkage rule for clusters. Data normalization was required to convert the mean of each treatment to the rate of variation compared with the average of the control (CL.Control). The intensity of modulation is illustrated by the range of colors, with green and red indicating respectively a decrease and an increase of the mean enzymatic activity in each treatment, by comparison with the mean value in the CL.Control. Black indicates no change by comparison with the mean value of CL.Control.

The hierarchical cluster analyses showed a tendency of the pesticide treatments to group according to the gut colonization status. In addition, physiological markers were not grouped together by body compartment. Only head LDH and midgut G6PDH were distant from the other enzymes, due to an overall increase of their activities in all treatments compared to those of CL.Control (Fig. 3, Fig. S3).

### Effect of exposure to pesticide and gut colonization on honey bee survival

The survival rate was recorded during the 5 days of exposure to pesticides. No differences in survival rates had been detected between the unexposed honey bees (CL.Control and MD.Control) and the bees exposed to the different pesticide treatments. In addition, no differences in survival rates had been detected between the CL and the MD honey bees following exposure to a common pesticide treatment (Fig. S4).

### Effect of exposure to pesticide and gut colonization on food consumption

The influence of pesticide treatments and gut colonization on the feeding behavior of honey bees was followed by measuring the daily food consumption. Honey bees exposed to all pesticide treatments consumed an equal amount of food. No differences in food consumption was observed between CL and MD honey bees exposed to a similar pesticide treatment (Fig. S5).

## Discussion

Numerous studies have revealed an impact of several pesticides on the gut microbiota of honey bees. The majority of these studies have focused on the effect of a single pesticide and used concentrations higher than those generally encountered by honey bees under field conditions. Bees are unlikely to be exposed to single pesticides while feeding in the beehive or foraging, as herbicides, insecticides and fungicides are often used in combination to improve crop yields and are detected simultaneously in the beehive matrices^17, 65–67^. It is thus important to assess the potential synergistic effects between different combinations of agrochemicals. The pesticides considered in this study belong to the three main classes of pesticides used worldwide. Imidacloprid is a neonicotinoid insecticide that disrupts the nervous system of insects by acting as an agonist to acetylcholine receptors^68^. It was detected at concentrations of 1.35 µg/kg in pollen and 0.14–0.275 µg/kg in honey^67, 69^. Glyphosate [N-(phosphonomethyl)glycine] is among the most widely used pesticides^70^, it is a herbicide that prevents the production of essential amino acids in plants through the inhibition of the enzyme 5-enolpyruvylshikimate-3-phosphate synthase (EPSPS) present also in some microorganisms^71, 72^. Glyphosate residues were detected in beebread at concentrations ranging between 52.4 and 58.4 µg/kg and in honey at concentrations ranging between 17 and 342 µg/kg^52, 54^. Difenoconazole is an ergosterol biosynthesis inhibitor fungicide, it inhibits the lanosterol 14-α-demethylase leading to the depletion of ergosterol which is a vital constituent of the fungi cell wall^73^. It was frequently detected in honey and pollen at concentrations of 0.6 and 43 µg/kg, respectively^55^.

Our study provides a first attempt to determine the effects of several pesticides individually and in mixture at environmental realistic concentrations on the early establishment of the gut microbiota and key physiological functions in honey bees. Our study shows that chronic exposure to low doses of imidacloprid, difenoconazole and glyphosate, individually and in ternary mixture, can directly affect the physiology of honey bee workers without disrupting their core gut microbiota. Notably, the differences between the effects of the pesticide treatments were more marked in microbiota-depleted bees than in colonized bees. In addition, we found that the overall effects significantly differed between microbiota-depleted and colonized bees, suggesting that the core gut microbiota plays a role in the bees’ physiological resilience to the action of pesticides. However, although no effect of pesticides on the establishment of the microbiota in emerging bees was observed in this study, it is noteworthy that long term exposure to pesticides, such as neonicotinoids, may impair the microbiota already established in older bees^74^.

To our knowledge, this is the first study about the effect of triazole fungicides on the establishment of the honey bee gut microbiota. However, previous studies on other organisms and ecosystems revealed the capacity of azole fungicides to disrupt the gut microbiota of female rats, and to stimulate or inhibit soil bacterial proliferation depending on the fungicide active ingredient^75, 76^. Concerning pesticide mixtures, data on their potential effects on honey bee gut microbiota are lacking despite the high occurrence of pesticides as a mixture in the beehive residues. Therefore, further studies are needed to understand the effect of azole fungicides and the different pesticide combinations on the honey bee gut microbiota during and after gut colonization.

Recent studies have focused on the effects of oral exposure to glyphosate on the honey bee gut microbiota, showing changes in community structure, with marked shifts in total abundance of specific symbionts (*Snodgrassella* and *Lactobacillus* Firm-4 in particular)^2, 43, 44, 51^. In these studies, the glyphosate concentrations used ranged from 0.01 mM (1691 µg/L) to 1 mM (169 070 µg/L), based on concentrations found in water sources^77–79^ and in a study performed under semi-field conditions in which hives were placed in insect-proof glasshouse^50^. Thus, because the highest concentration measured in honey and pollen is at least fivefold lower, these concentrations could be considered the worst-case realistic exposure levels^52–54, 80, 81^. Hence, the absence of an effect of glyphosate on the early gut colonization in our study could be linked to the low residual concentration (0.1 µg/L) to which newly emerged honey bees were exposed. This hypothesis is confirmed by the results of Motta and Moran^2^, who have found that the effects of glyphosate on gut microbiota increased with the concentration, with an absence of effects at 0.01 mM (1.69 mg/L). This dose–response relationship is also confirmed by Dai et al.^82^ who found a high impact of glyphosate on the gut microbiota of honey bee larvae exposed at the highest dose of 20 mg/L but not at 0.8 and 4.0 mg/L. Consequently, the glyphosate concentration used in our study might be considered too low to induce any effect. However, at this concentration, glyphosate can induce a chronic toxicity to bees through lethal and physiological effects, especially when it is associated with other pesticides^61, 82^.

The absence of an effect of imidacloprid on early gut colonization in our study is consistent with the results of Raymann et al.^45^, who reported an absence of effect on established gut microbiota following a 3 days exposure to 500 µg/L of imidacloprid. However, neonicotinoids may have a negative impact on the gut microbiota when honey bees are exposed to them for a long exposure period. This is supported by two independent studies. In the first study it was observed that the relative abundance of *Lactobacillus* spp. and *Bifidobacterium* spp. significantly decreased in winter and summer worker bees exposed to imidacloprid at 3.5 µg/kg for 18 days^46^. In the second study, a decrease in the absolute abundance of total bacteria. *Lactobacillus* Firm-5, and *Bombella apis* was found after a 7-day chronic exposure of middle-aged honey bees to thiacloprid, an insecticide belonging to the neonicotinoid family, at 200–2000 µg/L^48^.

In our experiment, the bacterial load was three-fold lower in microbiota-depleted bees than in colonized ones. This difference appears much smaller than the 100-fold difference reported in a study that explicitly quantified the bacterial loads in colonized and non-colonized bees^40^. This was of no particular concern for our study because our main aim was not to quantify physiological differences between MD and CL bees but rather the effects of exposures to pesticides.

There was no variation in the cumulated food consumption between the different pesticide treatments in colonized and microbiota-depleted honey bees. Therefore, bees had ingested an equal amount of pesticides in all treatments and these pesticides did not exhibit any attractive or repellent effect. The chronic exposure to the three pesticides and their ternary mixture did not affect bees’ survival. This may be due to the relatively short duration of exposure of 5 days. In a previous study, an exponential increase in mortality was observed 6–8 days after the beginning of the chronic exposure to the same pesticides in winter honey bees^61^. Gut colonization did not elicit a direct effect on bee survival, as both colonized and microbiota-depleted honey bees exhibited a similar survival rate. The absence of a negative effect on survival agrees with Zheng et al.^83^.

To assess the physiological effects induced by pesticides, we investigated the activity of GST, G6PDH and LDH in the head, abdomen (with the intestinal tract removed) and midguts, and the activity of ALP and POx in the midguts. GST and G6PDH are involved in the detoxification process and in the protection against oxidative stress. GST acts through the reduction of hydroperoxides into alcohols and the conjugation of reduced glutathione (GSH) to xenobiotics such as pesticides^84^. G6PDH yields NADPH, which is essential for cytochrome P450 (CYP 450) catalysis^85^. NADPH is also involved in the anti-oxidative defenses through the regeneration of GSH from its oxidized form^86^. LDH is involved in the energy metabolism in insects, precisely in the glycolytic pathway. Under anaerobic conditions, it catalyzes a reversible reduction of pyruvate into lactate using NADH as a cofactor^87^. ALP is a metabolic enzyme involved in the adsorption and transport mechanism through the gut epithelium; it is also involved in the immune response^88–90^. POx plays a role in the constitutive immune response of insects through catalysis of the melanization process involved in sealing wounds and encapsulation of foreign bodies such as parasites and pathogens^91, 92^.

We found no major effect of pesticides on the oxidative system, metabolism and immunity of colonized honey bees. Only the fungicide increased the LDH activity in the head and decreased the GST activity in the midgut. The short duration of exposure to imidacloprid and glyphosate may explain the absence of an effect of these two pesticides, which have both been reported in previous studies to induce oxidative stress in honey bees^21, 24, 93^.

Our results show differences in the modulation of physiological markers between colonized and microbiota-depleted honey bees exposed to the same pesticide treatments. They suggest that, in the presence of the core gut microbiota, bees have increased resilience to oxidative stress and improved detoxification of xenobiotics. Therefore, in the long-term, the core gut microbiota could decrease the toxicity of the ingested pesticides and increase host survival. This was reflected by a higher activity in CL bees of G6PDH in the head of herbicide treated bees, GST in the abdomen of ternary-mixture-treated bees, and GST in the midgut of control bees. G6PDH and GST are both involved in the detoxification process and fight against oxidative stress^85, 94, 95^. The role of specific core gut members in detoxification and oxidative balance was previously documented through the upregulation of esterase FE4-like, cytochrome Cyp6bd1 and multicopper oxidase 1 (MCO1) genes in *F. perrara* compared to honey bees inoculated with *S. alvi*. The esterase FE4-like gene is involved in the detoxification process and development of insecticide resistance in peach-potato aphids^96^. Moreover, this enzyme is involved in the oxidative response during adverse stress such as exposure to imidacloprid and paraquat in *Apis cerana cerana*^97^. The Cyp6b family plays a significant role in the development of insecticide resistance in *Culex pipiens pallen*^98^. MCO1 is indirectly involved in the regulation of oxidative stress through the catalysis of the oxidation of ferrous iron (Fe^2+^) into ferric iron (Fe^3+^)^99^.

Early gut colonization of honey bees did not seem to have an effect on the melanization response because POx had a similar activity in colonized and microbiota-depleted honey bees. However, we did expect an increase in the activity of this enzyme in CL bees due to the upregulation of serine protease activation cascade and serine protease inhibitors in the presence of *Frischella perrara*, which was only detected in CL bees. This bacterium, through the activation of the previously cited genes, leads to the formation of the scab phenotype that corresponds to a melanization process in the pylorus and hence should involve activation of POx^63^. The discrepancy observed between our results and those obtained by Emery et al.^63^ might be explained by differences in the types of gut colonization performed in the two studies. In the study of Emery et al.^63^, bees were colonized only with *F. perrara* or *Snodgrassella alvi* while, in our study, bees were colonized by the original worker microbiota in which competitive and homeostatic processes may occur. Thus, the presence of competitive flora might modulate the capability of *F. perrara* to modulate the melanization cascade.

The differences in LDH activity between colonized and microbiota-depleted honey bees were tissue-specific. For a same pesticide treatment, with imidacloprid or glyphosate, head LDH activity was lower in colonized honey bees than in microbiota-depleted ones, while abdomen LDH activity was higher in colonized honey bees. The existence of differences in the LDH activity between colonized and microbiota-depleted honey bees is another evidence that the gut microbiota influences the host metabolism^39, 42, 83, 100^.

The hierarchical cluster analyses showed a distinct physiological status in honey bees colonized with the normal gut microbiota compared to those deprived of their core microbial species. The microbiota effects on host physiology were not limited to the midgut but they were also present in the head and abdomen. Therefore, it is becoming increasingly clear that the microbiota exerts a systemic effect on its host rather than being localized in the gut. This is also reflected by the interference of the gut microbiota with the abundance of apidaecin in the honey bee haemolymph^101^, and its suggested effects on the honey bee nervous system by modulating the sugar intake through the increase in insulin sensitivity^83^.

In conclusion, our work showed that exposure to pesticide concentrations far lower than previously tested, individually and in ternary mixture, did not affect the early gut bacterial colonization by core members of the honey bee microbiota. However, these exposures directly induced changes in physiological markers broadly associated with honey bee health. These results are thus in agreement with previous studies raising concern about widespread pesticide use in agricultural landscapes damaging pollination services and insect populations more in general.

## Materials and methods

### Materials

Honey bees were obtained from five healthy looking colonies located in the experimental apiary of *Abeilles & Environnement* (Bees & Environment) Research Unit at Avignon INRAE Research Centre (South of France). To rear newly emerged honey bees lacking core gut bacteria, tan-colored dark-eyed pupae were removed from the brood combs using sterile forceps and were allowed to emerge in sterile plastic boxes. The plastic boxes were kept for 2 days at 34.5 ± 2 °C under 85% relative humidity. Newly emerged honey bees were then distributed in sterile plastic cages (6 × 8.5 × 10 cm; 30 bees per cage) by mixing bees from all five hives into each cage. Imidacloprid, difenoconazole and glyphosate were purchased from Dr. Ehrenstorfer GmbH (Augsburg, Germany) (Table 1).

**Table 1:** Characteristics of the pesticides.

Active substance	CAS number	Purity (%)	Pesticide family
Imidacloprid	138261-41-3	98	Neonicotinoid insecticide
Difenoconazole	119446-68-3	98	Triazole fungicide
Glyphosate	1071-83-6	98	5-Enolpyruvylshikimate-3-phosphate synthase inhibitor herbicide

### Exposure to pesticides

Imidacloprid, difenoconazole and glyphosate were used either alone or in a ternary mixture (Mix) at a final concentration of 0.1 µg/L, which is consistent with the residual concentrations of these pesticides in honey^53, 55, 69^. Just before the beginning of the experiments, stock pesticide solutions were prepared in sterilized water (glyphosate) or DMSO (imidacloprid and difenoconazole). From stock solutions, working 10X pesticide solutions were prepared in 1% (v/v) DMSO and were stored at -20 °C up to their extemporaneous dilution in 66.7% sterilized sucrose solution to obtain feeding solutions containing 0.1 µg/L pesticides, 60% (w/v) sucrose and 0.1% (v/v) DMSO. The accuracy of the pesticide concentration was checked by dosing the 10X and feeding solutions according to two analytical methods^102, 103^. For each solution RSD < 7%.

### Preparation of gut homogenate for bacterial inoculation of newly emerged bees

To obtain a single gut homogenate for subsequent bacterial colonization of experimental bees, we dissected the guts (hindgut, ileum and midgut, excluding the crop) of 15 forager honey bees, collected at the entrance of five hives, and homogenized them in sterile PBS (1 mL sterile PBS per gut) using the Qiagen^®^ TissueLyser II (30 Hz for 3 periods of 10 s, at 10 s intervals). The homogenates were then pooled and 1/3rd volume of 80% (v/v) glycerol solution was added to produce a final glycerol concentration of 20% (v/v). The homogenate was aliquoted in sterile Eppendorf tubes and stored at − 80 °C until use.

### Experimental colonization of honey bees and exposure to pesticides

Twenty-four hours after the emergence of adult honey bees, the bees present in half of the experimental cages were colonized from the gut homogenate. To achieve this, a 2-mL vial containing 300 µL of the gut homogenate was added to each cage. In the vial, the gut homogenate was diluted ten times with sterile PBS and mixed (1:1, v/v) with 50% (w/v) sterile sucrose solution. The bees were allowed to feed on this solution for 3 days. Microbiota-depleted honey bees (MD) were kept under the same conditions and fed sterile sucrose solution, diluted in PBS (1:1, v/v) lacking the gut homogenate.

Newly emerged bees, colonized (colonized bees; CL) or not (microbiota-depleted; MD) with the gut homogenate, were exposed or not (control) to the three pesticides (imidacloprid, glyphosate and difenoconazole) either individually or in a ternary mixture (Mix), in a two by four factorial design. At the beginning of the fourth day post emergence, honey bees were fed for 5 consecutive days with a sterile sucrose solution (60% (w/v)) containing or not (Control, C) pesticides and 0.1% (v/v) DMSO. Four replicates of 30 honey bees per treatment modality were made. During the experiment, the bees fed ad libitum with 60% (w/v) sterilized sucrose solution and gamma-irradiated bee pollen, which was obtained following the protocol of Emery et al.^63^. Cages were kept in an incubator at 30 ± 2 °C under 60% relative humidity until the end of the experiment.

For each cage, the daily survival rate and food consumption were recorded, starting on the first day of chronic exposure to pesticides, and dead bees were daily removed for sanitary considerations. Nine days after emergence, bees were anesthetized with CO_2_, decapitated, and the guts were extracted for analysis. For the gut microbiota analyses, we dissected four guts from honey bees collected from four cages per treatment (n = 16 per treatment). Each gut was placed into a sterile 2 mL Eppendorf tube, flash frozen in liquid nitrogen then stored at − 80 °C. Thus, 16 individual honey bee worker guts were analyzed per treatment modality (resulting in a total of 160 experimental samples). For the analysis of physiological life history traits, the honey bees from the four cages were mixed together and the heads, midguts and abdomens (with the intestinal tract removed) were separately sampled. Tissues from three bees were pooled together to form each sample, weighed and stored at − 80 °C until analysis. For each treatment, seven repetitions (n = 7 samples of three pooled tissues per sample) were analyzed and each sample was assayed in triplicate for enzymatic activity.

### DNA extraction from honey bee gut tissue

Total DNA was extracted from each dissected gut using the FastPure Bacteria DNA Isolation Mini Kit (Vazyme Biotech Co., Ltd (China)). Each gut was put in 1 mL PBS and bead beated twice, for 45 s at 6 m/s, with a FastPrep-24™ 5G homogenizer (MP Biomedicals). A fraction of 330 µL of each homogenate was then transferred to new sterile 1.5 mL tubes and centrifuged for 1 min at 10,000 rpm. The pellet was supplemented with 180 µL of lysozyme (prepared according to the manufacturer’s instructions) and placed in a water bath for 2 h at 37 °C. We then followed the rest of the manufacturer’s protocol. The resulting dry pellet was resuspended in 50 µL of sterile water. For each batch of DNA extractions, two blank extractions (extractions in which no experimental tissue was added to the reagents; 16 total blank extractions) were also performed and served to identify and exclude bacterial contaminants present in laboratory reagents during 16S rRNA gene amplicon sequencing (see below).

### Quantitative PCR for the determination of absolute abundance

Total bacterial loads in experimental honey bee guts was quantified by quantitative PCR (qPCR) assays with universal 16S rRNA gene primers (F: AGGATTAGATACCCTGGTAGTCC; R: YCGTACTCCCCAGGCGG^40^). The 16S rRNA gene copy numbers were normalized against the host actin gene (amplified with primers F: TGCCAACACTGTCCTTTCTG and R: AGAATTGACCCACCAATCCA^104^) as previously described^35, 40^. Briefly, qPCR was performed on a StepOnePlus instrument (Applied Biosystems) in 96-well plates. Each sample was amplified in triplicate, in a final volume of 10 µL containing 0.4 µL of each forward and reverse primer (5 µM), 5 µL of 2 × SYBR^®^ Select Master Mix, 3.2 μL of MilliQ water and 1 µL of extracted DNA. The PCR program consisted of an initial denaturation step at 50 °C for 2 min, followed by 95 °C for 2 min, and 40 cycles of 95 °C for 15 s and 60 °C for 1 min. Melting curves were generated after each run at 95 °C for 15 s and 60 °C for 1 min and increments of 0.3 °C until reaching 95 °C for 15 s. Each plate contained a positive and negative control. The absolute quantity of each target was determined based on the standard curves of serial dilutions of plasmids (pGEM®-T Easy vector; Promega) containing the target sequence^35^. The number of bacterial cells per gut were determined by first calculating ‘raw’ copy numbers of each target in 1 μL of DNA from the *cycle quantification* (Cq) value and the standard curve using the formula n = *E* ^(intercept—Cq)^^105^. These values were multiplied by the elution volume of the DNA extractions to obtain calculations per gut. We next normalized the bacterial 16S rRNA gene copies against the median number of actin gene copies, dividing by the ‘raw’ actin copy number for the given sample and multiplying by the median number of actin gene copies across all samples. Finally, these values were divided by four to calculate bacterial cell numbers, as this roughly represents the mean number of 16S rRNA gene loci present across honey bee gut symbionts^40^. Normalization with the actin gene was performed to reduce the effect of gut size variation and extraction efficiency.

### 16S rRNA gene amplicon sequencing

The V4 region of the 16S rRNA gene was amplified using primers 515F-Nex (TCGTCGGCAGCGTCAGATGTGTATAAGAGACAGGTGCCAGCMGCCGCGGTAA) and 806R-Nex (GTCTCGTGGGCTCGGAGATGTGTATAAGAGACAGGGACTACHVGGGTWTCTAA), containing the Illumina adapter sequences for Nextera XT indexes and the primers of the V4 region of the 16S rRNA gene^106^, as described in the Illumina 16S metagenomic sequencing library preparation guide (https://support.illumina.com/content/dam/illumina-support/documents/documentation/chemistry_documentation/16s/16s-metagenomic-library-prep-guide-15044223-b.pdf) and by Kešnerová et al.^35^. The first PCR step was performed in a total volume of 25 µL, using 12.5 µL of 2 × Phanta Max Master Mix (Vazyme, Nanjing, China), 5 μL of MilliQ water, 2.5 μL of each primer (5 μM), and 2.5 μL of template DNA. The PCR program consisted of an initial denaturation step at 98 °C for 30 s, followed by 25 cycles of 98 °C for 10 s, 55 °C for 20 s and 72 °C for 20 s, and a final extension step at 72 °C for 5 min. Amplifications were verified by 2% agarose gel electrophoresis. PCR products were purified with Clean NGS purification beads in a 1:0.8 ratio of PCR products to beads, and eluted in 27 µL of 10 mM Tris–HCl pH 8.5. A second PCR step was then performed to append unique dual indexes to each sample. The PCR was performed in a total volume of 25 µL, using 12.5 µL of 2 × Phanta Max Master Mix (Vazyme, Nanjing, China), 5 μL of MilliQ water, 2.5 μL of Nextera XT index primers 1 and 2 (Nextera XT Index kit, Illumina) and 2.5 μL of template DNA. The PCR program consisted of an initial denaturation step at 95 °C for 3 min followed by eight cycles of 95 °C for 30 s, 55 °C for 30 s and 72 °C for 30 s, and a final extension step at 72 °C for 5 min. The final libraries were purified using Clean NGS purification beads in a 1:1.1 ratio of PCR product to beads, and eluted in 27.5 μL of 10 mM Tris–HCl pH 8.5. The amplicon concentrations, including the negative PCR controls, were then quantified by PicoGreen and pooled in equimolar concentrations (with the exception of the negative controls and the blank extractions, which were pooled in equal volumes instead). We verified that the final pool was of the right size using a Fragment Analyzer (Advanced Analytical) and performed sequencing on an Illumina MiSeq sequencer, producing 2 × 250 bp reads, at the Genomic Technology Facility of the University of Lausanne.

### Processing of 16S rRNA gene amplicon-sequencing data

We obtained a total of 13,976,585 raw sequences across 160 honey bee gut samples, two negative PCR controls, two mock community samples and 16 blank DNA extractions. Raw sequencing data (deposited at the SRA Database under BioProject accession no. PRJNA699592) were quality filtered with Trimmomatic^107^ using LEADING:3, TRAILING:3, SLIDINGWINDOW:4:15, and MINLEN:180. The quality-filtered data were analyzed with the Divisive Amplicon Denoising Algorithm 2 (DADA2) pipeline (“dada2” package version 1.14.1 in R)^108^. All functions were run using the recommended parameters (https://benjjneb.github.io/dada2/tutorial.html) except that at the filtering step we truncated the F and R reads after 232 and 231 bp, respectively. We then set randomize = TRUE and nbases = 3e8 at the learnErrors step. The SILVA database (version 132) was used for taxonomy assignments of the identified amplicon-sequence variants (ASVs). Any ASV classified as mitochondria, chloroplast or Eukaryota (“phyloseq” package version 1.30.0^109^, “subset_taxa” function) were removed. We then used both the “prevalence” and “frequency” methods (method = “either”) in the R package “decontam” v.1.6.0^110^ to identify and remove contaminants introduced during wet lab procedures, using the negative PCR controls and the blank samples as reference, which allowed us to identify and filter out 109 such ASVs. After removing negative, blank and mock samples, the final dataset consisted of 6,524,951 reads belonging to 288 ASVs across the 160 experimental samples.

### Statistical analyses of combined 16S rRNA gene amplicon-sequence and qPCR data

To calculate absolute bacterial abundances of each ASV, the proportion of each ASV in each sample inferred from MiSeq data were multiplied by the normalized 16S rRNA gene copy number of each sample as measured by qPCR. Bray–Curtis dissimilarities and weighted and unweigthed UniFrac distances were calculated after aligning all ASV sequences with DECIPHER v.2.14.0 and building a phylogenetic tree with Phangorn v.2.5.5^111^. ADONIS and ANOSIM tests were then ran to assess differences in community structure between microbiota and pesticide treatments overall, and for pesticide treatment differences within each microbiota treatment individually.

To test for differences between pesticide treatments for each individual ASV within each microbiota treatment, we used a permutation approach (referred to as permutation ANOVA) as done in^35^. Briefly, we randomized the values of the calculated copy numbers for each ASV 10,000 times and computed the *t* values for the tested effect for each randomized dataset. The *p* values corresponding to the effects were calculated as the proportion of 10,000 *t* values that were equal or higher than the observed one. Pairwise comparisons between individual treatment groups were performed by Tukey’s HSD using “multcomp” package^112^ using *glht* function on the model (Tables S1, S2). *P* values were adjusted using the Bonferroni method.

### Survival and food consumption

Mortality and food consumption were followed daily during the whole experiment. Dead bees were counted at 8 am and removed for hygienic considerations. Individual food consumption was assessed by measuring the weight of the feeder daily. The food consumed was corrected by the bees remaining alive. An evaporation control was included to accurately calculate the food consumed by the bees.

### Analysis of physiological life history traits

For the analyses of physiological markers, heads, abdomens and midguts were mixed with an extraction medium to make 10% (w/v) extract. The extraction medium consisted of 10 mM sodium chloride (NaCl), 1% (w/v) Triton X-100 and 40 mM sodium phosphate pH 7.4, and contained protease inhibitors (2 µg/mL antipain, leupeptin and pepstatin A, 25 units/mL aprotinin and 0.1 mg/mL soybean trypsin inhibitor)^113^. The extract was ground using the Qiagen^®^ TissueLyser II (30 Hz for 5 periods of 30 s, at 30 s intervals), then centrifuged at 4 °C for 20 min at 15,000 g_av_. The supernatant was then collected for analysis.

The physiological markers of each repetition were spectrophotometrically assayed in triplicate at 25 °C. LDH activity was determined by measuring the regeneration of nicotinamide adenine dinucleotide (NAD^+^) at 340 nm. The reaction medium contained 0.2 mM of the reduced form of nicotinamide adenine dinucleotide (NADH), 5 mM disodium ethylenediaminetetraacetate dihydrate (EDTA), 2 mM sodium pyruvate and 50 mM triethanolamine pH 7.6^114, 115^. GST activity was determined by measuring the conjugation of GSH to 1-chloro-2,4-dinitrobenzene (CDNB) at 340 nm. The reaction medium contained 1 mM EDTA, 1 mM CDNB, 2.5 mM GSH and 100 mM Na/K phosphate pH 7.4^116^. G6PDH activity was determined by following the formation of the reduced form of nicotinamide adenine dinucleotide phosphate (NADPH) at 340 nm. The reaction medium contained 10 mM magnesium chloride (MgCl_2_), 0.5 mM nicotinamide adenine dinucleotide phosphate (NADP^+^), 1 mM glucose-*6*-phosphate (G6P) and 100 mM Tris–HCl pH 7.4^86^. ALP was determined by following the formation of p-nitrophenol at 410 nm. The reaction medium contained 2 mM p-nitrophenyl phosphate (*p*-NPP), 20 µM MgCl_2_ and 100 mM Tris–HCl pH 8.5^117^. POx was measured by following the transformation of 3,4-dihydroxy-L-dihydroxyphenylalanine (L-DOPA) into melanin at 490 nm. The reaction medium contained 20 mM NaCl, 0.4 mg/mL L-DOPA, and 10 mM monosodium phosphate pH 7.2^118^.

### Statistical analyses

Statistical analyses of survival, food consumption and effects of the treatments on enzymatic activities were performed using R software (Rstudio Version 1.1.463). We determined the effects of pesticide treatments and gut colonization on survival using the packages *survival* and *survminer*^119, 120^, and the Kaplan–Meier (log-rank test) method followed by post hoc test. The Kruskal–Wallis test was used to determine the effect of the different treatments on food consumption by comparing the individual cumulative sucrose consumption during the exposure period. To test the effects of the different treatments on the physiological markers, we performed an ANOVA followed by Tukey’s HSD test, when the data followed a normal distribution, or a Kruskal–Wallis test followed by post hoc Dunn’s test (with Benjamini–Hochberg correction using the *agricolae* package^121^), when the data followed a non-normal distribution. In addition, a cluster analysis (hierarchical clustering results) was performed using PermutMatrix software^122^. Each measure was normalized according to the colonized control treatment, and unweighted pair group method with arithmetic mean (UPGMA) was conducted to determine Euclidian distances to be used as the linkage rule for clusters.

## Supplementary Information

Supplementary Figure 1.

Supplementary Figure 2.

Supplementary Figure 3.

Supplementary Figure 4.

Supplementary Figure 5.

Supplementary Table 1.

Supplementary Table 2.

Supplementary Tables.

## Data Availability

The datasets used and/or analyzed during the current study are available from the corresponding authors on reasonable request.
