# Supplementary material for: Mild chronic exposure to pesticides alters physiological markers of honey bee health without perturbing the core gut microbiota
Source: Sci Rep. 2022 Mar 11;12:4281. doi: 10.1038/s41598-022-08009-2 (PMC8917129; doi:10.1038/s41598-022-08009-2)
Supplement: Supplementary file 1 — Supplementary Figure 1. [file 41598_2022_8009_MOESM1_ESM.docx]

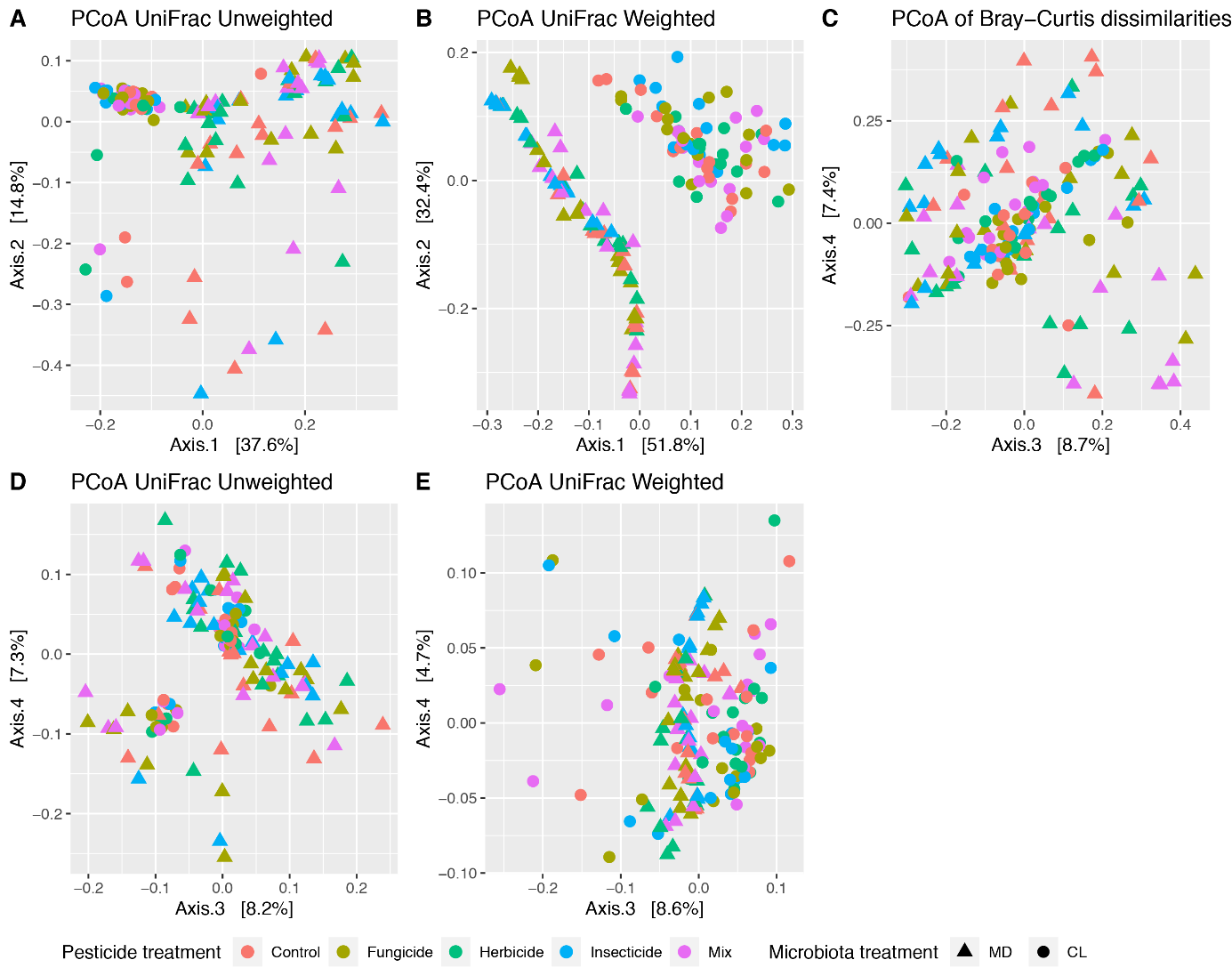


**Fig. S1** Principal coordinate analyses based on unweighted (A and D) and weighted (B and E) UniFrac metrics and Bray-Curtis dissimilarities (C). Panels A and B, and C, D and E, show axes 1 and 2, and 3 and 4, respectively, as well as the variance they explain. See Fig. 1E for axes 1 and 2 of Bray-Curtis dissimilarities.
