# Supplementary material for: Mild chronic exposure to pesticides alters physiological markers of honey bee health without perturbing the core gut microbiota
Source: Sci Rep. 2022 Mar 11;12:4281. doi: 10.1038/s41598-022-08009-2 (PMC8917129; doi:10.1038/s41598-022-08009-2)
Supplement: Supplementary file 2 — Supplementary Figure 2. [file 41598_2022_8009_MOESM2_ESM.docx]

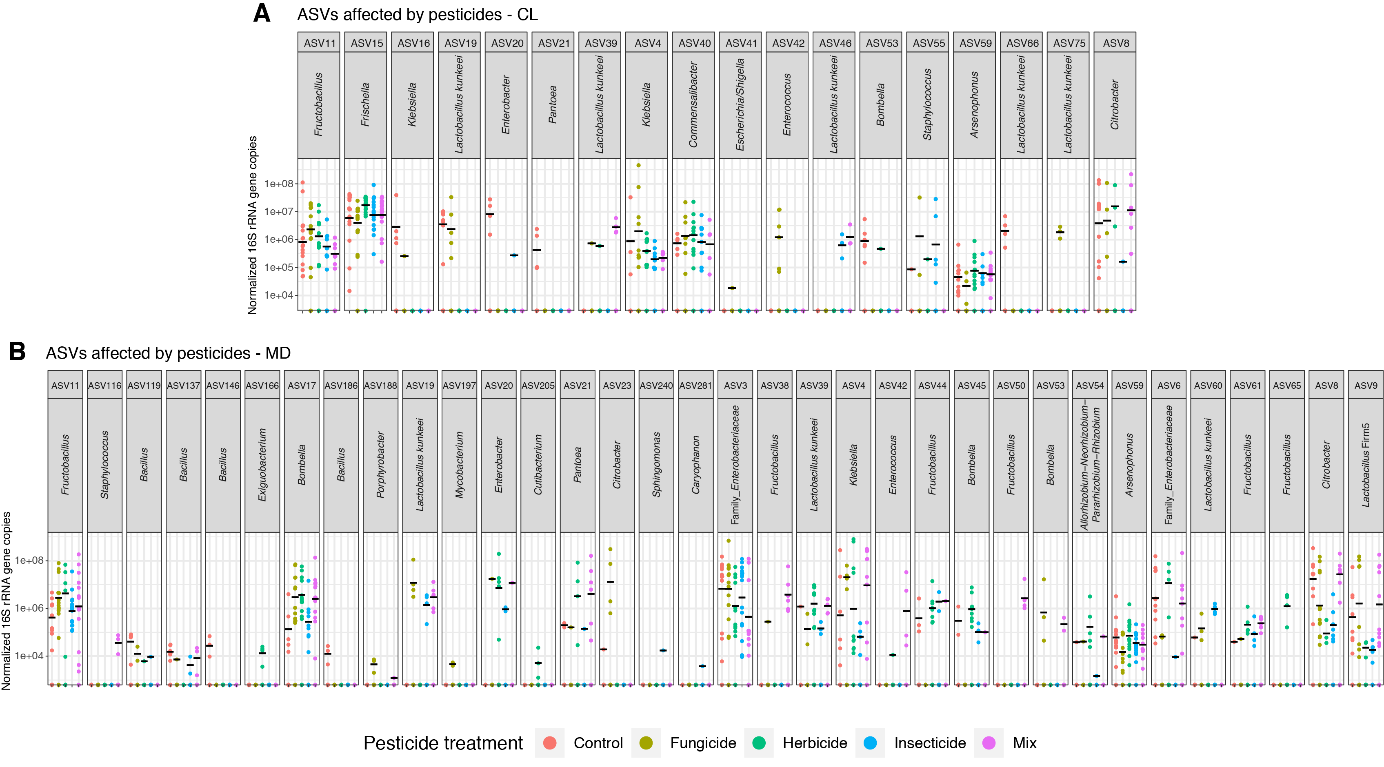


**Fig. S2** Absolute abundances (16S rRNA gene copies normalized by the amount of host actin) of amplicon-sequence variants (ASVs) that showed significant changes following pesticide exposure based on permutation ANOVAs in either the CL (A) or the MD group (B).
