# Supplementary material for: Mild chronic exposure to pesticides alters physiological markers of honey bee health without perturbing the core gut microbiota
Source: Sci Rep. 2022 Mar 11;12:4281. doi: 10.1038/s41598-022-08009-2 (PMC8917129; doi:10.1038/s41598-022-08009-2)
Supplement: Supplementary file 3 — Supplementary Figure 3. [file 41598_2022_8009_MOESM3_ESM.docx]

**Fig. S3.** Effects of the pesticides and gut colonization on the physiological state of newly emerged honey bees.

The Cluster analysis represents an interactive approach to assess the effect of pesticide treatments and gut colonization status on the physiological markers analyzed in the head (h), abdomen (a) and midgut (m) of the honey bee. Colonized (CL) and microbiota-depleted (MD) honey bees were fed sucrose solutions containing no pesticides (Control), imidacloprid (Insecticide), glyphosate (Herbicide), difenoconazole (Fungicide) or the ternary mixture (Mix) at concentrations of 0.1 µg/L in food. Euclidian distances were determined with UPGMA to serve as the linkage rule for clusters. Data normalization was required to convert each treatment to the rate of variation compared with the average of controls (CL.Control). The intensity of modulation is illustrated by the range of colors, with green and red indicating respectively a decrease and an increase of the mean enzymatic activity of each treatment, in comparison with the mean value in the CL.Control. Black indicates no change in comparison with the mean value of CL.Control.
