# Supplementary material for: Mild chronic exposure to pesticides alters physiological markers of honey bee health without perturbing the core gut microbiota
Source: Sci Rep. 2022 Mar 11;12:4281. doi: 10.1038/s41598-022-08009-2 (PMC8917129; doi:10.1038/s41598-022-08009-2)
Supplement: Supplementary file 4 — Supplementary Figure 4. [file 41598_2022_8009_MOESM4_ESM.docx]

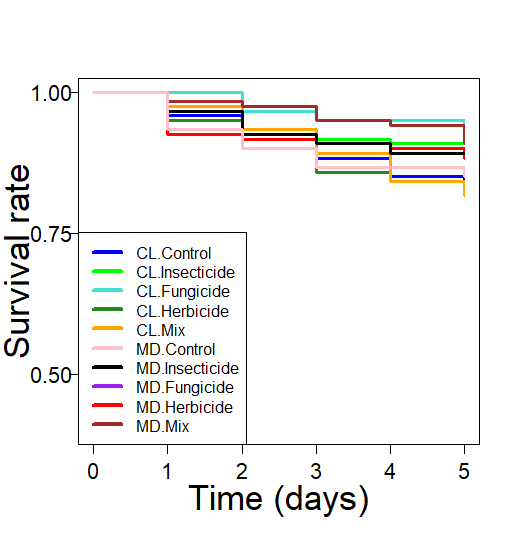


**Fig. S4.** Effects of pesticides on the longevity of colonized and microbiota-depleted honey bees

Microbiota-depleted (MD) and gut colonized (CL) honey bees were fed for five days sterile sucrose solutions containing no pesticides (Control) or imidacloprid (Insecticide), glyphosate (Herbicide), difenoconazole (Fungicide) alone or as a ternary mixture (Mix) at 0.1 µg/L in food. The data represent the proportion of surviving honeybees exposed to the different treatments. No significant differences in the survival rates were observed between the different treatments (Kaplan-Meier method (log-rank test), followed by a post hoc test).
