# Supplementary material for: Mild chronic exposure to pesticides alters physiological markers of honey bee health without perturbing the core gut microbiota
Source: Sci Rep. 2022 Mar 11;12:4281. doi: 10.1038/s41598-022-08009-2 (PMC8917129; doi:10.1038/s41598-022-08009-2)
Supplement: Supplementary file 5 — Supplementary Figure 5. [file 41598_2022_8009_MOESM5_ESM.docx]

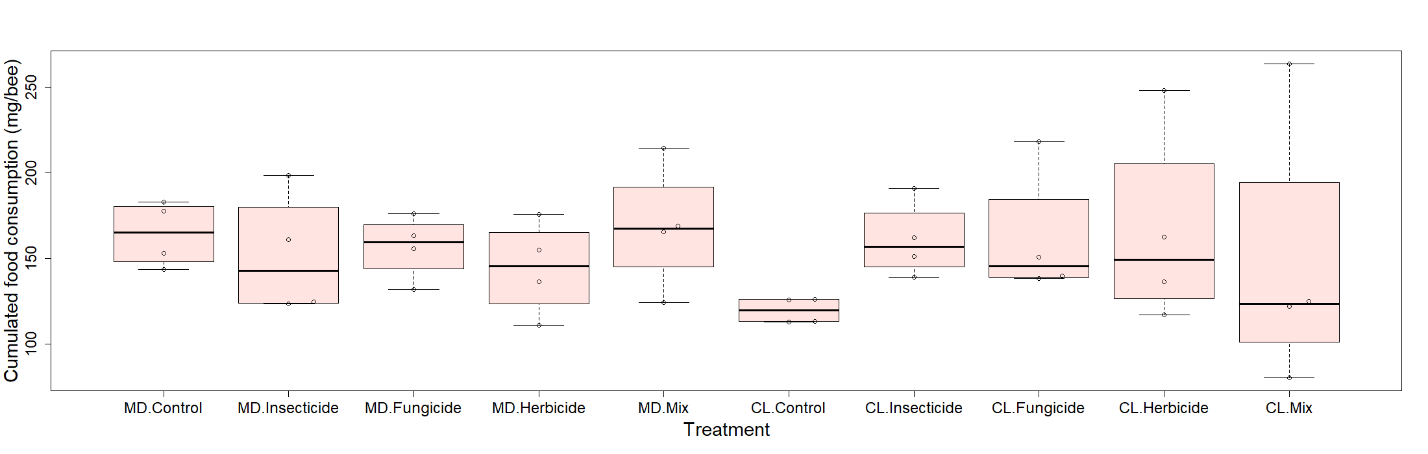


**Fig. S5.** Effects of pesticides the food consumption of colonized and microbiota-depleted honey bees

Microbiota-depleted (MD) and gut colonized (CL) honey bees were fed for five days sterile sucrose solutions containing no pesticides (Control) or imidacloprid (Insecticide), glyphosate (Herbicide), difenoconazole (Fungicide) alone or as a ternary mixture (Mix) at 0.1 µg/L in food. Box plots represent the cumulated individual consumption (mg/bee) for 4 cages of 30 bees per treatment. Statistical analyses were performed using the Kruskal-Wallis test followed by pairwise comparisons using the Wilcoxon rank sum test with the Benjamini-Hochberg correction. No differences in food consumption had been observed between the different treatments.
