# Supplementary material for: Mild chronic exposure to pesticides alters physiological markers of honey bee health without perturbing the core gut microbiota
Source: Sci Rep. 2022 Mar 11;12:4281. doi: 10.1038/s41598-022-08009-2 (PMC8917129; doi:10.1038/s41598-022-08009-2)
Supplement: Supplementary file 6 — Supplementary Table 1. [file 41598_2022_8009_MOESM6_ESM.pdf]

Table S1

ASV1

```
       Simultaneous Tests for General Linear Hypotheses

Multiple Comparisons of Means: Tukey Contrasts

Fit: lm(formula = log_cop ~ Treatment, data = dsub)

Linear Hypotheses:

               Estimate Std. Error t value Pr(>|t|)
CL.F - CL.C == 0   -0.22639    0.19652  -1.152    1.000
CL.H - CL.C == 0    0.12826    0.19652   0.653    1.000
CL.I - CL.C == 0    0.01951    0.19652   0.099    1.000
CL.Mix - CL.C == 0  0.27771    0.19652   1.413    1.000
CL.H - CL.F == 0    0.35465    0.19652   1.805    0.751
CL.I - CL.F == 0    0.24590    0.19652   1.251    1.000
CL.Mix - CL.F == 0  0.50410    0.19652   2.565    0.123
CL.I - CL.H == 0   -0.10875    0.19652  -0.553    1.000
CL.Mix - CL.H == 0  0.14946    0.19652   0.761    1.000
CL.Mix - CL.I == 0  0.25821    0.19652   1.314    1.000
(Adjusted p values reported -- bonferroni method)
```

ASV10

```
       Simultaneous Tests for General Linear Hypotheses

Multiple Comparisons of Means: Tukey Contrasts

Fit: lm(formula = log_cop ~ Treatment, data = dsub)

Linear Hypotheses:

                Estimate Std. Error t value Pr(>|t|)
CL.F - CL.C == 0    0.602139   0.320339   1.880    0.640
```

```
CL.H - CL.C == 0    0.643609   0.320339   2.009    0.481
CL.I - CL.C == 0    0.638960   0.320339   1.995    0.497
CL.Mix - CL.C == 0  0.440554   0.320339   1.375    1.000
CL.H - CL.F == 0    0.041470   0.320339   0.129    1.000
CL.I - CL.F == 0    0.036821   0.320339   0.115    1.000
CL.Mix - CL.F == 0 -0.161585   0.320339  -0.504    1.000
CL.I - CL.H == 0   -0.004649   0.320339  -0.015    1.000
CL.Mix - CL.H == 0 -0.203055   0.320339  -0.634    1.000
CL.Mix - CL.I == 0 -0.198406   0.320339  -0.619    1.000
(Adjusted p values reported -- bonferroni method)
```

ASV109

```
       Simultaneous Tests for General Linear Hypotheses
Multiple Comparisons of Means: Tukey Contrasts
Fit: lm(formula = log_cop ~ Treatment, data = dsub)

Linear Hypotheses:
                 Estimate Std. Error t value Pr(>|t|)
CL.F - CL.C == 0    3.975e-01  3.467e-01   1.147        1
CL.H - CL.C == 0    3.584e-16  3.467e-01   0.000        1
CL.I - CL.C == 0    3.059e-16  3.467e-01   0.000        1
CL.Mix - CL.C == 0  3.774e-01  3.467e-01   1.089        1
CL.H - CL.F == 0   -3.975e-01  3.467e-01  -1.147        1
CL.I - CL.F == 0   -3.975e-01  3.467e-01  -1.147        1
CL.Mix - CL.F == 0 -2.014e-02  3.467e-01  -0.058        1
CL.I - CL.H == 0   -5.243e-17  3.467e-01   0.000        1
CL.Mix - CL.H == 0  3.774e-01  3.467e-01   1.089        1
CL.Mix - CL.I == 0  3.774e-01  3.467e-01   1.089        1
```

```
(Adjusted p values reported -- bonferroni method)


ASV11
	 Simultaneous Tests for General Linear Hypotheses
Multiple Comparisons of Means: Tukey Contrasts
Fit: lm(formula = log_cop ~ Treatment, data = dsub)

Linear Hypotheses:
               Estimate Std. Error t value Pr(>|t|)
CL.F - CL.C == 0    -0.7459     0.9004  -0.828  1.00000
CL.H - CL.C == 0    -1.3313     0.9004  -1.478  1.00000
CL.I - CL.C == 0    -2.3251     0.9004  -2.582  0.11764
CL.Mix - CL.C == 0  -3.1755     0.9004  -3.527  0.00722 **
CL.H - CL.F == 0    -0.5853     0.9004  -0.650  1.00000
CL.I - CL.F == 0    -1.5792     0.9004  -1.754  0.83543
CL.Mix - CL.F == 0  -2.4296     0.9004  -2.698  0.08605 .
CL.I - CL.H == 0    -0.9939     0.9004  -1.104  1.00000
CL.Mix - CL.H == 0  -1.8443     0.9004  -2.048  0.44033
CL.Mix - CL.I == 0  -0.8504     0.9004  -0.944  1.00000
---
Signif. codes:  0 ‘***’ 0.001 ‘**’ 0.01 ‘*’ 0.05 ‘.’ 0.1 ‘ ’ 1
(Adjusted p values reported -- bonferroni method)


ASV110
	 Simultaneous Tests for General Linear Hypotheses
Multiple Comparisons of Means: Tukey Contrasts
Fit: lm(formula = log_cop ~ Treatment, data = dsub)
```

```
Linear Hypotheses:
                 Estimate Std. Error t value Pr(>|t|)
CL.F - CL.C == 0   -4.072e-01  2.575e-01  -1.581        1
CL.H - CL.C == 0   -4.072e-01  2.575e-01  -1.581        1
CL.I - CL.C == 0   -4.072e-01  2.575e-01  -1.581        1
CL.Mix - CL.C == 0 -4.072e-01  2.575e-01  -1.581        1
CL.H - CL.F == 0   -3.331e-16  2.575e-01   0.000        1
CL.I - CL.F == 0   -1.110e-16  2.575e-01   0.000        1
CL.Mix - CL.F == 0 -3.331e-16  2.575e-01   0.000        1
CL.I - CL.H == 0    2.220e-16  2.575e-01   0.000        1
CL.Mix - CL.H == 0  0.000e+00  2.575e-01   0.000        1
CL.Mix - CL.I == 0 -2.220e-16  2.575e-01   0.000        1
(Adjusted p values reported -- bonferroni method)
```

```
ASV111
       Simultaneous Tests for General Linear Hypotheses
Multiple Comparisons of Means: Tukey Contrasts
Fit: lm(formula = log_cop ~ Treatment, data = dsub)

Linear Hypotheses:
                 Estimate Std. Error t value Pr(>|t|)
CL.F - CL.C == 0   -0.3532416  0.4999772  -0.707        1
CL.H - CL.C == 0    0.0124312  0.4999772   0.025        1
CL.I - CL.C == 0    0.3664259  0.4999772   0.733        1
CL.Mix - CL.C == 0 -0.0009981  0.4999772  -0.002        1
CL.H - CL.F == 0    0.3656728  0.4999772   0.731        1
CL.I - CL.F == 0    0.7196675  0.4999772   1.439        1
CL.Mix - CL.F == 0  0.3522434  0.4999772   0.705        1
```

```
CL.I - CL.H == 0    0.3539947  0.4999772   0.708        1
CL.Mix - CL.H == 0 -0.0134293  0.4999772  -0.027        1
CL.Mix - CL.I == 0 -0.3674240  0.4999772  -0.735        1
(Adjusted p values reported -- bonferroni method)
```

```
ASV112

       Simultaneous Tests for General Linear Hypotheses

Multiple Comparisons of Means: Tukey Contrasts

Fit: lm(formula = log_cop ~ Treatment, data = dsub)

Linear Hypotheses:
                    Estimate Std. Error t value Pr(>|t|)
CL.F - CL.C == 0    3.170e-01  2.005e-01   1.581        1
CL.H - CL.C == 0    4.718e-18  2.005e-01   0.000        1
CL.I - CL.C == 0    6.725e-18  2.005e-01   0.000        1
CL.Mix - CL.C == 0  6.783e-17  2.005e-01   0.000        1
CL.H - CL.F == 0   -3.170e-01  2.005e-01  -1.581        1
CL.I - CL.F == 0   -3.170e-01  2.005e-01  -1.581        1
CL.Mix - CL.F == 0 -3.170e-01  2.005e-01  -1.581        1
CL.I - CL.H == 0    2.007e-18  2.005e-01   0.000        1
CL.Mix - CL.H == 0  6.311e-17  2.005e-01   0.000        1
CL.Mix - CL.I == 0  6.110e-17  2.005e-01   0.000        1
(Adjusted p values reported -- bonferroni method)
```

```
ASV113

       Simultaneous Tests for General Linear Hypotheses

Multiple Comparisons of Means: Tukey Contrasts
```

```
Fit: lm(formula = log_cop ~ Treatment, data = dsub)

Linear Hypotheses:
               Estimate Std. Error t value Pr(>|t|)
CL.F - CL.C == 0     0.7209     0.6775   1.064    1.000
CL.H - CL.C == 0     1.0967     0.6775   1.619    1.000
CL.I - CL.C == 0     1.4447     0.6775   2.132    0.363
CL.Mix - CL.C == 0   0.3703     0.6775   0.546    1.000
CL.H - CL.F == 0     0.3758     0.6775   0.555    1.000
CL.I - CL.F == 0     0.7238     0.6775   1.068    1.000
CL.Mix - CL.F == 0  -0.3506     0.6775  -0.518    1.000
CL.I - CL.H == 0     0.3480     0.6775   0.514    1.000
CL.Mix - CL.H == 0  -0.7264     0.6775  -1.072    1.000
CL.Mix - CL.I == 0  -1.0744     0.6775  -1.586    1.000
(Adjusted p values reported -- bonferroni method)



ASV116
       Simultaneous Tests for General Linear Hypotheses
Multiple Comparisons of Means: Tukey Contrasts
Fit: lm(formula = log_cop ~ Treatment, data = dsub)

Linear Hypotheses:
                 Estimate Std. Error t value Pr(>|t|)
CL.F - CL.C == 0   -7.809e-17  1.779e-01   0.000        1
CL.H - CL.C == 0   -6.410e-17  1.779e-01   0.000        1
CL.I - CL.C == 0    2.813e-01  1.779e-01   1.581        1
CL.Mix - CL.C == 0 -8.832e-17  1.779e-01   0.000        1
CL.H - CL.F == 0    1.399e-17  1.779e-01   0.000        1
CL.I - CL.F == 0    2.813e-01  1.779e-01   1.581        1
```

```
CL.Mix - CL.F == 0 -1.023e-17  1.779e-01   0.000        1
CL.I - CL.H == 0    2.813e-01  1.779e-01   1.581        1
CL.Mix - CL.H == 0 -2.422e-17  1.779e-01   0.000        1
CL.Mix - CL.I == 0 -2.813e-01  1.779e-01  -1.581        1
(Adjusted p values reported -- bonferroni method)
```

ASV119

```
	 Simultaneous Tests for General Linear Hypotheses

Multiple Comparisons of Means: Tukey Contrasts

Fit: lm(formula = log_cop ~ Treatment, data = dsub)

Linear Hypotheses:
                 Estimate Std. Error t value Pr(>|t|)
CL.F - CL.C == 0   -2.817e-01  1.782e-01  -1.581        1
CL.H - CL.C == 0   -2.817e-01  1.782e-01  -1.581        1
CL.I - CL.C == 0   -2.817e-01  1.782e-01  -1.581        1
CL.Mix - CL.C == 0 -2.817e-01  1.782e-01  -1.581        1
CL.H - CL.F == 0   -5.551e-17  1.782e-01   0.000        1
CL.I - CL.F == 0   -2.220e-16  1.782e-01   0.000        1
CL.Mix - CL.F == 0  5.551e-17  1.782e-01   0.000        1
CL.I - CL.H == 0   -1.665e-16  1.782e-01   0.000        1
CL.Mix - CL.H == 0  1.110e-16  1.782e-01   0.000        1
CL.Mix - CL.I == 0  2.776e-16  1.782e-01   0.000        1
(Adjusted p values reported -- bonferroni method)
```

ASV12

```
	 Simultaneous Tests for General Linear Hypotheses
```

```
Multiple Comparisons of Means: Tukey Contrasts

Fit: lm(formula = log_cop ~ Treatment, data = dsub)

Linear Hypotheses:
                   Estimate Std. Error t value Pr(>|t|)
CL.F - CL.C == 0    0.57947    0.33812   1.714    0.907
CL.H - CL.C == 0    0.61361    0.33812   1.815    0.736
CL.I - CL.C == 0    0.71269    0.33812   2.108    0.384
CL.Mix - CL.C == 0  0.67963    0.33812   2.010    0.480
CL.H - CL.F == 0    0.03414    0.33812   0.101    1.000
CL.I - CL.F == 0    0.13323    0.33812   0.394    1.000
CL.Mix - CL.F == 0  0.10017    0.33812   0.296    1.000
CL.I - CL.H == 0    0.09908    0.33812   0.293    1.000
CL.Mix - CL.H == 0  0.06602    0.33812   0.195    1.000
CL.Mix - CL.I == 0 -0.03306    0.33812  -0.098    1.000
(Adjusted p values reported -- bonferroni method)
```

ASV122

```
         Simultaneous Tests for General Linear Hypotheses

Multiple Comparisons of Means: Tukey Contrasts

Fit: lm(formula = log_cop ~ Treatment, data = dsub)

Linear Hypotheses:
                   Estimate Std. Error t value Pr(>|t|)
CL.F - CL.C == 0    0.35062    0.42722   0.821        1
CL.H - CL.C == 0   -0.32971    0.42722  -0.772        1
CL.I - CL.C == 0    0.03268    0.42722   0.076        1
CL.Mix - CL.C == 0 -0.32971    0.42722  -0.772        1
CL.H - CL.F == 0   -0.68033    0.42722  -1.592        1
```

```
CL.I - CL.F == 0   -0.31795    0.42722  -0.744        1
CL.Mix - CL.F == 0 -0.68033    0.42722  -1.592        1
CL.I - CL.H == 0    0.36239    0.42722   0.848        1
CL.Mix - CL.H == 0  0.00000    0.42722   0.000        1
CL.Mix - CL.I == 0 -0.36239    0.42722  -0.848        1
(Adjusted p values reported -- bonferroni method)
```

ASV13

```
	 Simultaneous Tests for General Linear Hypotheses
Multiple Comparisons of Means: Tukey Contrasts
Fit: lm(formula = log_cop ~ Treatment, data = dsub)

Linear Hypotheses:
               Estimate Std. Error t value Pr(>|t|)
CL.F - CL.C == 0    0.41916    0.32211   1.301    1.000
CL.H - CL.C == 0    0.45628    0.32211   1.417    1.000
CL.I - CL.C == 0    0.55481    0.32211   1.722    0.891
CL.Mix - CL.C == 0  0.62453    0.32211   1.939    0.563
CL.H - CL.F == 0    0.03712    0.32211   0.115    1.000
CL.I - CL.F == 0    0.13564    0.32211   0.421    1.000
CL.Mix - CL.F == 0  0.20537    0.32211   0.638    1.000
CL.I - CL.H == 0    0.09853    0.32211   0.306    1.000
CL.Mix - CL.H == 0  0.16825    0.32211   0.522    1.000
CL.Mix - CL.I == 0  0.06973    0.32211   0.216    1.000
(Adjusted p values reported -- bonferroni method)
```

ASV137

```
	 Simultaneous Tests for General Linear Hypotheses

Multiple Comparisons of Means: Tukey Contrasts

Fit: lm(formula = log_cop ~ Treatment, data = dsub)

Linear Hypotheses:
                 Estimate Std. Error t value Pr(>|t|)
CL.F - CL.C == 0   -6.512e-01  2.814e-01  -2.314    0.234
CL.H - CL.C == 0   -6.512e-01  2.814e-01  -2.314    0.234
CL.I - CL.C == 0   -6.512e-01  2.814e-01  -2.314    0.234
CL.Mix - CL.C == 0 -6.512e-01  2.814e-01  -2.314    0.234
CL.H - CL.F == 0   -4.441e-16  2.814e-01   0.000    1.000
CL.I - CL.F == 0   -4.441e-16  2.814e-01   0.000    1.000
CL.Mix - CL.F == 0 -3.331e-16  2.814e-01   0.000    1.000
CL.I - CL.H == 0    0.000e+00  2.814e-01   0.000    1.000
CL.Mix - CL.H == 0  1.110e-16  2.814e-01   0.000    1.000
CL.Mix - CL.I == 0  1.110e-16  2.814e-01   0.000    1.000
(Adjusted p values reported -- bonferroni method)


ASV14

	 Simultaneous Tests for General Linear Hypotheses

Multiple Comparisons of Means: Tukey Contrasts

Fit: lm(formula = log_cop ~ Treatment, data = dsub)

Linear Hypotheses:
               Estimate Std. Error t value Pr(>|t|)
CL.F - CL.C == 0    0.58406    0.32399   1.803    0.755
CL.H - CL.C == 0    0.45640    0.32399   1.409    1.000
CL.I - CL.C == 0    0.70278    0.32399   2.169    0.332
CL.Mix - CL.C == 0  0.63102    0.32399   1.948    0.552
```

```
CL.H - CL.F == 0   -0.12766    0.32399  -0.394    1.000
CL.I - CL.F == 0    0.11872    0.32399   0.366    1.000
CL.Mix - CL.F == 0  0.04696    0.32399   0.145    1.000
CL.I - CL.H == 0    0.24638    0.32399   0.760    1.000
CL.Mix - CL.H == 0  0.17463    0.32399   0.539    1.000
CL.Mix - CL.I == 0 -0.07176    0.32399  -0.221    1.000
(Adjusted p values reported -- bonferroni method)
```

ASV140

```
       Simultaneous Tests for General Linear Hypotheses

Multiple Comparisons of Means: Tukey Contrasts

Fit: lm(formula = log_cop ~ Treatment, data = dsub)

Linear Hypotheses:
                    Estimate Std. Error t value Pr(>|t|)
CL.F - CL.C == 0   -3.548e-01  2.244e-01  -1.581        1
CL.H - CL.C == 0   -3.548e-01  2.244e-01  -1.581        1
CL.I - CL.C == 0   -3.548e-01  2.244e-01  -1.581        1
CL.Mix - CL.C == 0 -3.548e-01  2.244e-01  -1.581        1
CL.H - CL.F == 0   -1.665e-16  2.244e-01   0.000        1
CL.I - CL.F == 0   -2.220e-16  2.244e-01   0.000        1
CL.Mix - CL.F == 0 -1.665e-16  2.244e-01   0.000        1
CL.I - CL.H == 0   -5.551e-17  2.244e-01   0.000        1
CL.Mix - CL.H == 0  0.000e+00  2.244e-01   0.000        1
CL.Mix - CL.I == 0  5.551e-17  2.244e-01   0.000        1
(Adjusted p values reported -- bonferroni method)
```

ASV15

```
	 Simultaneous Tests for General Linear Hypotheses

Multiple Comparisons of Means: Tukey Contrasts

Fit: lm(formula = log_cop ~ Treatment, data = dsub)

Linear Hypotheses:
                 Estimate Std. Error t value Pr(>|t|)
CL.F - CL.C == 0   -1.412898   0.558553  -2.530   0.1352
CL.H - CL.C == 0    0.009097   0.558553   0.016   1.0000
CL.I - CL.C == 0    0.101366   0.558553   0.181   1.0000
CL.Mix - CL.C == 0  0.112976   0.558553   0.202   1.0000
CL.H - CL.F == 0    1.421995   0.558553   2.546   0.1295
CL.I - CL.F == 0    1.514264   0.558553   2.711   0.0831 .
CL.Mix - CL.F == 0  1.525875   0.558553   2.732   0.0785 .
CL.I - CL.H == 0    0.092269   0.558553   0.165   1.0000
CL.Mix - CL.H == 0  0.103880   0.558553   0.186   1.0000
CL.Mix - CL.I == 0  0.011611   0.558553   0.021   1.0000
---
Signif. codes:  0 ‘***’ 0.001 ‘**’ 0.01 ‘*’ 0.05 ‘.’ 0.1 ‘ ’ 1
(Adjusted p values reported -- bonferroni method)
```

ASV155

```
	 Simultaneous Tests for General Linear Hypotheses

Multiple Comparisons of Means: Tukey Contrasts

Fit: lm(formula = log_cop ~ Treatment, data = dsub)

Linear Hypotheses:
                  Estimate Std. Error t value Pr(>|t|)
CL.F - CL.C == 0    9.812e-17  1.861e-01   0.000        1
```

```
CL.H - CL.C == 0    1.442e-16  1.861e-01   0.000        1
CL.I - CL.C == 0    2.943e-01  1.861e-01   1.581        1
CL.Mix - CL.C == 0  1.865e-16  1.861e-01   0.000        1
CL.H - CL.F == 0    4.610e-17  1.861e-01   0.000        1
CL.I - CL.F == 0    2.943e-01  1.861e-01   1.581        1
CL.Mix - CL.F == 0  8.833e-17  1.861e-01   0.000        1
CL.I - CL.H == 0    2.943e-01  1.861e-01   1.581        1
CL.Mix - CL.H == 0  4.223e-17  1.861e-01   0.000        1
CL.Mix - CL.I == 0 -2.943e-01  1.861e-01  -1.581        1
(Adjusted p values reported -- bonferroni method)
```

ASV157

```
         Simultaneous Tests for General Linear Hypotheses
Multiple Comparisons of Means: Tukey Contrasts
Fit: lm(formula = log_cop ~ Treatment, data = dsub)

Linear Hypotheses:
                  Estimate Std. Error t value Pr(>|t|)
CL.F - CL.C == 0   -3.475e-01  2.998e-01  -1.159        1
CL.H - CL.C == 0   -3.475e-01  2.998e-01  -1.159        1
CL.I - CL.C == 0   -2.514e-02  2.998e-01  -0.084        1
CL.Mix - CL.C == 0 -3.475e-01  2.998e-01  -1.159        1
CL.H - CL.F == 0   -2.220e-16  2.998e-01   0.000        1
CL.I - CL.F == 0    3.223e-01  2.998e-01   1.075        1
CL.Mix - CL.F == 0 -4.996e-16  2.998e-01   0.000        1
CL.I - CL.H == 0    3.223e-01  2.998e-01   1.075        1
CL.Mix - CL.H == 0 -2.776e-16  2.998e-01   0.000        1
CL.Mix - CL.I == 0 -3.223e-01  2.998e-01  -1.075        1
```

```
(Adjusted p values reported -- bonferroni method)



ASV16

	 Simultaneous Tests for General Linear Hypotheses

Multiple Comparisons of Means: Tukey Contrasts

Fit: lm(formula = log_cop ~ Treatment, data = dsub)

Linear Hypotheses:
                 Estimate Std. Error t value Pr(>|t|)
CL.F - CL.C == 0   -1.275e+00  5.069e-01  -2.516   0.1402
CL.H - CL.C == 0   -1.613e+00  5.069e-01  -3.183   0.0212 *
CL.I - CL.C == 0   -1.613e+00  5.069e-01  -3.183   0.0212 *
CL.Mix - CL.C == 0 -1.613e+00  5.069e-01  -3.183   0.0212 *
CL.H - CL.F == 0   -3.380e-01  5.069e-01  -0.667   1.0000
CL.I - CL.F == 0   -3.380e-01  5.069e-01  -0.667   1.0000
CL.Mix - CL.F == 0 -3.380e-01  5.069e-01  -0.667   1.0000
CL.I - CL.H == 0   -1.332e-15  5.069e-01   0.000   1.0000
CL.Mix - CL.H == 0 -1.332e-15  5.069e-01   0.000   1.0000
CL.Mix - CL.I == 0  0.000e+00  5.069e-01   0.000   1.0000
---
Signif. codes:  0 ‘***’ 0.001 ‘**’ 0.01 ‘*’ 0.05 ‘.’ 0.1 ‘ ’ 1
(Adjusted p values reported -- bonferroni method)



ASV169

	 Simultaneous Tests for General Linear Hypotheses

Multiple Comparisons of Means: Tukey Contrasts
```

```
Fit: lm(formula = log_cop ~ Treatment, data = dsub)

Linear Hypotheses:
                   Estimate Std. Error t value Pr(>|t|)
CL.F - CL.C == 0    3.488e-01  2.206e-01   1.581        1
CL.H - CL.C == 0    1.010e-17  2.206e-01   0.000        1
CL.I - CL.C == 0    1.395e-17  2.206e-01   0.000        1
CL.Mix - CL.C == 0  8.115e-17  2.206e-01   0.000        1
CL.H - CL.F == 0   -3.488e-01  2.206e-01  -1.581        1
CL.I - CL.F == 0   -3.488e-01  2.206e-01  -1.581        1
CL.Mix - CL.F == 0 -3.488e-01  2.206e-01  -1.581        1
CL.I - CL.H == 0    3.852e-18  2.206e-01   0.000        1
CL.Mix - CL.H == 0  7.105e-17  2.206e-01   0.000        1
CL.Mix - CL.I == 0  6.720e-17  2.206e-01   0.000        1
(Adjusted p values reported -- bonferroni method)
```

ASV17

```
       Simultaneous Tests for General Linear Hypotheses

Multiple Comparisons of Means: Tukey Contrasts

Fit: lm(formula = log_cop ~ Treatment, data = dsub)

Linear Hypotheses:
               Estimate Std. Error t value Pr(>|t|)
CL.F - CL.C == 0    -0.6122     0.9166  -0.668    1.000
CL.H - CL.C == 0    -0.3600     0.9166  -0.393    1.000
CL.I - CL.C == 0    -1.4368     0.9166  -1.567    1.000
CL.Mix - CL.C == 0  -2.0501     0.9166  -2.236    0.283
CL.H - CL.F == 0     0.2521     0.9166   0.275    1.000
CL.I - CL.F == 0    -0.8247     0.9166  -0.900    1.000
```

```
CL.Mix - CL.F == 0  -1.4379     0.9166  -1.569    1.000
CL.I - CL.H == 0    -1.0768     0.9166  -1.175    1.000
CL.Mix - CL.H == 0  -1.6900     0.9166  -1.844    0.692
CL.Mix - CL.I == 0  -0.6132     0.9166  -0.669    1.000
(Adjusted p values reported -- bonferroni method)
```

ASV18

```
	 Simultaneous Tests for General Linear Hypotheses

Multiple Comparisons of Means: Tukey Contrasts

Fit: lm(formula = log_cop ~ Treatment, data = dsub)

Linear Hypotheses:
                  Estimate Std. Error t value Pr(>|t|)
CL.F - CL.C == 0    0.9865368  0.7961567   1.239    1.000
CL.H - CL.C == 0    1.4148883  0.7961567   1.777    0.796
CL.I - CL.C == 0   -0.0009841  0.7961567  -0.001    1.000
CL.Mix - CL.C == 0  0.6480587  0.7961567   0.814    1.000
CL.H - CL.F == 0    0.4283515  0.7961567   0.538    1.000
CL.I - CL.F == 0   -0.9875209  0.7961567  -1.240    1.000
CL.Mix - CL.F == 0 -0.3384781  0.7961567  -0.425    1.000
CL.I - CL.H == 0   -1.4158725  0.7961567  -1.778    0.794
CL.Mix - CL.H == 0 -0.7668296  0.7961567  -0.963    1.000
CL.Mix - CL.I == 0  0.6490428  0.7961567   0.815    1.000
(Adjusted p values reported -- bonferroni method)
```

ASV184

```
	 Simultaneous Tests for General Linear Hypotheses
```

```
Multiple Comparisons of Means: Tukey Contrasts

Fit: lm(formula = log_cop ~ Treatment, data = dsub)

Linear Hypotheses:
                  Estimate Std. Error t value Pr(>|t|)
CL.F - CL.C == 0    4.448e-16  2.434e-01   0.000        1
CL.H - CL.C == 0    4.404e-16  2.434e-01   0.000        1
CL.I - CL.C == 0    4.079e-16  2.434e-01   0.000        1
CL.Mix - CL.C == 0  3.848e-01  2.434e-01   1.581        1
CL.H - CL.F == 0   -4.386e-18  2.434e-01   0.000        1
CL.I - CL.F == 0   -3.691e-17  2.434e-01   0.000        1
CL.Mix - CL.F == 0  3.848e-01  2.434e-01   1.581        1
CL.I - CL.H == 0   -3.252e-17  2.434e-01   0.000        1
CL.Mix - CL.H == 0  3.848e-01  2.434e-01   1.581        1
CL.Mix - CL.I == 0  3.848e-01  2.434e-01   1.581        1
(Adjusted p values reported -- bonferroni method)
```

ASV188

```
       Simultaneous Tests for General Linear Hypotheses

Multiple Comparisons of Means: Tukey Contrasts

Fit: lm(formula = log_cop ~ Treatment, data = dsub)

Linear Hypotheses:
                  Estimate Std. Error t value Pr(>|t|)
CL.F - CL.C == 0    2.324e-16  1.565e-01   0.000        1
CL.H - CL.C == 0    2.009e-16  1.565e-01   0.000        1
CL.I - CL.C == 0    1.700e-16  1.565e-01   0.000        1
CL.Mix - CL.C == 0  2.475e-01  1.565e-01   1.581        1
CL.H - CL.F == 0   -3.148e-17  1.565e-01   0.000        1
```

```
CL.I - CL.F == 0   -6.239e-17  1.565e-01   0.000        1
CL.Mix - CL.F == 0  2.475e-01  1.565e-01   1.581        1
CL.I - CL.H == 0   -3.091e-17  1.565e-01   0.000        1
CL.Mix - CL.H == 0  2.475e-01  1.565e-01   1.581        1
CL.Mix - CL.I == 0  2.475e-01  1.565e-01   1.581        1
(Adjusted p values reported -- bonferroni method)
```

```
ASV19
       Simultaneous Tests for General Linear Hypotheses
Multiple Comparisons of Means: Tukey Contrasts
Fit: lm(formula = log_cop ~ Treatment, data = dsub)

Linear Hypotheses:
                  Estimate Std. Error t value Pr(>|t|)
CL.F - CL.C == 0   -8.718e-01  7.234e-01  -1.205  1.00000
CL.H - CL.C == 0   -2.864e+00  7.234e-01  -3.959  0.00169 **
CL.I - CL.C == 0   -2.864e+00  7.234e-01  -3.959  0.00169 **
CL.Mix - CL.C == 0 -2.864e+00  7.234e-01  -3.959  0.00169 **
CL.H - CL.F == 0   -1.992e+00  7.234e-01  -2.754  0.07381 .
CL.I - CL.F == 0   -1.992e+00  7.234e-01  -2.754  0.07381 .
CL.Mix - CL.F == 0 -1.992e+00  7.234e-01  -2.754  0.07381 .
CL.I - CL.H == 0    4.441e-16  7.234e-01   0.000  1.00000
CL.Mix - CL.H == 0  1.332e-15  7.234e-01   0.000  1.00000
CL.Mix - CL.I == 0  8.882e-16  7.234e-01   0.000  1.00000
---
Signif. codes:  0 ‘***’ 0.001 ‘**’ 0.01 ‘*’ 0.05 ‘.’ 0.1 ‘ ’ 1
(Adjusted p values reported -- bonferroni method)
```

ASV196

```
       Simultaneous Tests for General Linear Hypotheses

Multiple Comparisons of Means: Tukey Contrasts

Fit: lm(formula = log_cop ~ Treatment, data = dsub)

Linear Hypotheses:
                  Estimate Std. Error t value Pr(>|t|)
CL.F - CL.C == 0    1.806e-17  1.950e-01   0.000        1
CL.H - CL.C == 0    3.205e-16  1.950e-01   0.000        1
CL.I - CL.C == 0    3.084e-01  1.950e-01   1.581        1
CL.Mix - CL.C == 0  2.159e-16  1.950e-01   0.000        1
CL.H - CL.F == 0    3.024e-16  1.950e-01   0.000        1
CL.I - CL.F == 0    3.084e-01  1.950e-01   1.581        1
CL.Mix - CL.F == 0  1.978e-16  1.950e-01   0.000        1
CL.I - CL.H == 0    3.084e-01  1.950e-01   1.581        1
CL.Mix - CL.H == 0 -1.046e-16  1.950e-01   0.000        1
CL.Mix - CL.I == 0 -3.084e-01  1.950e-01  -1.581        1
(Adjusted p values reported -- bonferroni method)
```

ASV2

```
       Simultaneous Tests for General Linear Hypotheses

Multiple Comparisons of Means: Tukey Contrasts

Fit: lm(formula = log_cop ~ Treatment, data = dsub)

Linear Hypotheses:
                Estimate Std. Error t value Pr(>|t|)
CL.F - CL.C == 0    0.23417    0.16884   1.387    1.000
CL.H - CL.C == 0    0.42664    0.16884   2.527    0.136
```

```
CL.I - CL.C == 0    0.28313    0.16884   1.677    0.977
CL.Mix - CL.C == 0  0.35724    0.16884   2.116    0.377
CL.H - CL.F == 0    0.19247    0.16884   1.140    1.000
CL.I - CL.F == 0    0.04896    0.16884   0.290    1.000
CL.Mix - CL.F == 0  0.12306    0.16884   0.729    1.000
CL.I - CL.H == 0   -0.14351    0.16884  -0.850    1.000
CL.Mix - CL.H == 0 -0.06941    0.16884  -0.411    1.000
CL.Mix - CL.I == 0  0.07411    0.16884   0.439    1.000
(Adjusted p values reported -- bonferroni method)
```

```
ASV20
       Simultaneous Tests for General Linear Hypotheses
Multiple Comparisons of Means: Tukey Contrasts
Fit: lm(formula = log_cop ~ Treatment, data = dsub)

Linear Hypotheses:
                  Estimate Std. Error t value Pr(>|t|)
CL.F - CL.C == 0   -2.160e+00  5.670e-01  -3.810  0.00282 **
CL.H - CL.C == 0   -2.160e+00  5.670e-01  -3.810  0.00282 **
CL.I - CL.C == 0   -1.820e+00  5.670e-01  -3.211  0.01949 *
CL.Mix - CL.C == 0 -2.160e+00  5.670e-01  -3.810  0.00282 **
CL.H - CL.F == 0   -1.776e-15  5.670e-01   0.000  1.00000
CL.I - CL.F == 0    3.397e-01  5.670e-01   0.599  1.00000
CL.Mix - CL.F == 0 -4.441e-16  5.670e-01   0.000  1.00000
CL.I - CL.H == 0    3.397e-01  5.670e-01   0.599  1.00000
CL.Mix - CL.H == 0  1.332e-15  5.670e-01   0.000  1.00000
CL.Mix - CL.I == 0 -3.397e-01  5.670e-01  -0.599  1.00000
---
```

```
Signif. codes:  0 ‘***’ 0.001 ‘**’ 0.01 ‘*’ 0.05 ‘.’ 0.1 ‘ ’ 1
(Adjusted p values reported -- bonferroni method)
```

ASV200

```
	 Simultaneous Tests for General Linear Hypotheses

Multiple Comparisons of Means: Tukey Contrasts

Fit: lm(formula = log_cop ~ Treatment, data = dsub)

Linear Hypotheses:
                 Estimate Std. Error t value Pr(>|t|)
CL.F - CL.C == 0   -5.384e-03  2.315e-01  -0.023        1
CL.H - CL.C == 0   -2.615e-01  2.315e-01  -1.130        1
CL.I - CL.C == 0   -2.615e-01  2.315e-01  -1.130        1
CL.Mix - CL.C == 0 -2.615e-01  2.315e-01  -1.130        1
CL.H - CL.F == 0   -2.561e-01  2.315e-01  -1.106        1
CL.I - CL.F == 0   -2.561e-01  2.315e-01  -1.106        1
CL.Mix - CL.F == 0 -2.561e-01  2.315e-01  -1.106        1
CL.I - CL.H == 0    5.551e-17  2.315e-01   0.000        1
CL.Mix - CL.H == 0  1.665e-16  2.315e-01   0.000        1
CL.Mix - CL.I == 0  1.110e-16  2.315e-01   0.000        1
(Adjusted p values reported -- bonferroni method)
```

ASV205

```
	 Simultaneous Tests for General Linear Hypotheses

Multiple Comparisons of Means: Tukey Contrasts

Fit: lm(formula = log_cop ~ Treatment, data = dsub)

Linear Hypotheses:
```

```
                    Estimate Std. Error t value Pr(>|t|)
CL.F - CL.C == 0    0.000e+00  1.956e-01   0.000        1
CL.H - CL.C == 0    3.092e-01  1.956e-01   1.581        1
CL.I - CL.C == 0    2.309e-17  1.956e-01   0.000        1
CL.Mix - CL.C == 0  1.429e-17  1.956e-01   0.000        1
CL.H - CL.F == 0    3.092e-01  1.956e-01   1.581        1
CL.I - CL.F == 0    2.309e-17  1.956e-01   0.000        1
CL.Mix - CL.F == 0  1.429e-17  1.956e-01   0.000        1
CL.I - CL.H == 0   -3.092e-01  1.956e-01  -1.581        1
CL.Mix - CL.H == 0 -3.092e-01  1.956e-01  -1.581        1
CL.Mix - CL.I == 0 -8.801e-18  1.956e-01   0.000        1
(Adjusted p values reported -- bonferroni method)
```

ASV209

```
         Simultaneous Tests for General Linear Hypotheses
Multiple Comparisons of Means: Tukey Contrasts
Fit: lm(formula = log_cop ~ Treatment, data = dsub)

Linear Hypotheses:
                    Estimate Std. Error t value Pr(>|t|)
CL.F - CL.C == 0    2.423e-01  1.532e-01   1.581        1
CL.H - CL.C == 0    1.711e-17  1.532e-01   0.000        1
CL.I - CL.C == 0    2.327e-17  1.532e-01   0.000        1
CL.Mix - CL.C == 0  1.251e-16  1.532e-01   0.000        1
CL.H - CL.F == 0   -2.423e-01  1.532e-01  -1.581        1
CL.I - CL.F == 0   -2.423e-01  1.532e-01  -1.581        1
CL.Mix - CL.F == 0 -2.423e-01  1.532e-01  -1.581        1
CL.I - CL.H == 0    6.153e-18  1.532e-01   0.000        1
```

```
CL.Mix - CL.H == 0  1.080e-16  1.532e-01   0.000        1
CL.Mix - CL.I == 0  1.019e-16  1.532e-01   0.000        1
(Adjusted p values reported -- bonferroni method)
```

ASV21

```
       Simultaneous Tests for General Linear Hypotheses

Multiple Comparisons of Means: Tukey Contrasts

Fit: lm(formula = log_cop ~ Treatment, data = dsub)

Linear Hypotheses:
                  Estimate Std. Error t value Pr(>|t|)
CL.F - CL.C == 0   -1.407e+00  4.014e-01  -3.506  0.00772 **
CL.H - CL.C == 0   -1.407e+00  4.014e-01  -3.506  0.00772 **
CL.I - CL.C == 0   -1.407e+00  4.014e-01  -3.506  0.00772 **
CL.Mix - CL.C == 0 -1.407e+00  4.014e-01  -3.506  0.00772 **
CL.H - CL.F == 0   -1.110e-15  4.014e-01   0.000  1.00000
CL.I - CL.F == 0   -2.220e-16  4.014e-01   0.000  1.00000
CL.Mix - CL.F == 0 -4.441e-16  4.014e-01   0.000  1.00000
CL.I - CL.H == 0    8.882e-16  4.014e-01   0.000  1.00000
CL.Mix - CL.H == 0  6.661e-16  4.014e-01   0.000  1.00000
CL.Mix - CL.I == 0 -2.220e-16  4.014e-01   0.000  1.00000
---
Signif. codes:  0 ‘***’ 0.001 ‘**’ 0.01 ‘*’ 0.05 ‘.’ 0.1 ‘ ’ 1
(Adjusted p values reported -- bonferroni method)
```

ASV217

```
       Simultaneous Tests for General Linear Hypotheses
```

Multiple Comparisons of Means: Tukey Contrasts

Fit: lm(formula = log_cop ~ Treatment, data = dsub)

Linear Hypotheses:

	Estimate	Std. Error	t value	Pr(>|t|)
CL.F - CL.C == 0	5.208e-17	1.960e-01	0.000	1
CL.H - CL.C == 0	2.083e-16	1.960e-01	0.000	1
CL.I - CL.C == 0	3.099e-01	1.960e-01	1.581	1
CL.Mix - CL.C == 0	5.888e-17	1.960e-01	0.000	1
CL.H - CL.F == 0	1.562e-16	1.960e-01	0.000	1
CL.I - CL.F == 0	3.099e-01	1.960e-01	1.581	1
CL.Mix - CL.F == 0	6.798e-18	1.960e-01	0.000	1
CL.I - CL.H == 0	3.099e-01	1.960e-01	1.581	1
CL.Mix - CL.H == 0	-1.494e-16	1.960e-01	0.000	1
CL.Mix - CL.I == 0	-3.099e-01	1.960e-01	-1.581	1

(Adjusted p values reported -- bonferroni method)

ASV22

Simultaneous Tests for General Linear Hypotheses

Multiple Comparisons of Means: Tukey Contrasts

Fit: lm(formula = log_cop ~ Treatment, data = dsub)

Linear Hypotheses:

	Estimate	Std. Error	t value	Pr(>|t|)
CL.F - CL.C == 0	-0.27234	0.61312	-0.444	1.000
CL.H - CL.C == 0	0.61659	0.61312	1.006	1.000
CL.I - CL.C == 0	0.94209	0.61312	1.537	1.000
CL.Mix - CL.C == 0	0.99932	0.61312	1.630	1.000
CL.H - CL.F == 0	0.88893	0.61312	1.450	1.000

```
CL.I - CL.F == 0    1.21443    0.61312   1.981    0.513
CL.Mix - CL.F == 0  1.27166    0.61312   2.074    0.415
CL.I - CL.H == 0    0.32550    0.61312   0.531    1.000
CL.Mix - CL.H == 0  0.38273    0.61312   0.624    1.000
CL.Mix - CL.I == 0  0.05723    0.61312   0.093    1.000
(Adjusted p values reported -- bonferroni method)
```

ASV23

```
      Simultaneous Tests for General Linear Hypotheses
Multiple Comparisons of Means: Tukey Contrasts
Fit: lm(formula = log_cop ~ Treatment, data = dsub)

Linear Hypotheses:
                  Estimate Std. Error t value Pr(>|t|)
CL.F - CL.C == 0    3.845e-01  4.793e-01   0.802        1
CL.H - CL.C == 0    7.796e-01  4.793e-01   1.627        1
CL.I - CL.C == 0   -1.020e-16  4.793e-01   0.000        1
CL.Mix - CL.C == 0  3.648e-01  4.793e-01   0.761        1
CL.H - CL.F == 0    3.952e-01  4.793e-01   0.825        1
CL.I - CL.F == 0   -3.845e-01  4.793e-01  -0.802        1
CL.Mix - CL.F == 0 -1.970e-02  4.793e-01  -0.041        1
CL.I - CL.H == 0   -7.796e-01  4.793e-01  -1.627        1
CL.Mix - CL.H == 0 -4.149e-01  4.793e-01  -0.866        1
CL.Mix - CL.I == 0  3.648e-01  4.793e-01   0.761        1
(Adjusted p values reported -- bonferroni method)
```

ASV231

```
	 Simultaneous Tests for General Linear Hypotheses

Multiple Comparisons of Means: Tukey Contrasts

Fit: lm(formula = log_cop ~ Treatment, data = dsub)

Linear Hypotheses:
                  Estimate Std. Error t value Pr(>|t|)
CL.F - CL.C == 0   -5.173e-01  2.235e-01  -2.314    0.234
CL.H - CL.C == 0   -5.173e-01  2.235e-01  -2.314    0.234
CL.I - CL.C == 0   -5.173e-01  2.235e-01  -2.314    0.234
CL.Mix - CL.C == 0 -5.173e-01  2.235e-01  -2.314    0.234
CL.H - CL.F == 0   -5.551e-16  2.235e-01   0.000    1.000
CL.I - CL.F == 0   -3.331e-16  2.235e-01   0.000    1.000
CL.Mix - CL.F == 0 -3.331e-16  2.235e-01   0.000    1.000
CL.I - CL.H == 0    2.220e-16  2.235e-01   0.000    1.000
CL.Mix - CL.H == 0  2.220e-16  2.235e-01   0.000    1.000
CL.Mix - CL.I == 0  0.000e+00  2.235e-01   0.000    1.000
(Adjusted p values reported -- bonferroni method)



ASV25

	 Simultaneous Tests for General Linear Hypotheses

Multiple Comparisons of Means: Tukey Contrasts

Fit: lm(formula = log_cop ~ Treatment, data = dsub)

Linear Hypotheses:
                  Estimate Std. Error t value Pr(>|t|)
CL.F - CL.C == 0    0.33188    0.57514   0.577    1.000
CL.H - CL.C == 0    0.24561    0.57514   0.427    1.000
CL.I - CL.C == 0    0.97975    0.57514   1.703    0.926
CL.Mix - CL.C == 0  1.06666    0.57514   1.855    0.676
```

```
CL.H - CL.F == 0   -0.08627    0.57514  -0.150    1.000
CL.I - CL.F == 0    0.64787    0.57514   1.126    1.000
CL.Mix - CL.F == 0  0.73478    0.57514   1.278    1.000
CL.I - CL.H == 0    0.73413    0.57514   1.276    1.000
CL.Mix - CL.H == 0  0.82105    0.57514   1.428    1.000
CL.Mix - CL.I == 0  0.08691    0.57514   0.151    1.000
(Adjusted p values reported -- bonferroni method)
```

ASV250

```
       Simultaneous Tests for General Linear Hypotheses

Multiple Comparisons of Means: Tukey Contrasts

Fit: lm(formula = log_cop ~ Treatment, data = dsub)

Linear Hypotheses:
                  Estimate Std. Error t value Pr(>|t|)
CL.F - CL.C == 0    1.176e-16  1.957e-01   0.000        1
CL.H - CL.C == 0    1.602e-16  1.957e-01   0.000        1
CL.I - CL.C == 0    3.094e-01  1.957e-01   1.581        1
CL.Mix - CL.C == 0  1.766e-16  1.957e-01   0.000        1
CL.H - CL.F == 0    4.261e-17  1.957e-01   0.000        1
CL.I - CL.F == 0    3.094e-01  1.957e-01   1.581        1
CL.Mix - CL.F == 0  5.899e-17  1.957e-01   0.000        1
CL.I - CL.H == 0    3.094e-01  1.957e-01   1.581        1
CL.Mix - CL.H == 0  1.639e-17  1.957e-01   0.000        1
CL.Mix - CL.I == 0 -3.094e-01  1.957e-01  -1.581        1
(Adjusted p values reported -- bonferroni method)
```

```
ASV256

	 Simultaneous Tests for General Linear Hypotheses

Multiple Comparisons of Means: Tukey Contrasts

Fit: lm(formula = log_cop ~ Treatment, data = dsub)

Linear Hypotheses:
                  Estimate Std. Error t value Pr(>|t|)
CL.F - CL.C == 0   -1.552e-17  1.774e-01   0.000        1
CL.H - CL.C == 0    2.806e-01  1.774e-01   1.581        1
CL.I - CL.C == 0    1.848e-17  1.774e-01   0.000        1
CL.Mix - CL.C == 0  1.658e-17  1.774e-01   0.000        1
CL.H - CL.F == 0    2.806e-01  1.774e-01   1.581        1
CL.I - CL.F == 0    3.400e-17  1.774e-01   0.000        1
CL.Mix - CL.F == 0  3.210e-17  1.774e-01   0.000        1
CL.I - CL.H == 0   -2.806e-01  1.774e-01  -1.581        1
CL.Mix - CL.H == 0 -2.806e-01  1.774e-01  -1.581        1
CL.Mix - CL.I == 0 -1.897e-18  1.774e-01   0.000        1
(Adjusted p values reported -- bonferroni method)


ASV26

	 Simultaneous Tests for General Linear Hypotheses

Multiple Comparisons of Means: Tukey Contrasts

Fit: lm(formula = log_cop ~ Treatment, data = dsub)

Linear Hypotheses:
                  Estimate Std. Error t value Pr(>|t|)
CL.F - CL.C == 0   -0.88279    0.61773  -1.429    1.000
CL.H - CL.C == 0    0.07826    0.61773   0.127    1.000
CL.I - CL.C == 0    0.63043    0.61773   1.021    1.000
```

```
CL.Mix - CL.C == 0 -0.16172    0.61773  -0.262    1.000
CL.H - CL.F == 0    0.96104    0.61773   1.556    1.000
CL.I - CL.F == 0    1.51322    0.61773   2.450    0.166
CL.Mix - CL.F == 0  0.72107    0.61773   1.167    1.000
CL.I - CL.H == 0    0.55218    0.61773   0.894    1.000
CL.Mix - CL.H == 0 -0.23998    0.61773  -0.388    1.000
CL.Mix - CL.I == 0 -0.79215    0.61773  -1.282    1.000
(Adjusted p values reported -- bonferroni method)
```

ASV275

```
      Simultaneous Tests for General Linear Hypotheses
Multiple Comparisons of Means: Tukey Contrasts
Fit: lm(formula = log_cop ~ Treatment, data = dsub)
Linear Hypotheses:
                 Estimate Std. Error t value Pr(>|t|)
CL.F - CL.C == 0    1.957e-16  1.899e-01   0.000        1
CL.H - CL.C == 0    2.243e-16  1.899e-01   0.000        1
CL.I - CL.C == 0    3.003e-01  1.899e-01   1.581        1
CL.Mix - CL.C == 0  2.748e-16  1.899e-01   0.000        1
CL.H - CL.F == 0    2.862e-17  1.899e-01   0.000        1
CL.I - CL.F == 0    3.003e-01  1.899e-01   1.581        1
CL.Mix - CL.F == 0  7.904e-17  1.899e-01   0.000        1
CL.I - CL.H == 0    3.003e-01  1.899e-01   1.581        1
CL.Mix - CL.H == 0  5.042e-17  1.899e-01   0.000        1
CL.Mix - CL.I == 0 -3.003e-01  1.899e-01  -1.581        1
(Adjusted p values reported -- bonferroni method)
```

ASV28

```
	 Simultaneous Tests for General Linear Hypotheses

Multiple Comparisons of Means: Tukey Contrasts

Fit: lm(formula = log_cop ~ Treatment, data = dsub)

Linear Hypotheses:
                 Estimate Std. Error t value Pr(>|t|)
CL.F - CL.C == 0     0.7257     0.7540   0.962    1.000
CL.H - CL.C == 0     1.2276     0.7540   1.628    1.000
CL.I - CL.C == 0     1.5511     0.7540   2.057    0.431
CL.Mix - CL.C == 0   1.9458     0.7540   2.581    0.118
CL.H - CL.F == 0     0.5019     0.7540   0.666    1.000
CL.I - CL.F == 0     0.8254     0.7540   1.095    1.000
CL.Mix - CL.F == 0   1.2201     0.7540   1.618    1.000
CL.I - CL.H == 0     0.3235     0.7540   0.429    1.000
CL.Mix - CL.H == 0   0.7182     0.7540   0.953    1.000
CL.Mix - CL.I == 0   0.3947     0.7540   0.523    1.000
(Adjusted p values reported -- bonferroni method)
```

ASV286

```
	 Simultaneous Tests for General Linear Hypotheses

Multiple Comparisons of Means: Tukey Contrasts

Fit: lm(formula = log_cop ~ Treatment, data = dsub)

Linear Hypotheses:
                   Estimate Std. Error t value Pr(>|t|)
CL.F - CL.C == 0   -2.497e-01  1.579e-01  -1.581        1
CL.H - CL.C == 0   -2.497e-01  1.579e-01  -1.581        1
CL.I - CL.C == 0   -2.497e-01  1.579e-01  -1.581        1
```

```
CL.Mix - CL.C == 0 -2.497e-01  1.579e-01  -1.581        1
CL.H - CL.F == 0   -1.388e-16  1.579e-01   0.000        1
CL.I - CL.F == 0   -5.551e-17  1.579e-01   0.000        1
CL.Mix - CL.F == 0 -5.551e-17  1.579e-01   0.000        1
CL.I - CL.H == 0    8.327e-17  1.579e-01   0.000        1
CL.Mix - CL.H == 0  8.327e-17  1.579e-01   0.000        1
CL.Mix - CL.I == 0  0.000e+00  1.579e-01   0.000        1
(Adjusted p values reported -- bonferroni method)
```

```
ASV287
       Simultaneous Tests for General Linear Hypotheses
Multiple Comparisons of Means: Tukey Contrasts
Fit: lm(formula = log_cop ~ Treatment, data = dsub)

Linear Hypotheses:
                    Estimate Std. Error t value Pr(>|t|)
CL.F - CL.C == 0    2.852e-01  1.804e-01   1.581        1
CL.H - CL.C == 0    1.021e-17  1.804e-01   0.000        1
CL.I - CL.C == 0    7.916e-18  1.804e-01   0.000        1
CL.Mix - CL.C == 0 -1.586e-16  1.804e-01   0.000        1
CL.H - CL.F == 0   -2.852e-01  1.804e-01  -1.581        1
CL.I - CL.F == 0   -2.852e-01  1.804e-01  -1.581        1
CL.Mix - CL.F == 0 -2.852e-01  1.804e-01  -1.581        1
CL.I - CL.H == 0   -2.298e-18  1.804e-01   0.000        1
CL.Mix - CL.H == 0 -1.688e-16  1.804e-01   0.000        1
CL.Mix - CL.I == 0 -1.665e-16  1.804e-01   0.000        1
(Adjusted p values reported -- bonferroni method)
```

ASV289

Simultaneous Tests for General Linear Hypotheses

Multiple Comparisons of Means: Tukey Contrasts

Fit: lm(formula = log_cop ~ Treatment, data = dsub)

Linear Hypotheses:

	Estimate	Std. Error	t value	Pr(>|t|)
CL.F - CL.C == 0	2.898e-01	1.833e-01	1.581	1
CL.H - CL.C == 0	2.613e-17	1.833e-01	0.000	1
CL.I - CL.C == 0	3.222e-17	1.833e-01	0.000	1
CL.Mix - CL.C == 0	2.384e-16	1.833e-01	0.000	1
CL.H - CL.F == 0	-2.898e-01	1.833e-01	-1.581	1
CL.I - CL.F == 0	-2.898e-01	1.833e-01	-1.581	1
CL.Mix - CL.F == 0	-2.898e-01	1.833e-01	-1.581	1
CL.I - CL.H == 0	6.087e-18	1.833e-01	0.000	1
CL.Mix - CL.H == 0	2.122e-16	1.833e-01	0.000	1
CL.Mix - CL.I == 0	2.061e-16	1.833e-01	0.000	1

(Adjusted p values reported -- bonferroni method)

ASV297

Simultaneous Tests for General Linear Hypotheses

Multiple Comparisons of Means: Tukey Contrasts

Fit: lm(formula = log_cop ~ Treatment, data = dsub)

Linear Hypotheses:

	Estimate	Std. Error	t value	Pr(>|t|)
CL.F - CL.C == 0	2.501e-01	1.582e-01	1.581	1
CL.H - CL.C == 0	7.390e-18	1.582e-01	0.000	1

```
CL.I - CL.C == 0    9.760e-18  1.582e-01   0.000        1
CL.Mix - CL.C == 0  4.840e-17  1.582e-01   0.000        1
CL.H - CL.F == 0   -2.501e-01  1.582e-01  -1.581        1
CL.I - CL.F == 0   -2.501e-01  1.582e-01  -1.581        1
CL.Mix - CL.F == 0 -2.501e-01  1.582e-01  -1.581        1
CL.I - CL.H == 0    2.370e-18  1.582e-01   0.000        1
CL.Mix - CL.H == 0  4.101e-17  1.582e-01   0.000        1
CL.Mix - CL.I == 0  3.864e-17  1.582e-01   0.000        1
(Adjusted p values reported -- bonferroni method)
```

ASV3

```
      Simultaneous Tests for General Linear Hypotheses
Multiple Comparisons of Means: Tukey Contrasts
Fit: lm(formula = log_cop ~ Treatment, data = dsub)

Linear Hypotheses:
               Estimate Std. Error t value Pr(>|t|)
CL.F - CL.C == 0    -1.1156     1.0248  -1.089    1.000
CL.H - CL.C == 0    -0.1695     1.0248  -0.165    1.000
CL.I - CL.C == 0    -0.6893     1.0248  -0.673    1.000
CL.Mix - CL.C == 0   1.5133     1.0248   1.477    1.000
CL.H - CL.F == 0     0.9462     1.0248   0.923    1.000
CL.I - CL.F == 0     0.4263     1.0248   0.416    1.000
CL.Mix - CL.F == 0   2.6289     1.0248   2.565    0.123
CL.I - CL.H == 0    -0.5199     1.0248  -0.507    1.000
CL.Mix - CL.H == 0   1.6827     1.0248   1.642    1.000
CL.Mix - CL.I == 0   2.2026     1.0248   2.149    0.348
(Adjusted p values reported -- bonferroni method)
```

ASV30

Simultaneous Tests for General Linear Hypotheses

Multiple Comparisons of Means: Tukey Contrasts

Fit: lm(formula = log_cop ~ Treatment, data = dsub)

Linear Hypotheses:

	Estimate	Std. Error	t value	Pr(>|t|)
CL.F - CL.C == 0	0.3216	0.6994	0.460	1.000
CL.H - CL.C == 0	1.1982	0.6994	1.713	0.908
CL.I - CL.C == 0	1.3257	0.6994	1.896	0.619
CL.Mix - CL.C == 0	1.6563	0.6994	2.368	0.205
CL.H - CL.F == 0	0.8766	0.6994	1.253	1.000
CL.I - CL.F == 0	1.0041	0.6994	1.436	1.000
CL.Mix - CL.F == 0	1.3347	0.6994	1.908	0.602
CL.I - CL.H == 0	0.1275	0.6994	0.182	1.000
CL.Mix - CL.H == 0	0.4580	0.6994	0.655	1.000
CL.Mix - CL.I == 0	0.3305	0.6994	0.473	1.000

(Adjusted p values reported -- bonferroni method)

ASV300

Simultaneous Tests for General Linear Hypotheses

Multiple Comparisons of Means: Tukey Contrasts

Fit: lm(formula = log_cop ~ Treatment, data = dsub)

Linear Hypotheses:

	Estimate	Std. Error	t value	Pr(>|t|)
CL.F - CL.C == 0	-3.052e-01	1.930e-01	-1.581	1

```
CL.H - CL.C == 0   -3.052e-01  1.930e-01  -1.581        1
CL.I - CL.C == 0   -3.052e-01  1.930e-01  -1.581        1
CL.Mix - CL.C == 0 -3.052e-01  1.930e-01  -1.581        1
CL.H - CL.F == 0   -2.220e-16  1.930e-01   0.000        1
CL.I - CL.F == 0   -1.665e-16  1.930e-01   0.000        1
CL.Mix - CL.F == 0 -2.220e-16  1.930e-01   0.000        1
CL.I - CL.H == 0    5.551e-17  1.930e-01   0.000        1
CL.Mix - CL.H == 0  0.000e+00  1.930e-01   0.000        1
CL.Mix - CL.I == 0 -5.551e-17  1.930e-01   0.000        1
(Adjusted p values reported -- bonferroni method)
```

ASV305

```
       Simultaneous Tests for General Linear Hypotheses
Multiple Comparisons of Means: Tukey Contrasts
Fit: lm(formula = log_cop ~ Treatment, data = dsub)

Linear Hypotheses:
                  Estimate Std. Error t value Pr(>|t|)
CL.F - CL.C == 0    3.377e-01  2.136e-01   1.581        1
CL.H - CL.C == 0    4.814e-17  2.136e-01   0.000        1
CL.I - CL.C == 0    3.512e-17  2.136e-01   0.000        1
CL.Mix - CL.C == 0  4.280e-17  2.136e-01   0.000        1
CL.H - CL.F == 0   -3.377e-01  2.136e-01  -1.581        1
CL.I - CL.F == 0   -3.377e-01  2.136e-01  -1.581        1
CL.Mix - CL.F == 0 -3.377e-01  2.136e-01  -1.581        1
CL.I - CL.H == 0   -1.303e-17  2.136e-01   0.000        1
CL.Mix - CL.H == 0 -5.340e-18  2.136e-01   0.000        1
CL.Mix - CL.I == 0  7.686e-18  2.136e-01   0.000        1
```

```
(Adjusted p values reported -- bonferroni method)
```

ASV306

```
	 Simultaneous Tests for General Linear Hypotheses

Multiple Comparisons of Means: Tukey Contrasts

Fit: lm(formula = log_cop ~ Treatment, data = dsub)

Linear Hypotheses:
                  Estimate Std. Error t value Pr(>|t|)
CL.F - CL.C == 0    7.758e-17  2.005e-01   0.000        1
CL.H - CL.C == 0    3.170e-01  2.005e-01   1.581        1
CL.I - CL.C == 0    3.186e-17  2.005e-01   0.000        1
CL.Mix - CL.C == 0  1.340e-17  2.005e-01   0.000        1
CL.H - CL.F == 0    3.170e-01  2.005e-01   1.581        1
CL.I - CL.F == 0   -4.572e-17  2.005e-01   0.000        1
CL.Mix - CL.F == 0 -6.418e-17  2.005e-01   0.000        1
CL.I - CL.H == 0   -3.170e-01  2.005e-01  -1.581        1
CL.Mix - CL.H == 0 -3.170e-01  2.005e-01  -1.581        1
CL.Mix - CL.I == 0 -1.846e-17  2.005e-01   0.000        1
(Adjusted p values reported -- bonferroni method)
```

ASV31

```
	 Simultaneous Tests for General Linear Hypotheses

Multiple Comparisons of Means: Tukey Contrasts

Fit: lm(formula = log_cop ~ Treatment, data = dsub)

Linear Hypotheses:
                Estimate Std. Error t value Pr(>|t|)
```

```
CL.F - CL.C == 0     0.2768     0.8577   0.323        1
CL.H - CL.C == 0    -0.2445     0.8577  -0.285        1
CL.I - CL.C == 0     0.1020     0.8577   0.119        1
CL.Mix - CL.C == 0  -0.1019     0.8577  -0.119        1
CL.H - CL.F == 0    -0.5213     0.8577  -0.608        1
CL.I - CL.F == 0    -0.1748     0.8577  -0.204        1
CL.Mix - CL.F == 0  -0.3787     0.8577  -0.442        1
CL.I - CL.H == 0     0.3465     0.8577   0.404        1
CL.Mix - CL.H == 0   0.1426     0.8577   0.166        1
CL.Mix - CL.I == 0  -0.2039     0.8577  -0.238        1
(Adjusted p values reported -- bonferroni method)
```

ASV32

```
	 Simultaneous Tests for General Linear Hypotheses

Multiple Comparisons of Means: Tukey Contrasts

Fit: lm(formula = log_cop ~ Treatment, data = dsub)

Linear Hypotheses:
                   Estimate Std. Error t value Pr(>|t|)
CL.F - CL.C == 0    -0.6224     0.9517  -0.654        1
CL.H - CL.C == 0     0.3605     0.9517   0.379        1
CL.I - CL.C == 0     0.1181     0.9517   0.124        1
CL.Mix - CL.C == 0  -0.2488     0.9517  -0.261        1
CL.H - CL.F == 0     0.9829     0.9517   1.033        1
CL.I - CL.F == 0     0.7404     0.9517   0.778        1
CL.Mix - CL.F == 0   0.3736     0.9517   0.393        1
CL.I - CL.H == 0    -0.2424     0.9517  -0.255        1
CL.Mix - CL.H == 0  -0.6093     0.9517  -0.640        1
```

```
CL.Mix - CL.I == 0  -0.3669     0.9517  -0.385        1
(Adjusted p values reported -- bonferroni method)
```

ASV321

```
         Simultaneous Tests for General Linear Hypotheses

Multiple Comparisons of Means: Tukey Contrasts

Fit: lm(formula = log_cop ~ Treatment, data = dsub)

Linear Hypotheses:
                  Estimate Std. Error t value Pr(>|t|)
CL.F - CL.C == 0   -2.615e-01  1.654e-01  -1.581        1
CL.H - CL.C == 0   -2.615e-01  1.654e-01  -1.581        1
CL.I - CL.C == 0   -2.615e-01  1.654e-01  -1.581        1
CL.Mix - CL.C == 0 -2.615e-01  1.654e-01  -1.581        1
CL.H - CL.F == 0   -2.776e-16  1.654e-01   0.000        1
CL.I - CL.F == 0   -1.665e-16  1.654e-01   0.000        1
CL.Mix - CL.F == 0 -1.665e-16  1.654e-01   0.000        1
CL.I - CL.H == 0    1.110e-16  1.654e-01   0.000        1
CL.Mix - CL.H == 0  1.110e-16  1.654e-01   0.000        1
CL.Mix - CL.I == 0  0.000e+00  1.654e-01   0.000        1
(Adjusted p values reported -- bonferroni method)
```

ASV328

```
         Simultaneous Tests for General Linear Hypotheses

Multiple Comparisons of Means: Tukey Contrasts

Fit: lm(formula = log_cop ~ Treatment, data = dsub)

Linear Hypotheses:
```

```
                   Estimate Std. Error t value Pr(>|t|)
CL.F - CL.C == 0    1.051e-16  1.728e-01   0.000        1
CL.H - CL.C == 0    4.807e-17  1.728e-01   0.000        1
CL.I - CL.C == 0    2.732e-01  1.728e-01   1.581        1
CL.Mix - CL.C == 0  4.907e-17  1.728e-01   0.000        1
CL.H - CL.F == 0   -5.704e-17  1.728e-01   0.000        1
CL.I - CL.F == 0    2.732e-01  1.728e-01   1.581        1
CL.Mix - CL.F == 0 -5.605e-17  1.728e-01   0.000        1
CL.I - CL.H == 0    2.732e-01  1.728e-01   1.581        1
CL.Mix - CL.H == 0  9.913e-19  1.728e-01   0.000        1
CL.Mix - CL.I == 0 -2.732e-01  1.728e-01  -1.581        1
(Adjusted p values reported -- bonferroni method)
```

ASV329

```
       Simultaneous Tests for General Linear Hypotheses
Multiple Comparisons of Means: Tukey Contrasts
Fit: lm(formula = log_cop ~ Treatment, data = dsub)

Linear Hypotheses:
                   Estimate Std. Error t value Pr(>|t|)
CL.F - CL.C == 0    1.219e-16  1.737e-01   0.000        1
CL.H - CL.C == 0    2.083e-16  1.737e-01   0.000        1
CL.I - CL.C == 0    2.746e-01  1.737e-01   1.581        1
CL.Mix - CL.C == 0  3.925e-17  1.737e-01   0.000        1
CL.H - CL.F == 0    8.642e-17  1.737e-01   0.000        1
CL.I - CL.F == 0    2.746e-01  1.737e-01   1.581        1
CL.Mix - CL.F == 0 -8.265e-17  1.737e-01   0.000        1
CL.I - CL.H == 0    2.746e-01  1.737e-01   1.581        1
```

```
CL.Mix - CL.H == 0 -1.691e-16  1.737e-01   0.000        1
CL.Mix - CL.I == 0 -2.746e-01  1.737e-01  -1.581        1
(Adjusted p values reported -- bonferroni method)
```

ASV33

```
       Simultaneous Tests for General Linear Hypotheses
Multiple Comparisons of Means: Tukey Contrasts
Fit: lm(formula = log_cop ~ Treatment, data = dsub)

Linear Hypotheses:
               Estimate Std. Error t value Pr(>|t|)
CL.F - CL.C == 0     0.5743     1.0399   0.552    1.000
CL.H - CL.C == 0     2.2126     1.0399   2.128    0.366
CL.I - CL.C == 0     1.4248     1.0399   1.370    1.000
CL.Mix - CL.C == 0   1.1794     1.0399   1.134    1.000
CL.H - CL.F == 0     1.6383     1.0399   1.575    1.000
CL.I - CL.F == 0     0.8505     1.0399   0.818    1.000
CL.Mix - CL.F == 0   0.6050     1.0399   0.582    1.000
CL.I - CL.H == 0    -0.7878     1.0399  -0.758    1.000
CL.Mix - CL.H == 0  -1.0333     1.0399  -0.994    1.000
CL.Mix - CL.I == 0  -0.2455     1.0399  -0.236    1.000
(Adjusted p values reported -- bonferroni method)
```

ASV330

```
       Simultaneous Tests for General Linear Hypotheses
Multiple Comparisons of Means: Tukey Contrasts
Fit: lm(formula = log_cop ~ Treatment, data = dsub)
```

```
Linear Hypotheses:
                    Estimate Std. Error t value Pr(>|t|)
CL.F - CL.C == 0    2.511e-01  1.588e-01   1.581        1
CL.H - CL.C == 0    9.631e-18  1.588e-01   0.000        1
CL.I - CL.C == 0    1.331e-17  1.588e-01   0.000        1
CL.Mix - CL.C == 0  7.608e-17  1.588e-01   0.000        1
CL.H - CL.F == 0   -2.511e-01  1.588e-01  -1.581        1
CL.I - CL.F == 0   -2.511e-01  1.588e-01  -1.581        1
CL.Mix - CL.F == 0 -2.511e-01  1.588e-01  -1.581        1
CL.I - CL.H == 0    3.677e-18  1.588e-01   0.000        1
CL.Mix - CL.H == 0  6.645e-17  1.588e-01   0.000        1
CL.Mix - CL.I == 0  6.278e-17  1.588e-01   0.000        1
(Adjusted p values reported -- bonferroni method)
```

ASV331

```
	 Simultaneous Tests for General Linear Hypotheses

Multiple Comparisons of Means: Tukey Contrasts

Fit: lm(formula = log_cop ~ Treatment, data = dsub)

Linear Hypotheses:
                    Estimate Std. Error t value Pr(>|t|)
CL.F - CL.C == 0   -4.655e-17  1.655e-01   0.000        1
CL.H - CL.C == 0    2.617e-01  1.655e-01   1.581        1
CL.I - CL.C == 0    6.991e-18  1.655e-01   0.000        1
CL.Mix - CL.C == 0  1.362e-17  1.655e-01   0.000        1
CL.H - CL.F == 0    2.617e-01  1.655e-01   1.581        1
CL.I - CL.F == 0    5.354e-17  1.655e-01   0.000        1
CL.Mix - CL.F == 0  6.017e-17  1.655e-01   0.000        1
```

```
CL.I - CL.H == 0   -2.617e-01  1.655e-01  -1.581        1
CL.Mix - CL.H == 0 -2.617e-01  1.655e-01  -1.581        1
CL.Mix - CL.I == 0  6.628e-18  1.655e-01   0.000        1
(Adjusted p values reported -- bonferroni method)
```

ASV333

```
       Simultaneous Tests for General Linear Hypotheses
Multiple Comparisons of Means: Tukey Contrasts
Fit: lm(formula = log_cop ~ Treatment, data = dsub)

Linear Hypotheses:
                  Estimate Std. Error t value Pr(>|t|)
CL.F - CL.C == 0    2.772e-01  1.753e-01   1.581        1
CL.H - CL.C == 0    1.286e-17  1.753e-01   0.000        1
CL.I - CL.C == 0    2.350e-17  1.753e-01   0.000        1
CL.Mix - CL.C == 0  6.008e-17  1.753e-01   0.000        1
CL.H - CL.F == 0   -2.772e-01  1.753e-01  -1.581        1
CL.I - CL.F == 0   -2.772e-01  1.753e-01  -1.581        1
CL.Mix - CL.F == 0 -2.772e-01  1.753e-01  -1.581        1
CL.I - CL.H == 0    1.064e-17  1.753e-01   0.000        1
CL.Mix - CL.H == 0  4.722e-17  1.753e-01   0.000        1
CL.Mix - CL.I == 0  3.658e-17  1.753e-01   0.000        1
(Adjusted p values reported -- bonferroni method)
```

ASV334

```
       Simultaneous Tests for General Linear Hypotheses
Multiple Comparisons of Means: Tukey Contrasts
```

```
Fit: lm(formula = log_cop ~ Treatment, data = dsub)

Linear Hypotheses:
                  Estimate Std. Error t value Pr(>|t|)
CL.F - CL.C == 0    2.772e-01  1.753e-01   1.581        1
CL.H - CL.C == 0    1.286e-17  1.753e-01   0.000        1
CL.I - CL.C == 0    2.350e-17  1.753e-01   0.000        1
CL.Mix - CL.C == 0  6.008e-17  1.753e-01   0.000        1
CL.H - CL.F == 0   -2.772e-01  1.753e-01  -1.581        1
CL.I - CL.F == 0   -2.772e-01  1.753e-01  -1.581        1
CL.Mix - CL.F == 0 -2.772e-01  1.753e-01  -1.581        1
CL.I - CL.H == 0    1.064e-17  1.753e-01   0.000        1
CL.Mix - CL.H == 0  4.722e-17  1.753e-01   0.000        1
CL.Mix - CL.I == 0  3.658e-17  1.753e-01   0.000        1
(Adjusted p values reported -- bonferroni method)
```

ASV342

```
       Simultaneous Tests for General Linear Hypotheses
Multiple Comparisons of Means: Tukey Contrasts
Fit: lm(formula = log_cop ~ Treatment, data = dsub)

Linear Hypotheses:
                  Estimate Std. Error t value Pr(>|t|)
CL.F - CL.C == 0    1.607e-16  1.812e-01   0.000        1
CL.H - CL.C == 0    2.083e-16  1.812e-01   0.000        1
CL.I - CL.C == 0    2.864e-01  1.812e-01   1.581        1
CL.Mix - CL.C == 0  2.355e-16  1.812e-01   0.000        1
CL.H - CL.F == 0    4.763e-17  1.812e-01   0.000        1
CL.I - CL.F == 0    2.864e-01  1.812e-01   1.581        1
```

```
CL.Mix - CL.F == 0  7.482e-17  1.812e-01   0.000        1
CL.I - CL.H == 0    2.864e-01  1.812e-01   1.581        1
CL.Mix - CL.H == 0  2.719e-17  1.812e-01   0.000        1
CL.Mix - CL.I == 0 -2.864e-01  1.812e-01  -1.581        1
(Adjusted p values reported -- bonferroni method)
```

ASV349

```
	 Simultaneous Tests for General Linear Hypotheses

Multiple Comparisons of Means: Tukey Contrasts

Fit: lm(formula = log_cop ~ Treatment, data = dsub)

Linear Hypotheses:
                 Estimate Std. Error t value Pr(>|t|)
CL.F - CL.C == 0   -2.975e-01  1.881e-01  -1.581        1
CL.H - CL.C == 0   -2.975e-01  1.881e-01  -1.581        1
CL.I - CL.C == 0   -2.975e-01  1.881e-01  -1.581        1
CL.Mix - CL.C == 0 -2.975e-01  1.881e-01  -1.581        1
CL.H - CL.F == 0   -5.551e-17  1.881e-01   0.000        1
CL.I - CL.F == 0    4.441e-16  1.881e-01   0.000        1
CL.Mix - CL.F == 0  5.551e-17  1.881e-01   0.000        1
CL.I - CL.H == 0    4.996e-16  1.881e-01   0.000        1
CL.Mix - CL.H == 0  1.110e-16  1.881e-01   0.000        1
CL.Mix - CL.I == 0 -3.886e-16  1.881e-01   0.000        1
(Adjusted p values reported -- bonferroni method)
```

ASV353

```
	 Simultaneous Tests for General Linear Hypotheses
```

```
Multiple Comparisons of Means: Tukey Contrasts

Fit: lm(formula = log_cop ~ Treatment, data = dsub)

Linear Hypotheses:
                  Estimate Std. Error t value Pr(>|t|)
CL.F - CL.C == 0   -1.552e-17  1.876e-01   0.000        1
CL.H - CL.C == 0    2.966e-01  1.876e-01   1.581        1
CL.I - CL.C == 0    5.271e-17  1.876e-01   0.000        1
CL.Mix - CL.C == 0  7.714e-17  1.876e-01   0.000        1
CL.H - CL.F == 0    2.966e-01  1.876e-01   1.581        1
CL.I - CL.F == 0    6.822e-17  1.876e-01   0.000        1
CL.Mix - CL.F == 0  9.266e-17  1.876e-01   0.000        1
CL.I - CL.H == 0   -2.966e-01  1.876e-01  -1.581        1
CL.Mix - CL.H == 0 -2.966e-01  1.876e-01  -1.581        1
CL.Mix - CL.I == 0  2.443e-17  1.876e-01   0.000        1
(Adjusted p values reported -- bonferroni method)
```

ASV354

```
       Simultaneous Tests for General Linear Hypotheses

Multiple Comparisons of Means: Tukey Contrasts

Fit: lm(formula = log_cop ~ Treatment, data = dsub)

Linear Hypotheses:
                  Estimate Std. Error t value Pr(>|t|)
CL.F - CL.C == 0   -2.115e-17  1.801e-01   0.000        1
CL.H - CL.C == 0   -1.518e-16  1.801e-01   0.000        1
CL.I - CL.C == 0   -1.190e-16  1.801e-01   0.000        1
CL.Mix - CL.C == 0  2.848e-01  1.801e-01   1.581        1
CL.H - CL.F == 0   -1.307e-16  1.801e-01   0.000        1
```

```
CL.I - CL.F == 0   -9.782e-17  1.801e-01   0.000        1
CL.Mix - CL.F == 0  2.848e-01  1.801e-01   1.581        1
CL.I - CL.H == 0    3.285e-17  1.801e-01   0.000        1
CL.Mix - CL.H == 0  2.848e-01  1.801e-01   1.581        1
CL.Mix - CL.I == 0  2.848e-01  1.801e-01   1.581        1
(Adjusted p values reported -- bonferroni method)
```

ASV355

```
       Simultaneous Tests for General Linear Hypotheses

Multiple Comparisons of Means: Tukey Contrasts

Fit: lm(formula = log_cop ~ Treatment, data = dsub)

Linear Hypotheses:
                  Estimate Std. Error t value Pr(>|t|)
CL.F - CL.C == 0   -2.309e-01  1.460e-01  -1.581        1
CL.H - CL.C == 0   -2.309e-01  1.460e-01  -1.581        1
CL.I - CL.C == 0   -2.309e-01  1.460e-01  -1.581        1
CL.Mix - CL.C == 0 -2.309e-01  1.460e-01  -1.581        1
CL.H - CL.F == 0   -2.220e-16  1.460e-01   0.000        1
CL.I - CL.F == 0   -1.665e-16  1.460e-01   0.000        1
CL.Mix - CL.F == 0 -3.886e-16  1.460e-01   0.000        1
CL.I - CL.H == 0    5.551e-17  1.460e-01   0.000        1
CL.Mix - CL.H == 0 -1.665e-16  1.460e-01   0.000        1
CL.Mix - CL.I == 0 -2.220e-16  1.460e-01   0.000        1
(Adjusted p values reported -- bonferroni method)
```

ASV356

```
	 Simultaneous Tests for General Linear Hypotheses

Multiple Comparisons of Means: Tukey Contrasts

Fit: lm(formula = log_cop ~ Treatment, data = dsub)

Linear Hypotheses:
                   Estimate Std. Error t value Pr(>|t|)
CL.F - CL.C == 0   -2.309e-01  1.460e-01  -1.581        1
CL.H - CL.C == 0   -2.309e-01  1.460e-01  -1.581        1
CL.I - CL.C == 0   -2.309e-01  1.460e-01  -1.581        1
CL.Mix - CL.C == 0 -2.309e-01  1.460e-01  -1.581        1
CL.H - CL.F == 0   -2.220e-16  1.460e-01   0.000        1
CL.I - CL.F == 0   -1.665e-16  1.460e-01   0.000        1
CL.Mix - CL.F == 0 -3.886e-16  1.460e-01   0.000        1
CL.I - CL.H == 0    5.551e-17  1.460e-01   0.000        1
CL.Mix - CL.H == 0 -1.665e-16  1.460e-01   0.000        1
CL.Mix - CL.I == 0 -2.220e-16  1.460e-01   0.000        1
(Adjusted p values reported -- bonferroni method)
```

ASV357

```
	 Simultaneous Tests for General Linear Hypotheses

Multiple Comparisons of Means: Tukey Contrasts

Fit: lm(formula = log_cop ~ Treatment, data = dsub)

Linear Hypotheses:
                   Estimate Std. Error t value Pr(>|t|)
CL.F - CL.C == 0   -2.309e-01  1.460e-01  -1.581        1
CL.H - CL.C == 0   -2.309e-01  1.460e-01  -1.581        1
CL.I - CL.C == 0   -2.309e-01  1.460e-01  -1.581        1
CL.Mix - CL.C == 0 -2.309e-01  1.460e-01  -1.581        1
```

```
CL.H - CL.F == 0   -2.220e-16  1.460e-01   0.000        1
CL.I - CL.F == 0   -1.665e-16  1.460e-01   0.000        1
CL.Mix - CL.F == 0 -3.886e-16  1.460e-01   0.000        1
CL.I - CL.H == 0    5.551e-17  1.460e-01   0.000        1
CL.Mix - CL.H == 0 -1.665e-16  1.460e-01   0.000        1
CL.Mix - CL.I == 0 -2.220e-16  1.460e-01   0.000        1
(Adjusted p values reported -- bonferroni method)
```

ASV36

```
       Simultaneous Tests for General Linear Hypotheses

Multiple Comparisons of Means: Tukey Contrasts

Fit: lm(formula = log_cop ~ Treatment, data = dsub)

Linear Hypotheses:
               Estimate Std. Error t value Pr(>|t|)
CL.F - CL.C == 0    0.06885    1.10933   0.062        1
CL.H - CL.C == 0   -0.31979    1.10933  -0.288        1
CL.I - CL.C == 0    0.29250    1.10933   0.264        1
CL.Mix - CL.C == 0  0.04962    1.10933   0.045        1
CL.H - CL.F == 0   -0.38864    1.10933  -0.350        1
CL.I - CL.F == 0    0.22365    1.10933   0.202        1
CL.Mix - CL.F == 0 -0.01923    1.10933  -0.017        1
CL.I - CL.H == 0    0.61229    1.10933   0.552        1
CL.Mix - CL.H == 0  0.36941    1.10933   0.333        1
CL.Mix - CL.I == 0 -0.24288    1.10933  -0.219        1
(Adjusted p values reported -- bonferroni method)
```

ASV361

```
       Simultaneous Tests for General Linear Hypotheses

Multiple Comparisons of Means: Tukey Contrasts

Fit: lm(formula = log_cop ~ Treatment, data = dsub)

Linear Hypotheses:
                   Estimate Std. Error t value Pr(>|t|)
CL.F - CL.C == 0    2.708e-01  1.713e-01   1.581        1
CL.H - CL.C == 0    4.900e-17  1.713e-01   0.000        1
CL.I - CL.C == 0    4.611e-17  1.713e-01   0.000        1
CL.Mix - CL.C == 0  2.704e-16  1.713e-01   0.000        1
CL.H - CL.F == 0   -2.708e-01  1.713e-01  -1.581        1
CL.I - CL.F == 0   -2.708e-01  1.713e-01  -1.581        1
CL.Mix - CL.F == 0 -2.708e-01  1.713e-01  -1.581        1
CL.I - CL.H == 0   -2.889e-18  1.713e-01   0.000        1
CL.Mix - CL.H == 0  2.214e-16  1.713e-01   0.000        1
CL.Mix - CL.I == 0  2.243e-16  1.713e-01   0.000        1
(Adjusted p values reported -- bonferroni method)
```

ASV362

```
       Simultaneous Tests for General Linear Hypotheses

Multiple Comparisons of Means: Tukey Contrasts

Fit: lm(formula = log_cop ~ Treatment, data = dsub)

Linear Hypotheses:
                   Estimate Std. Error t value Pr(>|t|)
CL.F - CL.C == 0    1.066e-16  1.993e-01   0.000        1
CL.H - CL.C == 0    3.865e-16  1.993e-01   0.000        1
CL.I - CL.C == 0    1.020e-16  1.993e-01   0.000        1
```

```
CL.Mix - CL.C == 0  3.152e-01  1.993e-01   1.581        1
CL.H - CL.F == 0    2.799e-16  1.993e-01   0.000        1
CL.I - CL.F == 0   -4.633e-18  1.993e-01   0.000        1
CL.Mix - CL.F == 0  3.152e-01  1.993e-01   1.581        1
CL.I - CL.H == 0   -2.846e-16  1.993e-01   0.000        1
CL.Mix - CL.H == 0  3.152e-01  1.993e-01   1.581        1
CL.Mix - CL.I == 0  3.152e-01  1.993e-01   1.581        1
(Adjusted p values reported -- bonferroni method)
```

ASV365

```
       Simultaneous Tests for General Linear Hypotheses

Multiple Comparisons of Means: Tukey Contrasts

Fit: lm(formula = log_cop ~ Treatment, data = dsub)

Linear Hypotheses:
                     Estimate Std. Error t value Pr(>|t|)
CL.F - CL.C == 0    2.710e-01  1.714e-01   1.581        1
CL.H - CL.C == 0    2.950e-18  1.714e-01   0.000        1
CL.I - CL.C == 0    2.970e-18  1.714e-01   0.000        1
CL.Mix - CL.C == 0 -2.221e-16  1.714e-01   0.000        1
CL.H - CL.F == 0   -2.710e-01  1.714e-01  -1.581        1
CL.I - CL.F == 0   -2.710e-01  1.714e-01  -1.581        1
CL.Mix - CL.F == 0 -2.710e-01  1.714e-01  -1.581        1
CL.I - CL.H == 0    2.025e-20  1.714e-01   0.000        1
CL.Mix - CL.H == 0 -2.251e-16  1.714e-01   0.000        1
CL.Mix - CL.I == 0 -2.251e-16  1.714e-01   0.000        1
(Adjusted p values reported -- bonferroni method)
```

ASV37

Simultaneous Tests for General Linear Hypotheses

Multiple Comparisons of Means: Tukey Contrasts

Fit: lm(formula = log_cop ~ Treatment, data = dsub)

Linear Hypotheses:

	Estimate	Std. Error	t value	Pr(>|t|)
CL.F - CL.C == 0	-0.1690	0.9484	-0.178	1.000
CL.H - CL.C == 0	0.1520	0.9484	0.160	1.000
CL.I - CL.C == 0	1.8691	0.9484	1.971	0.524
CL.Mix - CL.C == 0	-0.3114	0.9484	-0.328	1.000
CL.H - CL.F == 0	0.3210	0.9484	0.338	1.000
CL.I - CL.F == 0	2.0381	0.9484	2.149	0.349
CL.Mix - CL.F == 0	-0.1424	0.9484	-0.150	1.000
CL.I - CL.H == 0	1.7172	0.9484	1.811	0.742
CL.Mix - CL.H == 0	-0.4634	0.9484	-0.489	1.000
CL.Mix - CL.I == 0	-2.1805	0.9484	-2.299	0.243

(Adjusted p values reported -- bonferroni method)

ASV373

Simultaneous Tests for General Linear Hypotheses

Multiple Comparisons of Means: Tukey Contrasts

Fit: lm(formula = log_cop ~ Treatment, data = dsub)

Linear Hypotheses:

	Estimate	Std. Error	t value	Pr(>|t|)
CL.F - CL.C == 0	-1.629e-16	1.625e-01	0.000	1
CL.H - CL.C == 0	2.569e-01	1.625e-01	1.581	1

```
CL.I - CL.C == 0   -5.558e-17  1.625e-01   0.000        1
CL.Mix - CL.C == 0 -5.899e-17  1.625e-01   0.000        1
CL.H - CL.F == 0    2.569e-01  1.625e-01   1.581        1
CL.I - CL.F == 0    1.073e-16  1.625e-01   0.000        1
CL.Mix - CL.F == 0  1.039e-16  1.625e-01   0.000        1
CL.I - CL.H == 0   -2.569e-01  1.625e-01  -1.581        1
CL.Mix - CL.H == 0 -2.569e-01  1.625e-01  -1.581        1
CL.Mix - CL.I == 0 -3.409e-18  1.625e-01   0.000        1
(Adjusted p values reported -- bonferroni method)
```

```
ASV375
       Simultaneous Tests for General Linear Hypotheses
Multiple Comparisons of Means: Tukey Contrasts
Fit: lm(formula = log_cop ~ Treatment, data = dsub)

Linear Hypotheses:
                  Estimate Std. Error t value Pr(>|t|)
CL.F - CL.C == 0    1.629e-16  1.603e-01   0.000        1
CL.H - CL.C == 0    2.534e-01  1.603e-01   1.581        1
CL.I - CL.C == 0    5.082e-17  1.603e-01   0.000        1
CL.Mix - CL.C == 0  4.433e-17  1.603e-01   0.000        1
CL.H - CL.F == 0    2.534e-01  1.603e-01   1.581        1
CL.I - CL.F == 0   -1.121e-16  1.603e-01   0.000        1
CL.Mix - CL.F == 0 -1.186e-16  1.603e-01   0.000        1
CL.I - CL.H == 0   -2.534e-01  1.603e-01  -1.581        1
CL.Mix - CL.H == 0 -2.534e-01  1.603e-01  -1.581        1
CL.Mix - CL.I == 0 -6.495e-18  1.603e-01   0.000        1
(Adjusted p values reported -- bonferroni method)
```

ASV377

```
	Simultaneous Tests for General Linear Hypotheses

Multiple Comparisons of Means: Tukey Contrasts

Fit: lm(formula = log_cop ~ Treatment, data = dsub)

Linear Hypotheses:
                  Estimate Std. Error t value Pr(>|t|)
CL.F - CL.C == 0   -2.669e-01  1.688e-01  -1.581        1
CL.H - CL.C == 0   -2.669e-01  1.688e-01  -1.581        1
CL.I - CL.C == 0   -2.669e-01  1.688e-01  -1.581        1
CL.Mix - CL.C == 0 -2.669e-01  1.688e-01  -1.581        1
CL.H - CL.F == 0   -2.220e-16  1.688e-01   0.000        1
CL.I - CL.F == 0   -2.220e-16  1.688e-01   0.000        1
CL.Mix - CL.F == 0 -2.776e-16  1.688e-01   0.000        1
CL.I - CL.H == 0    0.000e+00  1.688e-01   0.000        1
CL.Mix - CL.H == 0 -5.551e-17  1.688e-01   0.000        1
CL.Mix - CL.I == 0 -5.551e-17  1.688e-01   0.000        1
(Adjusted p values reported -- bonferroni method)
```

ASV379

```
	Simultaneous Tests for General Linear Hypotheses

Multiple Comparisons of Means: Tukey Contrasts

Fit: lm(formula = log_cop ~ Treatment, data = dsub)

Linear Hypotheses:
                 Estimate Std. Error t value Pr(>|t|)
CL.F - CL.C == 0   2.007e-16  1.638e-01   0.000        1
```

```
CL.H - CL.C == 0   2.235e-16  1.638e-01   0.000        1
CL.I - CL.C == 0   2.380e-16  1.638e-01   0.000        1
CL.Mix - CL.C == 0 2.590e-01  1.638e-01   1.581        1
CL.H - CL.F == 0   2.283e-17  1.638e-01   0.000        1
CL.I - CL.F == 0   3.724e-17  1.638e-01   0.000        1
CL.Mix - CL.F == 0 2.590e-01  1.638e-01   1.581        1
CL.I - CL.H == 0   1.441e-17  1.638e-01   0.000        1
CL.Mix - CL.H == 0 2.590e-01  1.638e-01   1.581        1
CL.Mix - CL.I == 0 2.590e-01  1.638e-01   1.581        1
(Adjusted p values reported -- bonferroni method)
```

ASV38

```
       Simultaneous Tests for General Linear Hypotheses

Multiple Comparisons of Means: Tukey Contrasts

Fit: lm(formula = log_cop ~ Treatment, data = dsub)

Linear Hypotheses:
                    Estimate Std. Error t value Pr(>|t|)
CL.F - CL.C == 0   -7.841e-01  3.422e-01  -2.291    0.248
CL.H - CL.C == 0   -7.841e-01  3.422e-01  -2.291    0.248
CL.I - CL.C == 0   -7.841e-01  3.422e-01  -2.291    0.248
CL.Mix - CL.C == 0 -7.841e-01  3.422e-01  -2.291    0.248
CL.H - CL.F == 0   -5.551e-16  3.422e-01   0.000    1.000
CL.I - CL.F == 0   -9.992e-16  3.422e-01   0.000    1.000
CL.Mix - CL.F == 0 -2.220e-16  3.422e-01   0.000    1.000
CL.I - CL.H == 0   -4.441e-16  3.422e-01   0.000    1.000
CL.Mix - CL.H == 0  3.331e-16  3.422e-01   0.000    1.000
CL.Mix - CL.I == 0  7.772e-16  3.422e-01   0.000    1.000
```

(Adjusted p values reported -- bonferroni method)

ASV39

```
	 Simultaneous Tests for General Linear Hypotheses

Multiple Comparisons of Means: Tukey Contrasts

Fit: lm(formula = log_cop ~ Treatment, data = dsub)

Linear Hypotheses:
                  Estimate Std. Error t value Pr(>|t|)
CL.F - CL.C == 0    3.666e-01  5.601e-01   0.654   1.0000
CL.H - CL.C == 0    3.609e-01  5.601e-01   0.644   1.0000
CL.I - CL.C == 0    6.799e-16  5.601e-01   0.000   1.0000
CL.Mix - CL.C == 0  1.611e+00  5.601e-01   2.876   0.0524 .
CL.H - CL.F == 0   -5.666e-03  5.601e-01  -0.010   1.0000
CL.I - CL.F == 0   -3.666e-01  5.601e-01  -0.654   1.0000
CL.Mix - CL.F == 0  1.244e+00  5.601e-01   2.222   0.2932
CL.I - CL.H == 0   -3.609e-01  5.601e-01  -0.644   1.0000
CL.Mix - CL.H == 0  1.250e+00  5.601e-01   2.232   0.2861
CL.Mix - CL.I == 0  1.611e+00  5.601e-01   2.876   0.0524 .
---
Signif. codes:  0 ‘***’ 0.001 ‘**’ 0.01 ‘*’ 0.05 ‘.’ 0.1 ‘ ’ 1
(Adjusted p values reported -- bonferroni method)
```

ASV390

```
	 Simultaneous Tests for General Linear Hypotheses

Multiple Comparisons of Means: Tukey Contrasts

Fit: lm(formula = log_cop ~ Treatment, data = dsub)
```

```
Linear Hypotheses:
                    Estimate Std. Error t value Pr(>|t|)
CL.F - CL.C == 0    3.189e-01  2.017e-01   1.581        1
CL.H - CL.C == 0    4.750e-17  2.017e-01   0.000        1
CL.I - CL.C == 0    3.811e-17  2.017e-01   0.000        1
CL.Mix - CL.C == 0  7.076e-17  2.017e-01   0.000        1
CL.H - CL.F == 0   -3.189e-01  2.017e-01  -1.581        1
CL.I - CL.F == 0   -3.189e-01  2.017e-01  -1.581        1
CL.Mix - CL.F == 0 -3.189e-01  2.017e-01  -1.581        1
CL.I - CL.H == 0   -9.388e-18  2.017e-01   0.000        1
CL.Mix - CL.H == 0  2.326e-17  2.017e-01   0.000        1
CL.Mix - CL.I == 0  3.264e-17  2.017e-01   0.000        1
(Adjusted p values reported -- bonferroni method)
```

ASV391

```
	 Simultaneous Tests for General Linear Hypotheses

Multiple Comparisons of Means: Tukey Contrasts

Fit: lm(formula = log_cop ~ Treatment, data = dsub)

Linear Hypotheses:
                    Estimate Std. Error t value Pr(>|t|)
CL.F - CL.C == 0    4.655e-17  1.886e-01   0.000        1
CL.H - CL.C == 0    2.982e-01  1.886e-01   1.581        1
CL.I - CL.C == 0    8.463e-18  1.886e-01   0.000        1
CL.Mix - CL.C == 0 -1.072e-17  1.886e-01   0.000        1
CL.H - CL.F == 0    2.982e-01  1.886e-01   1.581        1
CL.I - CL.F == 0   -3.809e-17  1.886e-01   0.000        1
CL.Mix - CL.F == 0 -5.727e-17  1.886e-01   0.000        1
```

```
CL.I - CL.H == 0   -2.982e-01  1.886e-01  -1.581        1
CL.Mix - CL.H == 0 -2.982e-01  1.886e-01  -1.581        1
CL.Mix - CL.I == 0 -1.918e-17  1.886e-01   0.000        1
(Adjusted p values reported -- bonferroni method)
```

```
ASV392
	 Simultaneous Tests for General Linear Hypotheses
Multiple Comparisons of Means: Tukey Contrasts
Fit: lm(formula = log_cop ~ Treatment, data = dsub)

Linear Hypotheses:
                  Estimate Std. Error t value Pr(>|t|)
CL.F - CL.C == 0    4.655e-17  1.886e-01   0.000        1
CL.H - CL.C == 0    2.982e-01  1.886e-01   1.581        1
CL.I - CL.C == 0    8.463e-18  1.886e-01   0.000        1
CL.Mix - CL.C == 0 -1.072e-17  1.886e-01   0.000        1
CL.H - CL.F == 0    2.982e-01  1.886e-01   1.581        1
CL.I - CL.F == 0   -3.809e-17  1.886e-01   0.000        1
CL.Mix - CL.F == 0 -5.727e-17  1.886e-01   0.000        1
CL.I - CL.H == 0   -2.982e-01  1.886e-01  -1.581        1
CL.Mix - CL.H == 0 -2.982e-01  1.886e-01  -1.581        1
CL.Mix - CL.I == 0 -1.918e-17  1.886e-01   0.000        1
(Adjusted p values reported -- bonferroni method)
```

```
ASV396
	 Simultaneous Tests for General Linear Hypotheses
Multiple Comparisons of Means: Tukey Contrasts
```

```
Fit: lm(formula = log_cop ~ Treatment, data = dsub)

Linear Hypotheses:
                    Estimate Std. Error t value Pr(>|t|)
CL.F - CL.C == 0   9.621e-17  1.895e-01   0.000        1
CL.H - CL.C == 0   1.528e-16  1.895e-01   0.000        1
CL.I - CL.C == 0   1.700e-16  1.895e-01   0.000        1
CL.Mix - CL.C == 0 2.996e-01  1.895e-01   1.581        1
CL.H - CL.F == 0   5.660e-17  1.895e-01   0.000        1
CL.I - CL.F == 0   7.376e-17  1.895e-01   0.000        1
CL.Mix - CL.F == 0 2.996e-01  1.895e-01   1.581        1
CL.I - CL.H == 0   1.716e-17  1.895e-01   0.000        1
CL.Mix - CL.H == 0 2.996e-01  1.895e-01   1.581        1
CL.Mix - CL.I == 0 2.996e-01  1.895e-01   1.581        1
(Adjusted p values reported -- bonferroni method)
```

ASV4

```
         Simultaneous Tests for General Linear Hypotheses

Multiple Comparisons of Means: Tukey Contrasts

Fit: lm(formula = log_cop ~ Treatment, data = dsub)

Linear Hypotheses:
                   Estimate Std. Error t value Pr(>|t|)
CL.F - CL.C == 0     2.4297     0.9823   2.474   0.1564
CL.H - CL.C == 0     2.7314     0.9823   2.781   0.0685 .
CL.I - CL.C == 0     1.8645     0.9823   1.898   0.6152
CL.Mix - CL.C == 0   0.5560     0.9823   0.566   1.0000
CL.H - CL.F == 0     0.3017     0.9823   0.307   1.0000
CL.I - CL.F == 0    -0.5652     0.9823  -0.575   1.0000
```

```
CL.Mix - CL.F == 0  -1.8737     0.9823  -1.908   0.6028
CL.I - CL.H == 0    -0.8669     0.9823  -0.883   1.0000
CL.Mix - CL.H == 0  -2.1754     0.9823  -2.215   0.2982
CL.Mix - CL.I == 0  -1.3085     0.9823  -1.332   1.0000
---
Signif. codes:  0 ‘***’ 0.001 ‘**’ 0.01 ‘*’ 0.05 ‘.’ 0.1 ‘ ’ 1
(Adjusted p values reported -- bonferroni method)
```

ASV40

```
       Simultaneous Tests for General Linear Hypotheses
Multiple Comparisons of Means: Tukey Contrasts
Fit: lm(formula = log_cop ~ Treatment, data = dsub)

Linear Hypotheses:
               Estimate Std. Error t value Pr(>|t|)
CL.F - CL.C == 0    1.23156    1.03787   1.187   1.0000
CL.H - CL.C == 0    2.78427    1.03787   2.683   0.0898 .
CL.I - CL.C == 0    1.86064    1.03787   1.793   0.7704
CL.Mix - CL.C == 0 -0.01102    1.03787  -0.011   1.0000
CL.H - CL.F == 0    1.55271    1.03787   1.496   1.0000
CL.I - CL.F == 0    0.62908    1.03787   0.606   1.0000
CL.Mix - CL.F == 0 -1.24258    1.03787  -1.197   1.0000
CL.I - CL.H == 0   -0.92362    1.03787  -0.890   1.0000
CL.Mix - CL.H == 0 -2.79528    1.03787  -2.693   0.0872 .
CL.Mix - CL.I == 0 -1.87166    1.03787  -1.803   0.7535
---
Signif. codes:  0 ‘***’ 0.001 ‘**’ 0.01 ‘*’ 0.05 ‘.’ 0.1 ‘ ’ 1
(Adjusted p values reported -- bonferroni method)
```

ASV41

```
	 Simultaneous Tests for General Linear Hypotheses

Multiple Comparisons of Means: Tukey Contrasts

Fit: lm(formula = log_cop ~ Treatment, data = dsub)

Linear Hypotheses:
                   Estimate Std. Error t value Pr(>|t|)
CL.F - CL.C == 0    2.666e-01  1.686e-01   1.581        1
CL.H - CL.C == 0   -1.475e-18  1.686e-01   0.000        1
CL.I - CL.C == 0   -3.159e-18  1.686e-01   0.000        1
CL.Mix - CL.C == 0 -1.359e-16  1.686e-01   0.000        1
CL.H - CL.F == 0   -2.666e-01  1.686e-01  -1.581        1
CL.I - CL.F == 0   -2.666e-01  1.686e-01  -1.581        1
CL.Mix - CL.F == 0 -2.666e-01  1.686e-01  -1.581        1
CL.I - CL.H == 0   -1.685e-18  1.686e-01   0.000        1
CL.Mix - CL.H == 0 -1.345e-16  1.686e-01   0.000        1
CL.Mix - CL.I == 0 -1.328e-16  1.686e-01   0.000        1
(Adjusted p values reported -- bonferroni method)
```

ASV42

```
	 Simultaneous Tests for General Linear Hypotheses

Multiple Comparisons of Means: Tukey Contrasts

Fit: lm(formula = log_cop ~ Treatment, data = dsub)

Linear Hypotheses:
                   Estimate Std. Error t value Pr(>|t|)
CL.F - CL.C == 0    2.281e+00  4.893e-01   4.662 0.000133 ***
```

```
CL.H - CL.C == 0    6.097e-17  4.893e-01   0.000 1.000000
CL.I - CL.C == 0    7.359e-17  4.893e-01   0.000 1.000000
CL.Mix - CL.C == 0 -9.605e-17  4.893e-01   0.000 1.000000
CL.H - CL.F == 0   -2.281e+00  4.893e-01  -4.662 0.000133 ***
CL.I - CL.F == 0   -2.281e+00  4.893e-01  -4.662 0.000133 ***
CL.Mix - CL.F == 0 -2.281e+00  4.893e-01  -4.662 0.000133 ***
CL.I - CL.H == 0    1.262e-17  4.893e-01   0.000 1.000000
CL.Mix - CL.H == 0 -1.570e-16  4.893e-01   0.000 1.000000
CL.Mix - CL.I == 0 -1.696e-16  4.893e-01   0.000 1.000000
---
Signif. codes:  0 ‘***’ 0.001 ‘**’ 0.01 ‘*’ 0.05 ‘.’ 0.1 ‘ ’ 1
(Adjusted p values reported -- bonferroni method)
```

ASV44

```
	 Simultaneous Tests for General Linear Hypotheses
Multiple Comparisons of Means: Tukey Contrasts
Fit: lm(formula = log_cop ~ Treatment, data = dsub)

Linear Hypotheses:
                  Estimate Std. Error t value Pr(>|t|)
CL.F - CL.C == 0   -3.575e-01  3.991e-01  -0.896        1
CL.H - CL.C == 0    1.184e-02  3.991e-01   0.030        1
CL.I - CL.C == 0   -3.575e-01  3.991e-01  -0.896        1
CL.Mix - CL.C == 0  8.503e-03  3.991e-01   0.021        1
CL.H - CL.F == 0    3.693e-01  3.991e-01   0.925        1
CL.I - CL.F == 0   -7.772e-16  3.991e-01   0.000        1
CL.Mix - CL.F == 0  3.660e-01  3.991e-01   0.917        1
CL.I - CL.H == 0   -3.693e-01  3.991e-01  -0.925        1
```

```
CL.Mix - CL.H == 0 -3.341e-03  3.991e-01  -0.008        1
CL.Mix - CL.I == 0  3.660e-01  3.991e-01   0.917        1
(Adjusted p values reported -- bonferroni method)
```

ASV45

```
	 Simultaneous Tests for General Linear Hypotheses

Multiple Comparisons of Means: Tukey Contrasts

Fit: lm(formula = log_cop ~ Treatment, data = dsub)

Linear Hypotheses:
                    Estimate Std. Error t value Pr(>|t|)
CL.F - CL.C == 0    0.035837   0.724088   0.049        1
CL.H - CL.C == 0   -0.248471   0.724088  -0.343        1
CL.I - CL.C == 0   -1.047345   0.724088  -1.446        1
CL.Mix - CL.C == 0  0.038791   0.724088   0.054        1
CL.H - CL.F == 0   -0.284309   0.724088  -0.393        1
CL.I - CL.F == 0   -1.083182   0.724088  -1.496        1
CL.Mix - CL.F == 0  0.002954   0.724088   0.004        1
CL.I - CL.H == 0   -0.798873   0.724088  -1.103        1
CL.Mix - CL.H == 0  0.287262   0.724088   0.397        1
CL.Mix - CL.I == 0  1.086136   0.724088   1.500        1
(Adjusted p values reported -- bonferroni method)
```

ASV46

```
	 Simultaneous Tests for General Linear Hypotheses

Multiple Comparisons of Means: Tukey Contrasts

Fit: lm(formula = log_cop ~ Treatment, data = dsub)
```

```
Linear Hypotheses:
                   Estimate Std. Error t value Pr(>|t|)
CL.F - CL.C == 0   -2.882e-16  5.371e-01   0.000    1.000
CL.H - CL.C == 0    8.333e-16  5.371e-01   0.000    1.000
CL.I - CL.C == 0    1.087e+00  5.371e-01   2.024    0.465
CL.Mix - CL.C == 0  1.142e+00  5.371e-01   2.126    0.368
CL.H - CL.F == 0    1.121e-15  5.371e-01   0.000    1.000
CL.I - CL.F == 0    1.087e+00  5.371e-01   2.024    0.465
CL.Mix - CL.F == 0  1.142e+00  5.371e-01   2.126    0.368
CL.I - CL.H == 0    1.087e+00  5.371e-01   2.024    0.465
CL.Mix - CL.H == 0  1.142e+00  5.371e-01   2.126    0.368
CL.Mix - CL.I == 0  5.489e-02  5.371e-01   0.102    1.000
(Adjusted p values reported -- bonferroni method)
```

ASV48

```
       Simultaneous Tests for General Linear Hypotheses
Multiple Comparisons of Means: Tukey Contrasts
Fit: lm(formula = log_cop ~ Treatment, data = dsub)

Linear Hypotheses:
                   Estimate Std. Error t value Pr(>|t|)
CL.F - CL.C == 0    1.940e-16  1.759e-01   0.000        1
CL.H - CL.C == 0    0.000e+00  1.759e-01   0.000        1
CL.I - CL.C == 0    2.781e-01  1.759e-01   1.581        1
CL.Mix - CL.C == 0 -3.925e-17  1.759e-01   0.000        1
CL.H - CL.F == 0   -1.940e-16  1.759e-01   0.000        1
CL.I - CL.F == 0    2.781e-01  1.759e-01   1.581        1
CL.Mix - CL.F == 0 -2.332e-16  1.759e-01   0.000        1
```

```
CL.I - CL.H == 0    2.781e-01  1.759e-01   1.581        1
CL.Mix - CL.H == 0 -3.925e-17  1.759e-01   0.000        1
CL.Mix - CL.I == 0 -2.781e-01  1.759e-01  -1.581        1
(Adjusted p values reported -- bonferroni method)
```

ASV49

```
       Simultaneous Tests for General Linear Hypotheses
Multiple Comparisons of Means: Tukey Contrasts
Fit: lm(formula = log_cop ~ Treatment, data = dsub)

Linear Hypotheses:
               Estimate Std. Error t value Pr(>|t|)
CL.F - CL.C == 0    0.24814    0.36815   0.674        1
CL.H - CL.C == 0    0.26158    0.36815   0.711        1
CL.I - CL.C == 0    0.54983    0.36815   1.493        1
CL.Mix - CL.C == 0  0.53030    0.36815   1.440        1
CL.H - CL.F == 0    0.01344    0.36815   0.036        1
CL.I - CL.F == 0    0.30169    0.36815   0.819        1
CL.Mix - CL.F == 0  0.28216    0.36815   0.766        1
CL.I - CL.H == 0    0.28825    0.36815   0.783        1
CL.Mix - CL.H == 0  0.26872    0.36815   0.730        1
CL.Mix - CL.I == 0 -0.01953    0.36815  -0.053        1
(Adjusted p values reported -- bonferroni method)
```

ASV5

```
       Simultaneous Tests for General Linear Hypotheses
Multiple Comparisons of Means: Tukey Contrasts
```

```
Fit: lm(formula = log_cop ~ Treatment, data = dsub)

Linear Hypotheses:
                     Estimate Std. Error t value Pr(>|t|)
CL.F - CL.C == 0      0.25180    0.22195   1.134    1.000
CL.H - CL.C == 0      0.36551    0.22195   1.647    1.000
CL.I - CL.C == 0      0.20459    0.22195   0.922    1.000
CL.Mix - CL.C == 0  0.45122    0.22195   2.033    0.456
CL.H - CL.F == 0      0.11371    0.22195   0.512    1.000
CL.I - CL.F == 0     -0.04721    0.22195  -0.213    1.000
CL.Mix - CL.F == 0  0.19942    0.22195   0.899    1.000
CL.I - CL.H == 0     -0.16092    0.22195  -0.725    1.000
CL.Mix - CL.H == 0  0.08571    0.22195   0.386    1.000
CL.Mix - CL.I == 0  0.24663    0.22195   1.111    1.000
(Adjusted p values reported -- bonferroni method)
```

ASV50

```
       Simultaneous Tests for General Linear Hypotheses

Multiple Comparisons of Means: Tukey Contrasts

Fit: lm(formula = log_cop ~ Treatment, data = dsub)

Linear Hypotheses:
                     Estimate Std. Error t value Pr(>|t|)
CL.F - CL.C == 0     -4.223e-01  2.671e-01  -1.581        1
CL.H - CL.C == 0     -4.223e-01  2.671e-01  -1.581        1
CL.I - CL.C == 0     -4.223e-01  2.671e-01  -1.581        1
CL.Mix - CL.C == 0 -4.223e-01  2.671e-01  -1.581        1
CL.H - CL.F == 0     -6.106e-16  2.671e-01   0.000        1
CL.I - CL.F == 0      1.110e-16  2.671e-01   0.000        1
```

```
CL.Mix - CL.F == 0 -3.886e-16  2.671e-01   0.000        1
CL.I - CL.H == 0    7.216e-16  2.671e-01   0.000        1
CL.Mix - CL.H == 0  2.220e-16  2.671e-01   0.000        1
CL.Mix - CL.I == 0 -4.996e-16  2.671e-01   0.000        1
(Adjusted p values reported -- bonferroni method)
```

ASV51

```
	 Simultaneous Tests for General Linear Hypotheses

Multiple Comparisons of Means: Tukey Contrasts

Fit: lm(formula = log_cop ~ Treatment, data = dsub)

Linear Hypotheses:
                   Estimate Std. Error t value Pr(>|t|)
CL.F - CL.C == 0   -1.45935    0.98019  -1.489        1
CL.H - CL.C == 0    0.05261    0.98019   0.054        1
CL.I - CL.C == 0   -0.30751    0.98019  -0.314        1
CL.Mix - CL.C == 0 -0.70534    0.98019  -0.720        1
CL.H - CL.F == 0    1.51195    0.98019   1.543        1
CL.I - CL.F == 0    1.15184    0.98019   1.175        1
CL.Mix - CL.F == 0  0.75400    0.98019   0.769        1
CL.I - CL.H == 0   -0.36012    0.98019  -0.367        1
CL.Mix - CL.H == 0 -0.75795    0.98019  -0.773        1
CL.Mix - CL.I == 0 -0.39783    0.98019  -0.406        1
(Adjusted p values reported -- bonferroni method)
```

ASV53

```
	 Simultaneous Tests for General Linear Hypotheses
```

```
Multiple Comparisons of Means: Tukey Contrasts

Fit: lm(formula = log_cop ~ Treatment, data = dsub)

Linear Hypotheses:
                  Estimate Std. Error t value Pr(>|t|)
CL.F - CL.C == 0   -2.230e+00  5.230e-01  -4.264 0.000578 ***
CL.H - CL.C == 0   -1.876e+00  5.230e-01  -3.586 0.005948 **
CL.I - CL.C == 0   -2.230e+00  5.230e-01  -4.264 0.000578 ***
CL.Mix - CL.C == 0 -2.230e+00  5.230e-01  -4.264 0.000578 ***
CL.H - CL.F == 0    3.545e-01  5.230e-01   0.678 1.000000
CL.I - CL.F == 0   -2.665e-15  5.230e-01   0.000 1.000000
CL.Mix - CL.F == 0  0.000e+00  5.230e-01   0.000 1.000000
CL.I - CL.H == 0   -3.545e-01  5.230e-01  -0.678 1.000000
CL.Mix - CL.H == 0 -3.545e-01  5.230e-01  -0.678 1.000000
CL.Mix - CL.I == 0  2.665e-15  5.230e-01   0.000 1.000000
---
Signif. codes:  0 ‘***’ 0.001 ‘**’ 0.01 ‘*’ 0.05 ‘.’ 0.1 ‘ ’ 1
(Adjusted p values reported -- bonferroni method)
```

ASV55

```
       Simultaneous Tests for General Linear Hypotheses

Multiple Comparisons of Means: Tukey Contrasts

Fit: lm(formula = log_cop ~ Treatment, data = dsub)

Linear Hypotheses:
                Estimate Std. Error t value Pr(>|t|)
CL.F - CL.C == 0    0.45673    0.63459   0.720   1.0000
CL.H - CL.C == 0    0.02236    0.63459   0.035   1.0000
CL.I - CL.C == 0    1.51273    0.63459   2.384   0.1967
```

```
CL.Mix - CL.C == 0 -0.30847    0.63459  -0.486   1.0000
CL.H - CL.F == 0   -0.43437    0.63459  -0.684   1.0000
CL.I - CL.F == 0    1.05600    0.63459   1.664   1.0000
CL.Mix - CL.F == 0 -0.76520    0.63459  -1.206   1.0000
CL.I - CL.H == 0    1.49037    0.63459   2.349   0.2148
CL.Mix - CL.H == 0 -0.33083    0.63459  -0.521   1.0000
CL.Mix - CL.I == 0 -1.82120    0.63459  -2.870   0.0533 .
---
Signif. codes:  0 ‘***’ 0.001 ‘**’ 0.01 ‘*’ 0.05 ‘.’ 0.1 ‘ ’ 1
(Adjusted p values reported -- bonferroni method)
```

ASV57

```
       Simultaneous Tests for General Linear Hypotheses
Multiple Comparisons of Means: Tukey Contrasts
Fit: lm(formula = log_cop ~ Treatment, data = dsub)

Linear Hypotheses:
               Estimate Std. Error t value Pr(>|t|)
CL.F - CL.C == 0    0.37160    1.00634   0.369    1.000
CL.H - CL.C == 0   -0.04087    1.00634  -0.041    1.000
CL.I - CL.C == 0    1.18261    1.00634   1.175    1.000
CL.Mix - CL.C == 0 -0.80241    1.00634  -0.797    1.000
CL.H - CL.F == 0   -0.41248    1.00634  -0.410    1.000
CL.I - CL.F == 0    0.81100    1.00634   0.806    1.000
CL.Mix - CL.F == 0 -1.17402    1.00634  -1.167    1.000
CL.I - CL.H == 0    1.22348    1.00634   1.216    1.000
CL.Mix - CL.H == 0 -0.76154    1.00634  -0.757    1.000
CL.Mix - CL.I == 0 -1.98502    1.00634  -1.973    0.522
```

```
(Adjusted p values reported -- bonferroni method)
```

ASV59

```
	Simultaneous Tests for General Linear Hypotheses

Multiple Comparisons of Means: Tukey Contrasts

Fit: lm(formula = log_cop ~ Treatment, data = dsub)

Linear Hypotheses:
               Estimate Std. Error t value Pr(>|t|)
CL.F - CL.C == 0   -2.09746    0.79492  -2.639  0.10120
CL.H - CL.C == 0    0.75481    0.79492   0.950  1.00000
CL.I - CL.C == 0   -1.11213    0.79492  -1.399  1.00000
CL.Mix - CL.C == 0  0.06082    0.79492   0.077  1.00000
CL.H - CL.F == 0    2.85227    0.79492   3.588  0.00591 **
CL.I - CL.F == 0    0.98533    0.79492   1.240  1.00000
CL.Mix - CL.F == 0  2.15828    0.79492   2.715  0.08219 .
CL.I - CL.H == 0   -1.86693    0.79492  -2.349  0.21483
CL.Mix - CL.H == 0 -0.69398    0.79492  -0.873  1.00000
CL.Mix - CL.I == 0  1.17295    0.79492   1.476  1.00000
---
Signif. codes:  0 ‘***’ 0.001 ‘**’ 0.01 ‘*’ 0.05 ‘.’ 0.1 ‘ ’ 1
(Adjusted p values reported -- bonferroni method)
```

ASV6

```
	Simultaneous Tests for General Linear Hypotheses

Multiple Comparisons of Means: Tukey Contrasts

Fit: lm(formula = log_cop ~ Treatment, data = dsub)
```

Linear Hypotheses:

	Estimate	Std. Error	t value	Pr(>|t|)
CL.F - CL.C == 0	0.8783	0.8610	1.020	1.000
CL.H - CL.C == 0	1.8844	0.8610	2.189	0.317
CL.I - CL.C == 0	1.1092	0.8610	1.288	1.000
CL.Mix - CL.C == 0	0.4175	0.8610	0.485	1.000
CL.H - CL.F == 0	1.0060	0.8610	1.168	1.000
CL.I - CL.F == 0	0.2308	0.8610	0.268	1.000
CL.Mix - CL.F == 0	-0.4608	0.8610	-0.535	1.000
CL.I - CL.H == 0	-0.7752	0.8610	-0.900	1.000
CL.Mix - CL.H == 0	-1.4668	0.8610	-1.704	0.926
CL.Mix - CL.I == 0	-0.6916	0.8610	-0.803	1.000

(Adjusted p values reported -- bonferroni method)

ASV60

Simultaneous Tests for General Linear Hypotheses

Multiple Comparisons of Means: Tukey Contrasts

Ft: lm(formula = log_cop ~ Treatment, data = dsub)

Linear Hypotheses:

	Estimate	Std. Error	t value	Pr(>|t|)
CL.F - CL.C == 0	3.937e-01	2.490e-01	1.581	1
CL.H - CL.C == 0	1.215e-17	2.490e-01	0.000	1
CL.I - CL.C == 0	8.529e-18	2.490e-01	0.000	1
CL.Mix - CL.C == 0	1.174e-16	2.490e-01	0.000	1
CL.H - CL.F == 0	-3.937e-01	2.490e-01	-1.581	1
CL.I - CL.F == 0	-3.937e-01	2.490e-01	-1.581	1
CL.Mix - CL.F == 0	-3.937e-01	2.490e-01	-1.581	1

```
CL.I - CL.H == 0   -3.617e-18  2.490e-01   0.000        1
CL.Mix - CL.H == 0  1.052e-16  2.490e-01   0.000        1
CL.Mix - CL.I == 0  1.088e-16  2.490e-01   0.000        1
(Adjusted p values reported -- bonferroni method)
```

ASV61

```
       Simultaneous Tests for General Linear Hypotheses
Multiple Comparisons of Means: Tukey Contrasts
Fit: lm(formula = log_cop ~ Treatment, data = dsub)

Linear Hypotheses:
               Estimate Std. Error t value Pr(>|t|)
CL.F - CL.C == 0   -1.03610    0.53862  -1.924    0.582
CL.H - CL.C == 0   -0.69063    0.53862  -1.282    1.000
CL.I - CL.C == 0   -0.70663    0.53862  -1.312    1.000
CL.Mix - CL.C == 0 -0.61071    0.53862  -1.134    1.000
CL.H - CL.F == 0    0.34546    0.53862   0.641    1.000
CL.I - CL.F == 0    0.32947    0.53862   0.612    1.000
CL.Mix - CL.F == 0  0.42538    0.53862   0.790    1.000
CL.I - CL.H == 0   -0.01600    0.53862  -0.030    1.000
CL.Mix - CL.H == 0  0.07992    0.53862   0.148    1.000
CL.Mix - CL.I == 0  0.09592    0.53862   0.178    1.000
(Adjusted p values reported -- bonferroni method)
```

ASV62

```
       Simultaneous Tests for General Linear Hypotheses
Multiple Comparisons of Means: Tukey Contrasts
```

Fit: lm(formula = log_cop ~ Treatment, data = dsub)

Linear Hypotheses:

	Estimate	Std. Error	t value	Pr(>|t|)
CL.F - CL.C == 0	-4.613e-01	2.917e-01	-1.581	1
CL.H - CL.C == 0	-4.613e-01	2.917e-01	-1.581	1
CL.I - CL.C == 0	-4.613e-01	2.917e-01	-1.581	1
CL.Mix - CL.C == 0	-4.613e-01	2.917e-01	-1.581	1
CL.H - CL.F == 0	-3.886e-16	2.917e-01	0.000	1
CL.I - CL.F == 0	-6.106e-16	2.917e-01	0.000	1
CL.Mix - CL.F == 0	-2.220e-16	2.917e-01	0.000	1
CL.I - CL.H == 0	-2.220e-16	2.917e-01	0.000	1
CL.Mix - CL.H == 0	1.665e-16	2.917e-01	0.000	1
CL.Mix - CL.I == 0	3.886e-16	2.917e-01	0.000	1

(Adjusted p values reported -- bonferroni method)

ASV64

Simultaneous Tests for General Linear Hypotheses

Multiple Comparisons of Means: Tukey Contrasts

Fit: lm(formula = log_cop ~ Treatment, data = dsub)

Linear Hypotheses:

	Estimate	Std. Error	t value	Pr(>|t|)
CL.F - CL.C == 0	0.07979	0.95321	0.084	1.000
CL.H - CL.C == 0	1.63618	0.95321	1.716	0.902
CL.I - CL.C == 0	1.89437	0.95321	1.987	0.505
CL.Mix - CL.C == 0	1.53356	0.95321	1.609	1.000
CL.H - CL.F == 0	1.55639	0.95321	1.633	1.000
CL.I - CL.F == 0	1.81458	0.95321	1.904	0.608

```
CL.Mix - CL.F == 0  1.45378    0.95321   1.525    1.000

CL.I - CL.H == 0    0.25819    0.95321   0.271    1.000

CL.Mix - CL.H == 0 -0.10261    0.95321  -0.108    1.000

CL.Mix - CL.I == 0 -0.36080    0.95321  -0.379    1.000

(Adjusted p values reported -- bonferroni method)



ASV66

       Simultaneous Tests for General Linear Hypotheses

Multiple Comparisons of Means: Tukey Contrasts

Fit: lm(formula = log_cop ~ Treatment, data = dsub)


Linear Hypotheses:

                  Estimate Std. Error t value Pr(>|t|)

CL.F - CL.C == 0   -1.579e+00  4.479e-01  -3.525  0.00725 **

CL.H - CL.C == 0   -1.579e+00  4.479e-01  -3.525  0.00725 **

CL.I - CL.C == 0   -1.579e+00  4.479e-01  -3.525  0.00725 **

CL.Mix - CL.C == 0 -1.579e+00  4.479e-01  -3.525  0.00725 **

CL.H - CL.F == 0   -1.110e-15  4.479e-01   0.000  1.00000

CL.I - CL.F == 0   -1.776e-15  4.479e-01   0.000  1.00000

CL.Mix - CL.F == 0  8.882e-16  4.479e-01   0.000  1.00000

CL.I - CL.H == 0   -6.661e-16  4.479e-01   0.000  1.00000

CL.Mix - CL.H == 0  1.998e-15  4.479e-01   0.000  1.00000

CL.Mix - CL.I == 0  2.664e-15  4.479e-01   0.000  1.00000

---

Signif. codes:  0 ‘***’ 0.001 ‘**’ 0.01 ‘*’ 0.05 ‘.’ 0.1 ‘ ’ 1

(Adjusted p values reported -- bonferroni method)
```

ASV68

```
      Simultaneous Tests for General Linear Hypotheses

Multiple Comparisons of Means: Tukey Contrasts

Fit: lm(formula = log_cop ~ Treatment, data = dsub)

Linear Hypotheses:
                  Estimate Std. Error t value Pr(>|t|)
CL.F - CL.C == 0    3.698e-01  3.776e-01   0.979        1
CL.H - CL.C == 0    1.282e-16  3.776e-01   0.000        1
CL.I - CL.C == 0    4.688e-01  3.776e-01   1.241        1
CL.Mix - CL.C == 0  1.178e-16  3.776e-01   0.000        1
CL.H - CL.F == 0   -3.698e-01  3.776e-01  -0.979        1
CL.I - CL.F == 0    9.908e-02  3.776e-01   0.262        1
CL.Mix - CL.F == 0 -3.698e-01  3.776e-01  -0.979        1
CL.I - CL.H == 0    4.688e-01  3.776e-01   1.241        1
CL.Mix - CL.H == 0 -1.044e-17  3.776e-01   0.000        1
CL.Mix - CL.I == 0 -4.688e-01  3.776e-01  -1.241        1
(Adjusted p values reported -- bonferroni method)
```

ASV69

```
      Simultaneous Tests for General Linear Hypotheses

Multiple Comparisons of Means: Tukey Contrasts

Fit: lm(formula = log_cop ~ Treatment, data = dsub)

Linear Hypotheses:
                 Estimate Std. Error t value Pr(>|t|)
CL.F - CL.C == 0   -0.04070    0.64032  -0.064    1.000
CL.H - CL.C == 0    1.19277    0.64032   1.863    0.664
CL.I - CL.C == 0   -0.44399    0.64032  -0.693    1.000
```

```
CL.Mix - CL.C == 0 -0.08517    0.64032  -0.133    1.000
CL.H - CL.F == 0    1.23346    0.64032   1.926    0.579
CL.I - CL.F == 0   -0.40329    0.64032  -0.630    1.000
CL.Mix - CL.F == 0 -0.04447    0.64032  -0.069    1.000
CL.I - CL.H == 0   -1.63676    0.64032  -2.556    0.126
CL.Mix - CL.H == 0 -1.27794    0.64032  -1.996    0.496
CL.Mix - CL.I == 0  0.35882    0.64032   0.560    1.000
(Adjusted p values reported -- bonferroni method)
```

ASV7

```
       Simultaneous Tests for General Linear Hypotheses

Multiple Comparisons of Means: Tukey Contrasts

Fit: lm(formula = log_cop ~ Treatment, data = dsub)

Linear Hypotheses:
                Estimate Std. Error t value Pr(>|t|)
CL.F - CL.C == 0    0.156159   0.818590   0.191        1
CL.H - CL.C == 0   -0.022009   0.818590  -0.027        1
CL.I - CL.C == 0    0.651596   0.818590   0.796        1
CL.Mix - CL.C == 0 -0.006299   0.818590  -0.008        1
CL.H - CL.F == 0   -0.178168   0.818590  -0.218        1
CL.I - CL.F == 0    0.495437   0.818590   0.605        1
CL.Mix - CL.F == 0 -0.162458   0.818590  -0.198        1
CL.I - CL.H == 0    0.673604   0.818590   0.823        1
CL.Mix - CL.H == 0  0.015709   0.818590   0.019        1
CL.Mix - CL.I == 0 -0.657895   0.818590  -0.804        1
(Adjusted p values reported -- bonferroni method)
```

ASV70

Simultaneous Tests for General Linear Hypotheses

Multiple Comparisons of Means: Tukey Contrasts

Fit: lm(formula = log_cop ~ Treatment, data = dsub)

Linear Hypotheses:

	Estimate	Std. Error	t value	Pr(>|t|)
CL.F - CL.C == 0	4.398e-01	3.603e-01	1.221	1
CL.H - CL.C == 0	4.532e-17	3.603e-01	0.000	1
CL.I - CL.C == 0	1.360e-16	3.603e-01	0.000	1
CL.Mix - CL.C == 0	3.621e-01	3.603e-01	1.005	1
CL.H - CL.F == 0	-4.398e-01	3.603e-01	-1.221	1
CL.I - CL.F == 0	-4.398e-01	3.603e-01	-1.221	1
CL.Mix - CL.F == 0	-7.778e-02	3.603e-01	-0.216	1
CL.I - CL.H == 0	9.065e-17	3.603e-01	0.000	1
CL.Mix - CL.H == 0	3.621e-01	3.603e-01	1.005	1
CL.Mix - CL.I == 0	3.621e-01	3.603e-01	1.005	1

(Adjusted p values reported -- bonferroni method)

ASV71

Simultaneous Tests for General Linear Hypotheses

Multiple Comparisons of Means: Tukey Contrasts

Fit: lm(formula = log_cop ~ Treatment, data = dsub)

Linear Hypotheses:

	Estimate	Std. Error	t value	Pr(>|t|)
CL.F - CL.C == 0	-0.26249	0.67823	-0.387	1
CL.H - CL.C == 0	-0.32475	0.67823	-0.479	1

```
CL.I - CL.C == 0    0.08203    0.67823   0.121        1
CL.Mix - CL.C == 0 -0.59754    0.67823  -0.881        1
CL.H - CL.F == 0   -0.06226    0.67823  -0.092        1
CL.I - CL.F == 0    0.34453    0.67823   0.508        1
CL.Mix - CL.F == 0 -0.33504    0.67823  -0.494        1
CL.I - CL.H == 0    0.40679    0.67823   0.600        1
CL.Mix - CL.H == 0 -0.27278    0.67823  -0.402        1
CL.Mix - CL.I == 0 -0.67957    0.67823  -1.002        1
(Adjusted p values reported -- bonferroni method)
```

ASV75

```
       Simultaneous Tests for General Linear Hypotheses

Multiple Comparisons of Means: Tukey Contrasts

Fit: lm(formula = log_cop ~ Treatment, data = dsub)

Linear Hypotheses:
                    Estimate Std. Error t value Pr(>|t|)
CL.F - CL.C == 0    1.174e+00  3.994e-01    2.94   0.0436 *
CL.H - CL.C == 0    1.318e-16  3.994e-01    0.00   1.0000
CL.I - CL.C == 0    1.273e-16  3.994e-01    0.00   1.0000
CL.Mix - CL.C == 0  7.056e-16  3.994e-01    0.00   1.0000
CL.H - CL.F == 0   -1.174e+00  3.994e-01   -2.94   0.0436 *
CL.I - CL.F == 0   -1.174e+00  3.994e-01   -2.94   0.0436 *
CL.Mix - CL.F == 0 -1.174e+00  3.994e-01   -2.94   0.0436 *
CL.I - CL.H == 0   -4.489e-18  3.994e-01    0.00   1.0000
CL.Mix - CL.H == 0  5.738e-16  3.994e-01    0.00   1.0000
CL.Mix - CL.I == 0  5.783e-16  3.994e-01    0.00   1.0000
---
```

```
Signif. codes:  0 ‘***’ 0.001 ‘**’ 0.01 ‘*’ 0.05 ‘.’ 0.1 ‘ ’ 1
(Adjusted p values reported -- bonferroni method)
```

ASV76

```
	 Simultaneous Tests for General Linear Hypotheses

Multiple Comparisons of Means: Tukey Contrasts

Fit: lm(formula = log_cop ~ Treatment, data = dsub)

Linear Hypotheses:
               Estimate Std. Error t value Pr(>|t|)
CL.F - CL.C == 0    0.44222    0.85903   0.515        1
CL.H - CL.C == 0    0.11555    0.85903   0.135        1
CL.I - CL.C == 0    0.18339    0.85903   0.213        1
CL.Mix - CL.C == 0  0.08495    0.85903   0.099        1
CL.H - CL.F == 0   -0.32668    0.85903  -0.380        1
CL.I - CL.F == 0   -0.25883    0.85903  -0.301        1
CL.Mix - CL.F == 0 -0.35727    0.85903  -0.416        1
CL.I - CL.H == 0    0.06784    0.85903   0.079        1
CL.Mix - CL.H == 0 -0.03059    0.85903  -0.036        1
CL.Mix - CL.I == 0 -0.09843    0.85903  -0.115        1
(Adjusted p values reported -- bonferroni method)
```

ASV77

```
	 Simultaneous Tests for General Linear Hypotheses

Multiple Comparisons of Means: Tukey Contrasts

Fit: lm(formula = log_cop ~ Treatment, data = dsub)

Linear Hypotheses:
```

	Estimate	Std. Error	t value	Pr(>|t|)
CL.F - CL.C == 0	-1.44346	0.71100	-2.030	0.459
CL.H - CL.C == 0	-0.32311	0.71100	-0.454	1.000
CL.I - CL.C == 0	-0.36250	0.71100	-0.510	1.000
CL.Mix - CL.C == 0	-1.06743	0.71100	-1.501	1.000
CL.H - CL.F == 0	1.12035	0.71100	1.576	1.000
CL.I - CL.F == 0	1.08096	0.71100	1.520	1.000
CL.Mix - CL.F == 0	0.37603	0.71100	0.529	1.000
CL.I - CL.H == 0	-0.03939	0.71100	-0.055	1.000
CL.Mix - CL.H == 0	-0.74433	0.71100	-1.047	1.000
CL.Mix - CL.I == 0	-0.70493	0.71100	-0.991	1.000

(Adjusted p values reported -- bonferroni method)

ASV78

Simultaneous Tests for General Linear Hypotheses

Multiple Comparisons of Means: Tukey Contrasts

Fit: lm(formula = log_cop ~ Treatment, data = dsub)

Linear Hypotheses:

	Estimate	Std. Error	t value	Pr(>|t|)
CL.F - CL.C == 0	-0.01445	0.82959	-0.017	1
CL.H - CL.C == 0	-0.33606	0.82959	-0.405	1
CL.I - CL.C == 0	0.03860	0.82959	0.047	1
CL.Mix - CL.C == 0	0.75681	0.82959	0.912	1
CL.H - CL.F == 0	-0.32160	0.82959	-0.388	1
CL.I - CL.F == 0	0.05305	0.82959	0.064	1
CL.Mix - CL.F == 0	0.77126	0.82959	0.930	1
CL.I - CL.H == 0	0.37466	0.82959	0.452	1

```
CL.Mix - CL.H == 0  1.09286    0.82959   1.317        1
CL.Mix - CL.I == 0  0.71821    0.82959   0.866        1
(Adjusted p values reported -- bonferroni method)
```

ASV79

```
	 Simultaneous Tests for General Linear Hypotheses

Multiple Comparisons of Means: Tukey Contrasts

Fit: lm(formula = log_cop ~ Treatment, data = dsub)

Linear Hypotheses:
                 Estimate Std. Error t value Pr(>|t|)
CL.F - CL.C == 0    3.556e-01  4.398e-01   0.809        1
CL.H - CL.C == 0    7.100e-01  4.398e-01   1.615        1
CL.I - CL.C == 0    2.380e-16  4.398e-01   0.000        1
CL.Mix - CL.C == 0  3.476e-01  4.398e-01   0.790        1
CL.H - CL.F == 0    3.544e-01  4.398e-01   0.806        1
CL.I - CL.F == 0   -3.556e-01  4.398e-01  -0.809        1
CL.Mix - CL.F == 0 -8.079e-03  4.398e-01  -0.018        1
CL.I - CL.H == 0   -7.100e-01  4.398e-01  -1.615        1
CL.Mix - CL.H == 0 -3.625e-01  4.398e-01  -0.824        1
CL.Mix - CL.I == 0  3.476e-01  4.398e-01   0.790        1
(Adjusted p values reported -- bonferroni method)
```

ASV8

```
	 Simultaneous Tests for General Linear Hypotheses

Multiple Comparisons of Means: Tukey Contrasts

Fit: lm(formula = log_cop ~ Treatment, data = dsub)
```

```
Linear Hypotheses:
                   Estimate Std. Error t value Pr(>|t|)
CL.F - CL.C == 0    -2.4441     1.0476  -2.333  0.22331
CL.H - CL.C == 0    -2.7664     1.0476  -2.641  0.10064
CL.I - CL.C == 0    -3.7885     1.0476  -3.616  0.00539 **
CL.Mix - CL.C == 0  -1.4719     1.0476  -1.405  1.00000
CL.H - CL.F == 0    -0.3223     1.0476  -0.308  1.00000
CL.I - CL.F == 0    -1.3444     1.0476  -1.283  1.00000
CL.Mix - CL.F == 0   0.9722     1.0476   0.928  1.00000
CL.I - CL.H == 0    -1.0221     1.0476  -0.976  1.00000
CL.Mix - CL.H == 0   1.2945     1.0476   1.236  1.00000
CL.Mix - CL.I == 0   2.3166     1.0476   2.211  0.30061
---
Signif. codes:  0 ‘***’ 0.001 ‘**’ 0.01 ‘*’ 0.05 ‘.’ 0.1 ‘ ’ 1
(Adjusted p values reported -- bonferroni method)
```

ASV80

```
	 Simultaneous Tests for General Linear Hypotheses
Multiple Comparisons of Means: Tukey Contrasts
Fit: lm(formula = log_cop ~ Treatment, data = dsub)

Linear Hypotheses:
                    Estimate Std. Error t value Pr(>|t|)
CL.F - CL.C == 0    0.008016   0.610652   0.013        1
CL.H - CL.C == 0   -0.029104   0.610652  -0.048        1
CL.I - CL.C == 0    0.029825   0.610652   0.049        1
CL.Mix - CL.C == 0  0.715274   0.610652   1.171        1
CL.H - CL.F == 0   -0.037120   0.610652  -0.061        1
```

```
CL.I - CL.F == 0    0.021809   0.610652   0.036        1
CL.Mix - CL.F == 0  0.707258   0.610652   1.158        1
CL.I - CL.H == 0    0.058929   0.610652   0.097        1
CL.Mix - CL.H == 0  0.744378   0.610652   1.219        1
CL.Mix - CL.I == 0  0.685449   0.610652   1.122        1
(Adjusted p values reported -- bonferroni method)
```

ASV82

```
	 Simultaneous Tests for General Linear Hypotheses

Multiple Comparisons of Means: Tukey Contrasts

Fit: lm(formula = log_cop ~ Treatment, data = dsub)

Linear Hypotheses:
                  Estimate Std. Error t value Pr(>|t|)
CL.F - CL.C == 0    4.207e-01  4.935e-01   0.853    1.000
CL.H - CL.C == 0    7.603e-01  4.935e-01   1.541    1.000
CL.I - CL.C == 0   -2.725e-01  4.935e-01  -0.552    1.000
CL.Mix - CL.C == 0 -2.725e-01  4.935e-01  -0.552    1.000
CL.H - CL.F == 0    3.395e-01  4.935e-01   0.688    1.000
CL.I - CL.F == 0   -6.933e-01  4.935e-01  -1.405    1.000
CL.Mix - CL.F == 0 -6.933e-01  4.935e-01  -1.405    1.000
CL.I - CL.H == 0   -1.033e+00  4.935e-01  -2.093    0.397
CL.Mix - CL.H == 0 -1.033e+00  4.935e-01  -2.093    0.397
CL.Mix - CL.I == 0 -1.110e-16  4.935e-01   0.000    1.000
(Adjusted p values reported -- bonferroni method)
```

ASV85

Simultaneous Tests for General Linear Hypotheses

Multiple Comparisons of Means: Tukey Contrasts

Fit: lm(formula = log_cop ~ Treatment, data = dsub)

Linear Hypotheses:

	Estimate	Std. Error	t value	Pr(>|t|)
CL.F - CL.C == 0	1.552e-16	2.376e-01	0.000	1
CL.H - CL.C == 0	3.757e-01	2.376e-01	1.581	1
CL.I - CL.C == 0	6.547e-17	2.376e-01	0.000	1
CL.Mix - CL.C == 0	5.580e-17	2.376e-01	0.000	1
CL.H - CL.F == 0	3.757e-01	2.376e-01	1.581	1
CL.I - CL.F == 0	-8.969e-17	2.376e-01	0.000	1
CL.Mix - CL.F == 0	-9.936e-17	2.376e-01	0.000	1
CL.I - CL.H == 0	-3.757e-01	2.376e-01	-1.581	1
CL.Mix - CL.H == 0	-3.757e-01	2.376e-01	-1.581	1
CL.Mix - CL.I == 0	-9.668e-18	2.376e-01	0.000	1

(Adjusted p values reported -- bonferroni method)

ASV86

Simultaneous Tests for General Linear Hypotheses

Multiple Comparisons of Means: Tukey Contrasts

Fit: lm(formula = log_cop ~ Treatment, data = dsub)

Linear Hypotheses:

	Estimate	Std. Error	t value	Pr(>|t|)
CL.F - CL.C == 0	8.632e-01	6.539e-01	1.320	1.000
CL.H - CL.C == 0	7.673e-01	6.539e-01	1.173	1.000
CL.I - CL.C == 0	4.079e-16	6.539e-01	0.000	1.000
CL.Mix - CL.C == 0	1.242e+00	6.539e-01	1.899	0.614

```
CL.H - CL.F == 0   -9.590e-02  6.539e-01  -0.147    1.000
CL.I - CL.F == 0   -8.632e-01  6.539e-01  -1.320    1.000
CL.Mix - CL.F == 0  3.788e-01  6.539e-01   0.579    1.000
CL.I - CL.H == 0   -7.673e-01  6.539e-01  -1.173    1.000
CL.Mix - CL.H == 0  4.747e-01  6.539e-01   0.726    1.000
CL.Mix - CL.I == 0  1.242e+00  6.539e-01   1.899    0.614
(Adjusted p values reported -- bonferroni method)
```

ASV89

```
       Simultaneous Tests for General Linear Hypotheses
Multiple Comparisons of Means: Tukey Contrasts
Fit: lm(formula = log_cop ~ Treatment, data = dsub)

Linear Hypotheses:
               Estimate Std. Error t value Pr(>|t|)
CL.F - CL.C == 0   -0.59691    0.85433  -0.699        1
CL.H - CL.C == 0   -0.93332    0.85433  -1.092        1
CL.I - CL.C == 0   -0.24555    0.85433  -0.287        1
CL.Mix - CL.C == 0 -0.26800    0.85433  -0.314        1
CL.H - CL.F == 0   -0.33641    0.85433  -0.394        1
CL.I - CL.F == 0    0.35136    0.85433   0.411        1
CL.Mix - CL.F == 0  0.32892    0.85433   0.385        1
CL.I - CL.H == 0    0.68777    0.85433   0.805        1
CL.Mix - CL.H == 0  0.66532    0.85433   0.779        1
CL.Mix - CL.I == 0 -0.02244    0.85433  -0.026        1
(Adjusted p values reported -- bonferroni method)
```

ASV9

```
	 Simultaneous Tests for General Linear Hypotheses

Multiple Comparisons of Means: Tukey Contrasts

Fit: lm(formula = log_cop ~ Treatment, data = dsub)

Linear Hypotheses:
                  Estimate Std. Error t value Pr(>|t|)
CL.F - CL.C == 0    1.945e-01  1.825e-01   1.066    1.000
CL.H - CL.C == 0    3.408e-01  1.825e-01   1.867    0.658
CL.I - CL.C == 0    4.105e-01  1.825e-01   2.249    0.274
CL.Mix - CL.C == 0  3.408e-01  1.825e-01   1.867    0.658
CL.H - CL.F == 0    1.462e-01  1.825e-01   0.801    1.000
CL.I - CL.F == 0    2.160e-01  1.825e-01   1.184    1.000
CL.Mix - CL.F == 0  1.463e-01  1.825e-01   0.802    1.000
CL.I - CL.H == 0    6.975e-02  1.825e-01   0.382    1.000
CL.Mix - CL.H == 0  2.627e-05  1.825e-01   0.000    1.000
CL.Mix - CL.I == 0 -6.973e-02  1.825e-01  -0.382    1.000
(Adjusted p values reported -- bonferroni method)
```

ASV90

```
	 Simultaneous Tests for General Linear Hypotheses

Multiple Comparisons of Means: Tukey Contrasts

Fit: lm(formula = log_cop ~ Treatment, data = dsub)

Linear Hypotheses:
                  Estimate Std. Error t value Pr(>|t|)
CL.F - CL.C == 0    3.334e-02  5.520e-01   0.060        1
CL.H - CL.C == 0   -7.107e-01  5.520e-01  -1.287        1
CL.I - CL.C == 0    4.275e-02  5.520e-01   0.077        1
```

```
CL.Mix - CL.C == 0 -7.107e-01  5.520e-01  -1.287        1
CL.H - CL.F == 0   -7.440e-01  5.520e-01  -1.348        1
CL.I - CL.F == 0    9.408e-03  5.520e-01   0.017        1
CL.Mix - CL.F == 0 -7.440e-01  5.520e-01  -1.348        1
CL.I - CL.H == 0    7.534e-01  5.520e-01   1.365        1
CL.Mix - CL.H == 0  2.220e-16  5.520e-01   0.000        1
CL.Mix - CL.I == 0 -7.534e-01  5.520e-01  -1.365        1
(Adjusted p values reported -- bonferroni method)
```

```
ASV91
       Simultaneous Tests for General Linear Hypotheses
Multiple Comparisons of Means: Tukey Contrasts
Fit: lm(formula = log_cop ~ Treatment, data = dsub)

Linear Hypotheses:
                  Estimate Std. Error t value Pr(>|t|)
CL.F - CL.C == 0   -1.095e+00  5.976e-01  -1.833    0.708
CL.H - CL.C == 0   -1.478e+00  5.976e-01  -2.473    0.157
CL.I - CL.C == 0   -1.478e+00  5.976e-01  -2.473    0.157
CL.Mix - CL.C == 0 -4.721e-01  5.976e-01  -0.790    1.000
CL.H - CL.F == 0   -3.827e-01  5.976e-01  -0.640    1.000
CL.I - CL.F == 0   -3.827e-01  5.976e-01  -0.640    1.000
CL.Mix - CL.F == 0  6.233e-01  5.976e-01   1.043    1.000
CL.I - CL.H == 0    5.551e-15  5.976e-01   0.000    1.000
CL.Mix - CL.H == 0  1.006e+00  5.976e-01   1.683    0.965
CL.Mix - CL.I == 0  1.006e+00  5.976e-01   1.683    0.965
(Adjusted p values reported -- bonferroni method)
```

ASV92

```
	 Simultaneous Tests for General Linear Hypotheses

Multiple Comparisons of Means: Tukey Contrasts

Fit: lm(formula = log_cop ~ Treatment, data = dsub)

Linear Hypotheses:
                  Estimate Std. Error t value Pr(>|t|)
CL.F - CL.C == 0    4.259e-01  3.780e-01   1.127        1
CL.H - CL.C == 0    7.852e-16  3.780e-01   0.000        1
CL.I - CL.C == 0    4.194e-01  3.780e-01   1.109        1
CL.Mix - CL.C == 0  2.944e-16  3.780e-01   0.000        1
CL.H - CL.F == 0   -4.259e-01  3.780e-01  -1.127        1
CL.I - CL.F == 0   -6.485e-03  3.780e-01  -0.017        1
CL.Mix - CL.F == 0 -4.259e-01  3.780e-01  -1.127        1
CL.I - CL.H == 0    4.194e-01  3.780e-01   1.109        1
CL.Mix - CL.H == 0 -4.908e-16  3.780e-01   0.000        1
CL.Mix - CL.I == 0 -4.194e-01  3.780e-01  -1.109        1
(Adjusted p values reported -- bonferroni method)
```

ASV94

```
	 Simultaneous Tests for General Linear Hypotheses

Multiple Comparisons of Means: Tukey Contrasts

Fit: lm(formula = log_cop ~ Treatment, data = dsub)

Linear Hypotheses:
                  Estimate Std. Error t value Pr(>|t|)
CL.F - CL.C == 0   -4.545e-01  2.875e-01  -1.581        1
CL.H - CL.C == 0   -4.545e-01  2.875e-01  -1.581        1
```

```
CL.I - CL.C == 0   -4.545e-01  2.875e-01  -1.581        1
CL.Mix - CL.C == 0 -4.545e-01  2.875e-01  -1.581        1
CL.H - CL.F == 0    5.551e-17  2.875e-01   0.000        1
CL.I - CL.F == 0    0.000e+00  2.875e-01   0.000        1
CL.Mix - CL.F == 0  0.000e+00  2.875e-01   0.000        1
CL.I - CL.H == 0   -5.551e-17  2.875e-01   0.000        1
CL.Mix - CL.H == 0 -5.551e-17  2.875e-01   0.000        1
CL.Mix - CL.I == 0  0.000e+00  2.875e-01   0.000        1
(Adjusted p values reported -- bonferroni method)
```
