# Supplementary material for: Mild chronic exposure to pesticides alters physiological markers of honey bee health without perturbing the core gut microbiota
Source: Sci Rep. 2022 Mar 11;12:4281. doi: 10.1038/s41598-022-08009-2 (PMC8917129; doi:10.1038/s41598-022-08009-2)
Supplement: Supplementary file 7 — Supplementary Table 2. [file 41598_2022_8009_MOESM7_ESM.pdf]

Table S2

ASV1

```
	 Simultaneous Tests for General Linear Hypotheses

Multiple Comparisons of Means: Tukey Contrasts


Fit: lm(formula = log_cop ~ Treatment, data = dsub)

Linear Hypotheses:
                     Estimate Std. Error t value Pr(>|t|)
MD.H - MD.F == 0      0.16300    0.21494   0.758    1.000
MD.I - MD.F == 0      0.12810    0.21494   0.596    1.000
MD.Mix - MD.F == 0   -0.19966    0.21494  -0.929    1.000
MD.C - MD.F == 0     -0.09612    0.21494  -0.447    1.000
MD.I - MD.H == 0     -0.03490    0.21494  -0.162    1.000
MD.Mix - MD.H == 0   -0.36267    0.21494  -1.687    0.957
MD.C - MD.H == 0     -0.25912    0.21494  -1.206    1.000
MD.Mix - MD.I == 0   -0.32776    0.21494  -1.525    1.000
MD.C - MD.I == 0     -0.22422    0.21494  -1.043    1.000
MD.C - MD.Mix == 0    0.10355    0.21494   0.482    1.000
(Adjusted p values reported -- bonferroni method)
```

ASV10

Simultaneous Tests for General Linear Hypotheses

Multiple Comparisons of Means: Tukey Contrasts

Fit: lm(formula = log_cop ~ Treatment, data = dsub)

Linear Hypotheses:

	Estimate	Std. Error	t value	Pr(>|t|)
MD.H - MD.F == 0	0.3716	0.6648	0.559	1.000
MD.I - MD.F == 0	-0.7772	0.6648	-1.169	1.000
MD.Mix - MD.F == 0	-0.4595	0.6648	-0.691	1.000
MD.C - MD.F == 0	0.2267	0.6648	0.341	1.000
MD.I - MD.H == 0	-1.1488	0.6648	-1.728	0.881
MD.Mix - MD.H == 0	-0.8311	0.6648	-1.250	1.000
MD.C - MD.H == 0	-0.1448	0.6648	-0.218	1.000
MD.Mix - MD.I == 0	0.3178	0.6648	0.478	1.000
MD.C - MD.I == 0	1.0040	0.6648	1.510	1.000
MD.C - MD.Mix == 0	0.6862	0.6648	1.032	1.000

(Adjusted p values reported -- bonferroni method)

ASV104

Simultaneous Tests for General Linear Hypotheses

```
Multiple Comparisons of Means: Tukey Contrasts


Fit: lm(formula = log_cop ~ Treatment, data = dsub)

Linear Hypotheses:
               Estimate Std. Error t value Pr(>|t|)
MD.H - MD.F == 0    2.327e-16  2.291e-01   0.000        1
MD.I - MD.F == 0    3.623e-01  2.291e-01   1.581        1
MD.Mix - MD.F == 0  1.647e-16  2.291e-01   0.000        1
MD.C - MD.F == 0   -7.850e-17  2.291e-01   0.000        1
MD.I - MD.H == 0    3.623e-01  2.291e-01   1.581        1
MD.Mix - MD.H == 0 -6.803e-17  2.291e-01   0.000        1
MD.C - MD.H == 0   -3.112e-16  2.291e-01   0.000        1
MD.Mix - MD.I == 0 -3.623e-01  2.291e-01  -1.581        1
MD.C - MD.I == 0   -3.623e-01  2.291e-01  -1.581        1
MD.C - MD.Mix == 0 -2.432e-16  2.291e-01   0.000        1
(Adjusted p values reported -- bonferroni method)



ASV107

      Simultaneous Tests for General Linear Hypotheses

Multiple Comparisons of Means: Tukey Contrasts
```

```
Fit: lm(formula = log_cop ~ Treatment, data = dsub)

Linear Hypotheses:
                    Estimate Std. Error t value Pr(>|t|)
MD.H - MD.F == 0    2.774e-01  4.613e-01   0.601        1
MD.I - MD.F == 0   -3.246e-01  4.613e-01  -0.704        1
MD.Mix - MD.F == 0  4.171e-01  4.613e-01   0.904        1
MD.C - MD.F == 0   -3.246e-01  4.613e-01  -0.704        1
MD.I - MD.H == 0   -6.020e-01  4.613e-01  -1.305        1
MD.Mix - MD.H == 0  1.397e-01  4.613e-01   0.303        1
MD.C - MD.H == 0   -6.020e-01  4.613e-01  -1.305        1
MD.Mix - MD.I == 0  7.417e-01  4.613e-01   1.608        1
MD.C - MD.I == 0    3.331e-16  4.613e-01   0.000        1
MD.C - MD.Mix == 0 -7.417e-01  4.613e-01  -1.608        1
(Adjusted p values reported -- bonferroni method)
```

ASV109

```
         Simultaneous Tests for General Linear Hypotheses

Multiple Comparisons of Means: Tukey Contrasts


Fit: lm(formula = log_cop ~ Treatment, data = dsub)

Linear Hypotheses:
```

```
                   Estimate Std. Error t value Pr(>|t|)
MD.H - MD.F == 0   -1.814e-01  1.148e-01  -1.581        1
MD.I - MD.F == 0   -1.814e-01  1.148e-01  -1.581        1
MD.Mix - MD.F == 0 -1.814e-01  1.148e-01  -1.581        1
MD.C - MD.F == 0   -1.814e-01  1.148e-01  -1.581        1
MD.I - MD.H == 0    1.110e-16  1.148e-01   0.000        1
MD.Mix - MD.H == 0  0.000e+00  1.148e-01   0.000        1
MD.C - MD.H == 0   -5.551e-17  1.148e-01   0.000        1
MD.Mix - MD.I == 0 -1.110e-16  1.148e-01   0.000        1
MD.C - MD.I == 0   -1.665e-16  1.148e-01   0.000        1
MD.C - MD.Mix == 0 -5.551e-17  1.148e-01   0.000        1
(Adjusted p values reported -- bonferroni method)
```

ASV11

```
        Simultaneous Tests for General Linear Hypotheses

Multiple Comparisons of Means: Tukey Contrasts


Fit: lm(formula = log_cop ~ Treatment, data = dsub)

Linear Hypotheses:
               Estimate Std. Error t value Pr(>|t|)
MD.H - MD.F == 0    -3.5664     0.8870  -4.021 0.001370 **
MD.I - MD.F == 0     0.2527     0.8870   0.285 1.000000
```

```
MD.Mix - MD.F == 0  -0.3145     0.8870  -0.355 1.000000
MD.C - MD.F == 0    -2.4793     0.8870  -2.795 0.065872 .
MD.I - MD.H == 0     3.8191     0.8870   4.305 0.000498 ***
MD.Mix - MD.H == 0   3.2519     0.8870   3.666 0.004575 **
MD.C - MD.H == 0     1.0871     0.8870   1.226 1.000000
MD.Mix - MD.I == 0  -0.5672     0.8870  -0.639 1.000000
MD.C - MD.I == 0    -2.7320     0.8870  -3.080 0.028939 *
MD.C - MD.Mix == 0  -2.1648     0.8870  -2.440 0.170309
---
Signif. codes:  0 ‘***’ 0.001 ‘**’ 0.01 ‘*’ 0.05 ‘.’ 0.1 ‘ ’ 1
(Adjusted p values reported -- bonferroni method)
```

ASV112

```
	 Simultaneous Tests for General Linear Hypotheses

Multiple Comparisons of Means: Tukey Contrasts


Fit: lm(formula = log_cop ~ Treatment, data = dsub)

Linear Hypotheses:
             Estimate Std. Error t value Pr(>|t|)
MD.H - MD.F == 0    -0.6887     0.5440  -1.266    1.000
MD.I - MD.F == 0    -0.9540     0.5440  -1.754    0.836
MD.Mix - MD.F == 0   0.4121     0.5440   0.758    1.000
```

```
MD.C - MD.F == 0    -0.1244     0.5440  -0.229    1.000
MD.I - MD.H == 0    -0.2653     0.5440  -0.488    1.000
MD.Mix - MD.H == 0   1.1008     0.5440   2.024    0.466
MD.C - MD.H == 0     0.5643     0.5440   1.037    1.000
MD.Mix - MD.I == 0   1.3661     0.5440   2.511    0.142
MD.C - MD.I == 0     0.8296     0.5440   1.525    1.000
MD.C - MD.Mix == 0  -0.5365     0.5440  -0.986    1.000
(Adjusted p values reported -- bonferroni method)
```

ASV116

```
       Simultaneous Tests for General Linear Hypotheses

Multiple Comparisons of Means: Tukey Contrasts


Fit: lm(formula = log_cop ~ Treatment, data = dsub)

Linear Hypotheses:
               Estimate Std. Error t value Pr(>|t|)
MD.H - MD.F == 0    2.653e-16  2.915e-01   0.000   1.0000
MD.I - MD.F == 0    1.923e-16  2.915e-01   0.000   1.0000
MD.Mix - MD.F == 0  8.547e-01  2.915e-01   2.932   0.0447 *
MD.C - MD.F == 0   -3.925e-17  2.915e-01   0.000   1.0000
MD.I - MD.H == 0   -7.300e-17  2.915e-01   0.000   1.0000
MD.Mix - MD.H == 0  8.547e-01  2.915e-01   2.932   0.0447 *
```

```
MD.C - MD.H == 0   -3.045e-16  2.915e-01   0.000   1.0000
MD.Mix - MD.I == 0  8.547e-01  2.915e-01   2.932   0.0447 *
MD.C - MD.I == 0   -2.315e-16  2.915e-01   0.000   1.0000
MD.C - MD.Mix == 0 -8.547e-01  2.915e-01  -2.932   0.0447 *
---
Signif. codes:  0 ‘***’ 0.001 ‘**’ 0.01 ‘*’ 0.05 ‘.’ 0.1 ‘ ’ 1
(Adjusted p values reported -- bonferroni method)
```

ASV119

```
       Simultaneous Tests for General Linear Hypotheses

Multiple Comparisons of Means: Tukey Contrasts


Fit: lm(formula = log_cop ~ Treatment, data = dsub)

Linear Hypotheses:
             Estimate Std. Error t value Pr(>|t|)
MD.H - MD.F == 0   -0.27640    0.46898  -0.589   1.0000
MD.I - MD.F == 0   -0.26459    0.46898  -0.564   1.0000
MD.Mix - MD.F == 0 -0.51287    0.46898  -1.094   1.0000
MD.C - MD.F == 0    0.92669    0.46898   1.976   0.5183
MD.I - MD.H == 0    0.01181    0.46898   0.025   1.0000
MD.Mix - MD.H == 0 -0.23647    0.46898  -0.504   1.0000
MD.C - MD.H == 0    1.20309    0.46898   2.565   0.1230
```

```
MD.Mix - MD.I == 0 -0.24829    0.46898  -0.529   1.0000
MD.C - MD.I == 0    1.19128    0.46898   2.540   0.1315
MD.C - MD.Mix == 0  1.43956    0.46898   3.070   0.0298 *
---
Signif. codes:  0 ‘***’ 0.001 ‘**’ 0.01 ‘*’ 0.05 ‘.’ 0.1 ‘ ’ 1
(Adjusted p values reported -- bonferroni method)
```

ASV12

```
	 Simultaneous Tests for General Linear Hypotheses

Multiple Comparisons of Means: Tukey Contrasts


Fit: lm(formula = log_cop ~ Treatment, data = dsub)

Linear Hypotheses:
             Estimate Std. Error t value Pr(>|t|)
MD.H - MD.F == 0    1.72128    0.77137   2.231    0.286
MD.I - MD.F == 0    0.16770    0.77137   0.217    1.000
MD.Mix - MD.F == 0  0.06727    0.77137   0.087    1.000
MD.C - MD.F == 0    0.05474    0.77137   0.071    1.000
MD.I - MD.H == 0   -1.55357    0.77137  -2.014    0.476
MD.Mix - MD.H == 0 -1.65400    0.77137  -2.144    0.353
MD.C - MD.H == 0   -1.66654    0.77137  -2.160    0.339
MD.Mix - MD.I == 0 -0.10043    0.77137  -0.130    1.000
```

```
MD.C - MD.I == 0   -0.11296    0.77137  -0.146    1.000
MD.C - MD.Mix == 0 -0.01253    0.77137  -0.016    1.000
(Adjusted p values reported -- bonferroni method)
```

ASV123

```
	 Simultaneous Tests for General Linear Hypotheses

Multiple Comparisons of Means: Tukey Contrasts


Fit: lm(formula = log_cop ~ Treatment, data = dsub)

Linear Hypotheses:
             Estimate Std. Error t value Pr(>|t|)
MD.H - MD.F == 0    0.59233    0.56209   1.054        1
MD.I - MD.F == 0    0.03455    0.56209   0.061        1
MD.Mix - MD.F == 0  0.48948    0.56209   0.871        1
MD.C - MD.F == 0    0.76465    0.56209   1.360        1
MD.I - MD.H == 0   -0.55778    0.56209  -0.992        1
MD.Mix - MD.H == 0 -0.10285    0.56209  -0.183        1
MD.C - MD.H == 0    0.17232    0.56209   0.307        1
MD.Mix - MD.I == 0  0.45493    0.56209   0.809        1
MD.C - MD.I == 0    0.73010    0.56209   1.299        1
MD.C - MD.Mix == 0  0.27517    0.56209   0.490        1
(Adjusted p values reported -- bonferroni method)
```

ASV13

```
         Simultaneous Tests for General Linear Hypotheses

Multiple Comparisons of Means: Tukey Contrasts


Fit: lm(formula = log_cop ~ Treatment, data = dsub)

Linear Hypotheses:
               Estimate Std. Error t value Pr(>|t|)
MD.H - MD.F == 0    2.114e-16  2.653e-01   0.000    1.000
MD.I - MD.F == 0   -2.404e-16  2.653e-01   0.000    1.000
MD.Mix - MD.F == 0  2.186e-01  2.653e-01   0.824    1.000
MD.C - MD.F == 0    5.214e-01  2.653e-01   1.965    0.531
MD.I - MD.H == 0   -4.518e-16  2.653e-01   0.000    1.000
MD.Mix - MD.H == 0  2.186e-01  2.653e-01   0.824    1.000
MD.C - MD.H == 0    5.214e-01  2.653e-01   1.965    0.531
MD.Mix - MD.I == 0  2.186e-01  2.653e-01   0.824    1.000
MD.C - MD.I == 0    5.214e-01  2.653e-01   1.965    0.531
MD.C - MD.Mix == 0  3.028e-01  2.653e-01   1.141    1.000
(Adjusted p values reported -- bonferroni method)
```

ASV137

```
	 Simultaneous Tests for General Linear Hypotheses

Multiple Comparisons of Means: Tukey Contrasts


Fit: lm(formula = log_cop ~ Treatment, data = dsub)

Linear Hypotheses:
                   Estimate Std. Error t value Pr(>|t|)
MD.H - MD.F == 0    -0.2413     0.4909  -0.491   1.0000
MD.I - MD.F == 0     0.2118     0.4909   0.431   1.0000
MD.Mix - MD.F == 0   0.4923     0.4909   1.003   1.0000
MD.C - MD.F == 0     1.5898     0.4909   3.238   0.0179 *
MD.I - MD.H == 0     0.4531     0.4909   0.923   1.0000
MD.Mix - MD.H == 0   0.7336     0.4909   1.494   1.0000
MD.C - MD.H == 0     1.8311     0.4909   3.730   0.0037 **
MD.Mix - MD.I == 0   0.2805     0.4909   0.571   1.0000
MD.C - MD.I == 0     1.3780     0.4909   2.807   0.0637 .
MD.C - MD.Mix == 0   1.0974     0.4909   2.235   0.2837
---
Signif. codes:  0 ‘***’ 0.001 ‘**’ 0.01 ‘*’ 0.05 ‘.’ 0.1 ‘ ’ 1
(Adjusted p values reported -- bonferroni method)
```

ASV14

```
	 Simultaneous Tests for General Linear Hypotheses

Multiple Comparisons of Means: Tukey Contrasts


Fit: lm(formula = log_cop ~ Treatment, data = dsub)

Linear Hypotheses:
                     Estimate Std. Error t value Pr(>|t|)
MD.H - MD.F == 0      0.27498    0.35587   0.773        1
MD.I - MD.F == 0      0.30455    0.35587   0.856        1
MD.Mix - MD.F == 0    0.31944    0.35587   0.898        1
MD.C - MD.F == 0      0.21495    0.35587   0.604        1
MD.I - MD.H == 0      0.02956    0.35587   0.083        1
MD.Mix - MD.H == 0    0.04445    0.35587   0.125        1
MD.C - MD.H == 0     -0.06004    0.35587  -0.169        1
MD.Mix - MD.I == 0    0.01489    0.35587   0.042        1
MD.C - MD.I == 0     -0.08960    0.35587  -0.252        1
MD.C - MD.Mix == 0   -0.10449    0.35587  -0.294        1
(Adjusted p values reported -- bonferroni method)
```

ASV145

```
	 Simultaneous Tests for General Linear Hypotheses
```

```
Multiple Comparisons of Means: Tukey Contrasts


Fit: lm(formula = log_cop ~ Treatment, data = dsub)

Linear Hypotheses:
                Estimate Std. Error t value Pr(>|t|)
MD.H - MD.F == 0    3.486e-01  2.204e-01   1.581        1
MD.I - MD.F == 0   -2.201e-18  2.204e-01   0.000        1
MD.Mix - MD.F == 0 -1.301e-17  2.204e-01   0.000        1
MD.C - MD.F == 0    3.734e-16  2.204e-01   0.000        1
MD.I - MD.H == 0   -3.486e-01  2.204e-01  -1.581        1
MD.Mix - MD.H == 0 -3.486e-01  2.204e-01  -1.581        1
MD.C - MD.H == 0   -3.486e-01  2.204e-01  -1.581        1
MD.Mix - MD.I == 0 -1.081e-17  2.204e-01   0.000        1
MD.C - MD.I == 0    3.756e-16  2.204e-01   0.000        1
MD.C - MD.Mix == 0  3.864e-16  2.204e-01   0.000        1
(Adjusted p values reported -- bonferroni method)



ASV146

       Simultaneous Tests for General Linear Hypotheses

Multiple Comparisons of Means: Tukey Contrasts
```

```
Fit: lm(formula = log_cop ~ Treatment, data = dsub)

Linear Hypotheses:
                Estimate Std. Error t value Pr(>|t|)
MD.H - MD.F == 0    9.249e-17  2.837e-01    0.00   1.0000
MD.I - MD.F == 0   -4.752e-16  2.837e-01    0.00   1.0000
MD.Mix - MD.F == 0 -2.720e-16  2.837e-01    0.00   1.0000
MD.C - MD.F == 0    8.312e-01  2.837e-01    2.93   0.0448 *
MD.I - MD.H == 0   -5.677e-16  2.837e-01    0.00   1.0000
MD.Mix - MD.H == 0 -3.644e-16  2.837e-01    0.00   1.0000
MD.C - MD.H == 0    8.312e-01  2.837e-01    2.93   0.0448 *
MD.Mix - MD.I == 0  2.033e-16  2.837e-01    0.00   1.0000
MD.C - MD.I == 0    8.312e-01  2.837e-01    2.93   0.0448 *
MD.C - MD.Mix == 0  8.312e-01  2.837e-01    2.93   0.0448 *
---
Signif. codes:  0 ‘***’ 0.001 ‘**’ 0.01 ‘*’ 0.05 ‘.’ 0.1 ‘ ’ 1
(Adjusted p values reported -- bonferroni method)
```

ASV15

```
       Simultaneous Tests for General Linear Hypotheses

Multiple Comparisons of Means: Tukey Contrasts
```

Fit: lm(formula = log_cop ~ Treatment, data = dsub)

Linear Hypotheses:

	Estimate	Std. Error	t value	Pr(>|t|)
MD.H - MD.F == 0	-0.243341	0.348011	-0.699	1
MD.I - MD.F == 0	-0.483999	0.348011	-1.391	1
MD.Mix - MD.F == 0	-0.201812	0.348011	-0.580	1
MD.C - MD.F == 0	-0.248824	0.348011	-0.715	1
MD.I - MD.H == 0	-0.240658	0.348011	-0.692	1
MD.Mix - MD.H == 0	0.041529	0.348011	0.119	1
MD.C - MD.H == 0	-0.005482	0.348011	-0.016	1
MD.Mix - MD.I == 0	0.282187	0.348011	0.811	1
MD.C - MD.I == 0	0.235176	0.348011	0.676	1
MD.C - MD.Mix == 0	-0.047011	0.348011	-0.135	1

(Adjusted p values reported -- bonferroni method)

ASV155

Simultaneous Tests for General Linear Hypotheses

Multiple Comparisons of Means: Tukey Contrasts

Fit: lm(formula = log_cop ~ Treatment, data = dsub)

Linear Hypotheses:

```
                  Estimate Std. Error t value Pr(>|t|)
MD.H - MD.F == 0   -5.942e-01  2.567e-01  -2.315    0.234
MD.I - MD.F == 0   -5.942e-01  2.567e-01  -2.315    0.234
MD.Mix - MD.F == 0 -5.942e-01  2.567e-01  -2.315    0.234
MD.C - MD.F == 0   -5.942e-01  2.567e-01  -2.315    0.234
MD.I - MD.H == 0    4.441e-16  2.567e-01   0.000    1.000
MD.Mix - MD.H == 0  1.110e-16  2.567e-01   0.000    1.000
MD.C - MD.H == 0    3.331e-16  2.567e-01   0.000    1.000
MD.Mix - MD.I == 0 -3.331e-16  2.567e-01   0.000    1.000
MD.C - MD.I == 0   -1.110e-16  2.567e-01   0.000    1.000
MD.C - MD.Mix == 0  2.220e-16  2.567e-01   0.000    1.000
(Adjusted p values reported -- bonferroni method)
```

ASV16

```
	 Simultaneous Tests for General Linear Hypotheses

Multiple Comparisons of Means: Tukey Contrasts


Fit: lm(formula = log_cop ~ Treatment, data = dsub)

Linear Hypotheses:
                Estimate Std. Error t value Pr(>|t|)
MD.H - MD.F == 0   -0.90745    1.04241  -0.871    1.000
MD.I - MD.F == 0   -0.70795    1.04241  -0.679    1.000
```

```
MD.Mix - MD.F == 0 -1.89617    1.04241  -1.819    0.729
MD.C - MD.F == 0   -0.72502    1.04241  -0.696    1.000
MD.I - MD.H == 0    0.19950    1.04241   0.191    1.000
MD.Mix - MD.H == 0 -0.98872    1.04241  -0.948    1.000
MD.C - MD.H == 0    0.18243    1.04241   0.175    1.000
MD.Mix - MD.I == 0 -1.18822    1.04241  -1.140    1.000
MD.C - MD.I == 0   -0.01707    1.04241  -0.016    1.000
MD.C - MD.Mix == 0  1.17115    1.04241   1.123    1.000
(Adjusted p values reported -- bonferroni method)




ASV166


	 Simultaneous Tests for General Linear Hypotheses


Multiple Comparisons of Means: Tukey Contrasts



Fit: lm(formula = log_cop ~ Treatment, data = dsub)


Linear Hypotheses:
               Estimate Std. Error t value Pr(>|t|)
MD.H - MD.F == 0    1.031e+00  2.928e-01    3.52  0.00736 **
MD.I - MD.F == 0   -2.595e-18  2.928e-01    0.00  1.00000
MD.Mix - MD.F == 0  3.701e-18  2.928e-01    0.00  1.00000
MD.C - MD.F == 0   -6.570e-17  2.928e-01    0.00  1.00000
MD.I - MD.H == 0   -1.031e+00  2.928e-01   -3.52  0.00736 **
```

```
MD.Mix - MD.H == 0 -1.031e+00  2.928e-01   -3.52  0.00736 **
MD.C - MD.H == 0   -1.031e+00  2.928e-01   -3.52  0.00736 **
MD.Mix - MD.I == 0  6.297e-18  2.928e-01    0.00  1.00000
MD.C - MD.I == 0   -6.311e-17  2.928e-01    0.00  1.00000
MD.C - MD.Mix == 0 -6.941e-17  2.928e-01    0.00  1.00000
---
Signif. codes:  0 ‘***’ 0.001 ‘**’ 0.01 ‘*’ 0.05 ‘.’ 0.1 ‘ ’ 1
(Adjusted p values reported -- bonferroni method)
```

ASV17

```
       Simultaneous Tests for General Linear Hypotheses

Multiple Comparisons of Means: Tukey Contrasts


Fit: lm(formula = log_cop ~ Treatment, data = dsub)

Linear Hypotheses:
             Estimate Std. Error t value Pr(>|t|)
MD.H - MD.F == 0    0.06968    1.00056   0.070  1.00000
MD.I - MD.F == 0   -1.79589    1.00056  -1.795  0.76702
MD.Mix - MD.F == 0  0.35122    1.00056   0.351  1.00000
MD.C - MD.F == 0   -3.25315    1.00056  -3.251  0.01722 *
MD.I - MD.H == 0   -1.86557    1.00056  -1.865  0.66161
MD.Mix - MD.H == 0  0.28154    1.00056   0.281  1.00000
```

```
MD.C - MD.H == 0   -3.32283    1.00056  -3.321  0.01388 *
MD.Mix - MD.I == 0  2.14712    1.00056   2.146  0.35116
MD.C - MD.I == 0   -1.45726    1.00056  -1.456  1.00000
MD.C - MD.Mix == 0 -3.60438    1.00056  -3.602  0.00564 **
---
Signif. codes:  0 ‘***’ 0.001 ‘**’ 0.01 ‘*’ 0.05 ‘.’ 0.1 ‘ ’ 1
(Adjusted p values reported -- bonferroni method)
```

ASV172

```
	 Simultaneous Tests for General Linear Hypotheses

Multiple Comparisons of Means: Tukey Contrasts


Fit: lm(formula = log_cop ~ Treatment, data = dsub)

Linear Hypotheses:
               Estimate Std. Error t value Pr(>|t|)
MD.H - MD.F == 0   -1.552e-17  1.863e-01   0.000        1
MD.I - MD.F == 0    2.945e-01  1.863e-01   1.581        1
MD.Mix - MD.F == 0 -1.764e-16  1.863e-01   0.000        1
MD.C - MD.F == 0   -6.379e-17  1.863e-01   0.000        1
MD.I - MD.H == 0    2.945e-01  1.863e-01   1.581        1
MD.Mix - MD.H == 0 -1.608e-16  1.863e-01   0.000        1
MD.C - MD.H == 0   -4.827e-17  1.863e-01   0.000        1
```

```
MD.Mix - MD.I == 0 -2.945e-01  1.863e-01  -1.581        1
MD.C - MD.I == 0   -2.945e-01  1.863e-01  -1.581        1
MD.C - MD.Mix == 0  1.126e-16  1.863e-01   0.000        1
(Adjusted p values reported -- bonferroni method)
```

ASV18

```
         Simultaneous Tests for General Linear Hypotheses

Multiple Comparisons of Means: Tukey Contrasts


Fit: lm(formula = log_cop ~ Treatment, data = dsub)

Linear Hypotheses:
             Estimate Std. Error t value Pr(>|t|)
MD.H - MD.F == 0    -0.3224     1.0942  -0.295   1.0000
MD.I - MD.F == 0    -2.2352     1.0942  -2.043   0.4459
MD.Mix - MD.F == 0   0.6779     1.0942   0.620   1.0000
MD.C - MD.F == 0     0.2109     1.0942   0.193   1.0000
MD.I - MD.H == 0    -1.9127     1.0942  -1.748   0.8454
MD.Mix - MD.H == 0   1.0003     1.0942   0.914   1.0000
MD.C - MD.H == 0     0.5334     1.0942   0.487   1.0000
MD.Mix - MD.I == 0   2.9131     1.0942   2.662   0.0949 .
MD.C - MD.I == 0     2.4461     1.0942   2.236   0.2836
MD.C - MD.Mix == 0  -0.4670     1.0942  -0.427   1.0000
```

```
---
Signif. codes:  0 ‘***’ 0.001 ‘**’ 0.01 ‘*’ 0.05 ‘.’ 0.1 ‘ ’ 1
(Adjusted p values reported -- bonferroni method)




ASV181

	 Simultaneous Tests for General Linear Hypotheses

Multiple Comparisons of Means: Tukey Contrasts


Fit: lm(formula = log_cop ~ Treatment, data = dsub)

Linear Hypotheses:
                  Estimate Std. Error t value Pr(>|t|)
MD.H - MD.F == 0   -1.481e-16  1.863e-01   0.000        1
MD.I - MD.F == 0   -3.885e-16  1.863e-01   0.000        1
MD.Mix - MD.F == 0 -2.040e-16  1.863e-01   0.000        1
MD.C - MD.F == 0    2.946e-01  1.863e-01   1.581        1
MD.I - MD.H == 0   -2.404e-16  1.863e-01   0.000        1
MD.Mix - MD.H == 0 -5.585e-17  1.863e-01   0.000        1
MD.C - MD.H == 0    2.946e-01  1.863e-01   1.581        1
MD.Mix - MD.I == 0  1.845e-16  1.863e-01   0.000        1
MD.C - MD.I == 0    2.946e-01  1.863e-01   1.581        1
MD.C - MD.Mix == 0  2.946e-01  1.863e-01   1.581        1
(Adjusted p values reported -- bonferroni method)
```

ASV186

```
	 Simultaneous Tests for General Linear Hypotheses

Multiple Comparisons of Means: Tukey Contrasts


Fit: lm(formula = log_cop ~ Treatment, data = dsub)

Linear Hypotheses:
               Estimate Std. Error t value Pr(>|t|)  
MD.H - MD.F == 0    7.384e-15  2.624e-01    0.00   1.0000  
MD.I - MD.F == 0   -6.582e-16  2.624e-01    0.00   1.0000  
MD.Mix - MD.F == 0 -3.399e-16  2.624e-01    0.00   1.0000  
MD.C - MD.F == 0    7.689e-01  2.624e-01    2.93   0.0448 *
MD.I - MD.H == 0   -8.042e-15  2.624e-01    0.00   1.0000  
MD.Mix - MD.H == 0 -7.724e-15  2.624e-01    0.00   1.0000  
MD.C - MD.H == 0    7.689e-01  2.624e-01    2.93   0.0448 *
MD.Mix - MD.I == 0  3.182e-16  2.624e-01    0.00   1.0000  
MD.C - MD.I == 0    7.689e-01  2.624e-01    2.93   0.0448 *
MD.C - MD.Mix == 0  7.689e-01  2.624e-01    2.93   0.0448 *
---
Signif. codes:  0 ‘***’ 0.001 ‘**’ 0.01 ‘*’ 0.05 ‘.’ 0.1 ‘ ’ 1
(Adjusted p values reported -- bonferroni method)
```

ASV188

```
	 Simultaneous Tests for General Linear Hypotheses

Multiple Comparisons of Means: Tukey Contrasts


Fit: lm(formula = log_cop ~ Treatment, data = dsub)

Linear Hypotheses:
               Estimate Std. Error t value Pr(>|t|)
MD.H - MD.F == 0   -6.853e-01  2.636e-01  -2.600    0.112
MD.I - MD.F == 0   -6.853e-01  2.636e-01  -2.600    0.112
MD.Mix - MD.F == 0 -4.923e-01  2.636e-01  -1.868    0.657
MD.C - MD.F == 0   -6.853e-01  2.636e-01  -2.600    0.112
MD.I - MD.H == 0    4.441e-16  2.636e-01   0.000    1.000
MD.Mix - MD.H == 0  1.930e-01  2.636e-01   0.732    1.000
MD.C - MD.H == 0    1.110e-16  2.636e-01   0.000    1.000
MD.Mix - MD.I == 0  1.930e-01  2.636e-01   0.732    1.000
MD.C - MD.I == 0   -3.331e-16  2.636e-01   0.000    1.000
MD.C - MD.Mix == 0 -1.930e-01  2.636e-01  -0.732    1.000
(Adjusted p values reported -- bonferroni method)
```

ASV19

```
	 Simultaneous Tests for General Linear Hypotheses

Multiple Comparisons of Means: Tukey Contrasts


Fit: lm(formula = log_cop ~ Treatment, data = dsub)

Linear Hypotheses:
                     Estimate Std. Error t value Pr(>|t|)
MD.H - MD.F == 0   -1.770e+00  8.406e-01  -2.105    0.386
MD.I - MD.F == 0   -2.332e-01  8.406e-01  -0.277    1.000
MD.Mix - MD.F == 0  6.560e-01  8.406e-01   0.780    1.000
MD.C - MD.F == 0   -1.770e+00  8.406e-01  -2.105    0.386
MD.I - MD.H == 0    1.537e+00  8.406e-01   1.828    0.715
MD.Mix - MD.H == 0  2.426e+00  8.406e-01   2.886    0.051 .
MD.C - MD.H == 0   -6.661e-16  8.406e-01   0.000    1.000
MD.Mix - MD.I == 0  8.892e-01  8.406e-01   1.058    1.000
MD.C - MD.I == 0   -1.537e+00  8.406e-01  -1.828    0.715
MD.C - MD.Mix == 0 -2.426e+00  8.406e-01  -2.886    0.051 .
---
Signif. codes:  0 ‘***’ 0.001 ‘**’ 0.01 ‘*’ 0.05 ‘.’ 0.1 ‘ ’ 1
(Adjusted p values reported -- bonferroni method)
```

ASV194

Simultaneous Tests for General Linear Hypotheses

Multiple Comparisons of Means: Tukey Contrasts

Fit: lm(formula = log_cop ~ Treatment, data = dsub)

Linear Hypotheses:

```
                     Estimate Std. Error t value Pr(>|t|)
MD.H - MD.F == 0   -2.674e-01  2.282e-01  -1.172        1
MD.I - MD.F == 0   -2.505e-02  2.282e-01  -0.110        1
MD.Mix - MD.F == 0 -2.674e-01  2.282e-01  -1.172        1
MD.C - MD.F == 0   -2.674e-01  2.282e-01  -1.172        1
MD.I - MD.H == 0    2.423e-01  2.282e-01   1.062        1
MD.Mix - MD.H == 0  5.551e-17  2.282e-01   0.000        1
MD.C - MD.H == 0   -6.106e-16  2.282e-01   0.000        1
MD.Mix - MD.I == 0 -2.423e-01  2.282e-01  -1.062        1
MD.C - MD.I == 0   -2.423e-01  2.282e-01  -1.062        1
MD.C - MD.Mix == 0 -6.661e-16  2.282e-01   0.000        1
(Adjusted p values reported -- bonferroni method)
```

ASV197

Simultaneous Tests for General Linear Hypotheses

```
Multiple Comparisons of Means: Tukey Contrasts


Fit: lm(formula = log_cop ~ Treatment, data = dsub)

Linear Hypotheses:
               Estimate Std. Error t value Pr(>|t|)
MD.H - MD.F == 0   -4.583e-01  1.980e-01  -2.314    0.234
MD.I - MD.F == 0   -4.583e-01  1.980e-01  -2.314    0.234
MD.Mix - MD.F == 0 -4.583e-01  1.980e-01  -2.314    0.234
MD.C - MD.F == 0   -4.583e-01  1.980e-01  -2.314    0.234
MD.I - MD.H == 0   -2.220e-16  1.980e-01   0.000    1.000
MD.Mix - MD.H == 0 -4.996e-16  1.980e-01   0.000    1.000
MD.C - MD.H == 0   -5.551e-17  1.980e-01   0.000    1.000
MD.Mix - MD.I == 0 -2.776e-16  1.980e-01   0.000    1.000
MD.C - MD.I == 0    1.665e-16  1.980e-01   0.000    1.000
MD.C - MD.Mix == 0  4.441e-16  1.980e-01   0.000    1.000
(Adjusted p values reported -- bonferroni method)



ASV199

       Simultaneous Tests for General Linear Hypotheses

Multiple Comparisons of Means: Tukey Contrasts
```

```
Fit: lm(formula = log_cop ~ Treatment, data = dsub)

Linear Hypotheses:
                    Estimate Std. Error t value Pr(>|t|)
MD.H - MD.F == 0    5.493e-16  1.775e-01   0.000        1
MD.I - MD.F == 0    8.024e-16  1.775e-01   0.000        1
MD.Mix - MD.F == 0  3.399e-16  1.775e-01   0.000        1
MD.C - MD.F == 0    2.806e-01  1.775e-01   1.581        1
MD.I - MD.H == 0    2.530e-16  1.775e-01   0.000        1
MD.Mix - MD.H == 0 -2.094e-16  1.775e-01   0.000        1
MD.C - MD.H == 0    2.806e-01  1.775e-01   1.581        1
MD.Mix - MD.I == 0 -4.624e-16  1.775e-01   0.000        1
MD.C - MD.I == 0    2.806e-01  1.775e-01   1.581        1
MD.C - MD.Mix == 0  2.806e-01  1.775e-01   1.581        1
(Adjusted p values reported -- bonferroni method)
```

ASV2

```
         Simultaneous Tests for General Linear Hypotheses

Multiple Comparisons of Means: Tukey Contrasts


Fit: lm(formula = log_cop ~ Treatment, data = dsub)

Linear Hypotheses:
```

```
             Estimate Std. Error t value Pr(>|t|)
MD.H - MD.F == 0    0.05414    0.17517   0.309    1.000
MD.I - MD.F == 0   -0.27828    0.17517  -1.589    1.000
MD.Mix - MD.F == 0  0.12221    0.17517   0.698    1.000
MD.C - MD.F == 0   -0.07848    0.17517  -0.448    1.000
MD.I - MD.H == 0   -0.33242    0.17517  -1.898    0.616
MD.Mix - MD.H == 0  0.06807    0.17517   0.389    1.000
MD.C - MD.H == 0   -0.13262    0.17517  -0.757    1.000
MD.Mix - MD.I == 0  0.40049    0.17517   2.286    0.251
MD.C - MD.I == 0    0.19980    0.17517   1.141    1.000
MD.C - MD.Mix == 0 -0.20069    0.17517  -1.146    1.000
(Adjusted p values reported -- bonferroni method)
```

ASV20

```
       Simultaneous Tests for General Linear Hypotheses

Multiple Comparisons of Means: Tukey Contrasts


Fit: lm(formula = log_cop ~ Treatment, data = dsub)

Linear Hypotheses:
             Estimate Std. Error t value Pr(>|t|)
MD.H - MD.F == 0    1.69060    0.73816   2.290   0.2482
MD.I - MD.F == 0    0.29274    0.73816   0.397   1.0000
```

```
MD.Mix - MD.F == 0 -0.01059    0.73816  -0.014   1.0000
MD.C - MD.F == 0   -0.45258    0.73816  -0.613   1.0000
MD.I - MD.H == 0   -1.39786    0.73816  -1.894   0.6212
MD.Mix - MD.H == 0 -1.70119    0.73816  -2.305   0.2396
MD.C - MD.H == 0   -2.14318    0.73816  -2.903   0.0485 *
MD.Mix - MD.I == 0 -0.30333    0.73816  -0.411   1.0000
MD.C - MD.I == 0   -0.74532    0.73816  -1.010   1.0000
MD.C - MD.Mix == 0 -0.44199    0.73816  -0.599   1.0000
---
Signif. codes:  0 ‘***’ 0.001 ‘**’ 0.01 ‘*’ 0.05 ‘.’ 0.1 ‘ ’ 1
(Adjusted p values reported -- bonferroni method)
```

ASV200

```
	 Simultaneous Tests for General Linear Hypotheses

Multiple Comparisons of Means: Tukey Contrasts


Fit: lm(formula = log_cop ~ Treatment, data = dsub)

Linear Hypotheses:
               Estimate Std. Error t value Pr(>|t|)
MD.H - MD.F == 0   -2.265e-01  2.465e-01  -0.919        1
MD.I - MD.F == 0   -4.213e-02  2.465e-01  -0.171        1
MD.Mix - MD.F == 0 -2.265e-01  2.465e-01  -0.919        1
```

```
MD.C - MD.F == 0    3.166e-02  2.465e-01   0.128        1
MD.I - MD.H == 0    1.843e-01  2.465e-01   0.748        1
MD.Mix - MD.H == 0 -3.886e-16  2.465e-01   0.000        1
MD.C - MD.H == 0    2.581e-01  2.465e-01   1.047        1
MD.Mix - MD.I == 0 -1.843e-01  2.465e-01  -0.748        1
MD.C - MD.I == 0    7.379e-02  2.465e-01   0.299        1
MD.C - MD.Mix == 0  2.581e-01  2.465e-01   1.047        1
(Adjusted p values reported -- bonferroni method)
```

ASV205

```
       Simultaneous Tests for General Linear Hypotheses

Multiple Comparisons of Means: Tukey Contrasts


Fit: lm(formula = log_cop ~ Treatment, data = dsub)

Linear Hypotheses:
               Estimate Std. Error t value Pr(>|t|)
MD.H - MD.F == 0    6.971e-01  2.397e-01   2.908   0.0478 *
MD.I - MD.F == 0    8.978e-18  2.397e-01   0.000   1.0000
MD.Mix - MD.F == 0 -4.393e-19  2.397e-01   0.000   1.0000
MD.C - MD.F == 0   -1.776e-17  2.397e-01   0.000   1.0000
MD.I - MD.H == 0   -6.971e-01  2.397e-01  -2.908   0.0478 *
MD.Mix - MD.H == 0 -6.971e-01  2.397e-01  -2.908   0.0478 *
```

```
MD.C - MD.H == 0   -6.971e-01  2.397e-01  -2.908   0.0478 *
MD.Mix - MD.I == 0 -9.417e-18  2.397e-01   0.000   1.0000
MD.C - MD.I == 0   -2.674e-17  2.397e-01   0.000   1.0000
MD.C - MD.Mix == 0 -1.732e-17  2.397e-01   0.000   1.0000
---
Signif. codes:  0 ‘***’ 0.001 ‘**’ 0.01 ‘*’ 0.05 ‘.’ 0.1 ‘ ’ 1
(Adjusted p values reported -- bonferroni method)
```

ASV208

```
	 Simultaneous Tests for General Linear Hypotheses

Multiple Comparisons of Means: Tukey Contrasts


Fit: lm(formula = log_cop ~ Treatment, data = dsub)

Linear Hypotheses:
                   Estimate Std. Error t value Pr(>|t|)
MD.H - MD.F == 0   -1.791e-16  1.738e-01   0.000        1
MD.I - MD.F == 0   -3.593e-16  1.738e-01   0.000        1
MD.Mix - MD.F == 0 -3.569e-16  1.738e-01   0.000        1
MD.C - MD.F == 0    2.748e-01  1.738e-01   1.581        1
MD.I - MD.H == 0   -1.803e-16  1.738e-01   0.000        1
MD.Mix - MD.H == 0 -1.779e-16  1.738e-01   0.000        1
MD.C - MD.H == 0    2.748e-01  1.738e-01   1.581        1
```

```
MD.Mix - MD.I == 0  2.416e-18  1.738e-01   0.000        1
MD.C - MD.I == 0    2.748e-01  1.738e-01   1.581        1
MD.C - MD.Mix == 0  2.748e-01  1.738e-01   1.581        1
(Adjusted p values reported -- bonferroni method)
```

ASV209

```
	 Simultaneous Tests for General Linear Hypotheses

Multiple Comparisons of Means: Tukey Contrasts


Fit: lm(formula = log_cop ~ Treatment, data = dsub)

Linear Hypotheses:
                   Estimate Std. Error t value Pr(>|t|)
MD.H - MD.F == 0    3.555e-17  1.797e-01   0.000        1
MD.I - MD.F == 0    8.012e-17  1.797e-01   0.000        1
MD.Mix - MD.F == 0  2.842e-01  1.797e-01   1.581        1
MD.C - MD.F == 0   -3.557e-17  1.797e-01   0.000        1
MD.I - MD.H == 0    4.458e-17  1.797e-01   0.000        1
MD.Mix - MD.H == 0  2.842e-01  1.797e-01   1.581        1
MD.C - MD.H == 0   -7.112e-17  1.797e-01   0.000        1
MD.Mix - MD.I == 0  2.842e-01  1.797e-01   1.581        1
MD.C - MD.I == 0   -1.157e-16  1.797e-01   0.000        1
MD.C - MD.Mix == 0 -2.842e-01  1.797e-01  -1.581        1
```

```
(Adjusted p values reported -- bonferroni method)




ASV21

	 Simultaneous Tests for General Linear Hypotheses

Multiple Comparisons of Means: Tukey Contrasts


Fit: lm(formula = log_cop ~ Treatment, data = dsub)

Linear Hypotheses:
              Estimate Std. Error t value Pr(>|t|)
MD.H - MD.F == 0    1.301637   0.822585   1.582    1.000
MD.I - MD.F == 0   -0.003795   0.822585  -0.005    1.000
MD.Mix - MD.F == 0  2.154401   0.822585   2.619    0.107
MD.C - MD.F == 0    0.336757   0.822585   0.409    1.000
MD.I - MD.H == 0   -1.305432   0.822585  -1.587    1.000
MD.Mix - MD.H == 0  0.852764   0.822585   1.037    1.000
MD.C - MD.H == 0   -0.964880   0.822585  -1.173    1.000
MD.Mix - MD.I == 0  2.158195   0.822585   2.624    0.105
MD.C - MD.I == 0    0.340552   0.822585   0.414    1.000
MD.C - MD.Mix == 0 -1.817644   0.822585  -2.210    0.302
(Adjusted p values reported -- bonferroni method)
```

ASV212

Simultaneous Tests for General Linear Hypotheses

Multiple Comparisons of Means: Tukey Contrasts

Fit: lm(formula = log_cop ~ Treatment, data = dsub)

Linear Hypotheses:

```
                  Estimate Std. Error t value Pr(>|t|)
MD.H - MD.F == 0   -2.359e-01  1.492e-01  -1.581        1
MD.I - MD.F == 0   -2.359e-01  1.492e-01  -1.581        1
MD.Mix - MD.F == 0 -2.359e-01  1.492e-01  -1.581        1
MD.C - MD.F == 0   -2.359e-01  1.492e-01  -1.581        1
MD.I - MD.H == 0    2.776e-17  1.492e-01   0.000        1
MD.Mix - MD.H == 0 -2.498e-16  1.492e-01   0.000        1
MD.C - MD.H == 0    2.776e-17  1.492e-01   0.000        1
MD.Mix - MD.I == 0 -2.776e-16  1.492e-01   0.000        1
MD.C - MD.I == 0    0.000e+00  1.492e-01   0.000        1
MD.C - MD.Mix == 0  2.776e-16  1.492e-01   0.000        1
(Adjusted p values reported -- bonferroni method)
```

ASV219

```
	 Simultaneous Tests for General Linear Hypotheses

Multiple Comparisons of Means: Tukey Contrasts


Fit: lm(formula = log_cop ~ Treatment, data = dsub)

Linear Hypotheses:
               Estimate Std. Error t value Pr(>|t|)
MD.H - MD.F == 0    5.183e-01  2.975e-01   1.742    0.856
MD.I - MD.F == 0    1.282e-16  2.975e-01   0.000    1.000
MD.Mix - MD.F == 0  1.806e-01  2.975e-01   0.607    1.000
MD.C - MD.F == 0    2.350e-01  2.975e-01   0.790    1.000
MD.I - MD.H == 0   -5.183e-01  2.975e-01  -1.742    0.856
MD.Mix - MD.H == 0 -3.377e-01  2.975e-01  -1.135    1.000
MD.C - MD.H == 0   -2.834e-01  2.975e-01  -0.952    1.000
MD.Mix - MD.I == 0  1.806e-01  2.975e-01   0.607    1.000
MD.C - MD.I == 0    2.350e-01  2.975e-01   0.790    1.000
MD.C - MD.Mix == 0  5.432e-02  2.975e-01   0.183    1.000
(Adjusted p values reported -- bonferroni method)
```

ASV225

```
	 Simultaneous Tests for General Linear Hypotheses
```

```
Multiple Comparisons of Means: Tukey Contrasts


Fit: lm(formula = log_cop ~ Treatment, data = dsub)

Linear Hypotheses:
                      Estimate Std. Error t value Pr(>|t|)
MD.H - MD.F == 0     2.095e-16  2.044e-01   0.000        1
MD.I - MD.F == 0     1.856e-01  2.044e-01   0.908        1
MD.Mix - MD.F == 0   2.549e-16  2.044e-01   0.000        1
MD.C - MD.F == 0     2.645e-01  2.044e-01   1.294        1
MD.I - MD.H == 0     1.856e-01  2.044e-01   0.908        1
MD.Mix - MD.H == 0   4.549e-17  2.044e-01   0.000        1
MD.C - MD.H == 0     2.645e-01  2.044e-01   1.294        1
MD.Mix - MD.I == 0  -1.856e-01  2.044e-01  -0.908        1
MD.C - MD.I == 0     7.892e-02  2.044e-01   0.386        1
MD.C - MD.Mix == 0   2.645e-01  2.044e-01   1.294        1
(Adjusted p values reported -- bonferroni method)



ASV228

         Simultaneous Tests for General Linear Hypotheses

Multiple Comparisons of Means: Tukey Contrasts
```

```
Fit: lm(formula = log_cop ~ Treatment, data = dsub)

Linear Hypotheses:
                     Estimate Std. Error t value Pr(>|t|)
MD.H - MD.F == 0   -1.094e-18  1.341e-01   0.000        1
MD.I - MD.F == 0   -1.876e-16  1.341e-01   0.000        1
MD.Mix - MD.F == 0 -3.399e-17  1.341e-01   0.000        1
MD.C - MD.F == 0    2.121e-01  1.341e-01   1.581        1
MD.I - MD.H == 0   -1.865e-16  1.341e-01   0.000        1
MD.Mix - MD.H == 0 -3.290e-17  1.341e-01   0.000        1
MD.C - MD.H == 0    2.121e-01  1.341e-01   1.581        1
MD.Mix - MD.I == 0  1.536e-16  1.341e-01   0.000        1
MD.C - MD.I == 0    2.121e-01  1.341e-01   1.581        1
MD.C - MD.Mix == 0  2.121e-01  1.341e-01   1.581        1
(Adjusted p values reported -- bonferroni method)
```

ASV23

```
       Simultaneous Tests for General Linear Hypotheses

Multiple Comparisons of Means: Tukey Contrasts


Fit: lm(formula = log_cop ~ Treatment, data = dsub)

Linear Hypotheses:
```

```
                  Estimate Std. Error t value Pr(>|t|)
MD.H - MD.F == 0   -1.778e+00  5.382e-01  -3.304   0.0146 *
MD.I - MD.F == 0   -1.778e+00  5.382e-01  -3.304   0.0146 *
MD.Mix - MD.F == 0 -1.778e+00  5.382e-01  -3.304   0.0146 *
MD.C - MD.F == 0   -1.510e+00  5.382e-01  -2.807   0.0638 .
MD.I - MD.H == 0    6.661e-16  5.382e-01   0.000   1.0000
MD.Mix - MD.H == 0 -2.220e-16  5.382e-01   0.000   1.0000
MD.C - MD.H == 0    2.676e-01  5.382e-01   0.497   1.0000
MD.Mix - MD.I == 0 -8.882e-16  5.382e-01   0.000   1.0000
MD.C - MD.I == 0    2.676e-01  5.382e-01   0.497   1.0000
MD.C - MD.Mix == 0  2.676e-01  5.382e-01   0.497   1.0000
---
Signif. codes:  0 ‘***’ 0.001 ‘**’ 0.01 ‘*’ 0.05 ‘.’ 0.1 ‘ ’ 1
(Adjusted p values reported -- bonferroni method)
```

ASV230

```
       Simultaneous Tests for General Linear Hypotheses

Multiple Comparisons of Means: Tukey Contrasts


Fit: lm(formula = log_cop ~ Treatment, data = dsub)

Linear Hypotheses:
                  Estimate Std. Error t value Pr(>|t|)
```

```
MD.H - MD.F == 0   -7.758e-18  1.622e-01   0.000        1
MD.I - MD.F == 0    2.565e-01  1.622e-01   1.581        1
MD.Mix - MD.F == 0 -3.776e-17  1.622e-01   0.000        1
MD.C - MD.F == 0   -2.453e-17  1.622e-01   0.000        1
MD.I - MD.H == 0    2.565e-01  1.622e-01   1.581        1
MD.Mix - MD.H == 0 -3.000e-17  1.622e-01   0.000        1
MD.C - MD.H == 0   -1.677e-17  1.622e-01   0.000        1
MD.Mix - MD.I == 0 -2.565e-01  1.622e-01  -1.581        1
MD.C - MD.I == 0   -2.565e-01  1.622e-01  -1.581        1
MD.C - MD.Mix == 0  1.323e-17  1.622e-01   0.000        1
(Adjusted p values reported -- bonferroni method)
```

ASV232

```
	 Simultaneous Tests for General Linear Hypotheses

Multiple Comparisons of Means: Tukey Contrasts


Fit: lm(formula = log_cop ~ Treatment, data = dsub)

Linear Hypotheses:
                    Estimate Std. Error t value Pr(>|t|)
MD.H - MD.F == 0   -1.988e-01  1.766e-01  -1.125        1
MD.I - MD.F == 0   -1.988e-01  1.766e-01  -1.125        1
MD.Mix - MD.F == 0 -1.988e-01  1.766e-01  -1.125        1
```

```
MD.C - MD.F == 0   -2.632e-03  1.766e-01  -0.015        1
MD.I - MD.H == 0   -2.776e-17  1.766e-01   0.000        1
MD.Mix - MD.H == 0  5.551e-17  1.766e-01   0.000        1
MD.C - MD.H == 0    1.961e-01  1.766e-01   1.111        1
MD.Mix - MD.I == 0  8.327e-17  1.766e-01   0.000        1
MD.C - MD.I == 0    1.961e-01  1.766e-01   1.111        1
MD.C - MD.Mix == 0  1.961e-01  1.766e-01   1.111        1
(Adjusted p values reported -- bonferroni method)




ASV233


       Simultaneous Tests for General Linear Hypotheses


Multiple Comparisons of Means: Tukey Contrasts




Fit: lm(formula = log_cop ~ Treatment, data = dsub)


Linear Hypotheses:
               Estimate Std. Error t value Pr(>|t|)
MD.H - MD.F == 0    0.000e+00  1.342e-01   0.000        1
MD.I - MD.F == 0    2.122e-01  1.342e-01   1.581        1
MD.Mix - MD.F == 0  4.100e-17  1.342e-01   0.000        1
MD.C - MD.F == 0   -5.397e-17  1.342e-01   0.000        1
MD.I - MD.H == 0    2.122e-01  1.342e-01   1.581        1
MD.Mix - MD.H == 0  4.100e-17  1.342e-01   0.000        1
```

```
MD.C - MD.H == 0   -5.397e-17  1.342e-01   0.000        1
MD.Mix - MD.I == 0 -2.122e-01  1.342e-01  -1.581        1
MD.C - MD.I == 0   -2.122e-01  1.342e-01  -1.581        1
MD.C - MD.Mix == 0 -9.497e-17  1.342e-01   0.000        1
(Adjusted p values reported -- bonferroni method)
```

ASV238

```
       Simultaneous Tests for General Linear Hypotheses

Multiple Comparisons of Means: Tukey Contrasts


Fit: lm(formula = log_cop ~ Treatment, data = dsub)

Linear Hypotheses:
               Estimate Std. Error t value Pr(>|t|)
MD.H - MD.F == 0    6.206e-17  1.731e-01   0.000        1
MD.I - MD.F == 0    2.736e-01  1.731e-01   1.581        1
MD.Mix - MD.F == 0  1.188e-16  1.731e-01   0.000        1
MD.C - MD.F == 0   -3.435e-17  1.731e-01   0.000        1
MD.I - MD.H == 0    2.736e-01  1.731e-01   1.581        1
MD.Mix - MD.H == 0  5.674e-17  1.731e-01   0.000        1
MD.C - MD.H == 0   -9.641e-17  1.731e-01   0.000        1
MD.Mix - MD.I == 0 -2.736e-01  1.731e-01  -1.581        1
MD.C - MD.I == 0   -2.736e-01  1.731e-01  -1.581        1
```

```
MD.C - MD.Mix == 0 -1.532e-16  1.731e-01   0.000        1
(Adjusted p values reported -- bonferroni method)
```

ASV240

```
	 Simultaneous Tests for General Linear Hypotheses

Multiple Comparisons of Means: Tukey Contrasts


Fit: lm(formula = log_cop ~ Treatment, data = dsub)

Linear Hypotheses:
               Estimate Std. Error t value Pr(>|t|)
MD.H - MD.F == 0   -1.241e-15  1.676e-01   0.000        1
MD.I - MD.F == 0    2.650e-01  1.676e-01   1.581        1
MD.Mix - MD.F == 0 -3.935e-15  1.676e-01   0.000        1
MD.C - MD.F == 0   -2.551e-16  1.676e-01   0.000        1
MD.I - MD.H == 0    2.650e-01  1.676e-01   1.581        1
MD.Mix - MD.H == 0 -2.694e-15  1.676e-01   0.000        1
MD.C - MD.H == 0    9.861e-16  1.676e-01   0.000        1
MD.Mix - MD.I == 0 -2.650e-01  1.676e-01  -1.581        1
MD.C - MD.I == 0   -2.650e-01  1.676e-01  -1.581        1
MD.C - MD.Mix == 0  3.680e-15  1.676e-01   0.000        1
(Adjusted p values reported -- bonferroni method)
```

ASV242

```
	 Simultaneous Tests for General Linear Hypotheses

Multiple Comparisons of Means: Tukey Contrasts


Fit: lm(formula = log_cop ~ Treatment, data = dsub)

Linear Hypotheses:
               Estimate Std. Error t value Pr(>|t|)
MD.H - MD.F == 0   -2.212e-01  1.399e-01  -1.581        1
MD.I - MD.F == 0   -2.212e-01  1.399e-01  -1.581        1
MD.Mix - MD.F == 0 -2.212e-01  1.399e-01  -1.581        1
MD.C - MD.F == 0   -2.212e-01  1.399e-01  -1.581        1
MD.I - MD.H == 0    3.886e-16  1.399e-01   0.000        1
MD.Mix - MD.H == 0  8.327e-17  1.399e-01   0.000        1
MD.C - MD.H == 0    2.776e-16  1.399e-01   0.000        1
MD.Mix - MD.I == 0 -3.053e-16  1.399e-01   0.000        1
MD.C - MD.I == 0   -1.110e-16  1.399e-01   0.000        1
MD.C - MD.Mix == 0  1.943e-16  1.399e-01   0.000        1
(Adjusted p values reported -- bonferroni method)
```

ASV243

```
	 Simultaneous Tests for General Linear Hypotheses

Multiple Comparisons of Means: Tukey Contrasts


Fit: lm(formula = log_cop ~ Treatment, data = dsub)

Linear Hypotheses:
                   Estimate Std. Error t value Pr(>|t|)
MD.H - MD.F == 0   -1.314e-16  1.856e-01   0.000        1
MD.I - MD.F == 0    6.410e-17  1.856e-01   0.000        1
MD.Mix - MD.F == 0  1.700e-01  1.856e-01   0.916        1
MD.C - MD.F == 0    2.392e-01  1.856e-01   1.289        1
MD.I - MD.H == 0    1.955e-16  1.856e-01   0.000        1
MD.Mix - MD.H == 0  1.700e-01  1.856e-01   0.916        1
MD.C - MD.H == 0    2.392e-01  1.856e-01   1.289        1
MD.Mix - MD.I == 0  1.700e-01  1.856e-01   0.916        1
MD.C - MD.I == 0    2.392e-01  1.856e-01   1.289        1
MD.C - MD.Mix == 0  6.914e-02  1.856e-01   0.373        1
(Adjusted p values reported -- bonferroni method)
```

ASV244

```
	 Simultaneous Tests for General Linear Hypotheses
```

Multiple Comparisons of Means: Tukey Contrasts

Fit: lm(formula = log_cop ~ Treatment, data = dsub)

Linear Hypotheses:

```
                 Estimate Std. Error t value Pr(>|t|)
MD.H - MD.F == 0    1.099e-16  1.221e-01   0.000        1
MD.I - MD.F == 0    1.602e-16  1.221e-01   0.000        1
MD.Mix - MD.F == 0  1.930e-01  1.221e-01   1.581        1
MD.C - MD.F == 0   -1.533e-17  1.221e-01   0.000        1
MD.I - MD.H == 0    5.036e-17  1.221e-01   0.000        1
MD.Mix - MD.H == 0  1.930e-01  1.221e-01   1.581        1
MD.C - MD.H == 0   -1.252e-16  1.221e-01   0.000        1
MD.Mix - MD.I == 0  1.930e-01  1.221e-01   1.581        1
MD.C - MD.I == 0   -1.756e-16  1.221e-01   0.000        1
MD.C - MD.Mix == 0 -1.930e-01  1.221e-01  -1.581        1
(Adjusted p values reported -- bonferroni method)
```

ASV247

Simultaneous Tests for General Linear Hypotheses

Multiple Comparisons of Means: Tukey Contrasts

```
Fit: lm(formula = log_cop ~ Treatment, data = dsub)


Linear Hypotheses:
                     Estimate Std. Error t value Pr(>|t|)
MD.H - MD.F == 0    2.316e-01  1.465e-01   1.581        1
MD.I - MD.F == 0    4.950e-18  1.465e-01   0.000        1
MD.Mix - MD.F == 0 -7.627e-19  1.465e-01   0.000        1
MD.C - MD.F == 0   -5.394e-17  1.465e-01   0.000        1
MD.I - MD.H == 0   -2.316e-01  1.465e-01  -1.581        1
MD.Mix - MD.H == 0 -2.316e-01  1.465e-01  -1.581        1
MD.C - MD.H == 0   -2.316e-01  1.465e-01  -1.581        1
MD.Mix - MD.I == 0 -5.713e-18  1.465e-01   0.000        1
MD.C - MD.I == 0   -5.889e-17  1.465e-01   0.000        1
MD.C - MD.Mix == 0 -5.318e-17  1.465e-01   0.000        1
(Adjusted p values reported -- bonferroni method)
```

ASV25

```
	 Simultaneous Tests for General Linear Hypotheses

Multiple Comparisons of Means: Tukey Contrasts


Fit: lm(formula = log_cop ~ Treatment, data = dsub)
```

```
Linear Hypotheses:
                   Estimate Std. Error t value Pr(>|t|)
MD.H - MD.F == 0   -3.351e-17  1.491e-01   0.000        1
MD.I - MD.F == 0    1.763e-16  1.491e-01   0.000        1
MD.Mix - MD.F == 0  2.357e-01  1.491e-01   1.581        1
MD.C - MD.F == 0   -1.043e-17  1.491e-01   0.000        1
MD.I - MD.H == 0    2.098e-16  1.491e-01   0.000        1
MD.Mix - MD.H == 0  2.357e-01  1.491e-01   1.581        1
MD.C - MD.H == 0    2.308e-17  1.491e-01   0.000        1
MD.Mix - MD.I == 0  2.357e-01  1.491e-01   1.581        1
MD.C - MD.I == 0   -1.867e-16  1.491e-01   0.000        1
MD.C - MD.Mix == 0 -2.357e-01  1.491e-01  -1.581        1
(Adjusted p values reported -- bonferroni method)
```

ASV253

```
         Simultaneous Tests for General Linear Hypotheses

Multiple Comparisons of Means: Tukey Contrasts


Fit: lm(formula = log_cop ~ Treatment, data = dsub)

Linear Hypotheses:
                   Estimate Std. Error t value Pr(>|t|)
MD.H - MD.F == 0   -1.594e-16  1.194e-01   0.000        1
```

```
MD.I - MD.F == 0   -4.807e-17  1.194e-01   0.000        1
MD.Mix - MD.F == 0  1.888e-01  1.194e-01   1.581        1
MD.C - MD.F == 0   -6.746e-18  1.194e-01   0.000        1
MD.I - MD.H == 0    1.113e-16  1.194e-01   0.000        1
MD.Mix - MD.H == 0  1.888e-01  1.194e-01   1.581        1
MD.C - MD.H == 0    1.527e-16  1.194e-01   0.000        1
MD.Mix - MD.I == 0  1.888e-01  1.194e-01   1.581        1
MD.C - MD.I == 0    4.133e-17  1.194e-01   0.000        1
MD.C - MD.Mix == 0 -1.888e-01  1.194e-01  -1.581        1
(Adjusted p values reported -- bonferroni method)
```

ASV261

```
       Simultaneous Tests for General Linear Hypotheses

Multiple Comparisons of Means: Tukey Contrasts


Fit: lm(formula = log_cop ~ Treatment, data = dsub)

Linear Hypotheses:
                   Estimate Std. Error t value Pr(>|t|)
MD.H - MD.F == 0    1.752e-17  1.331e-01   0.000        1
MD.I - MD.F == 0    8.012e-18  1.331e-01   0.000        1
MD.Mix - MD.F == 0  2.105e-01  1.331e-01   1.581        1
MD.C - MD.F == 0   -1.472e-17  1.331e-01   0.000        1
```

```
MD.I - MD.H == 0   -9.507e-18  1.331e-01   0.000        1
MD.Mix - MD.H == 0  2.105e-01  1.331e-01   1.581        1
MD.C - MD.H == 0   -3.224e-17  1.331e-01   0.000        1
MD.Mix - MD.I == 0  2.105e-01  1.331e-01   1.581        1
MD.C - MD.I == 0   -2.273e-17  1.331e-01   0.000        1
MD.C - MD.Mix == 0 -2.105e-01  1.331e-01  -1.581        1
(Adjusted p values reported -- bonferroni method)
```

ASV263

```
       Simultaneous Tests for General Linear Hypotheses

Multiple Comparisons of Means: Tukey Contrasts


Fit: lm(formula = log_cop ~ Treatment, data = dsub)

Linear Hypotheses:
               Estimate Std. Error t value Pr(>|t|)
MD.H - MD.F == 0   -1.878e-01  1.995e-01  -0.941        1
MD.I - MD.F == 0   -1.878e-01  1.995e-01  -0.941        1
MD.Mix - MD.F == 0 -1.878e-01  1.995e-01  -0.941        1
MD.C - MD.F == 0    6.577e-02  1.995e-01   0.330        1
MD.I - MD.H == 0   -7.216e-16  1.995e-01   0.000        1
MD.Mix - MD.H == 0 -4.718e-16  1.995e-01   0.000        1
MD.C - MD.H == 0    2.535e-01  1.995e-01   1.271        1
```

```
MD.Mix - MD.I == 0  2.498e-16  1.995e-01   0.000        1
MD.C - MD.I == 0    2.535e-01  1.995e-01   1.271        1
MD.C - MD.Mix == 0  2.535e-01  1.995e-01   1.271        1
(Adjusted p values reported -- bonferroni method)
```

ASV264

```
	 Simultaneous Tests for General Linear Hypotheses

Multiple Comparisons of Means: Tukey Contrasts


Fit: lm(formula = log_cop ~ Treatment, data = dsub)

Linear Hypotheses:
               Estimate Std. Error t value Pr(>|t|)
MD.H - MD.F == 0    8.223e-16  1.619e-01   0.000        1
MD.I - MD.F == 0    2.559e-01  1.619e-01   1.581        1
MD.Mix - MD.F == 0  3.164e-15  1.619e-01   0.000        1
MD.C - MD.F == 0   -4.710e-16  1.619e-01   0.000        1
MD.I - MD.H == 0    2.559e-01  1.619e-01   1.581        1
MD.Mix - MD.H == 0  2.341e-15  1.619e-01   0.000        1
MD.C - MD.H == 0   -1.293e-15  1.619e-01   0.000        1
MD.Mix - MD.I == 0 -2.559e-01  1.619e-01  -1.581        1
MD.C - MD.I == 0   -2.559e-01  1.619e-01  -1.581        1
MD.C - MD.Mix == 0 -3.635e-15  1.619e-01   0.000        1
```

(Adjusted p values reported -- bonferroni method)

ASV265

Simultaneous Tests for General Linear Hypotheses

Multiple Comparisons of Means: Tukey Contrasts

Fit: lm(formula = log_cop ~ Treatment, data = dsub)

Linear Hypotheses:

	Estimate	Std. Error	t value	Pr(>|t|)
MD.H - MD.F == 0	1.286e-16	1.574e-01	0.000	1
MD.I - MD.F == 0	-6.490e-17	1.574e-01	0.000	1
MD.Mix - MD.F == 0	2.380e-16	1.574e-01	0.000	1
MD.C - MD.F == 0	2.488e-01	1.574e-01	1.581	1
MD.I - MD.H == 0	-1.935e-16	1.574e-01	0.000	1
MD.Mix - MD.H == 0	1.094e-16	1.574e-01	0.000	1
MD.C - MD.H == 0	2.488e-01	1.574e-01	1.581	1
MD.Mix - MD.I == 0	3.029e-16	1.574e-01	0.000	1
MD.C - MD.I == 0	2.488e-01	1.574e-01	1.581	1
MD.C - MD.Mix == 0	2.488e-01	1.574e-01	1.581	1

(Adjusted p values reported -- bonferroni method)

ASV266

```
	 Simultaneous Tests for General Linear Hypotheses

Multiple Comparisons of Means: Tukey Contrasts


Fit: lm(formula = log_cop ~ Treatment, data = dsub)

Linear Hypotheses:
                  Estimate Std. Error t value Pr(>|t|)
MD.H - MD.F == 0    1.286e-16  1.574e-01   0.000        1
MD.I - MD.F == 0   -6.490e-17  1.574e-01   0.000        1
MD.Mix - MD.F == 0  2.380e-16  1.574e-01   0.000        1
MD.C - MD.F == 0    2.488e-01  1.574e-01   1.581        1
MD.I - MD.H == 0   -1.935e-16  1.574e-01   0.000        1
MD.Mix - MD.H == 0  1.094e-16  1.574e-01   0.000        1
MD.C - MD.H == 0    2.488e-01  1.574e-01   1.581        1
MD.Mix - MD.I == 0  3.029e-16  1.574e-01   0.000        1
MD.C - MD.I == 0    2.488e-01  1.574e-01   1.581        1
MD.C - MD.Mix == 0  2.488e-01  1.574e-01   1.581        1
(Adjusted p values reported -- bonferroni method)
```

ASV268

```
         Simultaneous Tests for General Linear Hypotheses

Multiple Comparisons of Means: Tukey Contrasts


Fit: lm(formula = log_cop ~ Treatment, data = dsub)

Linear Hypotheses:
                   Estimate Std. Error t value Pr(>|t|)
MD.H - MD.F == 0    3.203e-16  1.758e-01   0.000        1
MD.I - MD.F == 0    4.006e-17  1.758e-01   0.000        1
MD.Mix - MD.F == 0  1.700e-01  1.758e-01   0.967        1
MD.C - MD.F == 0    2.199e-01  1.758e-01   1.251        1
MD.I - MD.H == 0   -2.803e-16  1.758e-01   0.000        1
MD.Mix - MD.H == 0  1.700e-01  1.758e-01   0.967        1
MD.C - MD.H == 0    2.199e-01  1.758e-01   1.251        1
MD.Mix - MD.I == 0  1.700e-01  1.758e-01   0.967        1
MD.C - MD.I == 0    2.199e-01  1.758e-01   1.251        1
MD.C - MD.Mix == 0  4.985e-02  1.758e-01   0.284        1
(Adjusted p values reported -- bonferroni method)



ASV271

         Simultaneous Tests for General Linear Hypotheses
```

Multiple Comparisons of Means: Tukey Contrasts

```
Fit: lm(formula = log_cop ~ Treatment, data = dsub)

Linear Hypotheses:
                     Estimate Std. Error t value Pr(>|t|)
MD.H - MD.F == 0    2.315e-01  1.464e-01   1.581        1
MD.I - MD.F == 0    8.435e-18  1.464e-01   0.000        1
MD.Mix - MD.F == 0  5.145e-18  1.464e-01   0.000        1
MD.C - MD.F == 0   -5.654e-17  1.464e-01   0.000        1
MD.I - MD.H == 0   -2.315e-01  1.464e-01  -1.581        1
MD.Mix - MD.H == 0 -2.315e-01  1.464e-01  -1.581        1
MD.C - MD.H == 0   -2.315e-01  1.464e-01  -1.581        1
MD.Mix - MD.I == 0 -3.290e-18  1.464e-01   0.000        1
MD.C - MD.I == 0   -6.498e-17  1.464e-01   0.000        1
MD.C - MD.Mix == 0 -6.169e-17  1.464e-01   0.000        1
(Adjusted p values reported -- bonferroni method)
```

ASV277

Simultaneous Tests for General Linear Hypotheses

Multiple Comparisons of Means: Tukey Contrasts

```
Fit: lm(formula = log_cop ~ Treatment, data = dsub)

Linear Hypotheses:
                    Estimate Std. Error t value Pr(>|t|)
MD.H - MD.F == 0    2.532e-01  1.602e-01   1.581        1
MD.I - MD.F == 0    2.329e-18  1.602e-01   0.000        1
MD.Mix - MD.F == 0  3.544e-19  1.602e-01   0.000        1
MD.C - MD.F == 0    1.639e-17  1.602e-01   0.000        1
MD.I - MD.H == 0   -2.532e-01  1.602e-01  -1.581        1
MD.Mix - MD.H == 0 -2.532e-01  1.602e-01  -1.581        1
MD.C - MD.H == 0   -2.532e-01  1.602e-01  -1.581        1
MD.Mix - MD.I == 0 -1.974e-18  1.602e-01   0.000        1
MD.C - MD.I == 0    1.406e-17  1.602e-01   0.000        1
MD.C - MD.Mix == 0  1.603e-17  1.602e-01   0.000        1
(Adjusted p values reported -- bonferroni method)
```

ASV279

```
	 Simultaneous Tests for General Linear Hypotheses

Multiple Comparisons of Means: Tukey Contrasts


Fit: lm(formula = log_cop ~ Treatment, data = dsub)

Linear Hypotheses:
```

```
                   Estimate Std. Error t value Pr(>|t|)
MD.H - MD.F == 0    7.593e-16  1.972e-01   0.000    1.000
MD.I - MD.F == 0    6.358e-16  1.972e-01   0.000    1.000
MD.Mix - MD.F == 0  8.498e-16  1.972e-01   0.000    1.000
MD.C - MD.F == 0    4.564e-01  1.972e-01   2.314    0.234
MD.I - MD.H == 0   -1.235e-16  1.972e-01   0.000    1.000
MD.Mix - MD.H == 0  9.053e-17  1.972e-01   0.000    1.000
MD.C - MD.H == 0    4.564e-01  1.972e-01   2.314    0.234
MD.Mix - MD.I == 0  2.140e-16  1.972e-01   0.000    1.000
MD.C - MD.I == 0    4.564e-01  1.972e-01   2.314    0.234
MD.C - MD.Mix == 0  4.564e-01  1.972e-01   2.314    0.234
(Adjusted p values reported -- bonferroni method)


ASV280

         Simultaneous Tests for General Linear Hypotheses

Multiple Comparisons of Means: Tukey Contrasts


Fit: lm(formula = log_cop ~ Treatment, data = dsub)

Linear Hypotheses:
                   Estimate Std. Error t value Pr(>|t|)
MD.H - MD.F == 0    3.438e-15  1.653e-01   0.000        1
MD.I - MD.F == 0   -3.949e-17  1.653e-01   0.000        1
```

```
MD.Mix - MD.F == 0  1.700e-16  1.653e-01   0.000        1
MD.C - MD.F == 0    2.613e-01  1.653e-01   1.581        1
MD.I - MD.H == 0   -3.478e-15  1.653e-01   0.000        1
MD.Mix - MD.H == 0 -3.268e-15  1.653e-01   0.000        1
MD.C - MD.H == 0    2.613e-01  1.653e-01   1.581        1
MD.Mix - MD.I == 0  2.095e-16  1.653e-01   0.000        1
MD.C - MD.I == 0    2.613e-01  1.653e-01   1.581        1
MD.C - MD.Mix == 0  2.613e-01  1.653e-01   1.581        1
(Adjusted p values reported -- bonferroni method)
```

ASV281

```
	 Simultaneous Tests for General Linear Hypotheses

Multiple Comparisons of Means: Tukey Contrasts


Fit: lm(formula = log_cop ~ Treatment, data = dsub)

Linear Hypotheses:
                   Estimate Std. Error t value Pr(>|t|)
MD.H - MD.F == 0    1.443e-15  1.415e-01   0.000        1
MD.I - MD.F == 0    2.237e-01  1.415e-01   1.581        1
MD.Mix - MD.F == 0  7.489e-16  1.415e-01   0.000        1
MD.C - MD.F == 0    6.820e-16  1.415e-01   0.000        1
MD.I - MD.H == 0    2.237e-01  1.415e-01   1.581        1
```

```
MD.Mix - MD.H == 0 -6.941e-16  1.415e-01   0.000        1
MD.C - MD.H == 0   -7.610e-16  1.415e-01   0.000        1
MD.Mix - MD.I == 0 -2.237e-01  1.415e-01  -1.581        1
MD.C - MD.I == 0   -2.237e-01  1.415e-01  -1.581        1
MD.C - MD.Mix == 0 -6.692e-17  1.415e-01   0.000        1
(Adjusted p values reported -- bonferroni method)
```

ASV282

```
	 Simultaneous Tests for General Linear Hypotheses

Multiple Comparisons of Means: Tukey Contrasts


Fit: lm(formula = log_cop ~ Treatment, data = dsub)

Linear Hypotheses:
               Estimate Std. Error t value Pr(>|t|)
MD.H - MD.F == 0    4.534e-17  1.755e-01   0.000        1
MD.I - MD.F == 0    3.365e-16  1.755e-01   0.000        1
MD.Mix - MD.F == 0  2.774e-01  1.755e-01   1.581        1
MD.C - MD.F == 0   -3.741e-17  1.755e-01   0.000        1
MD.I - MD.H == 0    2.912e-16  1.755e-01   0.000        1
MD.Mix - MD.H == 0  2.774e-01  1.755e-01   1.581        1
MD.C - MD.H == 0   -8.275e-17  1.755e-01   0.000        1
MD.Mix - MD.I == 0  2.774e-01  1.755e-01   1.581        1
```

```
MD.C - MD.I == 0   -3.739e-16  1.755e-01   0.000        1
MD.C - MD.Mix == 0 -2.774e-01  1.755e-01  -1.581        1
(Adjusted p values reported -- bonferroni method)
```

ASV284

```
	 Simultaneous Tests for General Linear Hypotheses

Multiple Comparisons of Means: Tukey Contrasts


Fit: lm(formula = log_cop ~ Treatment, data = dsub)

Linear Hypotheses:
               Estimate Std. Error t value Pr(>|t|)
MD.H - MD.F == 0   -3.181e-16  1.503e-01   0.000        1
MD.I - MD.F == 0    2.377e-01  1.503e-01   1.581        1
MD.Mix - MD.F == 0 -2.125e-16  1.503e-01   0.000        1
MD.C - MD.F == 0    0.000e+00  1.503e-01   0.000        1
MD.I - MD.H == 0    2.377e-01  1.503e-01   1.581        1
MD.Mix - MD.H == 0  1.056e-16  1.503e-01   0.000        1
MD.C - MD.H == 0    3.181e-16  1.503e-01   0.000        1
MD.Mix - MD.I == 0 -2.377e-01  1.503e-01  -1.581        1
MD.C - MD.I == 0   -2.377e-01  1.503e-01  -1.581        1
MD.C - MD.Mix == 0  2.125e-16  1.503e-01   0.000        1
(Adjusted p values reported -- bonferroni method)
```

ASV285

```
         Simultaneous Tests for General Linear Hypotheses

Multiple Comparisons of Means: Tukey Contrasts


Fit: lm(formula = log_cop ~ Treatment, data = dsub)

Linear Hypotheses:
               Estimate Std. Error t value Pr(>|t|)
MD.H - MD.F == 0   -2.003e-01  1.267e-01  -1.581        1
MD.I - MD.F == 0   -2.003e-01  1.267e-01  -1.581        1
MD.Mix - MD.F == 0 -2.003e-01  1.267e-01  -1.581        1
MD.C - MD.F == 0   -2.003e-01  1.267e-01  -1.581        1
MD.I - MD.H == 0    1.388e-16  1.267e-01   0.000        1
MD.Mix - MD.H == 0  2.776e-17  1.267e-01   0.000        1
MD.C - MD.H == 0    1.665e-16  1.267e-01   0.000        1
MD.Mix - MD.I == 0 -1.110e-16  1.267e-01   0.000        1
MD.C - MD.I == 0    2.776e-17  1.267e-01   0.000        1
MD.C - MD.Mix == 0  1.388e-16  1.267e-01   0.000        1
(Adjusted p values reported -- bonferroni method)
```

ASV288

```
	Simultaneous Tests for General Linear Hypotheses

Multiple Comparisons of Means: Tukey Contrasts


Fit: lm(formula = log_cop ~ Treatment, data = dsub)

Linear Hypotheses:
               Estimate Std. Error t value Pr(>|t|)
MD.H - MD.F == 0    1.724e-16  1.513e-01   0.000        1
MD.I - MD.F == 0    1.877e-17  1.513e-01   0.000        1
MD.Mix - MD.F == 0 -1.360e-16  1.513e-01   0.000        1
MD.C - MD.F == 0    2.392e-01  1.513e-01   1.581        1
MD.I - MD.H == 0   -1.536e-16  1.513e-01   0.000        1
MD.Mix - MD.H == 0 -3.084e-16  1.513e-01   0.000        1
MD.C - MD.H == 0    2.392e-01  1.513e-01   1.581        1
MD.Mix - MD.I == 0 -1.548e-16  1.513e-01   0.000        1
MD.C - MD.I == 0    2.392e-01  1.513e-01   1.581        1
MD.C - MD.Mix == 0  2.392e-01  1.513e-01   1.581        1
(Adjusted p values reported -- bonferroni method)
```

ASV291

```
        Simultaneous Tests for General Linear Hypotheses

Multiple Comparisons of Means: Tukey Contrasts


Fit: lm(formula = log_cop ~ Treatment, data = dsub)

Linear Hypotheses:
                      Estimate Std. Error t value Pr(>|t|)
MD.H - MD.F == 0     9.135e-17  1.125e-01   0.000        1
MD.I - MD.F == 0     2.404e-17  1.125e-01   0.000        1
MD.Mix - MD.F == 0  1.778e-01  1.125e-01   1.581        1
MD.C - MD.F == 0    -2.515e-17  1.125e-01   0.000        1
MD.I - MD.H == 0    -6.731e-17  1.125e-01   0.000        1
MD.Mix - MD.H == 0  1.778e-01  1.125e-01   1.581        1
MD.C - MD.H == 0    -1.165e-16  1.125e-01   0.000        1
MD.Mix - MD.I == 0  1.778e-01  1.125e-01   1.581        1
MD.C - MD.I == 0    -4.918e-17  1.125e-01   0.000        1
MD.C - MD.Mix == 0 -1.778e-01  1.125e-01  -1.581        1
(Adjusted p values reported -- bonferroni method)
```

ASV3

```
        Simultaneous Tests for General Linear Hypotheses

Multiple Comparisons of Means: Tukey Contrasts
```

```
Fit: lm(formula = log_cop ~ Treatment, data = dsub)

Linear Hypotheses:
             Estimate Std. Error t value Pr(>|t|)
MD.H - MD.F == 0    -1.3489     1.0033  -1.345   1.0000
MD.I - MD.F == 0     0.9135     1.0033   0.910   1.0000
MD.Mix - MD.F == 0  -2.0129     1.0033  -2.006   0.4843
MD.C - MD.F == 0    -0.4261     1.0033  -0.425   1.0000
MD.I - MD.H == 0     2.2624     1.0033   2.255   0.2705
MD.Mix - MD.H == 0  -0.6640     1.0033  -0.662   1.0000
MD.C - MD.H == 0     0.9229     1.0033   0.920   1.0000
MD.Mix - MD.I == 0  -2.9264     1.0033  -2.917   0.0466 *
MD.C - MD.I == 0    -1.3396     1.0033  -1.335   1.0000
MD.C - MD.Mix == 0   1.5869     1.0033   1.582   1.0000
---
Signif. codes:  0 ‘***’ 0.001 ‘**’ 0.01 ‘*’ 0.05 ‘.’ 0.1 ‘ ’ 1
(Adjusted p values reported -- bonferroni method)
```

ASV30

```
	 Simultaneous Tests for General Linear Hypotheses

Multiple Comparisons of Means: Tukey Contrasts
```

```
Fit: lm(formula = log_cop ~ Treatment, data = dsub)

Linear Hypotheses:
                  Estimate Std. Error t value Pr(>|t|)
MD.H - MD.F == 0    2.015e-01  1.274e-01   1.581        1
MD.I - MD.F == 0   -2.985e-18  1.274e-01   0.000        1
MD.Mix - MD.F == 0 -2.483e-18  1.274e-01   0.000        1
MD.C - MD.F == 0    7.425e-18  1.274e-01   0.000        1
MD.I - MD.H == 0   -2.015e-01  1.274e-01  -1.581        1
MD.Mix - MD.H == 0 -2.015e-01  1.274e-01  -1.581        1
MD.C - MD.H == 0   -2.015e-01  1.274e-01  -1.581        1
MD.Mix - MD.I == 0  5.015e-19  1.274e-01   0.000        1
MD.C - MD.I == 0    1.041e-17  1.274e-01   0.000        1
MD.C - MD.Mix == 0  9.908e-18  1.274e-01   0.000        1
(Adjusted p values reported -- bonferroni method)
```

ASV303

```
	 Simultaneous Tests for General Linear Hypotheses

Multiple Comparisons of Means: Tukey Contrasts


Fit: lm(formula = log_cop ~ Treatment, data = dsub)
```

```
Linear Hypotheses:
                  Estimate Std. Error t value Pr(>|t|)
MD.H - MD.F == 0    2.396e-01  1.515e-01   1.581        1
MD.I - MD.F == 0    2.934e-18  1.515e-01   0.000        1
MD.Mix - MD.F == 0  5.823e-18  1.515e-01   0.000        1
MD.C - MD.F == 0    1.500e-17  1.515e-01   0.000        1
MD.I - MD.H == 0   -2.396e-01  1.515e-01  -1.581        1
MD.Mix - MD.H == 0 -2.396e-01  1.515e-01  -1.581        1
MD.C - MD.H == 0   -2.396e-01  1.515e-01  -1.581        1
MD.Mix - MD.I == 0  2.889e-18  1.515e-01   0.000        1
MD.C - MD.I == 0    1.206e-17  1.515e-01   0.000        1
MD.C - MD.Mix == 0  9.176e-18  1.515e-01   0.000        1
(Adjusted p values reported -- bonferroni method)
```

ASV304

```
         Simultaneous Tests for General Linear Hypotheses

Multiple Comparisons of Means: Tukey Contrasts


Fit: lm(formula = log_cop ~ Treatment, data = dsub)

Linear Hypotheses:
                  Estimate Std. Error t value Pr(>|t|)
MD.H - MD.F == 0    3.504e-17  1.640e-01   0.000        1
```

```
MD.I - MD.F == 0    1.602e-17  1.640e-01   0.000        1
MD.Mix - MD.F == 0  2.593e-01  1.640e-01   1.581        1
MD.C - MD.F == 0   -3.741e-17  1.640e-01   0.000        1
MD.I - MD.H == 0   -1.901e-17  1.640e-01   0.000        1
MD.Mix - MD.H == 0  2.593e-01  1.640e-01   1.581        1
MD.C - MD.H == 0   -7.245e-17  1.640e-01   0.000        1
MD.Mix - MD.I == 0  2.593e-01  1.640e-01   1.581        1
MD.C - MD.I == 0   -5.344e-17  1.640e-01   0.000        1
MD.C - MD.Mix == 0 -2.593e-01  1.640e-01  -1.581        1
(Adjusted p values reported -- bonferroni method)
```

ASV307

```
       Simultaneous Tests for General Linear Hypotheses

Multiple Comparisons of Means: Tukey Contrasts


Fit: lm(formula = log_cop ~ Treatment, data = dsub)

Linear Hypotheses:
               Estimate Std. Error t value Pr(>|t|)
MD.H - MD.F == 0    2.649e-01  1.675e-01   1.581        1
MD.I - MD.F == 0   -1.906e-18  1.675e-01   0.000        1
MD.Mix - MD.F == 0 -6.091e-18  1.675e-01   0.000        1
MD.C - MD.F == 0    7.133e-18  1.675e-01   0.000        1
```

```
MD.I - MD.H == 0   -2.649e-01  1.675e-01  -1.581        1
MD.Mix - MD.H == 0 -2.649e-01  1.675e-01  -1.581        1
MD.C - MD.H == 0   -2.649e-01  1.675e-01  -1.581        1
MD.Mix - MD.I == 0 -4.185e-18  1.675e-01   0.000        1
MD.C - MD.I == 0    9.039e-18  1.675e-01   0.000        1
MD.C - MD.Mix == 0  1.322e-17  1.675e-01   0.000        1
(Adjusted p values reported -- bonferroni method)
```

ASV308

```
       Simultaneous Tests for General Linear Hypotheses

Multiple Comparisons of Means: Tukey Contrasts


Fit: lm(formula = log_cop ~ Treatment, data = dsub)

Linear Hypotheses:
               Estimate Std. Error t value Pr(>|t|)
MD.H - MD.F == 0    1.992e-16  1.705e-01   0.000        1
MD.I - MD.F == 0    1.763e-16  1.705e-01   0.000        1
MD.Mix - MD.F == 0  2.696e-01  1.705e-01   1.581        1
MD.C - MD.F == 0   -6.072e-17  1.705e-01   0.000        1
MD.I - MD.H == 0   -2.295e-17  1.705e-01   0.000        1
MD.Mix - MD.H == 0  2.696e-01  1.705e-01   1.581        1
MD.C - MD.H == 0   -2.599e-16  1.705e-01   0.000        1
```

```
MD.Mix - MD.I == 0  2.696e-01  1.705e-01   1.581        1
MD.C - MD.I == 0   -2.370e-16  1.705e-01   0.000        1
MD.C - MD.Mix == 0 -2.696e-01  1.705e-01  -1.581        1
(Adjusted p values reported -- bonferroni method)
```

ASV31

```
         Simultaneous Tests for General Linear Hypotheses

Multiple Comparisons of Means: Tukey Contrasts


Fit: lm(formula = log_cop ~ Treatment, data = dsub)

Linear Hypotheses:
                    Estimate Std. Error t value Pr(>|t|)
MD.H - MD.F == 0   -1.982e-01  1.254e-01  -1.581        1
MD.I - MD.F == 0   -1.982e-01  1.254e-01  -1.581        1
MD.Mix - MD.F == 0 -1.982e-01  1.254e-01  -1.581        1
MD.C - MD.F == 0   -1.982e-01  1.254e-01  -1.581        1
MD.I - MD.H == 0    3.331e-16  1.254e-01   0.000        1
MD.Mix - MD.H == 0  1.388e-16  1.254e-01   0.000        1
MD.C - MD.H == 0    3.053e-16  1.254e-01   0.000        1
MD.Mix - MD.I == 0 -1.943e-16  1.254e-01   0.000        1
MD.C - MD.I == 0   -2.776e-17  1.254e-01   0.000        1
MD.C - MD.Mix == 0  1.665e-16  1.254e-01   0.000        1
```

```
(Adjusted p values reported -- bonferroni method)
```

ASV310

```
	 Simultaneous Tests for General Linear Hypotheses

Multiple Comparisons of Means: Tukey Contrasts


Fit: lm(formula = log_cop ~ Treatment, data = dsub)

Linear Hypotheses:
               Estimate Std. Error t value Pr(>|t|)
MD.H - MD.F == 0    2.125e-01  1.344e-01   1.581        1
MD.I - MD.F == 0   -4.497e-18  1.344e-01   0.000        1
MD.Mix - MD.F == 0  4.415e-19  1.344e-01   0.000        1
MD.C - MD.F == 0    2.852e-17  1.344e-01   0.000        1
MD.I - MD.H == 0   -2.125e-01  1.344e-01  -1.581        1
MD.Mix - MD.H == 0 -2.125e-01  1.344e-01  -1.581        1
MD.C - MD.H == 0   -2.125e-01  1.344e-01  -1.581        1
MD.Mix - MD.I == 0  4.939e-18  1.344e-01   0.000        1
MD.C - MD.I == 0    3.302e-17  1.344e-01   0.000        1
MD.C - MD.Mix == 0  2.808e-17  1.344e-01   0.000        1
(Adjusted p values reported -- bonferroni method)
```

ASV311

```
	Simultaneous Tests for General Linear Hypotheses

Multiple Comparisons of Means: Tukey Contrasts


Fit: lm(formula = log_cop ~ Treatment, data = dsub)

Linear Hypotheses:
               Estimate Std. Error t value Pr(>|t|)
MD.H - MD.F == 0    6.334e-17  1.602e-01   0.000        1
MD.I - MD.F == 0    1.602e-16  1.602e-01   0.000        1
MD.Mix - MD.F == 0  2.533e-01  1.602e-01   1.581        1
MD.C - MD.F == 0   -2.269e-17  1.602e-01   0.000        1
MD.I - MD.H == 0    9.691e-17  1.602e-01   0.000        1
MD.Mix - MD.H == 0  2.533e-01  1.602e-01   1.581        1
MD.C - MD.H == 0   -8.603e-17  1.602e-01   0.000        1
MD.Mix - MD.I == 0  2.533e-01  1.602e-01   1.581        1
MD.C - MD.I == 0   -1.829e-16  1.602e-01   0.000        1
MD.C - MD.Mix == 0 -2.533e-01  1.602e-01  -1.581        1
(Adjusted p values reported -- bonferroni method)
```

ASV313

```
	 Simultaneous Tests for General Linear Hypotheses

Multiple Comparisons of Means: Tukey Contrasts


Fit: lm(formula = log_cop ~ Treatment, data = dsub)

Linear Hypotheses:
               Estimate Std. Error t value Pr(>|t|)
MD.H - MD.F == 0   -2.256e-01  1.427e-01  -1.581        1
MD.I - MD.F == 0   -2.256e-01  1.427e-01  -1.581        1
MD.Mix - MD.F == 0 -2.256e-01  1.427e-01  -1.581        1
MD.C - MD.F == 0   -2.256e-01  1.427e-01  -1.581        1
MD.I - MD.H == 0    8.327e-17  1.427e-01   0.000        1
MD.Mix - MD.H == 0 -3.331e-16  1.427e-01   0.000        1
MD.C - MD.H == 0    2.776e-17  1.427e-01   0.000        1
MD.Mix - MD.I == 0 -4.163e-16  1.427e-01   0.000        1
MD.C - MD.I == 0   -5.551e-17  1.427e-01   0.000        1
MD.C - MD.Mix == 0  3.608e-16  1.427e-01   0.000        1
(Adjusted p values reported -- bonferroni method)
```

ASV314

```
	 Simultaneous Tests for General Linear Hypotheses
```

```
Multiple Comparisons of Means: Tukey Contrasts


Fit: lm(formula = log_cop ~ Treatment, data = dsub)

Linear Hypotheses:
                   Estimate Std. Error t value Pr(>|t|)
MD.H - MD.F == 0   -1.988e-01  1.257e-01  -1.581        1
MD.I - MD.F == 0   -1.988e-01  1.257e-01  -1.581        1
MD.Mix - MD.F == 0 -1.988e-01  1.257e-01  -1.581        1
MD.C - MD.F == 0   -1.988e-01  1.257e-01  -1.581        1
MD.I - MD.H == 0    1.665e-16  1.257e-01   0.000        1
MD.Mix - MD.H == 0  1.665e-16  1.257e-01   0.000        1
MD.C - MD.H == 0   -2.776e-17  1.257e-01   0.000        1
MD.Mix - MD.I == 0  0.000e+00  1.257e-01   0.000        1
MD.C - MD.I == 0   -1.943e-16  1.257e-01   0.000        1
MD.C - MD.Mix == 0 -1.943e-16  1.257e-01   0.000        1
(Adjusted p values reported -- bonferroni method)
```

ASV315

```
	 Simultaneous Tests for General Linear Hypotheses

Multiple Comparisons of Means: Tukey Contrasts
```

```
Fit: lm(formula = log_cop ~ Treatment, data = dsub)

Linear Hypotheses:
                   Estimate Std. Error t value Pr(>|t|)
MD.H - MD.F == 0    6.731e-17  1.486e-01   0.000        1
MD.I - MD.F == 0   -4.364e-16  1.486e-01   0.000        1
MD.Mix - MD.F == 0  8.498e-17  1.486e-01   0.000        1
MD.C - MD.F == 0    2.350e-01  1.486e-01   1.581        1
MD.I - MD.H == 0   -5.037e-16  1.486e-01   0.000        1
MD.Mix - MD.H == 0  1.768e-17  1.486e-01   0.000        1
MD.C - MD.H == 0    2.350e-01  1.486e-01   1.581        1
MD.Mix - MD.I == 0  5.214e-16  1.486e-01   0.000        1
MD.C - MD.I == 0    2.350e-01  1.486e-01   1.581        1
MD.C - MD.Mix == 0  2.350e-01  1.486e-01   1.581        1
(Adjusted p values reported -- bonferroni method)
```

ASV316

```
         Simultaneous Tests for General Linear Hypotheses

Multiple Comparisons of Means: Tukey Contrasts


Fit: lm(formula = log_cop ~ Treatment, data = dsub)

Linear Hypotheses:
```

```
                Estimate Std. Error t value Pr(>|t|)
MD.H - MD.F == 0    6.731e-17  1.486e-01   0.000        1
MD.I - MD.F == 0   -4.364e-16  1.486e-01   0.000        1
MD.Mix - MD.F == 0  8.498e-17  1.486e-01   0.000        1
MD.C - MD.F == 0    2.350e-01  1.486e-01   1.581        1
MD.I - MD.H == 0   -5.037e-16  1.486e-01   0.000        1
MD.Mix - MD.H == 0  1.768e-17  1.486e-01   0.000        1
MD.C - MD.H == 0    2.350e-01  1.486e-01   1.581        1
MD.Mix - MD.I == 0  5.214e-16  1.486e-01   0.000        1
MD.C - MD.I == 0    2.350e-01  1.486e-01   1.581        1
MD.C - MD.Mix == 0  2.350e-01  1.486e-01   1.581        1
(Adjusted p values reported -- bonferroni method)
```

ASV317

```
       Simultaneous Tests for General Linear Hypotheses

Multiple Comparisons of Means: Tukey Contrasts


Fit: lm(formula = log_cop ~ Treatment, data = dsub)

Linear Hypotheses:
                Estimate Std. Error t value Pr(>|t|)
MD.H - MD.F == 0    6.731e-17  1.486e-01   0.000        1
MD.I - MD.F == 0   -4.364e-16  1.486e-01   0.000        1
```

```
MD.Mix - MD.F == 0  8.498e-17  1.486e-01   0.000        1
MD.C - MD.F == 0    2.350e-01  1.486e-01   1.581        1
MD.I - MD.H == 0   -5.037e-16  1.486e-01   0.000        1
MD.Mix - MD.H == 0  1.768e-17  1.486e-01   0.000        1
MD.C - MD.H == 0    2.350e-01  1.486e-01   1.581        1
MD.Mix - MD.I == 0  5.214e-16  1.486e-01   0.000        1
MD.C - MD.I == 0    2.350e-01  1.486e-01   1.581        1
MD.C - MD.Mix == 0  2.350e-01  1.486e-01   1.581        1
(Adjusted p values reported -- bonferroni method)
```

ASV318

```
	 Simultaneous Tests for General Linear Hypotheses

Multiple Comparisons of Means: Tukey Contrasts


Fit: lm(formula = log_cop ~ Treatment, data = dsub)

Linear Hypotheses:
               Estimate Std. Error t value Pr(>|t|)
MD.H - MD.F == 0   -5.431e-17  1.174e-01   0.000        1
MD.I - MD.F == 0    1.856e-01  1.174e-01   1.581        1
MD.Mix - MD.F == 0 -6.264e-17  1.174e-01   0.000        1
MD.C - MD.F == 0   -2.331e-17  1.174e-01   0.000        1
MD.I - MD.H == 0    1.856e-01  1.174e-01   1.581        1
```

```
MD.Mix - MD.H == 0 -8.338e-18  1.174e-01   0.000        1
MD.C - MD.H == 0    3.100e-17  1.174e-01   0.000        1
MD.Mix - MD.I == 0 -1.856e-01  1.174e-01  -1.581        1
MD.C - MD.I == 0   -1.856e-01  1.174e-01  -1.581        1
MD.C - MD.Mix == 0  3.934e-17  1.174e-01   0.000        1
(Adjusted p values reported -- bonferroni method)
```

ASV319

```
	 Simultaneous Tests for General Linear Hypotheses

Multiple Comparisons of Means: Tukey Contrasts


Fit: lm(formula = log_cop ~ Treatment, data = dsub)

Linear Hypotheses:
               Estimate Std. Error t value Pr(>|t|)
MD.H - MD.F == 0   -5.682e-17  1.075e-01   0.000        1
MD.I - MD.F == 0   -7.211e-17  1.075e-01   0.000        1
MD.Mix - MD.F == 0  1.700e-01  1.075e-01   1.581        1
MD.C - MD.F == 0   -3.067e-19  1.075e-01   0.000        1
MD.I - MD.H == 0   -1.529e-17  1.075e-01   0.000        1
MD.Mix - MD.H == 0  1.700e-01  1.075e-01   1.581        1
MD.C - MD.H == 0    5.651e-17  1.075e-01   0.000        1
MD.Mix - MD.I == 0  1.700e-01  1.075e-01   1.581        1
```

```
MD.C - MD.I == 0    7.180e-17  1.075e-01   0.000        1
MD.C - MD.Mix == 0 -1.700e-01  1.075e-01  -1.581        1
(Adjusted p values reported -- bonferroni method)


ASV322

	 Simultaneous Tests for General Linear Hypotheses

Multiple Comparisons of Means: Tukey Contrasts


Fit: lm(formula = log_cop ~ Treatment, data = dsub)

Linear Hypotheses:
                     Estimate Std. Error t value Pr(>|t|)
MD.H - MD.F == 0   -2.375e-01  1.502e-01  -1.581        1
MD.I - MD.F == 0   -2.375e-01  1.502e-01  -1.581        1
MD.Mix - MD.F == 0 -2.375e-01  1.502e-01  -1.581        1
MD.C - MD.F == 0   -2.375e-01  1.502e-01  -1.581        1
MD.I - MD.H == 0    1.943e-16  1.502e-01   0.000        1
MD.Mix - MD.H == 0  1.943e-16  1.502e-01   0.000        1
MD.C - MD.H == 0    2.498e-16  1.502e-01   0.000        1
MD.Mix - MD.I == 0  0.000e+00  1.502e-01   0.000        1
MD.C - MD.I == 0    5.551e-17  1.502e-01   0.000        1
MD.C - MD.Mix == 0  5.551e-17  1.502e-01   0.000        1
(Adjusted p values reported -- bonferroni method)
```

ASV323

```
	 Simultaneous Tests for General Linear Hypotheses

Multiple Comparisons of Means: Tukey Contrasts


Fit: lm(formula = log_cop ~ Treatment, data = dsub)

Linear Hypotheses:
                      Estimate Std. Error t value Pr(>|t|)
MD.H - MD.F == 0    -1.086e-16  1.376e-01   0.000        1
MD.I - MD.F == 0     2.176e-01  1.376e-01   1.581        1
MD.Mix - MD.F == 0 -4.924e-17  1.376e-01   0.000        1
MD.C - MD.F == 0    -1.349e-17  1.376e-01   0.000        1
MD.I - MD.H == 0     2.176e-01  1.376e-01   1.581        1
MD.Mix - MD.H == 0  5.937e-17  1.376e-01   0.000        1
MD.C - MD.H == 0     9.512e-17  1.376e-01   0.000        1
MD.Mix - MD.I == 0 -2.176e-01  1.376e-01  -1.581        1
MD.C - MD.I == 0    -2.176e-01  1.376e-01  -1.581        1
MD.C - MD.Mix == 0  3.575e-17  1.376e-01   0.000        1
(Adjusted p values reported -- bonferroni method)
```

ASV324

```
	 Simultaneous Tests for General Linear Hypotheses

Multiple Comparisons of Means: Tukey Contrasts


Fit: lm(formula = log_cop ~ Treatment, data = dsub)

Linear Hypotheses:
               Estimate Std. Error t value Pr(>|t|)
MD.H - MD.F == 0    7.503e-18  1.716e-01   0.000        1
MD.I - MD.F == 0   -3.205e-17  1.716e-01   0.000        1
MD.Mix - MD.F == 0  2.713e-01  1.716e-01   1.581        1
MD.C - MD.F == 0   -1.165e-17  1.716e-01   0.000        1
MD.I - MD.H == 0   -3.955e-17  1.716e-01   0.000        1
MD.Mix - MD.H == 0  2.713e-01  1.716e-01   1.581        1
MD.C - MD.H == 0   -1.916e-17  1.716e-01   0.000        1
MD.Mix - MD.I == 0  2.713e-01  1.716e-01   1.581        1
MD.C - MD.I == 0    2.040e-17  1.716e-01   0.000        1
MD.C - MD.Mix == 0 -2.713e-01  1.716e-01  -1.581        1
(Adjusted p values reported -- bonferroni method)
```

ASV337

```
	 Simultaneous Tests for General Linear Hypotheses

Multiple Comparisons of Means: Tukey Contrasts


Fit: lm(formula = log_cop ~ Treatment, data = dsub)

Linear Hypotheses:
                   Estimate Std. Error t value Pr(>|t|)
MD.H - MD.F == 0    2.327e-17  1.532e-01   0.000        1
MD.I - MD.F == 0    2.423e-01  1.532e-01   1.581        1
MD.Mix - MD.F == 0  1.191e-17  1.532e-01   0.000        1
MD.C - MD.F == 0   -4.416e-17  1.532e-01   0.000        1
MD.I - MD.H == 0    2.423e-01  1.532e-01   1.581        1
MD.Mix - MD.H == 0 -1.136e-17  1.532e-01   0.000        1
MD.C - MD.H == 0   -6.743e-17  1.532e-01   0.000        1
MD.Mix - MD.I == 0 -2.423e-01  1.532e-01  -1.581        1
MD.C - MD.I == 0   -2.423e-01  1.532e-01  -1.581        1
MD.C - MD.Mix == 0 -5.607e-17  1.532e-01   0.000        1
(Adjusted p values reported -- bonferroni method)



ASV338

	 Simultaneous Tests for General Linear Hypotheses

Multiple Comparisons of Means: Tukey Contrasts
```

```
Fit: lm(formula = log_cop ~ Treatment, data = dsub)

Linear Hypotheses:
                    Estimate Std. Error t value Pr(>|t|)
MD.H - MD.F == 0   -2.728e-17  1.590e-01   0.000        1
MD.I - MD.F == 0   -1.602e-17  1.590e-01   0.000        1
MD.Mix - MD.F == 0  2.515e-01  1.590e-01   1.581        1
MD.C - MD.F == 0   -9.813e-18  1.590e-01   0.000        1
MD.I - MD.H == 0    1.126e-17  1.590e-01   0.000        1
MD.Mix - MD.H == 0  2.515e-01  1.590e-01   1.581        1
MD.C - MD.H == 0    1.747e-17  1.590e-01   0.000        1
MD.Mix - MD.I == 0  2.515e-01  1.590e-01   1.581        1
MD.C - MD.I == 0    6.212e-18  1.590e-01   0.000        1
MD.C - MD.Mix == 0 -2.515e-01  1.590e-01  -1.581        1
(Adjusted p values reported -- bonferroni method)
```

ASV339

```
         Simultaneous Tests for General Linear Hypotheses

Multiple Comparisons of Means: Tukey Contrasts

Fit: lm(formula = log_cop ~ Treatment, data = dsub)
```

```
Linear Hypotheses:
                      Estimate Std. Error t value Pr(>|t|)
MD.H - MD.F == 0    7.596e-17  1.299e-01   0.000        1
MD.I - MD.F == 0   -1.786e-16  1.299e-01   0.000        1
MD.Mix - MD.F == 0  1.700e-17  1.299e-01   0.000        1
MD.C - MD.F == 0    2.054e-01  1.299e-01   1.581        1
MD.I - MD.H == 0   -2.546e-16  1.299e-01   0.000        1
MD.Mix - MD.H == 0 -5.897e-17  1.299e-01   0.000        1
MD.C - MD.H == 0    2.054e-01  1.299e-01   1.581        1
MD.Mix - MD.I == 0  1.956e-16  1.299e-01   0.000        1
MD.C - MD.I == 0    2.054e-01  1.299e-01   1.581        1
MD.C - MD.Mix == 0  2.054e-01  1.299e-01   1.581        1
(Adjusted p values reported -- bonferroni method)
```

ASV340

```
         Simultaneous Tests for General Linear Hypotheses

Multiple Comparisons of Means: Tukey Contrasts


Fit: lm(formula = log_cop ~ Treatment, data = dsub)

Linear Hypotheses:
                      Estimate Std. Error t value Pr(>|t|)
```

```
MD.H - MD.F == 0    7.596e-17  1.299e-01   0.000        1
MD.I - MD.F == 0   -1.786e-16  1.299e-01   0.000        1
MD.Mix - MD.F == 0  1.700e-17  1.299e-01   0.000        1
MD.C - MD.F == 0    2.054e-01  1.299e-01   1.581        1
MD.I - MD.H == 0   -2.546e-16  1.299e-01   0.000        1
MD.Mix - MD.H == 0 -5.897e-17  1.299e-01   0.000        1
MD.C - MD.H == 0    2.054e-01  1.299e-01   1.581        1
MD.Mix - MD.I == 0  1.956e-16  1.299e-01   0.000        1
MD.C - MD.I == 0    2.054e-01  1.299e-01   1.581        1
MD.C - MD.Mix == 0  2.054e-01  1.299e-01   1.581        1
(Adjusted p values reported -- bonferroni method)
```

ASV341

```
	 Simultaneous Tests for General Linear Hypotheses

Multiple Comparisons of Means: Tukey Contrasts


Fit: lm(formula = log_cop ~ Treatment, data = dsub)

Linear Hypotheses:
               Estimate Std. Error t value Pr(>|t|)
MD.H - MD.F == 0    7.596e-17  1.299e-01   0.000        1
MD.I - MD.F == 0   -1.786e-16  1.299e-01   0.000        1
MD.Mix - MD.F == 0  1.700e-17  1.299e-01   0.000        1
```

```
MD.C - MD.F == 0    2.054e-01  1.299e-01   1.581        1
MD.I - MD.H == 0   -2.546e-16  1.299e-01   0.000        1
MD.Mix - MD.H == 0 -5.897e-17  1.299e-01   0.000        1
MD.C - MD.H == 0    2.054e-01  1.299e-01   1.581        1
MD.Mix - MD.I == 0  1.956e-16  1.299e-01   0.000        1
MD.C - MD.I == 0    2.054e-01  1.299e-01   1.581        1
MD.C - MD.Mix == 0  2.054e-01  1.299e-01   1.581        1
(Adjusted p values reported -- bonferroni method)
```

ASV345

```
       Simultaneous Tests for General Linear Hypotheses

Multiple Comparisons of Means: Tukey Contrasts


Fit: lm(formula = log_cop ~ Treatment, data = dsub)

Linear Hypotheses:
               Estimate Std. Error t value Pr(>|t|)
MD.H - MD.F == 0    2.728e-17  1.212e-01   0.000        1
MD.I - MD.F == 0    1.602e-17  1.212e-01   0.000        1
MD.Mix - MD.F == 0  1.916e-01  1.212e-01   1.581        1
MD.C - MD.F == 0   -1.717e-17  1.212e-01   0.000        1
MD.I - MD.H == 0   -1.126e-17  1.212e-01   0.000        1
MD.Mix - MD.H == 0  1.916e-01  1.212e-01   1.581        1
```

```
MD.C - MD.H == 0   -4.445e-17  1.212e-01   0.000        1
MD.Mix - MD.I == 0  1.916e-01  1.212e-01   1.581        1
MD.C - MD.I == 0   -3.320e-17  1.212e-01   0.000        1
MD.C - MD.Mix == 0 -1.916e-01  1.212e-01  -1.581        1
(Adjusted p values reported -- bonferroni method)




ASV347

       Simultaneous Tests for General Linear Hypotheses

Multiple Comparisons of Means: Tukey Contrasts


Fit: lm(formula = log_cop ~ Treatment, data = dsub)

Linear Hypotheses:
               Estimate Std. Error t value Pr(>|t|)
MD.H - MD.F == 0    6.982e-17  1.296e-01   0.000        1
MD.I - MD.F == 0    2.048e-01  1.296e-01   1.581        1
MD.Mix - MD.F == 0  1.493e-16  1.296e-01   0.000        1
MD.C - MD.F == 0    7.065e-16  1.296e-01   0.000        1
MD.I - MD.H == 0    2.048e-01  1.296e-01   1.581        1
MD.Mix - MD.H == 0  7.949e-17  1.296e-01   0.000        1
MD.C - MD.H == 0    6.367e-16  1.296e-01   0.000        1
MD.Mix - MD.I == 0 -2.048e-01  1.296e-01  -1.581        1
MD.C - MD.I == 0   -2.048e-01  1.296e-01  -1.581        1
```

```
MD.C - MD.Mix == 0  5.572e-16  1.296e-01   0.000        1
(Adjusted p values reported -- bonferroni method)
```

ASV351

```
	 Simultaneous Tests for General Linear Hypotheses

Multiple Comparisons of Means: Tukey Contrasts


Fit: lm(formula = log_cop ~ Treatment, data = dsub)

Linear Hypotheses:
                   Estimate Std. Error t value Pr(>|t|)
MD.H - MD.F == 0   -4.655e-17  1.384e-01   0.000        1
MD.I - MD.F == 0    2.189e-01  1.384e-01   1.581        1
MD.Mix - MD.F == 0  1.407e-16  1.384e-01   0.000        1
MD.C - MD.F == 0   -2.453e-17  1.384e-01   0.000        1
MD.I - MD.H == 0    2.189e-01  1.384e-01   1.581        1
MD.Mix - MD.H == 0  1.873e-16  1.384e-01   0.000        1
MD.C - MD.H == 0    2.201e-17  1.384e-01   0.000        1
MD.Mix - MD.I == 0 -2.189e-01  1.384e-01  -1.581        1
MD.C - MD.I == 0   -2.189e-01  1.384e-01  -1.581        1
MD.C - MD.Mix == 0 -1.652e-16  1.384e-01   0.000        1
(Adjusted p values reported -- bonferroni method)
```

ASV352

```
	 Simultaneous Tests for General Linear Hypotheses

Multiple Comparisons of Means: Tukey Contrasts


Fit: lm(formula = log_cop ~ Treatment, data = dsub)

Linear Hypotheses:
               Estimate Std. Error t value Pr(>|t|)
MD.H - MD.F == 0   -4.655e-17  1.384e-01   0.000        1
MD.I - MD.F == 0    2.189e-01  1.384e-01   1.581        1
MD.Mix - MD.F == 0  1.407e-16  1.384e-01   0.000        1
MD.C - MD.F == 0   -2.453e-17  1.384e-01   0.000        1
MD.I - MD.H == 0    2.189e-01  1.384e-01   1.581        1
MD.Mix - MD.H == 0  1.873e-16  1.384e-01   0.000        1
MD.C - MD.H == 0    2.201e-17  1.384e-01   0.000        1
MD.Mix - MD.I == 0 -2.189e-01  1.384e-01  -1.581        1
MD.C - MD.I == 0   -2.189e-01  1.384e-01  -1.581        1
MD.C - MD.Mix == 0 -1.652e-16  1.384e-01   0.000        1
(Adjusted p values reported -- bonferroni method)
```

ASV358

```
       Simultaneous Tests for General Linear Hypotheses

Multiple Comparisons of Means: Tukey Contrasts


Fit: lm(formula = log_cop ~ Treatment, data = dsub)

Linear Hypotheses:
               Estimate Std. Error t value Pr(>|t|)
MD.H - MD.F == 0   -1.872e-01  1.184e-01  -1.581        1
MD.I - MD.F == 0   -1.872e-01  1.184e-01  -1.581        1
MD.Mix - MD.F == 0 -1.872e-01  1.184e-01  -1.581        1
MD.C - MD.F == 0   -1.872e-01  1.184e-01  -1.581        1
MD.I - MD.H == 0    0.000e+00  1.184e-01   0.000        1
MD.Mix - MD.H == 0  0.000e+00  1.184e-01   0.000        1
MD.C - MD.H == 0   -5.551e-17  1.184e-01   0.000        1
MD.Mix - MD.I == 0  0.000e+00  1.184e-01   0.000        1
MD.C - MD.I == 0   -5.551e-17  1.184e-01   0.000        1
MD.C - MD.Mix == 0 -5.551e-17  1.184e-01   0.000        1
(Adjusted p values reported -- bonferroni method)
```

ASV360

```
       Simultaneous Tests for General Linear Hypotheses
```

```
Multiple Comparisons of Means: Tukey Contrasts


Fit: lm(formula = log_cop ~ Treatment, data = dsub)

Linear Hypotheses:
                 Estimate Std. Error t value Pr(>|t|)
MD.H - MD.F == 0   -2.146e-01  1.357e-01  -1.581        1
MD.I - MD.F == 0   -2.146e-01  1.357e-01  -1.581        1
MD.Mix - MD.F == 0 -2.146e-01  1.357e-01  -1.581        1
MD.C - MD.F == 0   -2.146e-01  1.357e-01  -1.581        1
MD.I - MD.H == 0    1.110e-16  1.357e-01   0.000        1
MD.Mix - MD.H == 0 -5.551e-17  1.357e-01   0.000        1
MD.C - MD.H == 0   -5.551e-17  1.357e-01   0.000        1
MD.Mix - MD.I == 0 -1.665e-16  1.357e-01   0.000        1
MD.C - MD.I == 0   -1.665e-16  1.357e-01   0.000        1
MD.C - MD.Mix == 0  0.000e+00  1.357e-01   0.000        1
(Adjusted p values reported -- bonferroni method)



ASV363

       Simultaneous Tests for General Linear Hypotheses

Multiple Comparisons of Means: Tukey Contrasts
```

```
Fit: lm(formula = log_cop ~ Treatment, data = dsub)

Linear Hypotheses:
                    Estimate Std. Error t value Pr(>|t|)
MD.H - MD.F == 0    2.528e-16  1.394e-01   0.000        1
MD.I - MD.F == 0   -1.808e-16  1.394e-01   0.000        1
MD.Mix - MD.F == 0  3.229e-16  1.394e-01   0.000        1
MD.C - MD.F == 0    2.203e-01  1.394e-01   1.581        1
MD.I - MD.H == 0   -4.336e-16  1.394e-01   0.000        1
MD.Mix - MD.H == 0  7.018e-17  1.394e-01   0.000        1
MD.C - MD.H == 0    2.203e-01  1.394e-01   1.581        1
MD.Mix - MD.I == 0  5.037e-16  1.394e-01   0.000        1
MD.C - MD.I == 0    2.203e-01  1.394e-01   1.581        1
MD.C - MD.Mix == 0  2.203e-01  1.394e-01   1.581        1
(Adjusted p values reported -- bonferroni method)
```

ASV364

```
         Simultaneous Tests for General Linear Hypotheses

Multiple Comparisons of Means: Tukey Contrasts


Fit: lm(formula = log_cop ~ Treatment, data = dsub)
```

```
Linear Hypotheses:
                    Estimate Std. Error t value Pr(>|t|)
MD.H - MD.F == 0    2.528e-16  1.394e-01   0.000        1
MD.I - MD.F == 0   -1.808e-16  1.394e-01   0.000        1
MD.Mix - MD.F == 0  3.229e-16  1.394e-01   0.000        1
MD.C - MD.F == 0    2.203e-01  1.394e-01   1.581        1
MD.I - MD.H == 0   -4.336e-16  1.394e-01   0.000        1
MD.Mix - MD.H == 0  7.018e-17  1.394e-01   0.000        1
MD.C - MD.H == 0    2.203e-01  1.394e-01   1.581        1
MD.Mix - MD.I == 0  5.037e-16  1.394e-01   0.000        1
MD.C - MD.I == 0    2.203e-01  1.394e-01   1.581        1
MD.C - MD.Mix == 0  2.203e-01  1.394e-01   1.581        1
(Adjusted p values reported -- bonferroni method)
```

ASV366

```
         Simultaneous Tests for General Linear Hypotheses

Multiple Comparisons of Means: Tukey Contrasts


Fit: lm(formula = log_cop ~ Treatment, data = dsub)

Linear Hypotheses:
                    Estimate Std. Error t value Pr(>|t|)
MD.H - MD.F == 0    1.936e-01  1.225e-01   1.581        1
```

```
MD.I - MD.F == 0    3.538e-18  1.225e-01   0.000        1
MD.Mix - MD.F == 0 -3.489e-19  1.225e-01   0.000        1
MD.C - MD.F == 0   -9.055e-17  1.225e-01   0.000        1
MD.I - MD.H == 0   -1.936e-01  1.225e-01  -1.581        1
MD.Mix - MD.H == 0 -1.936e-01  1.225e-01  -1.581        1
MD.C - MD.H == 0   -1.936e-01  1.225e-01  -1.581        1
MD.Mix - MD.I == 0 -3.887e-18  1.225e-01   0.000        1
MD.C - MD.I == 0   -9.409e-17  1.225e-01   0.000        1
MD.C - MD.Mix == 0 -9.020e-17  1.225e-01   0.000        1
(Adjusted p values reported -- bonferroni method)
```

ASV367

```
	 Simultaneous Tests for General Linear Hypotheses

Multiple Comparisons of Means: Tukey Contrasts


Fit: lm(formula = log_cop ~ Treatment, data = dsub)

Linear Hypotheses:
               Estimate Std. Error t value Pr(>|t|)
MD.H - MD.F == 0    8.220e-17  1.416e-01   0.000        1
MD.I - MD.F == 0   -6.638e-18  1.416e-01   0.000        1
MD.Mix - MD.F == 0 -6.799e-17  1.416e-01   0.000        1
MD.C - MD.F == 0    2.239e-01  1.416e-01   1.581        1
```

```
MD.I - MD.H == 0   -8.883e-17  1.416e-01   0.000        1
MD.Mix - MD.H == 0 -1.502e-16  1.416e-01   0.000        1
MD.C - MD.H == 0    2.239e-01  1.416e-01   1.581        1
MD.Mix - MD.I == 0 -6.135e-17  1.416e-01   0.000        1
MD.C - MD.I == 0    2.239e-01  1.416e-01   1.581        1
MD.C - MD.Mix == 0  2.239e-01  1.416e-01   1.581        1
(Adjusted p values reported -- bonferroni method)
```

ASV368

```
	 Simultaneous Tests for General Linear Hypotheses

Multiple Comparisons of Means: Tukey Contrasts


Fit: lm(formula = log_cop ~ Treatment, data = dsub)

Linear Hypotheses:
               Estimate Std. Error t value Pr(>|t|)
MD.H - MD.F == 0    8.220e-17  1.416e-01   0.000        1
MD.I - MD.F == 0   -6.638e-18  1.416e-01   0.000        1
MD.Mix - MD.F == 0 -6.799e-17  1.416e-01   0.000        1
MD.C - MD.F == 0    2.239e-01  1.416e-01   1.581        1
MD.I - MD.H == 0   -8.883e-17  1.416e-01   0.000        1
MD.Mix - MD.H == 0 -1.502e-16  1.416e-01   0.000        1
MD.C - MD.H == 0    2.239e-01  1.416e-01   1.581        1
```

```
MD.Mix - MD.I == 0 -6.135e-17  1.416e-01   0.000        1
MD.C - MD.I == 0    2.239e-01  1.416e-01   1.581        1
MD.C - MD.Mix == 0  2.239e-01  1.416e-01   1.581        1
(Adjusted p values reported -- bonferroni method)
```

ASV369

```
	 Simultaneous Tests for General Linear Hypotheses

Multiple Comparisons of Means: Tukey Contrasts


Fit: lm(formula = log_cop ~ Treatment, data = dsub)

Linear Hypotheses:
               Estimate Std. Error t value Pr(>|t|)
MD.H - MD.F == 0    4.229e-17  1.006e-01   0.000        1
MD.I - MD.F == 0   -4.807e-17  1.006e-01   0.000        1
MD.Mix - MD.F == 0  1.590e-01  1.006e-01   1.581        1
MD.C - MD.F == 0   -1.993e-17  1.006e-01   0.000        1
MD.I - MD.H == 0   -9.036e-17  1.006e-01   0.000        1
MD.Mix - MD.H == 0  1.590e-01  1.006e-01   1.581        1
MD.C - MD.H == 0   -6.222e-17  1.006e-01   0.000        1
MD.Mix - MD.I == 0  1.590e-01  1.006e-01   1.581        1
MD.C - MD.I == 0    2.814e-17  1.006e-01   0.000        1
MD.C - MD.Mix == 0 -1.590e-01  1.006e-01  -1.581        1
```

```
(Adjusted p values reported -- bonferroni method)

ASV370

       Simultaneous Tests for General Linear Hypotheses

Multiple Comparisons of Means: Tukey Contrasts


Fit: lm(formula = log_cop ~ Treatment, data = dsub)

Linear Hypotheses:
                    Estimate Std. Error t value Pr(>|t|)
MD.H - MD.F == 0    4.229e-17  1.006e-01   0.000        1
MD.I - MD.F == 0   -4.807e-17  1.006e-01   0.000        1
MD.Mix - MD.F == 0  1.590e-01  1.006e-01   1.581        1
MD.C - MD.F == 0   -1.993e-17  1.006e-01   0.000        1
MD.I - MD.H == 0   -9.036e-17  1.006e-01   0.000        1
MD.Mix - MD.H == 0  1.590e-01  1.006e-01   1.581        1
MD.C - MD.H == 0   -6.222e-17  1.006e-01   0.000        1
MD.Mix - MD.I == 0  1.590e-01  1.006e-01   1.581        1
MD.C - MD.I == 0    2.814e-17  1.006e-01   0.000        1
MD.C - MD.Mix == 0 -1.590e-01  1.006e-01  -1.581        1
(Adjusted p values reported -- bonferroni method)
```

ASV371

```
	 Simultaneous Tests for General Linear Hypotheses

Multiple Comparisons of Means: Tukey Contrasts


Fit: lm(formula = log_cop ~ Treatment, data = dsub)

Linear Hypotheses:
               Estimate Std. Error t value Pr(>|t|)
MD.H - MD.F == 0    4.229e-17  1.006e-01   0.000        1
MD.I - MD.F == 0   -4.807e-17  1.006e-01   0.000        1
MD.Mix - MD.F == 0  1.590e-01  1.006e-01   1.581        1
MD.C - MD.F == 0   -1.993e-17  1.006e-01   0.000        1
MD.I - MD.H == 0   -9.036e-17  1.006e-01   0.000        1
MD.Mix - MD.H == 0  1.590e-01  1.006e-01   1.581        1
MD.C - MD.H == 0   -6.222e-17  1.006e-01   0.000        1
MD.Mix - MD.I == 0  1.590e-01  1.006e-01   1.581        1
MD.C - MD.I == 0    2.814e-17  1.006e-01   0.000        1
MD.C - MD.Mix == 0 -1.590e-01  1.006e-01  -1.581        1
(Adjusted p values reported -- bonferroni method)
```

ASV374

```
	 Simultaneous Tests for General Linear Hypotheses

Multiple Comparisons of Means: Tukey Contrasts


Fit: lm(formula = log_cop ~ Treatment, data = dsub)

Linear Hypotheses:
                   Estimate Std. Error t value Pr(>|t|)
MD.H - MD.F == 0    2.067e-01  1.307e-01   1.581        1
MD.I - MD.F == 0   -3.760e-19  1.307e-01   0.000        1
MD.Mix - MD.F == 0  4.292e-18  1.307e-01   0.000        1
MD.C - MD.F == 0    9.044e-17  1.307e-01   0.000        1
MD.I - MD.H == 0   -2.067e-01  1.307e-01  -1.581        1
MD.Mix - MD.H == 0 -2.067e-01  1.307e-01  -1.581        1
MD.C - MD.H == 0   -2.067e-01  1.307e-01  -1.581        1
MD.Mix - MD.I == 0  4.668e-18  1.307e-01   0.000        1
MD.C - MD.I == 0    9.081e-17  1.307e-01   0.000        1
MD.C - MD.Mix == 0  8.614e-17  1.307e-01   0.000        1
(Adjusted p values reported -- bonferroni method)
```

ASV378

```
	 Simultaneous Tests for General Linear Hypotheses
```

```
Multiple Comparisons of Means: Tukey Contrasts


Fit: lm(formula = log_cop ~ Treatment, data = dsub)

Linear Hypotheses:
                    Estimate Std. Error t value Pr(>|t|)
MD.H - MD.F == 0   -8.209e-17  1.354e-01   0.000        1
MD.I - MD.F == 0   -8.012e-17  1.354e-01   0.000        1
MD.Mix - MD.F == 0  2.141e-01  1.354e-01   1.581        1
MD.C - MD.F == 0    3.067e-18  1.354e-01   0.000        1
MD.I - MD.H == 0    1.971e-18  1.354e-01   0.000        1
MD.Mix - MD.H == 0  2.141e-01  1.354e-01   1.581        1
MD.C - MD.H == 0    8.516e-17  1.354e-01   0.000        1
MD.Mix - MD.I == 0  2.141e-01  1.354e-01   1.581        1
MD.C - MD.I == 0    8.319e-17  1.354e-01   0.000        1
MD.C - MD.Mix == 0 -2.141e-01  1.354e-01  -1.581        1
(Adjusted p values reported -- bonferroni method)




ASV38

         Simultaneous Tests for General Linear Hypotheses

Multiple Comparisons of Means: Tukey Contrasts
```

```
Fit: lm(formula = log_cop ~ Treatment, data = dsub)

Linear Hypotheses:
                    Estimate Std. Error t value Pr(>|t|)
MD.H - MD.F == 0   -3.404e-01  5.667e-01  -0.601 1.000000
MD.I - MD.F == 0   -3.404e-01  5.667e-01  -0.601 1.000000
MD.Mix - MD.F == 0  2.128e+00  5.667e-01   3.754 0.003410 **
MD.C - MD.F == 0   -3.404e-01  5.667e-01  -0.601 1.000000
MD.I - MD.H == 0    9.437e-16  5.667e-01   0.000 1.000000
MD.Mix - MD.H == 0  2.468e+00  5.667e-01   4.355 0.000416 ***
MD.C - MD.H == 0    2.220e-16  5.667e-01   0.000 1.000000
MD.Mix - MD.I == 0  2.468e+00  5.667e-01   4.355 0.000416 ***
MD.C - MD.I == 0   -7.216e-16  5.667e-01   0.000 1.000000
MD.C - MD.Mix == 0 -2.468e+00  5.667e-01  -4.355 0.000416 ***
---
Signif. codes:  0 ‘***’ 0.001 ‘**’ 0.01 ‘*’ 0.05 ‘.’ 0.1 ‘ ’ 1
(Adjusted p values reported -- bonferroni method)
```

ASV380

```
         Simultaneous Tests for General Linear Hypotheses

Multiple Comparisons of Means: Tukey Contrasts


Fit: lm(formula = log_cop ~ Treatment, data = dsub)
```

```
Linear Hypotheses:
               Estimate Std. Error t value Pr(>|t|)
MD.H - MD.F == 0    1.095e-17  1.563e-01   0.000        1
MD.I - MD.F == 0   -2.857e-16  1.563e-01   0.000        1
MD.Mix - MD.F == 0 -1.360e-16  1.563e-01   0.000        1
MD.C - MD.F == 0    2.471e-01  1.563e-01   1.581        1
MD.I - MD.H == 0   -2.966e-16  1.563e-01   0.000        1
MD.Mix - MD.H == 0 -1.469e-16  1.563e-01   0.000        1
MD.C - MD.H == 0    2.471e-01  1.563e-01   1.581        1
MD.Mix - MD.I == 0  1.497e-16  1.563e-01   0.000        1
MD.C - MD.I == 0    2.471e-01  1.563e-01   1.581        1
MD.C - MD.Mix == 0  2.471e-01  1.563e-01   1.581        1
(Adjusted p values reported -- bonferroni method)
```

ASV381

```
	 Simultaneous Tests for General Linear Hypotheses

Multiple Comparisons of Means: Tukey Contrasts


Fit: lm(formula = log_cop ~ Treatment, data = dsub)

Linear Hypotheses:
               Estimate Std. Error t value Pr(>|t|)
```

```
MD.H - MD.F == 0    1.552e-17  1.096e-01   0.000        1
MD.I - MD.F == 0    1.733e-01  1.096e-01   1.581        1
MD.Mix - MD.F == 0 -6.098e-17  1.096e-01   0.000        1
MD.C - MD.F == 0   -5.397e-17  1.096e-01   0.000        1
MD.I - MD.H == 0    1.733e-01  1.096e-01   1.581        1
MD.Mix - MD.H == 0 -7.649e-17  1.096e-01   0.000        1
MD.C - MD.H == 0   -6.949e-17  1.096e-01   0.000        1
MD.Mix - MD.I == 0 -1.733e-01  1.096e-01  -1.581        1
MD.C - MD.I == 0   -1.733e-01  1.096e-01  -1.581        1
MD.C - MD.Mix == 0  7.008e-18  1.096e-01   0.000        1
(Adjusted p values reported -- bonferroni method)
```

ASV382

```
	 Simultaneous Tests for General Linear Hypotheses

Multiple Comparisons of Means: Tukey Contrasts


Fit: lm(formula = log_cop ~ Treatment, data = dsub)

Linear Hypotheses:
                    Estimate Std. Error t value Pr(>|t|)
MD.H - MD.F == 0    1.552e-17  1.096e-01   0.000        1
MD.I - MD.F == 0    1.733e-01  1.096e-01   1.581        1
MD.Mix - MD.F == 0 -6.098e-17  1.096e-01   0.000        1
```

```
MD.C - MD.F == 0   -5.397e-17  1.096e-01   0.000        1
MD.I - MD.H == 0    1.733e-01  1.096e-01   1.581        1
MD.Mix - MD.H == 0 -7.649e-17  1.096e-01   0.000        1
MD.C - MD.H == 0   -6.949e-17  1.096e-01   0.000        1
MD.Mix - MD.I == 0 -1.733e-01  1.096e-01  -1.581        1
MD.C - MD.I == 0   -1.733e-01  1.096e-01  -1.581        1
MD.C - MD.Mix == 0  7.008e-18  1.096e-01   0.000        1
(Adjusted p values reported -- bonferroni method)
```

ASV383

```
	 Simultaneous Tests for General Linear Hypotheses

Multiple Comparisons of Means: Tukey Contrasts


Fit: lm(formula = log_cop ~ Treatment, data = dsub)

Linear Hypotheses:
                   Estimate Std. Error t value Pr(>|t|)
MD.H - MD.F == 0   -2.340e-01  1.480e-01  -1.581        1
MD.I - MD.F == 0   -2.340e-01  1.480e-01  -1.581        1
MD.Mix - MD.F == 0 -2.340e-01  1.480e-01  -1.581        1
MD.C - MD.F == 0   -2.340e-01  1.480e-01  -1.581        1
MD.I - MD.H == 0    1.110e-16  1.480e-01   0.000        1
MD.Mix - MD.H == 0 -2.776e-17  1.480e-01   0.000        1
```

```
MD.C - MD.H == 0    8.327e-17  1.480e-01   0.000        1
MD.Mix - MD.I == 0 -1.388e-16  1.480e-01   0.000        1
MD.C - MD.I == 0   -2.776e-17  1.480e-01   0.000        1
MD.C - MD.Mix == 0  1.110e-16  1.480e-01   0.000        1
(Adjusted p values reported -- bonferroni method)
```

ASV384

```
       Simultaneous Tests for General Linear Hypotheses

Multiple Comparisons of Means: Tukey Contrasts


Fit: lm(formula = log_cop ~ Treatment, data = dsub)

Linear Hypotheses:
               Estimate Std. Error t value Pr(>|t|)
MD.H - MD.F == 0   -2.340e-01  1.480e-01  -1.581        1
MD.I - MD.F == 0   -2.340e-01  1.480e-01  -1.581        1
MD.Mix - MD.F == 0 -2.340e-01  1.480e-01  -1.581        1
MD.C - MD.F == 0   -2.340e-01  1.480e-01  -1.581        1
MD.I - MD.H == 0    1.110e-16  1.480e-01   0.000        1
MD.Mix - MD.H == 0 -2.776e-17  1.480e-01   0.000        1
MD.C - MD.H == 0    8.327e-17  1.480e-01   0.000        1
MD.Mix - MD.I == 0 -1.388e-16  1.480e-01   0.000        1
MD.C - MD.I == 0   -2.776e-17  1.480e-01   0.000        1
```

```
MD.C - MD.Mix == 0  1.110e-16  1.480e-01   0.000        1
(Adjusted p values reported -- bonferroni method)
```

ASV386

```
       Simultaneous Tests for General Linear Hypotheses

Multiple Comparisons of Means: Tukey Contrasts


Fit: lm(formula = log_cop ~ Treatment, data = dsub)

Linear Hypotheses:
                  Estimate Std. Error t value Pr(>|t|)
MD.H - MD.F == 0    5.431e-17  1.344e-01   0.000        1
MD.I - MD.F == 0    2.125e-01  1.344e-01   1.581        1
MD.Mix - MD.F == 0  2.054e-16  1.344e-01   0.000        1
MD.C - MD.F == 0   -6.501e-17  1.344e-01   0.000        1
MD.I - MD.H == 0    2.125e-01  1.344e-01   1.581        1
MD.Mix - MD.H == 0  1.511e-16  1.344e-01   0.000        1
MD.C - MD.H == 0   -1.193e-16  1.344e-01   0.000        1
MD.Mix - MD.I == 0 -2.125e-01  1.344e-01  -1.581        1
MD.C - MD.I == 0   -2.125e-01  1.344e-01  -1.581        1
MD.C - MD.Mix == 0 -2.705e-16  1.344e-01   0.000        1
(Adjusted p values reported -- bonferroni method)
```

ASV387

```
	 Simultaneous Tests for General Linear Hypotheses

Multiple Comparisons of Means: Tukey Contrasts


Fit: lm(formula = log_cop ~ Treatment, data = dsub)

Linear Hypotheses:
                   Estimate Std. Error t value Pr(>|t|)
MD.H - MD.F == 0   -2.559e-01  1.618e-01  -1.581        1
MD.I - MD.F == 0   -2.559e-01  1.618e-01  -1.581        1
MD.Mix - MD.F == 0 -2.559e-01  1.618e-01  -1.581        1
MD.C - MD.F == 0   -2.559e-01  1.618e-01  -1.581        1
MD.I - MD.H == 0    1.665e-16  1.618e-01   0.000        1
MD.Mix - MD.H == 0 -5.551e-17  1.618e-01   0.000        1
MD.C - MD.H == 0    5.551e-17  1.618e-01   0.000        1
MD.Mix - MD.I == 0 -2.220e-16  1.618e-01   0.000        1
MD.C - MD.I == 0   -1.110e-16  1.618e-01   0.000        1
MD.C - MD.Mix == 0  1.110e-16  1.618e-01   0.000        1
(Adjusted p values reported -- bonferroni method)
```

ASV388

```
	 Simultaneous Tests for General Linear Hypotheses

Multiple Comparisons of Means: Tukey Contrasts


Fit: lm(formula = log_cop ~ Treatment, data = dsub)

Linear Hypotheses:
                  Estimate Std. Error t value Pr(>|t|)
MD.H - MD.F == 0   -3.378e-17  1.229e-01   0.000        1
MD.I - MD.F == 0   -1.772e-16  1.229e-01   0.000        1
MD.Mix - MD.F == 0 -5.099e-17  1.229e-01   0.000        1
MD.C - MD.F == 0    1.943e-01  1.229e-01   1.581        1
MD.I - MD.H == 0   -1.435e-16  1.229e-01   0.000        1
MD.Mix - MD.H == 0 -1.721e-17  1.229e-01   0.000        1
MD.C - MD.H == 0    1.943e-01  1.229e-01   1.581        1
MD.Mix - MD.I == 0  1.263e-16  1.229e-01   0.000        1
MD.C - MD.I == 0    1.943e-01  1.229e-01   1.581        1
MD.C - MD.Mix == 0  1.943e-01  1.229e-01   1.581        1
(Adjusted p values reported -- bonferroni method)
```

ASV389

```
	 Simultaneous Tests for General Linear Hypotheses
```

```
Multiple Comparisons of Means: Tukey Contrasts


Fit: lm(formula = log_cop ~ Treatment, data = dsub)

Linear Hypotheses:
                      Estimate Std. Error t value Pr(>|t|)
MD.H - MD.F == 0    3.803e-17  1.083e-01   0.000        1
MD.I - MD.F == 0   -9.615e-17  1.083e-01   0.000        1
MD.Mix - MD.F == 0  0.000e+00  1.083e-01   0.000        1
MD.C - MD.F == 0    1.713e-01  1.083e-01   1.581        1
MD.I - MD.H == 0   -1.342e-16  1.083e-01   0.000        1
MD.Mix - MD.H == 0 -3.803e-17  1.083e-01   0.000        1
MD.C - MD.H == 0    1.713e-01  1.083e-01   1.581        1
MD.Mix - MD.I == 0  9.615e-17  1.083e-01   0.000        1
MD.C - MD.I == 0    1.713e-01  1.083e-01   1.581        1
MD.C - MD.Mix == 0  1.713e-01  1.083e-01   1.581        1
(Adjusted p values reported -- bonferroni method)




ASV39

	 Simultaneous Tests for General Linear Hypotheses

Multiple Comparisons of Means: Tukey Contrasts
```

```
Fit: lm(formula = log_cop ~ Treatment, data = dsub)

Linear Hypotheses:
                   Estimate Std. Error t value Pr(>|t|)
MD.H - MD.F == 0     3.2329     0.8403   3.847 0.002488 **
MD.I - MD.F == 0     0.3262     0.8403   0.388 1.000000
MD.Mix - MD.F == 0   1.2666     0.8403   1.507 1.000000
MD.C - MD.F == 0    -0.2620     0.8403  -0.312 1.000000
MD.I - MD.H == 0    -2.9067     0.8403  -3.459 0.008968 **
MD.Mix - MD.H == 0  -1.9663     0.8403  -2.340 0.219441
MD.C - MD.H == 0    -3.4948     0.8403  -4.159 0.000841 ***
MD.Mix - MD.I == 0   0.9404     0.8403   1.119 1.000000
MD.C - MD.I == 0    -0.5882     0.8403  -0.700 1.000000
MD.C - MD.Mix == 0  -1.5286     0.8403  -1.819 0.728865
---
Signif. codes:  0 ‘***’ 0.001 ‘**’ 0.01 ‘*’ 0.05 ‘.’ 0.1 ‘ ’ 1
(Adjusted p values reported -- bonferroni method)
```

ASV394

```
         Simultaneous Tests for General Linear Hypotheses

Multiple Comparisons of Means: Tukey Contrasts
```

```
Fit: lm(formula = log_cop ~ Treatment, data = dsub)

Linear Hypotheses:
                  Estimate Std. Error t value Pr(>|t|)
MD.H - MD.F == 0   -2.186e-01  1.383e-01  -1.581        1
MD.I - MD.F == 0   -2.186e-01  1.383e-01  -1.581        1
MD.Mix - MD.F == 0 -2.186e-01  1.383e-01  -1.581        1
MD.C - MD.F == 0   -2.186e-01  1.383e-01  -1.581        1
MD.I - MD.H == 0    1.665e-16  1.383e-01   0.000        1
MD.Mix - MD.H == 0 -2.776e-17  1.383e-01   0.000        1
MD.C - MD.H == 0    2.776e-17  1.383e-01   0.000        1
MD.Mix - MD.I == 0 -1.943e-16  1.383e-01   0.000        1
MD.C - MD.I == 0   -1.388e-16  1.383e-01   0.000        1
MD.C - MD.Mix == 0  5.551e-17  1.383e-01   0.000        1
(Adjusted p values reported -- bonferroni method)
```

ASV395

```
       Simultaneous Tests for General Linear Hypotheses

Multiple Comparisons of Means: Tukey Contrasts


Fit: lm(formula = log_cop ~ Treatment, data = dsub)

Linear Hypotheses:
```

```
                 Estimate Std. Error t value Pr(>|t|)
MD.H - MD.F == 0    1.577e-17  1.142e-01   0.000        1
MD.I - MD.F == 0    3.205e-17  1.142e-01   0.000        1
MD.Mix - MD.F == 0  1.806e-01  1.142e-01   1.581        1
MD.C - MD.F == 0   -1.227e-17  1.142e-01   0.000        1
MD.I - MD.H == 0    1.628e-17  1.142e-01   0.000        1
MD.Mix - MD.H == 0  1.806e-01  1.142e-01   1.581        1
MD.C - MD.H == 0   -2.804e-17  1.142e-01   0.000        1
MD.Mix - MD.I == 0  1.806e-01  1.142e-01   1.581        1
MD.C - MD.I == 0   -4.432e-17  1.142e-01   0.000        1
MD.C - MD.Mix == 0 -1.806e-01  1.142e-01  -1.581        1
(Adjusted p values reported -- bonferroni method)
```

ASV397

```
       Simultaneous Tests for General Linear Hypotheses

Multiple Comparisons of Means: Tukey Contrasts


Fit: lm(formula = log_cop ~ Treatment, data = dsub)

Linear Hypotheses:
                 Estimate Std. Error t value Pr(>|t|)
MD.H - MD.F == 0    1.099e-16  1.221e-01   0.000        1
MD.I - MD.F == 0    1.602e-16  1.221e-01   0.000        1
```

```
MD.Mix - MD.F == 0  1.930e-01  1.221e-01   1.581        1
MD.C - MD.F == 0   -1.533e-17  1.221e-01   0.000        1
MD.I - MD.H == 0    5.036e-17  1.221e-01   0.000        1
MD.Mix - MD.H == 0  1.930e-01  1.221e-01   1.581        1
MD.C - MD.H == 0   -1.252e-16  1.221e-01   0.000        1
MD.Mix - MD.I == 0  1.930e-01  1.221e-01   1.581        1
MD.C - MD.I == 0   -1.756e-16  1.221e-01   0.000        1
MD.C - MD.Mix == 0 -1.930e-01  1.221e-01  -1.581        1
(Adjusted p values reported -- bonferroni method)
```

ASV4

```
	 Simultaneous Tests for General Linear Hypotheses

Multiple Comparisons of Means: Tukey Contrasts


Fit: lm(formula = log_cop ~ Treatment, data = dsub)

Linear Hypotheses:
             Estimate Std. Error t value Pr(>|t|)
MD.H - MD.F == 0     1.9113     1.1175   1.710   0.9135
MD.I - MD.F == 0    -0.6229     1.1175  -0.557   1.0000
MD.Mix - MD.F == 0   2.5257     1.1175   2.260   0.2672
MD.C - MD.F == 0     0.3154     1.1175   0.282   1.0000
MD.I - MD.H == 0    -2.5342     1.1175  -2.268   0.2623
```

```
MD.Mix - MD.H == 0   0.6144     1.1175   0.550   1.0000
MD.C - MD.H == 0    -1.5959     1.1175  -1.428   1.0000
MD.Mix - MD.I == 0   3.1486     1.1175   2.817   0.0618 .
MD.C - MD.I == 0     0.9383     1.1175   0.840   1.0000
MD.C - MD.Mix == 0  -2.2103     1.1175  -1.978   0.5162
---
Signif. codes:  0 ‘***’ 0.001 ‘**’ 0.01 ‘*’ 0.05 ‘.’ 0.1 ‘ ’ 1
(Adjusted p values reported -- bonferroni method)
```

ASV42

```
	 Simultaneous Tests for General Linear Hypotheses

Multiple Comparisons of Means: Tukey Contrasts


Fit: lm(formula = log_cop ~ Treatment, data = dsub)

Linear Hypotheses:
                      Estimate Std. Error t value Pr(>|t|)
MD.H - MD.F == 0    2.532e-01  4.584e-01   0.552   1.0000
MD.I - MD.F == 0    1.026e-15  4.584e-01   0.000   1.0000
MD.Mix - MD.F == 0  1.473e+00  4.584e-01   3.214   0.0193 *
MD.C - MD.F == 0   -1.030e-16  4.584e-01   0.000   1.0000
MD.I - MD.H == 0   -2.532e-01  4.584e-01  -0.552   1.0000
MD.Mix - MD.H == 0  1.220e+00  4.584e-01   2.662   0.0951 .
```

```
MD.C - MD.H == 0   -2.532e-01  4.584e-01  -0.552   1.0000
MD.Mix - MD.I == 0  1.473e+00  4.584e-01   3.214   0.0193 *
MD.C - MD.I == 0   -1.129e-15  4.584e-01   0.000   1.0000
MD.C - MD.Mix == 0 -1.473e+00  4.584e-01  -3.214   0.0193 *
---
Signif. codes:  0 ‘***’ 0.001 ‘**’ 0.01 ‘*’ 0.05 ‘.’ 0.1 ‘ ’ 1
(Adjusted p values reported -- bonferroni method)
```

ASV43

```
       Simultaneous Tests for General Linear Hypotheses

Multiple Comparisons of Means: Tukey Contrasts


Fit: lm(formula = log_cop ~ Treatment, data = dsub)

Linear Hypotheses:
               Estimate Std. Error t value Pr(>|t|)
MD.H - MD.F == 0   -7.777e-01  4.812e-01  -1.616    1.000
MD.I - MD.F == 0   -5.119e-01  4.812e-01  -1.064    1.000
MD.Mix - MD.F == 0 -7.777e-01  4.812e-01  -1.616    1.000
MD.C - MD.F == 0    5.262e-02  4.812e-01   0.109    1.000
MD.I - MD.H == 0    2.658e-01  4.812e-01   0.552    1.000
MD.Mix - MD.H == 0 -5.551e-16  4.812e-01   0.000    1.000
MD.C - MD.H == 0    8.304e-01  4.812e-01   1.726    0.885
```

```
MD.Mix - MD.I == 0 -2.658e-01  4.812e-01  -0.552    1.000
MD.C - MD.I == 0    5.645e-01  4.812e-01   1.173    1.000
MD.C - MD.Mix == 0  8.304e-01  4.812e-01   1.726    0.885
(Adjusted p values reported -- bonferroni method)
```

ASV44

```
       Simultaneous Tests for General Linear Hypotheses

Multiple Comparisons of Means: Tukey Contrasts


Fit: lm(formula = log_cop ~ Treatment, data = dsub)

Linear Hypotheses:
             Estimate Std. Error t value Pr(>|t|)
MD.H - MD.F == 0     3.0062     0.7424   4.049  0.00124 **
MD.I - MD.F == 0     0.7858     0.7424   1.058  1.00000
MD.Mix - MD.F == 0   0.3941     0.7424   0.531  1.00000
MD.C - MD.F == 0     1.0471     0.7424   1.410  1.00000
MD.I - MD.H == 0    -2.2204     0.7424  -2.991  0.03762 *
MD.Mix - MD.H == 0  -2.6121     0.7424  -3.518  0.00741 **
MD.C - MD.H == 0    -1.9591     0.7424  -2.639  0.10109
MD.Mix - MD.I == 0  -0.3917     0.7424  -0.528  1.00000
MD.C - MD.I == 0     0.2613     0.7424   0.352  1.00000
MD.C - MD.Mix == 0   0.6529     0.7424   0.880  1.00000
```

```
---
Signif. codes:  0 ‘***’ 0.001 ‘**’ 0.01 ‘*’ 0.05 ‘.’ 0.1 ‘ ’ 1
(Adjusted p values reported -- bonferroni method)




ASV45

	 Simultaneous Tests for General Linear Hypotheses

Multiple Comparisons of Means: Tukey Contrasts


Fit: lm(formula = log_cop ~ Treatment, data = dsub)

Linear Hypotheses:
             Estimate Std. Error t value Pr(>|t|)    
MD.H - MD.F == 0     3.3569     0.6842   4.906 5.26e-05 ***
MD.I - MD.F == 0     0.9392     0.6842   1.373 1.000000    
MD.Mix - MD.F == 0   0.3126     0.6842   0.457 1.000000    
MD.C - MD.F == 0     0.6860     0.6842   1.003 1.000000    
MD.I - MD.H == 0    -2.4177     0.6842  -3.534 0.007060 ** 
MD.Mix - MD.H == 0  -3.0443     0.6842  -4.449 0.000294 ***
MD.C - MD.H == 0    -2.6710     0.6842  -3.904 0.002054 ** 
MD.Mix - MD.I == 0  -0.6266     0.6842  -0.916 1.000000    
MD.C - MD.I == 0    -0.2532     0.6842  -0.370 1.000000    
MD.C - MD.Mix == 0   0.3733     0.6842   0.546 1.000000    
---
```

```
Signif. codes:  0 ‘***’ 0.001 ‘**’ 0.01 ‘*’ 0.05 ‘.’ 0.1 ‘ ’ 1
(Adjusted p values reported -- bonferroni method)
```

ASV46

```
	 Simultaneous Tests for General Linear Hypotheses

Multiple Comparisons of Means: Tukey Contrasts


Fit: lm(formula = log_cop ~ Treatment, data = dsub)

Linear Hypotheses:
                    Estimate Std. Error t value Pr(>|t|)
MD.H - MD.F == 0    -1.6317     0.7326  -2.227    0.289
MD.I - MD.F == 0    -0.1753     0.7326  -0.239    1.000
MD.Mix - MD.F == 0  -1.2681     0.7326  -1.731    0.876
MD.C - MD.F == 0    -0.9042     0.7326  -1.234    1.000
MD.I - MD.H == 0     1.4564     0.7326   1.988    0.505
MD.Mix - MD.H == 0   0.3637     0.7326   0.496    1.000
MD.C - MD.H == 0     0.7275     0.7326   0.993    1.000
MD.Mix - MD.I == 0  -1.0927     0.7326  -1.492    1.000
MD.C - MD.I == 0    -0.7289     0.7326  -0.995    1.000
MD.C - MD.Mix == 0   0.3638     0.7326   0.497    1.000
(Adjusted p values reported -- bonferroni method)
```

ASV48

```
	 Simultaneous Tests for General Linear Hypotheses

Multiple Comparisons of Means: Tukey Contrasts


Fit: lm(formula = log_cop ~ Treatment, data = dsub)

Linear Hypotheses:
                   Estimate Std. Error t value Pr(>|t|)
MD.H - MD.F == 0   -1.025e+00  5.203e-01  -1.969    0.526
MD.I - MD.F == 0   -1.025e+00  5.203e-01  -1.969    0.526
MD.Mix - MD.F == 0 -2.469e-01  5.203e-01  -0.474    1.000
MD.C - MD.F == 0   -7.557e-01  5.203e-01  -1.452    1.000
MD.I - MD.H == 0   -1.110e-15  5.203e-01   0.000    1.000
MD.Mix - MD.H == 0  7.778e-01  5.203e-01   1.495    1.000
MD.C - MD.H == 0    2.690e-01  5.203e-01   0.517    1.000
MD.Mix - MD.I == 0  7.778e-01  5.203e-01   1.495    1.000
MD.C - MD.I == 0    2.690e-01  5.203e-01   0.517    1.000
MD.C - MD.Mix == 0 -5.088e-01  5.203e-01  -0.978    1.000
(Adjusted p values reported -- bonferroni method)
```

ASV5

```
	 Simultaneous Tests for General Linear Hypotheses

Multiple Comparisons of Means: Tukey Contrasts


Fit: lm(formula = log_cop ~ Treatment, data = dsub)

Linear Hypotheses:
                  Estimate Std. Error t value Pr(>|t|)
MD.H - MD.F == 0    1.46242    0.80075   1.826    0.718
MD.I - MD.F == 0    0.67586    0.80075   0.844    1.000
MD.Mix - MD.F == 0  0.92701    0.80075   1.158    1.000
MD.C - MD.F == 0    0.99985    0.80075   1.249    1.000
MD.I - MD.H == 0   -0.78656    0.80075  -0.982    1.000
MD.Mix - MD.H == 0 -0.53541    0.80075  -0.669    1.000
MD.C - MD.H == 0   -0.46257    0.80075  -0.578    1.000
MD.Mix - MD.I == 0  0.25115    0.80075   0.314    1.000
MD.C - MD.I == 0    0.32399    0.80075   0.405    1.000
MD.C - MD.Mix == 0  0.07284    0.80075   0.091    1.000
(Adjusted p values reported -- bonferroni method)
```

ASV50

```
	 Simultaneous Tests for General Linear Hypotheses
```

```
Multiple Comparisons of Means: Tukey Contrasts


Fit: lm(formula = log_cop ~ Treatment, data = dsub)

Linear Hypotheses:
               Estimate Std. Error t value Pr(>|t|)
MD.H - MD.F == 0    1.039e-15  4.876e-01   0.000 1.000000
MD.I - MD.F == 0    1.923e-15  4.876e-01   0.000 1.000000
MD.Mix - MD.F == 0  2.007e+00  4.876e-01   4.115 0.000983 ***
MD.C - MD.F == 0   -2.699e-16  4.876e-01   0.000 1.000000
MD.I - MD.H == 0    8.837e-16  4.876e-01   0.000 1.000000
MD.Mix - MD.H == 0  2.007e+00  4.876e-01   4.115 0.000983 ***
MD.C - MD.H == 0   -1.309e-15  4.876e-01   0.000 1.000000
MD.Mix - MD.I == 0  2.007e+00  4.876e-01   4.115 0.000983 ***
MD.C - MD.I == 0   -2.193e-15  4.876e-01   0.000 1.000000
MD.C - MD.Mix == 0 -2.007e+00  4.876e-01  -4.115 0.000983 ***
---
Signif. codes:  0 ‘***’ 0.001 ‘**’ 0.01 ‘*’ 0.05 ‘.’ 0.1 ‘ ’ 1
(Adjusted p values reported -- bonferroni method)



ASV53

      Simultaneous Tests for General Linear Hypotheses
```

```
Multiple Comparisons of Means: Tukey Contrasts


Fit: lm(formula = log_cop ~ Treatment, data = dsub)

Linear Hypotheses:
                   Estimate Std. Error t value Pr(>|t|)
MD.H - MD.F == 0   -1.094e+00  4.768e-01  -2.294    0.246
MD.I - MD.F == 0   -1.094e+00  4.768e-01  -2.294    0.246
MD.Mix - MD.F == 0 -4.263e-01  4.768e-01  -0.894    1.000
MD.C - MD.F == 0   -1.094e+00  4.768e-01  -2.294    0.246
MD.I - MD.H == 0    6.661e-16  4.768e-01   0.000    1.000
MD.Mix - MD.H == 0  6.676e-01  4.768e-01   1.400    1.000
MD.C - MD.H == 0    2.220e-16  4.768e-01   0.000    1.000
MD.Mix - MD.I == 0  6.676e-01  4.768e-01   1.400    1.000
MD.C - MD.I == 0   -4.441e-16  4.768e-01   0.000    1.000
MD.C - MD.Mix == 0 -6.676e-01  4.768e-01  -1.400    1.000
(Adjusted p values reported -- bonferroni method)



ASV54

	 Simultaneous Tests for General Linear Hypotheses

Multiple Comparisons of Means: Tukey Contrasts
```

```
Fit: lm(formula = log_cop ~ Treatment, data = dsub)

Linear Hypotheses:
                  Estimate Std. Error t value Pr(>|t|)
MD.H - MD.F == 0    1.345658   0.528879   2.544   0.1300
MD.I - MD.F == 0   -0.090065   0.528879  -0.170   1.0000
MD.Mix - MD.F == 0  0.013383   0.528879   0.025   1.0000
MD.C - MD.F == 0   -0.001627   0.528879  -0.003   1.0000
MD.I - MD.H == 0   -1.435723   0.528879  -2.715   0.0823 .
MD.Mix - MD.H == 0 -1.332275   0.528879  -2.519   0.1390
MD.C - MD.H == 0   -1.347285   0.528879  -2.547   0.1290
MD.Mix - MD.I == 0  0.103448   0.528879   0.196   1.0000
MD.C - MD.I == 0    0.088438   0.528879   0.167   1.0000
MD.C - MD.Mix == 0 -0.015010   0.528879  -0.028   1.0000
---
Signif. codes:  0 ‘***’ 0.001 ‘**’ 0.01 ‘*’ 0.05 ‘.’ 0.1 ‘ ’ 1
(Adjusted p values reported -- bonferroni method)
```

ASV55

```
	 Simultaneous Tests for General Linear Hypotheses

Multiple Comparisons of Means: Tukey Contrasts


Fit: lm(formula = log_cop ~ Treatment, data = dsub)
```

```
Linear Hypotheses:
                     Estimate Std. Error t value Pr(>|t|)
MD.H - MD.F == 0   -0.93795    0.78118  -1.201   1.0000
MD.I - MD.F == 0    1.25913    0.78118   1.612   1.0000
MD.Mix - MD.F == 0  0.06849    0.78118   0.088   1.0000
MD.C - MD.F == 0    0.18546    0.78118   0.237   1.0000
MD.I - MD.H == 0    2.19708    0.78118   2.813   0.0627 .
MD.Mix - MD.H == 0  1.00644    0.78118   1.288   1.0000
MD.C - MD.H == 0    1.12341    0.78118   1.438   1.0000
MD.Mix - MD.I == 0 -1.19064    0.78118  -1.524   1.0000
MD.C - MD.I == 0   -1.07367    0.78118  -1.374   1.0000
MD.C - MD.Mix == 0  0.11697    0.78118   0.150   1.0000
---
Signif. codes:  0 ‘***’ 0.001 ‘**’ 0.01 ‘*’ 0.05 ‘.’ 0.1 ‘ ’ 1
(Adjusted p values reported -- bonferroni method)
```

ASV58

```
       Simultaneous Tests for General Linear Hypotheses

Multiple Comparisons of Means: Tukey Contrasts


Fit: lm(formula = log_cop ~ Treatment, data = dsub)
```

```
Linear Hypotheses:
                    Estimate Std. Error t value Pr(>|t|)
MD.H - MD.F == 0     0.82082    0.64481   1.273    1.000
MD.I - MD.F == 0     1.21280    0.64481   1.881    0.639
MD.Mix - MD.F == 0   0.06958    0.64481   0.108    1.000
MD.C - MD.F == 0    -0.29628    0.64481  -0.459    1.000
MD.I - MD.H == 0     0.39198    0.64481   0.608    1.000
MD.Mix - MD.H == 0  -0.75124    0.64481  -1.165    1.000
MD.C - MD.H == 0    -1.11710    0.64481  -1.732    0.873
MD.Mix - MD.I == 0  -1.14322    0.64481  -1.773    0.803
MD.C - MD.I == 0    -1.50908    0.64481  -2.340    0.219
MD.C - MD.Mix == 0  -0.36586    0.64481  -0.567    1.000
(Adjusted p values reported -- bonferroni method)
```

ASV59

```
         Simultaneous Tests for General Linear Hypotheses

Multiple Comparisons of Means: Tukey Contrasts


Fit: lm(formula = log_cop ~ Treatment, data = dsub)

Linear Hypotheses:
                    Estimate Std. Error t value Pr(>|t|)
MD.H - MD.F == 0     2.20292    0.64142   3.434   0.0097 **
```

```
MD.I - MD.F == 0    1.91820    0.64142   2.991   0.0377 *
MD.Mix - MD.F == 0  0.44930    0.64142   0.700   1.0000
MD.C - MD.F == 0    1.83392    0.64142   2.859   0.0550 .
MD.I - MD.H == 0   -0.28472    0.64142  -0.444   1.0000
MD.Mix - MD.H == 0 -1.75362    0.64142  -2.734   0.0780 .
MD.C - MD.H == 0   -0.36901    0.64142  -0.575   1.0000
MD.Mix - MD.I == 0 -1.46890    0.64142  -2.290   0.2483
MD.C - MD.I == 0   -0.08429    0.64142  -0.131   1.0000
MD.C - MD.Mix == 0  1.38461    0.64142   2.159   0.3407
---
Signif. codes:  0 ‘***’ 0.001 ‘**’ 0.01 ‘*’ 0.05 ‘.’ 0.1 ‘ ’ 1
(Adjusted p values reported -- bonferroni method)
```

ASV6

```
	 Simultaneous Tests for General Linear Hypotheses

Multiple Comparisons of Means: Tukey Contrasts


Fit: lm(formula = log_cop ~ Treatment, data = dsub)

Linear Hypotheses:
             Estimate Std. Error t value Pr(>|t|)
MD.H - MD.F == 0     1.1622     0.9400   1.236 1.000000
MD.I - MD.F == 0    -0.3555     0.9400  -0.378 1.000000
```

```
MD.Mix - MD.F == 0   2.5007     0.9400   2.660 0.095465 .
MD.C - MD.F == 0     3.8171     0.9400   4.060 0.001191 **
MD.I - MD.H == 0    -1.5177     0.9400  -1.614 1.000000
MD.Mix - MD.H == 0   1.3385     0.9400   1.424 1.000000
MD.C - MD.H == 0     2.6549     0.9400   2.824 0.060682 .
MD.Mix - MD.I == 0   2.8562     0.9400   3.038 0.032726 *
MD.C - MD.I == 0     4.1726     0.9400   4.439 0.000306 ***
MD.C - MD.Mix == 0   1.3164     0.9400   1.400 1.000000
---
Signif. codes:  0 ‘***’ 0.001 ‘**’ 0.01 ‘*’ 0.05 ‘.’ 0.1 ‘ ’ 1
(Adjusted p values reported -- bonferroni method)
```

ASV60

```
	 Simultaneous Tests for General Linear Hypotheses

Multiple Comparisons of Means: Tukey Contrasts


Fit: lm(formula = log_cop ~ Treatment, data = dsub)

Linear Hypotheses:
               Estimate Std. Error t value Pr(>|t|)
MD.H - MD.F == 0   -9.687e-01  5.690e-01  -1.703    0.928
MD.I - MD.F == 0    5.242e-01  5.690e-01   0.921    1.000
MD.Mix - MD.F == 0 -9.687e-01  5.690e-01  -1.703    0.928
```

```
MD.C - MD.F == 0   -6.698e-01  5.690e-01  -1.177    1.000
MD.I - MD.H == 0    1.493e+00  5.690e-01   2.624    0.105
MD.Mix - MD.H == 0 -5.551e-16  5.690e-01   0.000    1.000
MD.C - MD.H == 0    2.989e-01  5.690e-01   0.525    1.000
MD.Mix - MD.I == 0 -1.493e+00  5.690e-01  -2.624    0.105
MD.C - MD.I == 0   -1.194e+00  5.690e-01  -2.099    0.392
MD.C - MD.Mix == 0  2.989e-01  5.690e-01   0.525    1.000
(Adjusted p values reported -- bonferroni method)
```

ASV61

```
       Simultaneous Tests for General Linear Hypotheses

Multiple Comparisons of Means: Tukey Contrasts


Fit: lm(formula = log_cop ~ Treatment, data = dsub)

Linear Hypotheses:
              Estimate Std. Error t value Pr(>|t|)
MD.H - MD.F == 0    1.368012   0.738105   1.853   0.6776
MD.I - MD.F == 0    0.937897   0.738105   1.271   1.0000
MD.Mix - MD.F == 0  2.056824   0.738105   2.787   0.0674 .
MD.C - MD.F == 0   -0.007564   0.738105  -0.010   1.0000
MD.I - MD.H == 0   -0.430115   0.738105  -0.583   1.0000
MD.Mix - MD.H == 0  0.688812   0.738105   0.933   1.0000
```

```
MD.C - MD.H == 0   -1.375576   0.738105  -1.864   0.6628
MD.Mix - MD.I == 0  1.118927   0.738105   1.516   1.0000
MD.C - MD.I == 0   -0.945461   0.738105  -1.281   1.0000
MD.C - MD.Mix == 0 -2.064388   0.738105  -2.797   0.0655 .
---
Signif. codes:  0 ‘***’ 0.001 ‘**’ 0.01 ‘*’ 0.05 ‘.’ 0.1 ‘ ’ 1
(Adjusted p values reported -- bonferroni method)
```

ASV65

```
	 Simultaneous Tests for General Linear Hypotheses

Multiple Comparisons of Means: Tukey Contrasts


Fit: lm(formula = log_cop ~ Treatment, data = dsub)

Linear Hypotheses:
               Estimate Std. Error t value Pr(>|t|)
MD.H - MD.F == 0    1.523e+00  4.331e-01   3.517  0.00744 **
MD.I - MD.F == 0   -9.348e-18  4.331e-01   0.000  1.00000
MD.Mix - MD.F == 0 -5.625e-18  4.331e-01   0.000  1.00000
MD.C - MD.F == 0   -2.301e-16  4.331e-01   0.000  1.00000
MD.I - MD.H == 0   -1.523e+00  4.331e-01  -3.517  0.00744 **
MD.Mix - MD.H == 0 -1.523e+00  4.331e-01  -3.517  0.00744 **
MD.C - MD.H == 0   -1.523e+00  4.331e-01  -3.517  0.00744 **
```

```
MD.Mix - MD.I == 0  3.723e-18  4.331e-01   0.000  1.00000
MD.C - MD.I == 0   -2.207e-16  4.331e-01   0.000  1.00000
MD.C - MD.Mix == 0 -2.244e-16  4.331e-01   0.000  1.00000
---
Signif. codes:  0 ‘***’ 0.001 ‘**’ 0.01 ‘*’ 0.05 ‘.’ 0.1 ‘ ’ 1
(Adjusted p values reported -- bonferroni method)
```

ASV7

```
	 Simultaneous Tests for General Linear Hypotheses

Multiple Comparisons of Means: Tukey Contrasts


Fit: lm(formula = log_cop ~ Treatment, data = dsub)

Linear Hypotheses:
                   Estimate Std. Error t value Pr(>|t|)
MD.H - MD.F == 0    0.82359    0.60790   1.355    1.000
MD.I - MD.F == 0   -0.04974    0.60790  -0.082    1.000
MD.Mix - MD.F == 0  0.23947    0.60790   0.394    1.000
MD.C - MD.F == 0    1.35829    0.60790   2.234    0.284
MD.I - MD.H == 0   -0.87334    0.60790  -1.437    1.000
MD.Mix - MD.H == 0 -0.58412    0.60790  -0.961    1.000
MD.C - MD.H == 0    0.53470    0.60790   0.880    1.000
MD.Mix - MD.I == 0  0.28921    0.60790   0.476    1.000
```

```
MD.C - MD.I == 0    1.40804    0.60790   2.316    0.233
MD.C - MD.Mix == 0  1.11882    0.60790   1.840    0.697
(Adjusted p values reported -- bonferroni method)
```

ASV71

```
         Simultaneous Tests for General Linear Hypotheses

Multiple Comparisons of Means: Tukey Contrasts


Fit: lm(formula = log_cop ~ Treatment, data = dsub)

Linear Hypotheses:
             Estimate Std. Error t value Pr(>|t|)
MD.H - MD.F == 0   -0.35654    0.72098  -0.495        1
MD.I - MD.F == 0   -0.15096    0.72098  -0.209        1
MD.Mix - MD.F == 0 -0.04880    0.72098  -0.068        1
MD.C - MD.F == 0   -0.08518    0.72098  -0.118        1
MD.I - MD.H == 0    0.20558    0.72098   0.285        1
MD.Mix - MD.H == 0  0.30773    0.72098   0.427        1
MD.C - MD.H == 0    0.27135    0.72098   0.376        1
MD.Mix - MD.I == 0  0.10215    0.72098   0.142        1
MD.C - MD.I == 0    0.06577    0.72098   0.091        1
MD.C - MD.Mix == 0 -0.03638    0.72098  -0.050        1
(Adjusted p values reported -- bonferroni method)
```

ASV79

```
	 Simultaneous Tests for General Linear Hypotheses

Multiple Comparisons of Means: Tukey Contrasts


Fit: lm(formula = log_cop ~ Treatment, data = dsub)

Linear Hypotheses:
                  Estimate Std. Error t value Pr(>|t|)
MD.H - MD.F == 0   -3.169e-01  4.135e-01  -0.766    1.000
MD.I - MD.F == 0    7.389e-01  4.135e-01   1.787    0.780
MD.Mix - MD.F == 0 -3.169e-01  4.135e-01  -0.766    1.000
MD.C - MD.F == 0   -3.169e-01  4.135e-01  -0.766    1.000
MD.I - MD.H == 0    1.056e+00  4.135e-01   2.553    0.127
MD.Mix - MD.H == 0 -2.776e-16  4.135e-01   0.000    1.000
MD.C - MD.H == 0   -3.331e-16  4.135e-01   0.000    1.000
MD.Mix - MD.I == 0 -1.056e+00  4.135e-01  -2.553    0.127
MD.C - MD.I == 0   -1.056e+00  4.135e-01  -2.553    0.127
MD.C - MD.Mix == 0 -5.551e-17  4.135e-01   0.000    1.000
(Adjusted p values reported -- bonferroni method)
```

ASV8

```
	 Simultaneous Tests for General Linear Hypotheses

Multiple Comparisons of Means: Tukey Contrasts


Fit: lm(formula = log_cop ~ Treatment, data = dsub)

Linear Hypotheses:
                  Estimate Std. Error t value Pr(>|t|)
MD.H - MD.F == 0    -4.0480     1.0932  -3.703  0.00405 **
MD.I - MD.F == 0    -2.6579     1.0932  -2.431  0.17430
MD.Mix - MD.F == 0  -1.7263     1.0932  -1.579  1.00000
MD.C - MD.F == 0    -0.9059     1.0932  -0.829  1.00000
MD.I - MD.H == 0     1.3901     1.0932   1.272  1.00000
MD.Mix - MD.H == 0   2.3217     1.0932   2.124  0.36983
MD.C - MD.H == 0     3.1421     1.0932   2.874  0.05267 .
MD.Mix - MD.I == 0   0.9316     1.0932   0.852  1.00000
MD.C - MD.I == 0     1.7520     1.0932   1.603  1.00000
MD.C - MD.Mix == 0   0.8204     1.0932   0.750  1.00000
---
Signif. codes:  0 ‘***’ 0.001 ‘**’ 0.01 ‘*’ 0.05 ‘.’ 0.1 ‘ ’ 1
(Adjusted p values reported -- bonferroni method)
```

ASV81

```
	 Simultaneous Tests for General Linear Hypotheses

Multiple Comparisons of Means: Tukey Contrasts


Fit: lm(formula = log_cop ~ Treatment, data = dsub)

Linear Hypotheses:
                  Estimate Std. Error t value Pr(>|t|)
MD.H - MD.F == 0    4.175e-01  2.641e-01   1.581        1
MD.I - MD.F == 0    6.502e-18  2.641e-01   0.000        1
MD.Mix - MD.F == 0  9.712e-20  2.641e-01   0.000        1
MD.C - MD.F == 0    1.038e-17  2.641e-01   0.000        1
MD.I - MD.H == 0   -4.175e-01  2.641e-01  -1.581        1
MD.Mix - MD.H == 0 -4.175e-01  2.641e-01  -1.581        1
MD.C - MD.H == 0   -4.175e-01  2.641e-01  -1.581        1
MD.Mix - MD.I == 0 -6.405e-18  2.641e-01   0.000        1
MD.C - MD.I == 0    3.876e-18  2.641e-01   0.000        1
MD.C - MD.Mix == 0  1.028e-17  2.641e-01   0.000        1
(Adjusted p values reported -- bonferroni method)
```

ASV82

```
	 Simultaneous Tests for General Linear Hypotheses
```

Multiple Comparisons of Means: Tukey Contrasts

Fit: lm(formula = log_cop ~ Treatment, data = dsub)

Linear Hypotheses:

	Estimate	Std. Error	t value	Pr(>|t|)
MD.H - MD.F == 0	-0.5710	0.4970	-1.149	1
MD.I - MD.F == 0	-0.3118	0.4970	-0.627	1
MD.Mix - MD.F == 0	-0.4584	0.4970	-0.922	1
MD.C - MD.F == 0	-0.8128	0.4970	-1.636	1
MD.I - MD.H == 0	0.2592	0.4970	0.521	1
MD.Mix - MD.H == 0	0.1126	0.4970	0.227	1
MD.C - MD.H == 0	-0.2418	0.4970	-0.487	1
MD.Mix - MD.I == 0	-0.1466	0.4970	-0.295	1
MD.C - MD.I == 0	-0.5010	0.4970	-1.008	1
MD.C - MD.Mix == 0	-0.3544	0.4970	-0.713	1

(Adjusted p values reported -- bonferroni method)

ASV83

Simultaneous Tests for General Linear Hypotheses

Multiple Comparisons of Means: Tukey Contrasts

```
Fit: lm(formula = log_cop ~ Treatment, data = dsub)

Linear Hypotheses:
                   Estimate Std. Error t value Pr(>|t|)
MD.H - MD.F == 0   -3.569e-16  2.426e-01   0.000        1
MD.I - MD.F == 0    3.836e-01  2.426e-01   1.581        1
MD.Mix - MD.F == 0 -1.347e-16  2.426e-01   0.000        1
MD.C - MD.F == 0    2.453e-18  2.426e-01   0.000        1
MD.I - MD.H == 0    3.836e-01  2.426e-01   1.581        1
MD.Mix - MD.H == 0  2.221e-16  2.426e-01   0.000        1
MD.C - MD.H == 0    3.593e-16  2.426e-01   0.000        1
MD.Mix - MD.I == 0 -3.836e-01  2.426e-01  -1.581        1
MD.C - MD.I == 0   -3.836e-01  2.426e-01  -1.581        1
MD.C - MD.Mix == 0  1.372e-16  2.426e-01   0.000        1
(Adjusted p values reported -- bonferroni method)
```

ASV85

```
	 Simultaneous Tests for General Linear Hypotheses

Multiple Comparisons of Means: Tukey Contrasts


Fit: lm(formula = log_cop ~ Treatment, data = dsub)
```

```
Linear Hypotheses:
                     Estimate Std. Error t value Pr(>|t|)
MD.H - MD.F == 0    4.055e-01  3.613e-01   1.122        1
MD.I - MD.F == 0   -3.686e-16  3.613e-01   0.000        1
MD.Mix - MD.F == 0  0.000e+00  3.613e-01   0.000        1
MD.C - MD.F == 0    4.023e-01  3.613e-01   1.114        1
MD.I - MD.H == 0   -4.055e-01  3.613e-01  -1.122        1
MD.Mix - MD.H == 0 -4.055e-01  3.613e-01  -1.122        1
MD.C - MD.H == 0   -3.159e-03  3.613e-01  -0.009        1
MD.Mix - MD.I == 0  3.686e-16  3.613e-01   0.000        1
MD.C - MD.I == 0    4.023e-01  3.613e-01   1.114        1
MD.C - MD.Mix == 0  4.023e-01  3.613e-01   1.114        1
(Adjusted p values reported -- bonferroni method)
```

ASV9

```
	 Simultaneous Tests for General Linear Hypotheses

Multiple Comparisons of Means: Tukey Contrasts


Fit: lm(formula = log_cop ~ Treatment, data = dsub)

Linear Hypotheses:
                   Estimate Std. Error t value Pr(>|t|)
MD.H - MD.F == 0    -2.2875     0.9964  -2.296   0.2449
```

```
MD.I - MD.F == 0    -1.5044     0.9964  -1.510   1.0000
MD.Mix - MD.F == 0   0.7481     0.9964   0.751   1.0000
MD.C - MD.F == 0    -0.2878     0.9964  -0.289   1.0000
MD.I - MD.H == 0     0.7831     0.9964   0.786   1.0000
MD.Mix - MD.H == 0   3.0356     0.9964   3.047   0.0319 *
MD.C - MD.H == 0     1.9997     0.9964   2.007   0.4836
MD.Mix - MD.I == 0   2.2525     0.9964   2.261   0.2668
MD.C - MD.I == 0     1.2167     0.9964   1.221   1.0000
MD.C - MD.Mix == 0  -1.0359     0.9964  -1.040   1.0000
---
Signif. codes:  0 ‘***’ 0.001 ‘**’ 0.01 ‘*’ 0.05 ‘.’ 0.1 ‘ ’ 1
(Adjusted p values reported -- bonferroni method)
```

ASV99

```
	 Simultaneous Tests for General Linear Hypotheses

Multiple Comparisons of Means: Tukey Contrasts


Fit: lm(formula = log_cop ~ Treatment, data = dsub)

Linear Hypotheses:
               Estimate Std. Error t value Pr(>|t|)
MD.H - MD.F == 0   -2.777e-01  3.458e-01  -0.803    1.000
MD.I - MD.F == 0   -2.777e-01  3.458e-01  -0.803    1.000
```

```
MD.Mix - MD.F == 0 -2.777e-01  3.458e-01  -0.803    1.000
MD.C - MD.F == 0    4.116e-01  3.458e-01   1.190    1.000
MD.I - MD.H == 0   -8.327e-16  3.458e-01   0.000    1.000
MD.Mix - MD.H == 0 -7.216e-16  3.458e-01   0.000    1.000
MD.C - MD.H == 0    6.893e-01  3.458e-01   1.994    0.498
MD.Mix - MD.I == 0  1.110e-16  3.458e-01   0.000    1.000
MD.C - MD.I == 0    6.893e-01  3.458e-01   1.994    0.498
MD.C - MD.Mix == 0  6.893e-01  3.458e-01   1.994    0.498
(Adjusted p values reported -- bonferroni method)
```
