# Supplementary material for: Mild chronic exposure to pesticides alters physiological markers of honey bee health without perturbing the core gut microbiota
Source: Sci Rep. 2022 Mar 11;12:4281. doi: 10.1038/s41598-022-08009-2 (PMC8917129; doi:10.1038/s41598-022-08009-2)
Supplement: Supplementary file 8 — Supplementary Tables. [file 41598_2022_8009_MOESM8_ESM.pdf]

Table S3. Effects of pesticide combinations and gut colonization on the physiological state of newly emerged honey bees

Microbiota-depleted and colonized honeybees were generated. Two days after their emergence, honeybees were fed for five days sterile sucrose solutions containing no pesticides (Control) or imidacloprid (Insecticide), glyphosate (Herbicide), difenoconazole (Fungicide) alone or as a ternary mixture (Mix) at 0.1 µg/L. GST, G6PDH and LDH were measured in the head (h), abdomen (a) and midgut (m). ALP and POx were chosen as specific markers in the midgut (m). On the 7th day, 7 samples of 3 tissues were collected in each treatment. For each treatment, the data represent the mean values of enzymatic activities expressed in milli absorbance units of per minute and per mg of tissue (mAU.min^{-1}.mg of tissue^{-1}) ± standard deviations (SD). ANOVA or Kruskal-Wallis tests were applied to detect significant differences between treatments. Treatments with different letters are significantly different ($p < 0.05$). Red arrows indicate an increase (↑) and a decrease (↓) in the enzymatic activity of colonized honey bees exposed to pesticides relative to their control (CL.Control). Blue arrows indicate an increase (↑) and a decrease (↓) in the enzymatic activity of microbiota-depleted honey bees exposed to pesticides relative to their control (MD.Control).

	CL					MD				
	Activity of physiological markers (mAU.min^{-1}.mg of tissue^{-1})					Activity of physiological markers (mAU.min^{-1}.mg of tissue^{-1})				
	CL.Control	CL.Insecticide	CL.Fungicide	CL.Herbicide	CL.Mix	MD.Control	MD.Insecticide	MD.Fungicide	MD.Herbicide	MD.Mix
GST$_{(h)}$	100,196 ± 11,811	102,654 ± 4,968	103,080 ± 6,262	107,650 ± 3,301	111,278 ± 7,867	105,116 ± 12,334	101,222 ± 6,872	99,842 ± 7,812	97,362 ± 13,622	103,620 ± 12,387
G6PDH$_{(h)}$	7,401 ± 2,186 ^{ab}	7,804 ± 0,808 ^{ab}	8,457 ± 1,673 ^{ab}	8,787 ± 0,637 ^{b}	8,130 ± 1,353 ^{ab}	7,728 ± 1,377 ^{ab}	9,116 ± 1,183 ^{b}	7,744 ± 1,381 ^{ab}	6,350 ± 1,170 ^{a}	7,442 ± 1,336 ^{ab}
LDH$_{(h)}$	8,605 ± 3,930 ^{de}	7,625 ± 1,654 ^{e}	13,638 ± 3,333 ^{bc}↑	9,362 ± 2,145 ^{de}	8,328 ± 0,664 ^{de}	12,493 ± 5,812 ^{cd}	14,652 ± 2,487 ^{bc}	22,081 ± 2,942 ^{a} ↑	16,848 ± 3,750 ^{ab}↑	22,606 ± 3,767 ^{a}↑
GST$_{(a)}$	76,907 ± 17,688 ^{abcd}	63,622 ± 15,387 ^{cd}	83,433 ± 12,188 ^{abc}	37,766 ± 7,316 ^{d}	113,362 ± 28,068 ^{a}	95,244 ± 19,998 ^{abc}	81,410 ± 21,713 ^{abc}	100,440 ± 27,080 ^{ab}	67,387 ± 21,651 ^{bcd}	69,943 ± 17,983 ^{bcd}
G6PDH$_{(a)}$	23,034 ± 4,770	25,270 ± 9,206	25,991 ± 7,705	20,724 ± 6,792	28,092 ± 15,750	34,531 ± 11,958	30,678 ± 11,400	28,079 ± 12,866	25,357 ± 10,428	26,499 ± 8,217
LDH$_{(a)}$	4,766 ± 1,146 ^{abcd}	5,569 ± 2,882 ^{abc}	7,137 ± 3,890 ^{ab}	7,675 ± 2,789 ^{a}	6,000 ± 1,207 ^{ab}	3,667 ± 2,157 ^{cd}	3,005 ± 1,123 ^{d}	6,319 ± 1,478 ^{ab}↑	2,870 ± 1,353 ^{cd}	4,204 ± 1,914 ^{bcd}
GST$_{(m)}$	18,783 ± 2,548 ^{ab}	22,895 ± 11,099 ^{ab}	11,664 ± 2,141 ^{cd}↓	26,742 ± 8,207 ^{a}	15,795 ± 6,201 ^{bcd}	9,261 ± 3,999 ^{d}	16,536 ± 5,324 ^{bcd}	17,616 ± 6,465 ^{abc}↑	18,459 ± 9,137 ^{abc}↑	20,104 ± 4,893 ^{ab}↑
G6PDH$_{(m)}$	1,892 ± 0,936	4,277 ± 1,096	3,374 ± 1,717	6,128 ± 2,655	5,457 ± 2,126	6,064 ± 2,722	6,277 ± 3,817	5,515 ± 2,793	6,213 ± 2,191	4,319 ± 2,738
LDH$_{(m)}$	3,229 ± 0,956	2,833 ± 1,653	2,381 ± 0,630	4,772 ± 0,916	2,710 ± 1,219	2,553 ± 1,042	2,006 ± 0,682	4,191 ± 1,328	3,100 ± 2,456	3,457 ± 2,140
ALP$_{(m)}$	10,650 ± 2,733 ^{ab}	9,659 ± 2,547 ^{ab}	10,626 ± 1,093 ^{ab}	7,949 ± 0,519 ^{b}	13,419 ± 2,858 ^{a}	11,095 ± 1,968 ^{ab}	11,241 ± 3,408 ^{ab}	15,204 ± 4,129 ^{a}	10,532 ± 3,491 ^{ab}	12,023 ± 3,983 ^{ab}
POx$_{(m)}$	18,720 ± 5,220 ^{ab}	17,467 ± 7,882 ^{ab}	21,278 ± 3,661 ^{ab}	25,978 ± 12,079 ^{ab}	22,427 ± 5,183 ^{ab}	29,154 ± 7,654 ^{ab}	19,543 ± 7,005 ^{ab}	33,236 ± 10,748 ^{b}	14,281 ± 8,520 ^{a}	21,442 ± 9,915 ^{ab}

Table S4A. Effect of pesticide treatments on the physiological state of colonized honey bees

After emergence, honey bees were colonized or not from the gut homogenate. Two days after their emergence, honeybees were fed for five days sterile sucrose solutions containing no pesticides (Control) or imidacloprid (Insecticide), glyphosate (Herbicide), difenoconazole (Fungicide) alone or as a ternary mixture (Mix) at 0.1 µg/L. The enzymatic activities were compared between colonized (CL) honey bees exposed to the different pesticides and the control (CL.Control). Three groups were distinguished: (i) Enzymes exhibiting an activity similar to that of the control (No modulation). (ii) Enzymes exhibiting an activity higher than that of the control in at least one pesticide treatment. (iii) Enzymes exhibiting an activity lower than that of the control in at least one pesticide treatment.

No modulation	Increase of activity	Decrease of activity
Head GST	Head LDH (CL.Fungicide ↑)	Midgut GST (CL.Fungicide ↓)
Head G6PDH	-	-
Abdomen GST	-	-
Abdomen G6PDH	-	-
Abdomen LDH	-	-
Midgut G6PDH	-	-
Midgut LDH	-	-
Midgut ALP	-	-
Midgut POx	-	-

Table S4B. Effect of pesticide treatments on the physiological state of microbiota-depleted honeybees

After emergence, honey bees were colonized or not from the gut homogenate. Two days after their emergence, honeybees were fed for five days sterile sucrose solutions containing no pesticides (Control) or imidacloprid (Insecticide), glyphosate (Herbicide), difenoconazole (Fungicide) alone or as a ternary mixture (Mix) at 0.1 µg/L. The enzymatic activities were compared between microbiota-depleted honey bees exposed to the different pesticides and the control (MD.Control). Two groups were distinguished: (i) Enzymes exhibiting an activity similar to that of the control. (ii) Enzymes exhibiting an activity higher than that of the control.

No modulation	Increase of enzymatic activity
Head GST	Head LDH: (MD.Fungicide ↑, MD.Herbicide ↑, MD.Mix ↑)
Head G6PDH	Abdomen LDH: (MD.Fungicide ↑)
Abdomen GST	Midgut GST: (MD.Fungicide ↑, MD.Herbicide ↑, MD.Mix ↑)
Abdomen G6PDH	-
Midgut G6PDH	-
Midgut LDH	-
Midgut ALP	-
Midgut POx	-

Table S5. Global effect of gut colonization on physiological markers

After emergence, honey bees were colonized or not from the gut homogenate. Two days after their emergence, honeybees were fed for five days sterile sucrose solutions containing no pesticides (Control) or imidacloprid (Insecticide), glyphosate (Herbicide), difenoconazole (Fungicide) alone or as a ternary mixture (Mix) at 0.1 µg/L. The enzymatic activities of the physiological markers were compared between microbiota-depleted (MD) and colonized (CL) honey bee exposed to the same pesticides.

Activity higher in colonized bees than microbiota-depleted bees	Activity lower in colonized bees than microbiota-depleted bees
**Head G6PDH**<br>- CL.Herbicide > MD.Herbicide	**Head LDH**<br>- CL.Insecticide < MD.Insecticide<br>- CL.Fungicide < MD.Fungicide<br>- CL.Herbicide < MD.Herbicide<br>- CL.Mix < MD.Mix
**Abdomen GST**<br>- CL.Mix > MD.Mix	-
**Abdomen LDH**<br>- CL.Insecticide > MD.Insecticide<br>- CL.Herbicide > MD.Herbicide	-
**Midgut GST**<br>- CL.Control > MD.Control	-
